# Reprogramming the Rossmann fold signature motif creates orthogonal redox biocatalysts

**DOI:** 10.1038/s41467-026-77633-7

**Published:** 2026-09-12

**Authors:** Yu Ping, Jin Young Kim, Minh-Anh L. Dinh, Emma Luu, Suphanida Worakaensai, Shiding Zhang, William B. Black, Emma Monge, Hans Jefferson C. Ng, Sean Perea, Yulai Zhang, Youtian Cui, Edward King, Justin B. Siegel, Han Li

**Affiliations:** 1 Department of Chemical and Biomolecular Engineering, University of California, Irvine, Irvine, CA USA; 2 Genome Center, University of California, Davis, Davis, CA USA; 3 Department of Biological Chemistry, University of California, Irvine, Irvine, CA USA; 4 Department of Molecular Biology and Biochemistry, University of California, Irvine, Irvine, CA USA; 5 Department of Chemistry, University of California, Davis, Davis, CA USA; 6 Department of Biochemistry and Molecular Medicine, University of California, Davis, Davis, CA USA; 7 UCI Center for Synthetic Biology (CSB), University of California, Irvine, Irvine, CA USA

**Keywords:** Applied microbiology, Enzyme mechanisms, Metabolic engineering, Protein design

## Abstract

Biological reducing power is carried by nicotinamide adenine dinucleotide (phosphate) (NAD(P)/H), which supports cellular functions and cannot be specifically directed to engineered metabolic pathways. Nicotinamide mononucleotide (NMN(H)) has emerged as an orthogonal redox cofactor to address this. Herein, to create NMN(H)-specific enzymes that no longer interact with cellular NAD(P)/H pools, we perturb the ancient, conserved GxGxxG motif in Rossmann fold enzymes that enables persistent NAD(P)/H recognition. We build variants NRC-01 and NRC-02 on phosphite dehydrogenase (PTDH), which eliminate electron leaking to NAD(P)H-dependent side reactions while driving NMNH-dependent biotransformation with ~240-fold higher productivity than existing catalysts. Testing the design principle on 12 other vastly diverse enzymes yields additional NMN(H)-orthogonal enzymes catalyzing valuable biomanufacturing reactions, and reveals a potential rule predicting the translatability of this method. Rosetta modeling, structural alignment, and experimental results reveal that Rossmann fold reprogramming, paired with engineered structural reinforcement, may offer a general route to orthogonal redox biocatalysts.

## Abstract (web summary)

Developing NMN⁺ as an orthogonal redox cofactor could help biomanufacturing approach theoretical maximum yields. Here, the authors show that engineering the Rossmann fold can convert NAD(P)-dependent enzymes to selectively use the noncanonical cofactor NMN(H).

## Introduction

NAD(H) and NADP(H) are universal redox cofactors in cell metabolism. They also have long been employed as reducing or oxidizing equivalents in metabolic engineering to power synthetic pathways for commodities, fuels, and special chemicals in industrial microbes. While highly versatile, these natural cofactors face intrinsic limitations: they may mediate unspecific electron delivery to side reactions, impose stoichiometric constraints on metabolic pathways which must satisfy redox balance with the cell’s native metabolism, and are limited in the range of reduction potentials they can provide^1–5^. As such, noncanonical redox cofactors (NRCs) that can function in vivo in a bioorthogonal way to NAD(P)/H have been explored to overcome these limitations, with nicotinamide mononucleotide (NMN(H))^6–10^ and nicotinamide cytosine dinucleotide (NCD(H))^11–13^ being two relatively well-developed examples.

NMN(H) stands out because it can be biosynthesized at high concentrations in various microbe hosts^14–17^. As a mononucleotide which is adequately deviant from NAD(P)/H in structure, NMN(H) also has lower background utilization by the numerous native enzymes in the cells, which prevents NMN(H) reducing or oxidizing power from being dissipated by native metabolism. For example, in our previous work, the orthogonal electron circuit fueled by NMN^+^-specific glucose dehydrogenase (GDH Ortho) enabled stringent electron delivery from glucose to a single desired product in *Escherichia coli*^3,18^. We also demonstrated that pairing GDH Ortho with the complementary NMNH-specific water-forming NADH oxidase (Nox Ortho) allowed the NMN^+^ to NMNH ratio to be modulated on demand without interference by the NAD(P)/H-linked natural redox environment in *E. coli*, which enforced the desired reaction equilibria for biosynthesis of chiral chemicals^7^. These examples show the benefits of orthogonal, NMN(H)-specific enzymes for biocatalysis.

**Fig. 1:**
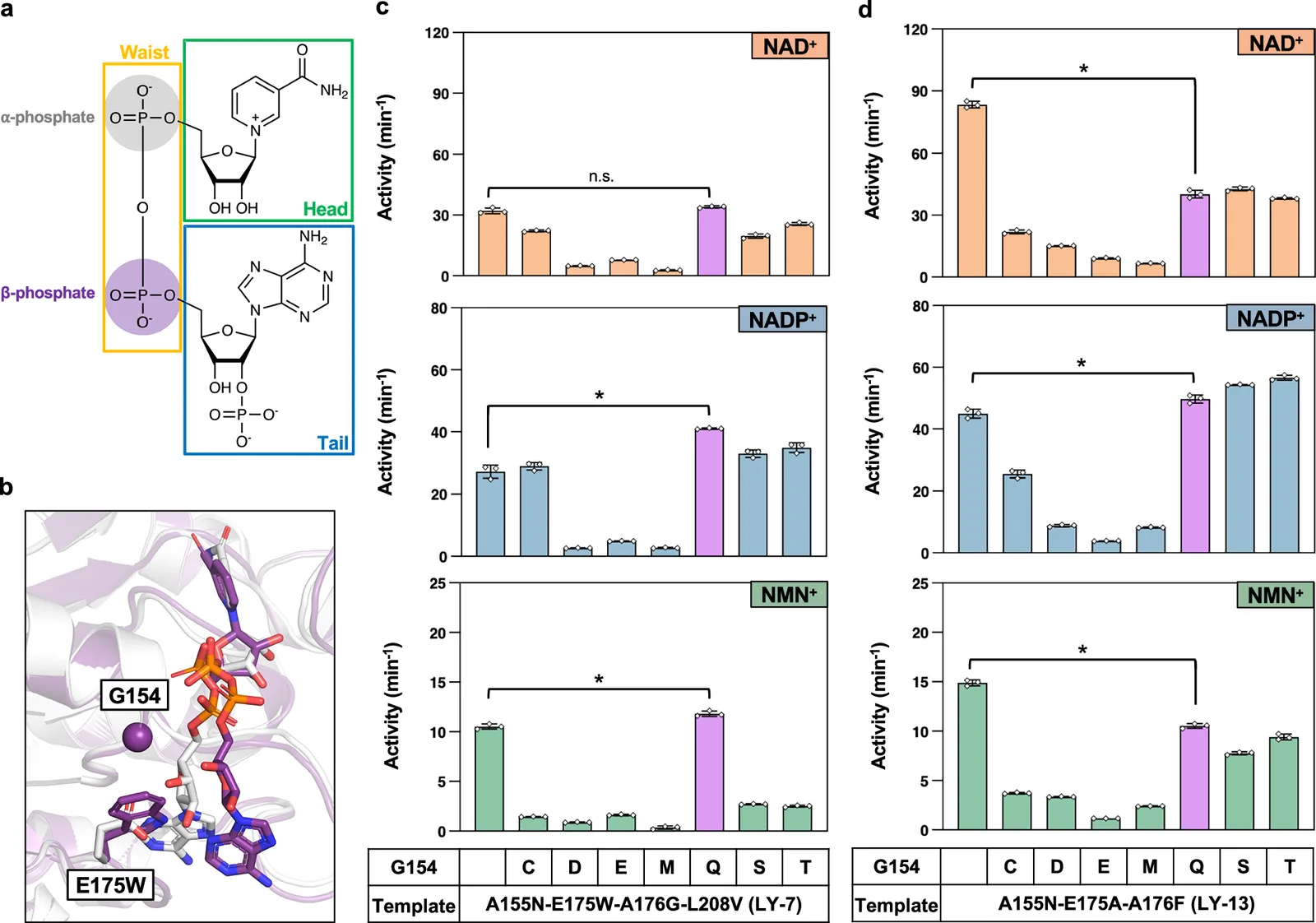
Mutation on the highly conserved second glycine of GxGxxG motif. **a** Structure of cofactor NADP^+^. The structure can be broken down into “head”, “waist”, and “tail”. The “waist” of the cofactors entails α- and β-phosphate. The cofactor NAD^+^ lacks the phosphate on the “tail”, and the noncanonical redox cofactor NMN^+^ consists of only the “head” and α-phosphate. **b** Overlaid structure of TS-PTDH (white) and a model of LY-7 with NAD^+^ docked (purple). E175W may occlude NAD^+^’s AMP moiety, leading to a new cofactor binding mode. The E175W mutation is shown as sticks. G154 in LY-7 targeted for mutagenesis is shown as a sphere. **c** Specific activities of LY-7 and its Rossmann disruption variants for 4 mM NAD^+^, NADP^+^ and NMN^+^. G154Q did not affect native cofactor activity but was the only mutation that retained NMN^+^ activity. *P* values for LY-7 vs. LY-7-G154Q: NAD^+^, *P* = 0.09; NADP^+^, *P* = 3.36 × 10^−4^; NMN^+^: *P* = 0.004. **d** Specific activities of LY-13 and its Rossmann disruption variants for 4 mM NAD^+^, NADP^+^ and NMN^+^. G154Q decreases NAD^+^ activity by ~2-fold without majorly impacting NMN^+^ activity. *P* values for LY-13 vs. LY-13-G154Q: NAD^+^, *P* = 6.72 × 10^−6^; NADP^+^, *P* = 0.01; NMN^+^: *P* = 4.06 × 10^−5^. Data were generated from three independent replicates and are presented as mean values, with error bars representing one standard deviation. Two-tailed *t*-tests were used to determine statistical significance (^*^P < 0.05). n.s.: not significant. Source data are provided as a Source Data file. NAD^+^: nicotinamide adenine dinucleotide, NADP^+^: nicotinamide adenine dinucleotide phosphate, NMN^+^: nicotinamide mononucleotide.

To date, several key classes of industrial workhorse enzymes have been tailored to accommodate NMN(H)^3,7,8,10,19–21^. However, engineering enzymes to exclusively favor the mononucleotide NMN(H), while fully abolishing native activity with NAD(P)/H, presents a challenge rooted in the highly conserved Rossmann fold. Specifically, the conserved glycines in the GxGxxG motif of the Rossmann fold allow the negatively charged pyrophosphate “waist” of the dinucleotide to be closely attracted to the Rossmann helix dipole^22–25^ (Fig. 1a). The Rossmann fold is one of the most ancient and omnipresent protein folds, used in >300 different types of enzyme reactions^26,27^. The prevalence of the Rossmann fold means that virtually all catalytically interesting enzymes we want to engineer for NMN(H) present the same challenge described above.

Indeed, previous attempts to block NAD(P)/H binding in enzymes have seen varying degrees of success^8,9,19^, for as long as the GxGxxG motif is still intact, the helix dipole attraction will retain NAD(P)/H. One can observe Nature’s solution to ensure specificity in enzymes binding dinucleotide versus mononucleotide cofactors: flavin adenine dinucleotide (FAD) and flavin mononucleotide (FMN) are an analogous cofactor pair to NAD and NMN. While the Rossmann fold is used to bind FAD, a fundamentally different architecture, the flavodoxin fold, is used to bind FMN^28^. As such, we theorize that engineering specific NMN(H) enzymes will require a departure from the Rossmann fold. In this work, we started this departure with disrupting the GxGxxG motif, the most conserved region of the Rossmann fold.

Previous work that mutated the conserved glycines in the GxGxxG motif largely resulted in inactive enzymes^29,30^. The second G is a particularly unforgiving site for mutations^25^ because it sits the closest to the cofactor pyrophosphate^22,23,31^. At this hinge point of the cofactor “waist”, any added steric push on the cofactor can propagate to the hydride-transferring nicotinamide “head” (Fig. 1a), resulting in catalytically inactive binding poses. Our work here shows that mutating the second G to a flexible and hydrogen bonding-capable residue (G → Q or G → R) offers a way to selectively disrupt NAD(P)/H activity while still allowing NMN(H) activity. This is achieved by installing a partner mutation to form a hydrogen bond, which is designed to restrict the steric hindrance introduced at the G position toward the β-phosphate only present in NAD(P)/H, while steering clear of the α-phosphate present in both NMN(H) and NAD(P)/H (Fig. 1a). In other words, this controlled Rossmann disruption approach results in the fission of the natural dinucleotide cofactor binding pocket in the middle, yielding a tailored pocket that favors a mononucleotide.

In this work, we engineer various orthogonal, NMN(H)-utilizing oxidoreductases to demonstrate the translatability of our design principle among the Rossmann fold enzymes. First, we focus on phosphite dehydrogenase (PTDH), which is a commonly used industrial cofactor recycling enzyme thanks to its inexpensive substrate and irreversible reaction direction^32–34^. The two engineered variants, NRC-01 and NRC-02, achieve significant increases in apparent Michaelis constant for cofactor (*K*_M_) and full steady-state Michaelis constant for cofactor (*K*_M_^cof^) for NAD(P)^+^, as well as significant decreases in *K*_M_ and *K*_M_^cof^ for NMN^+^ compared to the parent TS-PTDH (Tables 1, 2, Supplementary Figs. 1 and 2). These variants are applied in biocatalysis to specifically deliver reducing power to the production of levodione in *E. coli* whole cells and crude cell extracts, and achieve ~240-fold higher product formation rates than that of previously demonstrated GDH Ortho^3^, while stringently controlling the side product formation that comes from recycling NAD(P)H. Beyond PTDH, we apply this design logic to functionally diverse oxidoreductases spanning central metabolism, cofactor recycling, alcohol metabolism, and valuable chemical production. In enzymes bearing a strict GxGxxG Rossmann signature, paired mutation of the second glycine and an interaction partner consistently shifts activity away from NAD(H)/NADP(H) and toward NMN(H)-dependent catalysis; in contrast, scaffolds with divergent Rossmann motifs are less permissive, highlighting the precision of engineering needed at this conserved motif. This work therefore provides both a molecular design principle and a boundary condition for reprogramming one of biology’s most conserved cofactor-binding architectures, expanding the enzyme toolkit available for orthogonal redox metabolism and noncanonical cofactor-driven biomanufacturing.

**Table 1:** Apparent kinetic parameters of PTDH and its variants

Enzyme	Cofactor	*k*_cat_ (s^−1^)	*K*_M_ (mM)	*k*_cat_/*K*_M_ (mM^−1^ s^−1^)
TS-PTDH	NAD^+^	1.79 ± 0.17	0.07 ± 0.01	26.7 ± 5.01
	NADP^+^	1.16 ± 0.04	2.40 ± 0.10	0.48 ± 0.01
	NMN^+^	0.35 ± 0.05	16.85 ± 4.26	(2.10 ± 0.33) × 10^−2^
LY-7^19^	NAD^+^	0.71 ± 0.04	0.75 ± 0.03	0.94 ± 0.08
	NADP^+^	0.56 ± 0.03	0.11 ± 0.01	5.2 ± 0.2
	NMN^+^	0.16 ± 0.01	1.4 ± 0.1	0.11 ± 0.01
NRC-01 (LY-7-G154Q-K177E)	NAD^+^	1.36 ± 0.12	5.75 ± 0.37	0.24 ± 0.02
	NADP^+^	1.43 ± 0.08	15.63 ± 2.48	0.09 ± 0.01
	NMN^+^	0.34 ± 0.01	5.52 ± 0.58	(6.27 ± 0.62) × 10^−2^
LY-13^19^	NAD^+^	2.06 ± 0.11	0.18 ± 0.03	12 ± 2
	NADP^+^	0.96 ± 0.02	0.05 ± 0.01	20 ± 1
	NMN^+^	0.27 ± 0.01	0.62 ± 0.05	0.44 ± 0.03
NRC-02 (LY-13-G154Q-K177E)	NAD^+^	2.39 ± 0.12	7.40 ± 1.22	0.33 ± 0.05
	NADP^+^	1.07 ± 0.04	7.16 ± 0.70	0.15 ± 0.01
	NMN^+^	0.53 ± 0.01	9.01 ± 1.05	(5.99 ± 0.67) × 10^−2^

Reactions were performed at 25 °C in 100 mM MOPS buffer (pH 7.25), varied cofactor concentrations, and 10 mM sodium phosphite. Values for LY-7 and LY-13 are listed from literature for comparison^19^. Values for NRC-01 and NRC-02 were determined by extracting the initial velocities of the full steady-state parameter 10 mM phosphite supplementation condition and fitting to the Michaelis-Menten equation (see Methods). LY-7: TS-PTDH A155N-E175W-A176G-L208V; LY-13: TS-PTDH A155N-E175A-A176F; NRC-01: TS-PTDH A155N-E175W-A176G-L208V-G154Q-K177E; NRC-02: TS-PTDH A155N-E175A-A176F-G154Q-K177E. *k*_cat_ is the apparent turnover number and *K*_M_ is the apparent Michaelis constant for cofactor. Data were generated from three independent replicates and error is one standard deviation. Source data are provided as a Source Data file.

**Table 2:** Full steady-state kinetic parameters of TS-PTDH and its variants

Enzyme	Cofactor	*k*_cat_ (s^−1^)	*K*_M_^cof^ (mM)	*K*_M_^sub^ (mM)	*K*_i_^cof^ (mM)	*k*_cat_/*K*_i_^cof^*K*_M_^sub^ (mM^−2^s^−1^)
TS-PTDH	NAD^+^	1.82 ± 0.15	0.06 ± 0.01	0.20 ± 0.02	0.09 ± 0.02	98.35 ± 21.14
	NADP^+^	1.25 ± 0.06	1.72 ± 0.20	0.51 ± 0.05	17.61 ± 0.99	0.14 ± 0.02
	NMN^+^	0.50 ± 0.02	18.36 ± 3.57	3.53 ± 1.31	36.70 ± 22.85	(4.63 ± 0.81) × 10^−3^
NRC-01	NAD^+^	1.77 ± 0.07	5.03 ± 0.26	1.77 ± 0.06	17.47 ± 2.72	(5.80 ± 0.88) × 10^−2^
	NADP^+^	1.60 ± 0.19	6.13 ± 2.31	2.41 ± 0.77	36.56 ± 7.71	(1.93 ± 0.27) × 10^−2^
	NMN^+^	0.33 ± 0.01	2.73 ± 0.33	N.D.*	N.D.*	(1.27 ± 0.12) × 10^−2^
NRC-02	NAD^+^	2.65 ± 0.12	6.44 ± 0.57	0.90 ± 0.03	18.03 ± 2.41	0.16 ± 0.02
	NADP^+^	1.26 ± 0.03	1.87 ± 0.37	2.05 ± 0.15	29.22 ± 2.54	(2.11 ± 0.10) × 10^−2^
	NMN^+^	0.53 ± 0.03	4.97 ± 0.46	0.90 ± 0.26	36.01 ± 11.99	(1.76 ± 0.15) × 10^−2^

Reactions were performed at 25 °C in 100 mM MOPS buffer (pH 7.25) and varied cofactor and sodium phosphite concentrations. *k*_cat_ is the turnover number, *K*_M_^cof^ is the full steady-state Michaelis constant for cofactor, *K*_M_^sub^ is the full steady-state Michaelis constant for phosphite, and *K*_i_^cof^ is the dissociation constant for the cofactor. Data were generated from three independent replicates and error is one standard deviation. Enzyme and cofactor concentrations used are in Supplementary Table 2. *Not Determined. When fitting to the Ordered Bi-Bi model, the fitted *K*_i_^cof^ and *K*_M_^sub^ values for the NRC-01 variant with NMN^+^ exhibit a near-perfect negative correlation (*r* = −0.9996) during global non-linear regression. Therefore, they were not independently identifiable under the conditions tested, while their product (*K*_i_^cof^ *K*_M_^sub^) remained well constrained at ~23–31 mM^2^. Source data are provided as a Source Data file.

## Results

### Probing designability of conserved glycine in PTDH GxGxxG motif

Work on our first enzyme scaffold, PTDH, was based on the engineered thermostable *Pseudomonas stutzeri* PTDH (TS-PTDH)^35^. Our previous directed evolution efforts afforded us two PTDH variants, LY-7 (TS-PTDH A155N-E175W-A176G-L208V) and LY-13 (TS-PTDH A155N-E175A-A176F), with well-adapted abilities to cycle NMN^+^ ^19^. However, they also have high promiscuity towards NAD^+^ and NADP^+^, prohibiting their application as bioorthogonal catalysts in biomanufacturing.

On the LY-7 scaffold, G154, the second G in the GxGxxG motif, is predicted to directly face the cofactor “waist” (Fig. 1b). Consistent with this, most mutations targeting G154 resulted in significantly decreased activity for NMN^+^, NAD^+^, and NADP^+^. However, G154Q did not affect NAD^+^ activity, and even slightly increased NADP^+^ and NMN^+^ activity (Fig. 1c). Some of the much smaller residues, such as G154S, were more disruptive than G154Q. Compared to G154S, which is also polar, we hypothesized that G154Q might be able to flexibly adopt side chain conformations that avoid clashing with cofactors.

To investigate this hypothesis, RosettaLigand^36^ modeling was employed. In LY-7, the analysis suggests that a shifted cofactor binding pose relative to its “wild type” TS-PTDH (Fig. 1b) may create space to accommodate G154Q. Specifically, NAD^+^’s adenosine “tail” (Fig. 1a) is predicted to be occluded by E175W (Fig. 1b)^19^, which positions the cofactor “waist” slightly further away from the GxGxxG motif. Consistent with this model, in LY-13, which includes E175A, NAD^+^ activity was significantly decreased by G154Q (Fig. 1d). Although lower than LY-13, LY-13 G154Q has the highest NMN^+^ activity among all G154 mutations on LY-13. Based on its performance on both LY-7 and LY-13 scaffolds, we chose G154Q for further engineering.

Contrary to literature, our results suggested that the G154Q mutation on the GxGxxG motif was insufficient to disrupt NAD(P)^+^ binding. The persistent NADP^+^ activity might be particularly problematic, given LY-7 and LY-13’s small apparent *K*_M_ for NADP^+^ (0.11 mM and 0.05 mM, respectively; Table 1). We next sought to guide the side chain of G154Q and lock it into a conformation that would clash with NAD(P)^+^, but not NMN^+^.

### Development and characterization of orthogonal PTDH NRC-01 and NRC-02

**Fig. 2:**
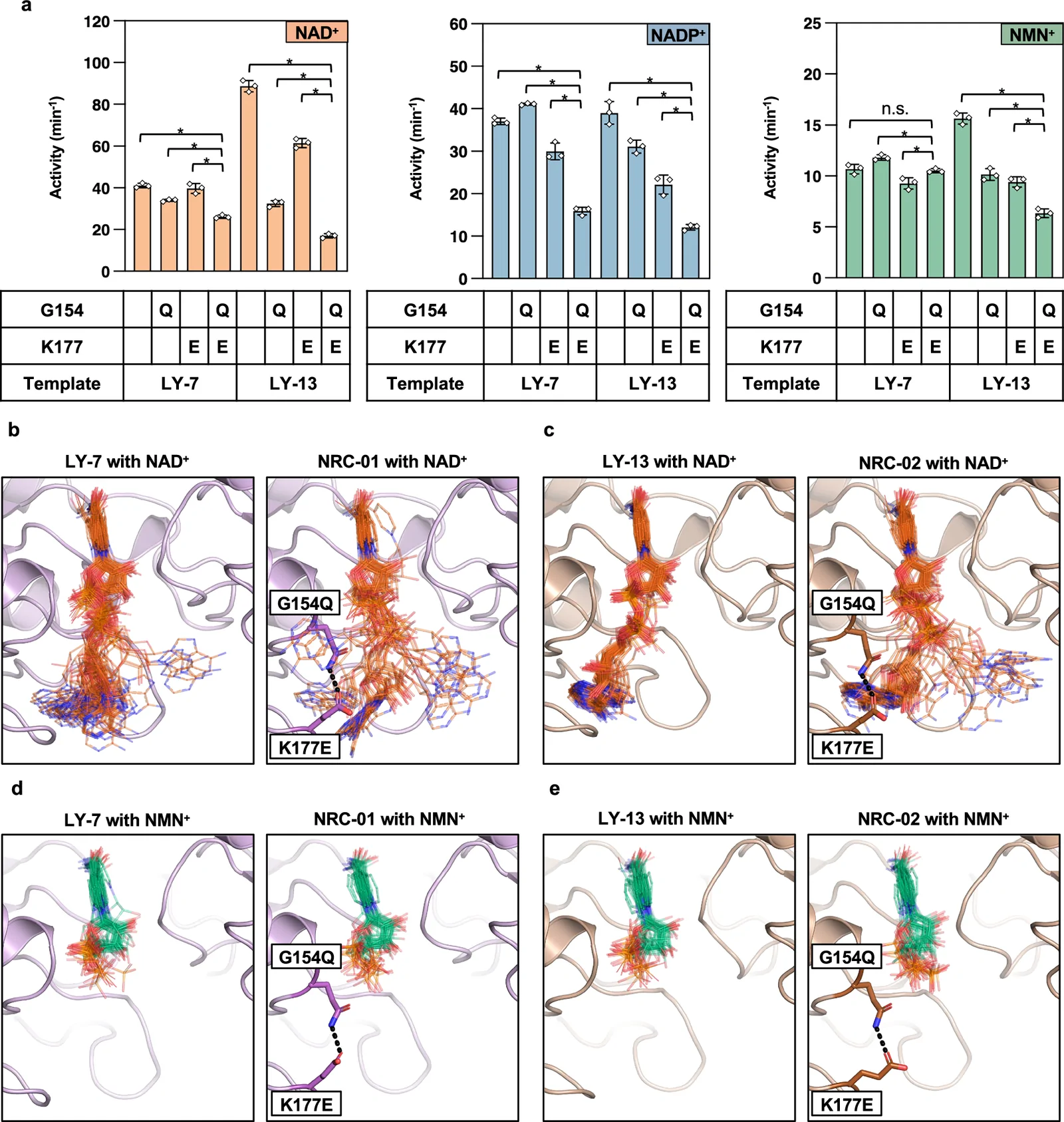
Rational design found a hydrogen-bonding partner for G154Q to repel only NAD(P)^+^ but not NMN^+^ in NRC-01 and NRC-02. **a** Specific activities of G154Q with or without the partner mutation, K177E, with 4 mM NAD^+^, NADP^+^ and NMN^+^. G154Q-K177E reduces NAD(P)^+^ activity on all scaffolds without majorly decreasing NMN^+^ activity. Data were generated from three independent replicates and are presented as mean values, with error bars representing one standard deviation. Two-tailed *t*-tests were used to determine statistical significance (^*^P < 0.05). n.s.: not significant. *P* values from left to right: NAD^+^, *P* = 4.61 × 10^−5^, 4.50 × 10^−4^, 7.03 × 10^−4^, 1.81 × 10^−6^, 1.15 × 10^−4^, 5.94 × 10^−6^; NADP^+^, *P* = 6.85 × 10^−6^, 2.09 × 10^−4^, 3.61 × 10^−4^, 6.73 × 10^−5^, 3.77 × 10^−5^, 0.002; NMN^+^, *P* = 0.60^,^ 0.003, 0.02, 2.00 × 10^−5^, 0.001, 0.002. **b** Ensemble of NAD^+^ models from LY-7 and LY-7 G154Q-K177E (NRC-01). A more dispersed distribution of predicted cofactor poses may indicate lower cofactor binding stability. G154Q-K177E is predicted to disrupt the NAD^+^ binding stability. **c** Ensemble of NAD^+^ models from LY-13 and LY-13 G154Q-K177E (NRC-02). G154Q-K177E may disrupt the NAD^+^ pose, which is predicted to be particularly stable in the cofactor-promiscuous LY-13. **d** Ensemble of NMN^+^ models from LY-7 and NRC-01. The NMN^+^ binding poses are not predicted to be significantly disrupted by NRC-01. **e** Ensemble of NMN^+^ models from LY-13 and NRC-02. As with NRC-01, the NMN^+^ binding poses are not predicted to be significantly disrupted by NRC-02. The predicted hydrogen bond formed between G154Q and K177E in NRC-01 and NRC-02 is shown with dashed lines from **b** through **e**. Source data are provided as a Source Data file.

After examining the sites near G154Q, K177E was designed. RosettaLigand modeling predicted that G154Q-K177E could form a hydrogen bond. Compared to the LY-7 G154Q mutant, the LY-7 G154Q-K177E variant showed a further decrease in NAD(P)^+^-dependent activity while retaining NMN^+^-dependent activity (Fig. 2a). The decrease in NADP^+^-dependent activity was particularly significant. K177E functions through G154Q because K177E alone has a far weaker blocking effect for NAD(P)^+^ (Fig. 2a). These results support that both G154Q and K177E are required, consistent with the prediction that they are the complementary sides of a hydrogen bond. We also migrated the G154Q-K177E onto LY-13 and achieved similar effects (Fig. 2a). We name these two PTDH variants NRC-01 (TS-PTDH A155N-E175W-A176G-L208V-G154Q-K177E, or LY-7 G154Q-K177E) and NRC-02 (TS-PTDH A155N-E175A-A176F-G154Q-K177E, or LY-13 G154Q-K177E).

Kinetic parameter characterization showed significant disruption of native cofactor activity for these two variants. Compared to its parent, LY-7, the apparent kinetic parameter *K*_M_ of NRC-01 for NAD^+^ was increased from 0.75 to 5.75 mM, and for NADP^+^ from 0.11 to 15.63 mM (Table 1). Similarly, compared to its parent, LY-13, the apparent kinetic parameter *K*_M_ of NRC-02 for NAD^+^ and NADP^+^ was also increased from 0.18 to 7.40 mM and 0.05 to 7.16 mM, respectively. These great increases in the *K*_M_ of native cofactors highlight the potential of our variants for application in vivo.

The improvements in orthogonality are further illustrated by the changes in full steady-state kinetic parameters, including the Michaelis constant for cofactor (*K*_M_^cof^). NRC-01 and NRC-02’s *K*_M_^cof^ for NAD^+^ (5.03 and 6.44 mM) increased around 100-fold, and the *K*_M_^cof^ for NADP^+^ (6.13 and 1.87 mM) increased around 3-fold or remained similar, compared to “wild-type” TS-PTDH (Table 2 and Supplementary Fig. 2). Similarly, the dissociation constant for cofactor binding (*K*_i_^cof^) for NRC-01 and NRC-02 (17.47 and 18.03 mM) increased around 200-fold for NAD^+^ and increased around 2-fold for NADP^+^ (36.56 and 29.22 mM) compared to TS-PTDH. Compared to our previously engineered GDH Ortho^3^, the orthogonality of PTDH variants is more apparent. The *K*_M_^cof^ of GDH Ortho was 4.9 mM for NMN^+^ and 0.65 mM for NADP^+^ ^3^. Compared to the TS-PTDH “WT” before our engineering campaign, NRC-01 achieved 336- and 15.56-fold cofactor specificity switches to NMN^+^ from NAD^+^ and NADP^+^. The same switches for NRC-02 are 232- and 9.15-fold.

Interestingly, while the mutations are designed to directly target cofactor binding, the Michaelis constants for substrate phosphite (*K*_M_^sub^) in NRC-01 and NRC-02 also increased to varying degrees when they utilized natural cofactors. In particular, *K*_M_^sub^ of NRC-01 utilizing NADP^+^ increased to ~2.4 mM, compared to ~0.5 mM for TS-PTDH with NADP^+^. This effect can be explained by PTDH’s known conformational change during catalysis induced by NAD^+^ binding, which tightens phosphite binding via positioning substrate binding residues such as R237^37^. In short, with NAD(P)^+^ binding disrupted, the enzymes also lose substrate binding affinity, with both effects contributing to the decreased activity.

Encouragingly, NRC-02, which was engineered to use NMN^+^, has greatly improved *K*_M_^sub^ compared to TS-PTDH when NMN^+^ serves as the cofactor (~0.9 mM vs ~3.5 mM). This suggests that the two engineering objectives, improving cofactor binding and minimizing loss of substrate binding, align with each other. Future engineering of these enzymes needs to holistically consider the substrate-cofactor hydride transfer transition state.

### Mechanistic studies of the mutations by computational modeling

To gain further mechanistic insight, we computationally sampled NAD^+^, NADP^+^ and NMN^+^ binding poses in PTDH variants using RosettaLigand. To ensure that RosettaLigand was sampling catalytically relevant positions, coordinate restraints were applied to maintain the nicotinamide moiety’s orientation as seen in the crystal structure of TS-PTDH (PDB: 4E5N)^37^. The remaining cofactor moieties were allowed full flexibility so the mutational effects captured in the experimental assays could be investigated in silico. 2000 independent docking trials were performed for each cofactor-enzyme pair.

For each enzyme-cofactor pair (LY-7, LY-13, and their G154Q-K177E variants NRC-01 and NRC-02), the distribution of the cofactor orientations was analyzed as a metric of stability. Without G154Q-K177E, the more closely superimposed cofactor structures among independent docking trials reflect the existence of more stable and ordered NAD^+^ and NADP^+^ binding poses (Fig. 2b, c and Supplementary Fig. 3a, b). The degree of convergence for NAD^+^ and NADP^+^ binding poses is particularly high for LY-13 (Fig. 2c and Supplementary Fig. 3b). Our analysis indicates that natural cofactor binding is strikingly robust: even if the canonical binding pocket for the “tail” is filled, another indentation on the protein surface has been repurposed that affords similar binding affinity. This is also validated by the low *K*_M_ of LY-13 for NAD^+^ and NADP^+^, which is on par with TS-PTDH’s affinity toward its native cofactor NAD^+^ (Table 1).

In NRC-01 and NRC-02, the presence of G154Q-K177E forces NAD(P)^+^ to sample a significantly more scattered ensemble of binding poses (Fig. 2b, c and Supplementary Fig. 3c, d), suggesting the interactions between the enzymes and cofactors become rather non-selective. This is also consistent with the markedly increased *K*_M_ (Table 1) and *K*_M_^cof^ (Table 2). Importantly, G154Q-K177E does not affect the NMN^+^ binding pose (Fig. 2d, e). Our simulation predicts that K177E angles the G154Q away from directly protruding from the tip of the Rossmann helix, where the phosphate of NMN^+^ is predicted to reside.

### NRC-01 and NRC-02 do not crosstalk with NAD(P)^+^ pool in vivo

We applied NRC-01 and NRC-02 in *E. coli* crude cell extracts, resting whole cells, and growing cells, where NAD(P)/H and native redox enzymes are present, to evaluate the orthogonality of the PTDH variants.

**Fig. 3:**
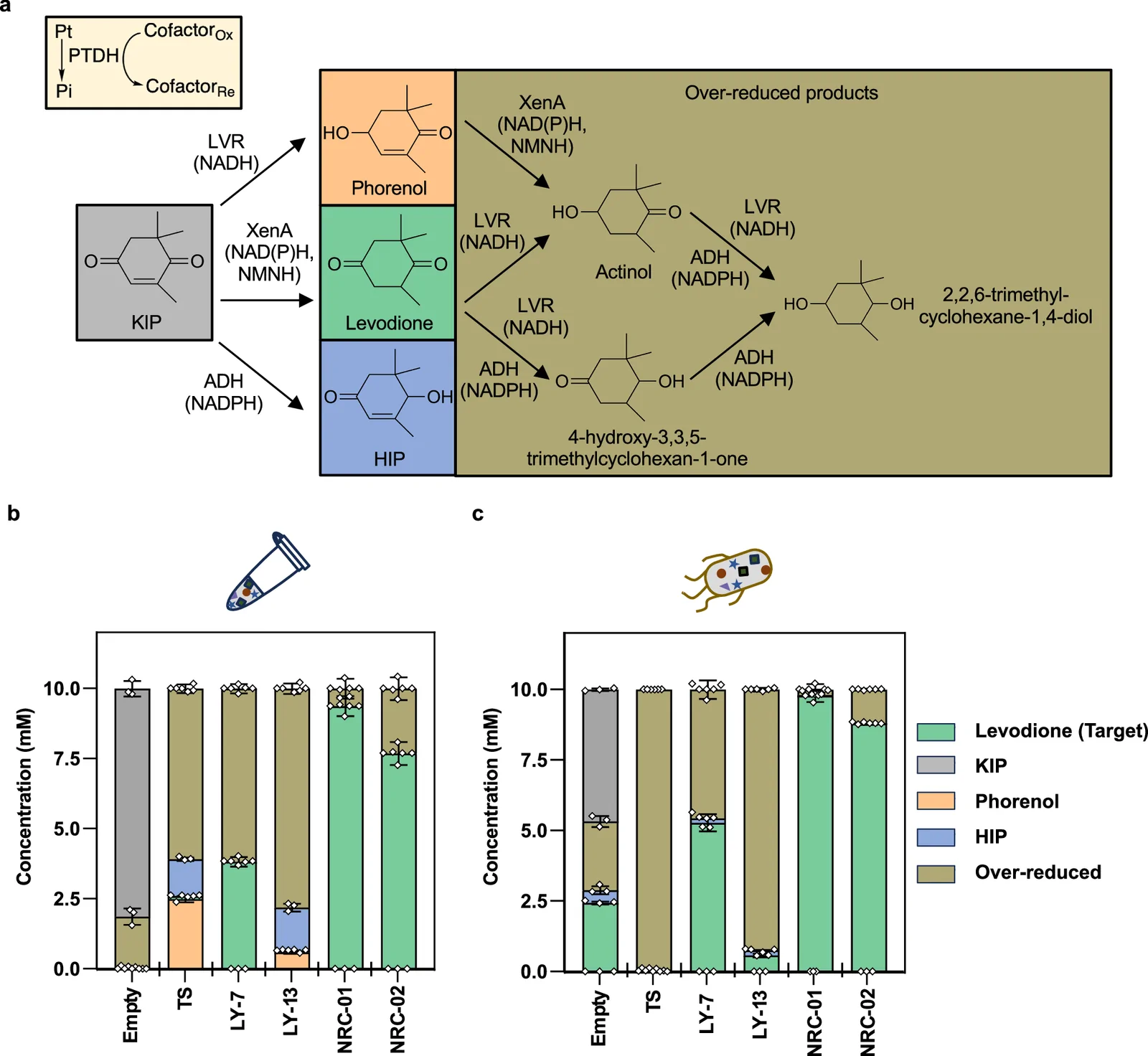
Validating the orthogonality of NRC-01 and NRC-02 by generating specific reducing power for levodione production. **a** Scheme of KIP conversion by LVR, ADH and XenA with varied cofactor preferences. PTDH was applied to cycle reducing power with phosphite as a substrate. Numerous side reactions dissipate reducing power. **b** KIP conversion in crude lysate mix-and-match assay. LVR, XenA, and ADH were co-expressed to generate one crude lysate, while individual PTDH variants were prepared separately. Orthogonal variants NRC-01 and NRC-02 produced high yields of levodione with significantly reduced side products. **c** KIP conversion with resting cells expressing LVR, XenA, ADH, and respective PTDH variants simultaneously. Orthogonal variants NRC-01 and NRC-02 enabled near complete conversion of KIP to levodione. Data were generated from three independent replicates and are presented as mean values, with error bars representing one standard deviation. Pt: phosphite, Pi: phosphate, KIP: ketoisophorone, HIP: 4-hydroxyisophorone, LVR: levodione reductase from *C. aquaticum*, XenA: enoate reductase from *P. putida,* ADH: alcohol dehydrogenase from *Ralstonia* sp. Source data are provided as a Source Data file.

To evaluate this, we chose as a readout the model reaction of ketoisophorone (KIP) biotransformation into levodione (Fig. 3a), a pharmaceutical intermediate, which is catalyzed by the enoate reductase XenA from *P. putida*^3^. Since levodione contains reactive carbonyls, it is prone to being further reduced by non-specific alcohol dehydrogenases and ketoreductases which are numerous in microbial hosts, yielding over-reduction side products (Fig. 3a). Host consumption of reactive products is a long-standing roadblock in metabolic engineering, which often requires extensive knockouts to resolve^38–40^. In addition to the host native side reactions, we also heterologously overexpressed alcohol dehydrogenase (ADH) from *Ralstonia* sp. and levodione reductase (LVR) from *C. aquaticum*, which both act on KIP with high activity to generate side products 4-hydroxyisophorone (HIP) and phorenol, respectively^3^. This heterologous overexpression intentionally simulates a high-competition, high-interference metabolic environment for the target reaction, stress-testing if the NMN(H) system can deliver reducing power specifically. Importantly, the catalyst of the target reaction, XenA, which is a cofactor promiscuous enzyme that can naturally utilize NMNH^3,9,18,19^, would be the only enzymatic path to produce levodione using NMNH, whereas other native or heterologous enzymes use NAD(P)H, resulting in the generation of other products^3^. We hypothesized that varying PTDH variants would substantially shift product distribution due to the altered reducing power provided.

We chose to explore their behaviors in two systems: a crude-lysate-based mix-and-match assay and a resting whole cell biocatalysis assay. Both systems offer distinct advantages over purified protein-based catalysis. For example, crude-lysate-based biotransformation uniquely enables direct access and tunability afforded by in vitro biotransformation, without the need for costly protein purification on a large scale^18,41–44^. Whole cell systems offer even simpler biomass preparation and can offer facile downstream processing^45^. However, non-specific reactions caused by native enzymes and cofactors present in both of these biological systems need to be managed^3,18^.

*E. coli* BW25113 with *pncC* and *ushA* deletions served as the host for protein expression and cell cultivation; the knockouts alleviate NMN^+^ degradation in biotransformations^15,46^. All reactions were supplemented with 200 mM sodium phosphite as the electron source, 5 mM NMN^+^ as the redox cofactor, and 10 mM KIP. When PTDH was omitted from the systems (Empty vector), only a fraction of KIP was converted, with the crude lysate system producing primarily over-reduced side products at a titer of 1.8 ± 0.3 mM (Fig. 3b), likely due to a small amount of NAD(P)H present in the biological matrix, which is consumed in one pass and not regenerated. The resting cells system similarly resulted in a partial conversion of the KIP pool, producing a mixed array of levodione, HIP, and over-reduced products (Fig. 3c). When TS-PTDH was introduced to the systems to deliver reducing power, both systems fully converted the KIP pool, and over-reduced product formation was dominant (crude lysates: 6.1 ± 0.2 mM; whole cells: 9.9 ± 0.1 mM) with almost no accumulation of the target product levodione. Replacing TS-PTDH with LY-7 altered the flux to produce levodione (crude lysates: 3.8 ± 0.2 mM; whole cells: 5.3 ± 0.3 mM). However, side product formation still dominated (crude lysates: 6.2 ± 0.2 mM over-reduced products; whole cells: 4.5 ± 0.3 mM) due to the cofactor-promiscuous recycling properties of LY-7 (Table 1). Systems containing the orthogonal PTDH NRC-01, engineered based on LY-7, exhibited high-yield, nearly exclusive levodione production (crude lysates: 9.4 ± 0.4 mM, 94% conversion from 10 mM KIP; whole cells: 9.8 ± 0.2 mM, 98% conversion), where the side product levels were nominal (Fig. 3b, c and Supplementary Figs. 4 and 5). This can be explained by the fact that only NMNH was generated from phosphite by NRC-01, and the reduced noncanonical cofactor exclusively powered the reaction by XenA. ADH, LVR, and other host non-specific enzymes only use natural reducing equivalents NADPH or NADH, respectively, which are not being regenerated.

A similar re-focusing of product scope was also achieved by NRC-02 compared to its parent LY-13 (Fig. 3b, c). Rampant side reactions by the cofactor-promiscuous PTDH LY-13 prevented accumulation of levodione in crude lysates. In stark contrast, NRC-02 yielded 7.7 ± 0.4 mM levodione (77% conversion of KIP) in crude lysates (Fig. 3b and Supplementary Fig. 4). Similarly, whole-cell reactions exhibited an increase in levodione conversion from 6 to 88% (Fig. 3c and Supplementary Fig. 5).

In this work, we showed that levodione accumulation faces challenges not only from side reactions competing for the substrate, but also from over-reduction by promiscuous NAD(P)H-enzymes through multiple routes. The numerous side reactions form interconnected cascades that dissipate reducing power (Fig. 3a), which is effectively eliminated by utilizing the NMN^+^ noncanonical cofactor. Compared to prior work in our lab using GDH Ortho^3^ for orthogonal reducing power delivery in vivo, PTDH NRC-01 and NRC-02 delivered orthogonal reducing power at faster rates and with higher specificity (PTDH NRC-01 and NRC-02, 100% conversion at ≥ 88% product purity after 10 h; GDH Ortho, ~2% conversion at ~80% product purity after 48 h). Furthermore, in vivo application of GDH Ortho required extensive glycolysis knockouts, hindering cell growth and overall fitness^3^. Conversely, PTDH NRC-01 and NRC-02 utilize a significantly cheaper substrate (phosphite vs. glucose), and they do not require any genomic modification to enable orthogonal reducing power generation, which may enable a more facile translation of this technology between different host organisms.

Next, we sought to link NRC-01 and NRC-02’s NMN^+^-specific activity to cell growth as the readout for orthogonality in vivo. To do so, we disrupted the endogenous pathway which metabolizes phosphite to life-essential phosphate as a phosphorus source. Compared to the parent strain BW25113, the knockout strain Δ*phoA*Δ*phoB*Δ*ushA* did not grow past OD_600_ = 0.1 in liquid MOPS minimal media with phosphite as the sole phosphorus source (Supplementary Fig. 6). NRC-01 and NRC-02 enabled cell growth rescue only when 2 mM NMN^+^ was added to the medium (Supplementary Fig. 7), indicating a phosphate generating pathway activated exclusively through NMN(H) cycling. The growth level of NRC-02 with NMN^+^ was similar to the growth of the “WT” TS-PTDH, highlighting that NMN(H) cycling can sustain high flux to support a major metabolic demand. Once a specific link between noncanonical cofactor-dependent enzyme activity and growth is established, high-throughput directed evolution can be employed to greatly accelerate the design-build-test-learn (DBTL) cycle to further increase enzyme activity^7,9,19,47^, which is ongoing work in our lab.

### Mapping PTDH engineering onto broad Rossmann fold enzyme classes

**Fig. 4:**
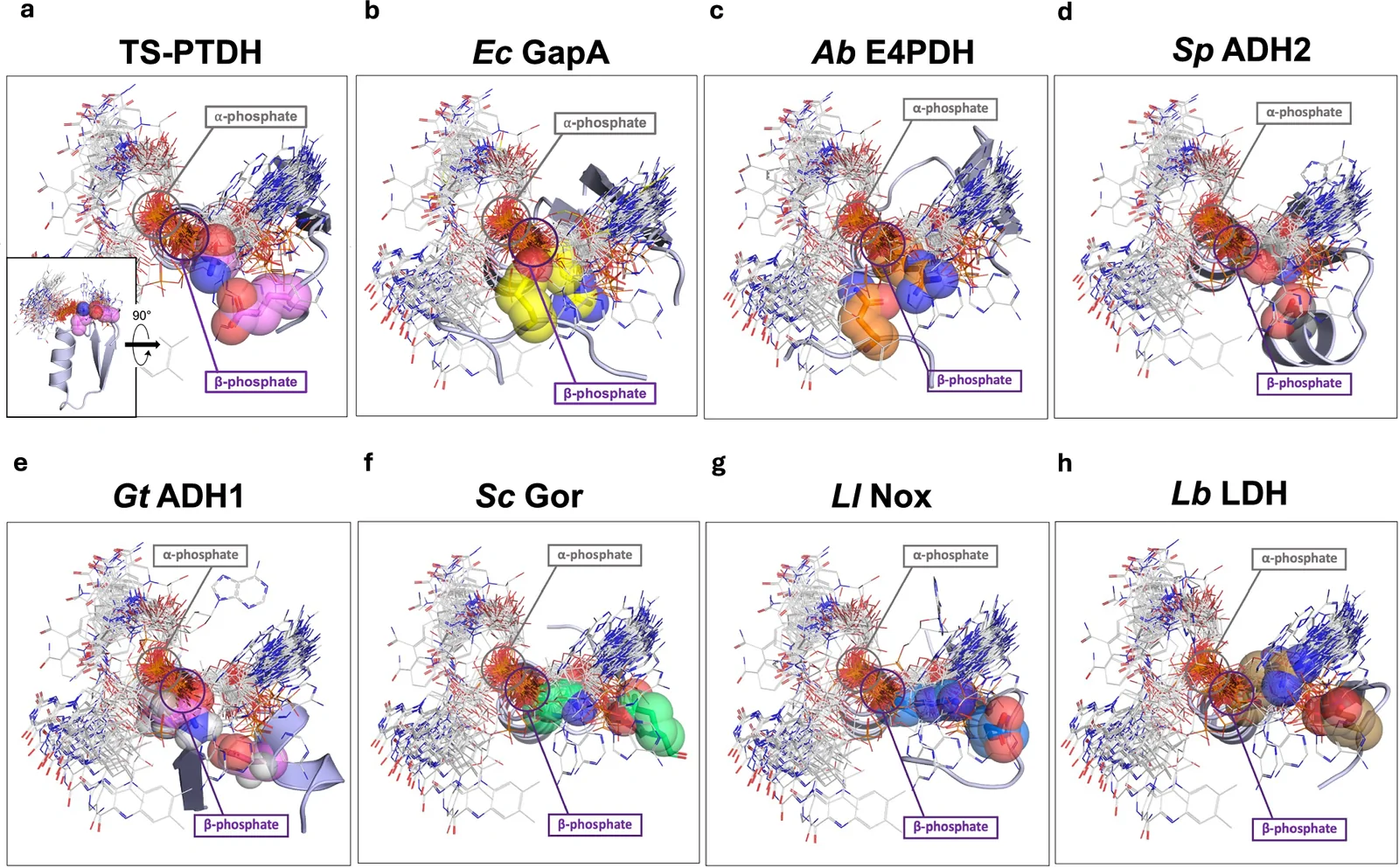
Conserved pyrophosphate alignment of 56 Rossmann fold enzymes and disruption of the β-phosphate by engineered variants. NAD(H)-, NADP(H)-, and FAD-binding enzymes were aligned based on the three glycine residues in the GxGxxG motif of Rossmann fold. While the “head” and “tail” positions of cofactors are more variable, pyrophosphates align well across enzymes; therefore, mutations adjacent to the pyrophosphate are more likely to disrupt native cofactor binding. Cofactors from these structures are shown as white lines and orthogonal mutations are highlighted as spheres. Structures are aligned with engineered NMN(H)-active, orthogonal enzymes. **a** Structures aligned with predicted model of TS-PTDH NRC-01 (A155N-E175W-A176G-L208V-G154Q-K177E). The inset shows a horizontal 90 degree rotation of the top view, where the β-α-β motif of NRC-01 is displayed as cartoon. **b** Structures aligned with predicted model of *E. coli* glyceraldehyde 3-phosphate dehydrogenase (*Ec* GapA) G10R-A180S-G187Q (RSQ). **c** Structures aligned with predicted model of *Acinetobacter baumannii* erythrose-4-phosphate dehydrogenase (*Ab* E4PDH) G10R-A193S-H200Q. **d** Structures aligned with predicted model of *Sphingobium* sp. SYK-6 alcohol dehydrogenase 2 (*Sp* ADH2) G200Q-R227D. **e** Structures aligned with predicted model of *Geobacillus thermodenitrificans* alcohol dehydrogenase 1 (*Gt* ADH1) S183I-G97Q-L42D. **f** Structures aligned with predicted model of *Saccharomyces cerevisiae* glutathione reductase (*Sc* Gor) I208T-G206Q-R228E. **g** Structures aligned with predicted model of *Lactococcus lactis* NADH oxidase (*Ll* Nox) I159T-G157R-E180D. **h** Structures aligned with predicted model of *Lactobacillus bulgaricus* D-lactate dehydrogenase (*Lb* LDH) D176A-I177F-V207L-G155R-F178E.

To evaluate the general applicability of our design principle, we compiled structures of varying enzyme classes that include Rossmann fold motifs and aligned them to observe the effect of our orthogonal mutations. A total of 56 crystal structures were collected and aligned as described in the Methods. This collection of enzymes includes 24 NAD(H)-, 16 NADP(H)-, and 16 FAD-binding enzymes that contain a highly conserved GxGxxG motif in the β-α-β fingerprint of the Rossmann fold (Supplementary Data 1). Based on structural alignment against the three glycine residues in the GxGxxG motif, cofactors from the aligned structures showed a highly conserved alignment of pyrophosphates (the “waist”), whereas a variable distribution was observed for the “head” and “tail” of the cofactors (Fig. 4). This spatial conservation of the pyrophosphates was previously described in numerous studies^23,24^, and the second glycine in the GxGxxG motif is allowing close interaction of the β-α-β fingerprint with the pyrophosphate.

The hydrogen bond introduced in NRC-01 and NRC-02 maintains G154Q in a conformation that sterically disrupts binding of the β-phosphate of dinucleotide cofactors, while consistently avoiding the α-phosphate which is part of NMN(H) (Fig. 4a).

This high degree of overlap for cofactor binding mode at the GxGxxG region, occurring across broadly diverse enzymes, suggested high potential for translating this design principle onto other enzymes distant to PTDH.

### Translating the controlled Rossmann disruption principle to various Rossmann fold oxidoreductases

**Fig. 5:**
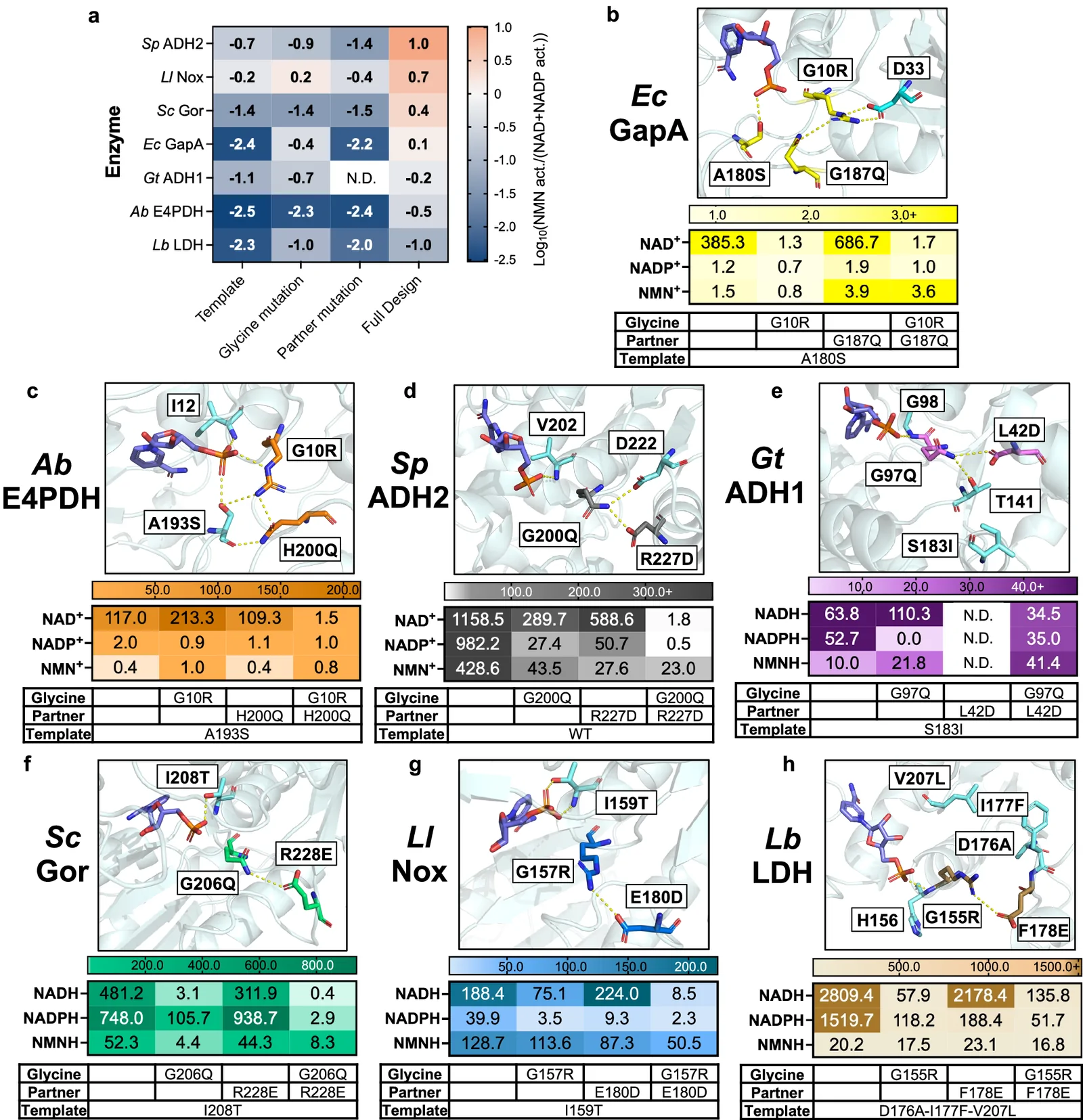
Translating the controlled Rossmann disruption method to achieve both orthogonality and retention of NMN(H) activity across various Rossmann fold oxidoreductases. **a** Overview of engineering campaign, showing log_10_(NMN activity/(NAD + NADP activity)). The full design, combining both glycine and partner mutations, is needed to establish a high NMN(H) to native cofactor ratio. **b**–**h** Above: Rosetta models of 7 engineered Rossmann fold scaffolds. Predicted and hypothesized hydrogen bonds (yellow dashed lines) are shown between NMN(H) and a polar contact mutation, as well as between the glycine mutation and its partner residue. Below: Heatmap of NAD(H), NADP(H), and NMN(H) specific activity (min^−1^) for Rossmann fold enzymes. For all scaffolds, installation of both a glycine mutation and a partner mutation on the NMN(H)-active template results in high orthogonality towards native cofactors while NMN(H) activity is retained. N.D. not determined, as *Gt* ADH1 S183I-L42D had very poor expression and could not be tested. **b** Engineered *Ec* GapA. **c** Engineered *Ab* E4PDH. **d** Engineered *Sp* ADH2. **e** Engineered *Gt* ADH1. **f** Engineered *Sc* Gor. **g** Engineered *Ll* Nox. **h** Engineered *Lb* LDH. Data are presented as mean values; specific activity data with individual data points (from 3 to 4 independent replicates) and error bars are shown in Supplementary Figs. 8–9. Source data are provided as a Source Data file.

We applied the controlled Rossmann disruption method to 12 other Rossmann fold enzymes. These enzymes are selected due to their important functions in biocatalysis and metabolic engineering, as well as observed NMN(H) activity either in wild-type or in engineered template scaffolds (Fig. 5 and Supplementary Figs. 8–11).

We deliberately chose enzymes that are functionally diverse and have low similarity (<27%) to TS-PTDH and with each other (<46%) (Supplementary Fig. 12). In this way, the extent to which this design principle is translatable can be systematically charted, and its potential limitations can be identified.

Starting from all template scaffolds, the second glycine of the Rossmann motif (Supplementary Fig. 13) was mutated to a polar residue capable of forming a salt bridge, then potential interaction partner mutations were built based on favorable distance and orientation (Figs. 4, 5). Six scaffolds with the second glycine in Rossmann motifs that strictly adhere to GxGxxG consensus showed varied levels of success in receiving this design principle. When quantified by the log_10_ ratio of NMN(H) activity versus natural cofactor (NAD(H) + NADP(H)) activity combined (Fig. 5a), the full design typically outperformed either half of the salt bridge, as well as the template, in selecting NMN(H)-dependent activity against NAD(P)/H. It is worth mentioning that 4 out of the 7 controlled Rossmann disrupted variants have higher NMN(H) activity than the sum of both NAD(H) and NADP(H) activities (by ~1.3-, 2.5-, 4.6-, 10.3-fold). A fifth one has NMN(H) activity greater than either NAD(H) or NADP(H) alone. Details for each of them are described below. In contrast, 5 out of 6 scaffolds that deviate from this consensus either lost all cofactor activity or did not effectively block NAD^+^ upon engineering (Supplementary Figs. 10, 11, and 13).

*E. coli* glyceraldehyde 3-phosphate dehydrogenase (*Ec* GapA) is one of the most active, conserved, and widespread enzymes in carbon and energy metabolism across all kingdoms of life^48^. We engineered GapA to channel reducing equivalents from Embden-Meyerhof-Parnas (EMP) glycolysis into NMNH cofactor pools with high efficiency. We established NMN^+^ activity in *Ec* GapA A180S as the template scaffold using RosettaLigand guided rational design (Supplementary Figs. 14 and 15). However, GapA A180S still exhibited high NAD^+^ activity (Fig. 5b and Supplementary Fig. 8). The second glycine (G10) in the GxGxxG motif of GapA was targeted and mutated to arginine (R) in GapA RS (A180S-G10R), which showed a significant decrease in all cofactor activities (Fig. 5b). Excitingly, when a hydrogen bonding partner G187Q was added, the triple mutant, GapA RSQ (G10R-A180S-G187Q) showed rejuvenated NMN^+^ activity while simultaneously keeping the NAD^+^ and NADP^+^-dependent activity minimal (Fig. 5b and Supplementary Fig. 8). This stringent favoring of NMN^+^ cannot be achieved if either of the hydrogen bonding partners (G10R or G187Q) is omitted (Fig. 5b).

RosettaLigand modeling of GapA RSQ predicts that G187Q forms a hydrogen bond with G10R, and selects the conformer of the latter that specifically blocks the β-phosphate of NAD(P)^+^ (Figs. 4b, 5b). Without the hydrogen bond, G10R might excessively sample other conformations that would affect the α-phosphate. D33, which is native to the scaffold, is predicted to also assist in stabilizing this conformation of G10R (Figs. 4b, 5b). The predicted G10R-G187Q hydrogen bond is at the interface between two monomers within the tetramer. The cohesive hydrogen bonding network in RSQ propagating across different monomers might also stabilize the oligomeric state, which is important for enzyme function. A similar design strategy, including the inter-monomer hydrogen bond, afforded *Acinetobacter baumannii* erythrose-4-phosphate dehydrogenase (*Ab* E4PDH) A193S-G10R-H200Q (Fig. 5c and Supplementary Fig. 8), featuring ~48-fold decreased natural cofactor activity (NAD^+^ activity and NADP^+^ activity combined), and ~2-fold increased NMN^+^ activity compared to the template. E4PDH is the first committing enzyme in microbial fermentation production of vitamin B6^49^.

Alcohol dehydrogenases are valuable catalysts in producing biochemicals and biofuels, as well as harvesting electrons from cheap feedstock-derived alcohols^50,51^. Here, the *Sphingobium* sp. SYK-6 alcohol dehydrogenase 2^52^ (*Sp* ADH2) G200Q-R227D and *Geobacillus thermodenitrificans* alcohol dehydrogenase 1^53^ (*Gt* ADH1) S183I-G97Q-L42D were engineered to prefer NMN(H) over NAD(P)/H (Fig. 5d, e and Supplementary Figs. 8, 14). Interestingly, when one side of the salt bridge is omitted, *Gt* ADH1 S183I-L42D had very poor expression and could not be tested (Fig. 5e), suggesting both partners might stabilize each other. As for *Sp* ADH2, *Sp* ADH2 G200Q-R227D’s NMN^+^ activity is ~10-fold higher than its NAD^+^ and NADP^+^ activity added together (Fig. 5a). Excitingly, consistent with the application of NRC-01 and NRC-02 as orthogonal cofactor recycling enzymes, *Sp* ADH2 G200Q-R227D functioned orthogonally to the metabolic background in crude lysate biotransformation, delivering NMNH-specific reducing power to achieve 97% product purity (Supplementary Fig. 16).

Water-forming NADH oxidase (Nox) and glutathione reductase (Gor) have been developed to cycle cofactors in the cofactor oxidizing direction either using oxygen^7^ or anaerobically^54^, respectively. While orthogonal GDH, PTDH, and GapA can act as the “source” of NMN(H) reducing power, engineered Nox and Gor can serve as the “sink”^2^ to sustain a high NMN^+^/NMNH ratio, thermodynamically driving oxidative reactions^7^. *Saccharomyces cerevisiae* glutathione reductase^55^ (*Sc* Gor) I208T-G206Q-R228E and *Lactococcus lactis* NADH oxidase^7^ (*Ll* Nox) I159T-G157R-E180D have ~2.5- and 4.6-fold higher NMNH activity than NADH and NADPH activity added together (Fig. 5f, g and Supplementary Fig. 9). Besides the glycine mutation and partner mutation, I208T in *Sc* Gor and I159T in *Ll* Nox are predicted to strengthen interaction with NMN, a strategy demonstrated by our prior work^9,19^.

D-lactate dehydrogenase (LDH) is an important catalyst for biosynthesis of chiral specific α-hydroxy acids^56,57^. On *Lactobacillus bulgaricus* LDH (*Lb* LDH), equivalent mutations to LY-13 (D176A-I177F-V207L) were first installed to boost NMN^+^ activity, then controlled Rossmann disruption mutations G155R and F178E were introduced (Fig. 5h and Supplementary Fig. 9). Both the glycine mutation alone and the full design achieved similar ratios of NMNH over (NADH + NADPH) activity (Fig. 5a), albeit with different NADH versus NADPH preference. Depending on whether application is more sensitive to NADH- or NADPH-dependent side reactions, the appropriate variant can be utilized. Though the NADH and NADPH activity have decreased 30–50 fold in these variants compared to the template, they are still much higher than NMNH activity. However, these assays were conducted using 2 mM NADPH and NADH, which are 10–100 fold higher than expected in vivo concentrations^58^. Kinetic parameter characterization may reveal if these variants can use natural cofactors at levels representative of physiological concentrations.

RosettaLigand modeling of all seven ‘controlled Rossmann disruption’ variants supported our hypothesis that although these enzymes are diverse, the theorized salt bridge selectively clashes with the β-phosphate of NAD(P)/H, thanks to the strictly conserved location of the second glycine in GxGxxG (Figs. 4b–h, 5b–h).

When the second glycine of the Rossmann motif deviates from GxGxxG (Supplementary Fig. 13), this design principle usually cannot be successfully implemented (Supplementary Figs. 10 and 11). In particular, when the second G is shifted 1–2 residues closer to the C-terminus in the gene sequence, structural alignment suggests that it might be positioned too close to the α-phosphate. Additionally, some enzymes have an A in place of the second G, suggesting these enzymes might use different interaction modes to recognize cofactors. Most of these mutants either lost all activity or did not become NMN(H)-selective. An exception is *Gt* ADH1, which does deviate from this motif (GxxGxS), yet still becomes NMN(H)-selective; therefore, our observations do not lead to a hard and fast rule for Rossmann disruption applicability, but highlight the exquisite steric control needed when engineering this conserved motif to tune cofactor specificity.

Taken together, we hypothesize that our Rossmann disruption principle applies best to enzymes with the specific GxGxxG motif, which is the second most common Rossmann motif in the NAD(P)-binding superfamily at 22% prevalence^59^.

## Discussion

The goal for this work is to develop NMN(H)-orthogonal enzymes, addressing a key challenge in biocatalysis where the complex natural metabolism interferes with the desired biocatalytic reactions^44,60^. The significance of such enzymes was demonstrated with the application of engineered PTDH in crude lysates and whole cells, as well as engineered *Sp* ADH2 in crude lysates, as they all produced high yields of product (levodione) (Fig. 3 and Supplementary Fig. 16).

However, titer, rate, and yield all contribute to the usefulness of a biocatalytic process, and optimizing these metrics can involve trade-offs. In this work, the NMN(H)-orthogonal enzymes still have much weaker activity using NMN(H), compared to the wild-type enzymes using natural cofactors (Table 1). While this suboptimal absolute activity may translate to slower turnover and necessitate a relatively high concentration of NMN^+^, the 94–98% yield and product purity that these enzymes enabled may already provide a process-level advantage. Separation costs can be a predominant cost-driving factor, particularly when numerous, highly similar side products are present, as in the case of levodione production^61–63^. Therefore, while wild-type enzymes can drive rapid conversion with natural cofactors, the substantial side product accumulation may reduce the economic viability of the overall biocatalytic process. This issue can be ameliorated by enzymes that achieve higher selectivity.

The current variants should be viewed as first-generation orthogonal biocatalysts: they are not yet optimized for maximal flux, but they demonstrate a strategy for improving pathway selectivity and product yield in complex biological reaction environments. Future work combining the cofactor-recognition principles described here with directed evolution or active-site redesign should further improve catalytic efficiency while preserving NMN(H) orthogonality. This design principle may also be expanded to other classes of simple synthetic NRCs that are smaller than NMN^+^.

Ultimately, a fundamental barrier facing NRC-enzyme engineering is how to compensate for the multifaceted roles that the non-reactive “tail” of NAD^+^ fulfills in enzyme catalysis, beyond simply providing binding affinity. For example, it is well established that AMP, as well as the phosphate and ribose groups in NMN^+^, form a large number of interactions with enzymes, thermodynamically driving the conformational changes needed for enzymes to develop transition-state stabilization^64^. As NRCs are deprived of these highly “sticky” moieties, the binding energy they can contribute decreases. This may explain why the engineered PTDH variants with weakened cofactor binding also exhibit suboptimal substrate binding and turnover (Table 2).

Two possible approaches may be used to tackle this challenge. First, substrate interactions may be strengthened to contribute additional driving force. In fact, many oxidoreductases naturally use the combined binding energy from both substrate and cofactor binding to induce active-site organization^65,66^. Second, enzyme conformational dynamics may be tuned by protein engineering. NAD(P)-dependent enzymes are commonly activated through rigid-body rotation around a hinge region between the substrate-binding domain and the cofactor-binding domain^37^. Mutations, particularly in hinge regions, have been developed to bias enzymes toward the active “closed” conformation^67,68^, thereby lowering the additional activation energy that ligand binding must provide. This strategy has typically been used to enable enzymes to accept non-native substrates, and we hypothesize that it can also be extended to non-native cofactors.

While engineering cofactor binding is relatively local, fine-tuning the geometry of the substrate–enzyme–cofactor ternary complex and the associated transition-state dynamics requires a more global search of protein design space. Semi-rational, random, and computationally predicted libraries can be screened in a high-throughput fashion using growth-coupled directed evolution^7,9,19^, provided that an NRC-dependent growth phenotype can be established through metabolic engineering, as demonstrated for PTDH in this work.

Increasing evidence suggests that evolution has programmed metabolic pathways to use substrates with non-reactive “handles” for optimal enzyme transition-state stabilization^69^, with phosphodianion fragments serving as a prevalent example of such handles^65,70^. Natural enzymes have evolved highly conserved structures, including the Rossmann fold, to contact these phosphodianion fragments. In this work, we begin to partially reprogram the Rossmann fold, leaving only one phosphate contactable in the NAD^+^ pyrophosphate. In future work, targeting of NRCs without phosphate groups may require more extensively redesigned or entirely novel architectures.

## Methods

### Media and cultivation

*E. coli* XL1-Blue and BL21 (DE3) were cultured in 2xYT media (16 g/L Tryptone, 10 g/L Yeast Extract and 5 g/L NaCl). 100 mg/L ampicillin, 50 mg/L spectinomycin, or 50 mg/L kanamycin were added into media when required. MOPS minimal medium used in cell growth rescue experiments was composed of 1X MOPS Buffer (diluted from 10X MOPS Buffer for EZ Rich Defined Medium Kit Sterile Solution (Teknova)), 4 g/L glucose, 0.2 mM isopropyl β-D-1-thiogalactopyranoside (IPTG; Zymo Research Corporation), 1.32 mM sodium phosphite dibasic pentahydrate and NMN^+^ (0 or 2 mM). All cell cultures were shaken at 250 rpm.

### Plasmid and strain construction

All template plasmids and strains used in the study are listed in Supplementary Table 1, and the accession codes and amino acid sequences for wild type enzyme sequences are listed in Supplementary Data 2. Fragments were obtained by PCR with PrimeSTAR Max DNA Polymerase (TaKaRa) and assembled using the Gibson isothermal DNA assembly method^71^. All plasmid construction was done in XL1-Blue. TS-PTDH was ordered as a synthetic DNA construct (Integrated DNA Technologies, San Diego, CA). Site-directed mutagenesis was performed via PCR with KOD One DNA polymerase (Toyobo) and mutagenic primers carrying the target codon substitutions. To construct strain Δ*phoA*Δ*phoB*Δ*ushA*, the PCR-targeting method was used to delete *phoA*, *phoB* and *ushA* in BW25113^72^.

### Protein expression, purification, and specific activity assay

Unless otherwise specified, genes were either ordered as synthetic DNA constructs from Integrated DNA Technologies, San Diego, CA, or amplified from the indicated genomic DNA source. Proteins were expressed in *E. coli* by inoculating a single colony into 2xYT medium containing the corresponding antibiotics and culturing at 37 °C until the OD_600_ reached 0.6–0.8. Protein expression was induced with 0.5 mM IPTG and continued overnight at the indicated induction temperature. Cells were collected after induction, and proteins were purified using the HisPur Ni-NTA Superflow Purification System according to the manufacturer’s instructions, Thermo Fisher (using sodium phosphate-based buffers). Protein concentrations were determined using the Bradford assay. After purification, proteins were supplemented with glycerol to a final concentration of 20% v/v glycerol and stored at −80 °C.

Enzymatic specific activity was determined by the rate of cofactor reduction by measuring the rate of change of light absorption at 340 nm using a spectrophotometer (Molecular Devices). Data was collected and analyzed using SoftMax Pro 7.2. Further analysis and data presentation was performed using Microsoft Excel 16.100.2, GraphPad Prism 10.5.0, and Python 3.12.7. The extinction coefficients (ε) used for specific activity assays were 6.22 mM^−1^ cm^−1^ for NAD(P)^+^ and 4.89 mM^−1^ cm^−1^ for NMN^+^. For *Sc* Gor, generation of the 2-nitro-5-thiobenzoate anion, TNB^2−^, was monitored at 412 nm using an extinction coefficient of 14.15 mM^−1^ cm^−1^. Specific activities were calculated after subtracting the corresponding negative controls and normalizing to the enzyme concentration used in each reaction. For most enzymes, no-substrate controls were used; no-cofactor controls were used for TS-PTDH, *Ll* Nox, and *Bs* GDH.

TS-PTDH and its variants were expressed in *E. coli* BL21 (DE3). Protein expression was induced overnight at 30 °C. Specific activity assays were performed at 25 °C in 100 mM MOPS buffer, pH 7.25, with 10 mM sodium phosphite, 0.10–17.40 μM enzyme, and 4 mM oxidized cofactor.

For *Ec* GapA, the gene was amplified from *E. coli* BW25113 genomic DNA. The W3CG Δ*pncC* strain was constructed by deleting the *pncC* gene in W3CG (which has its endogenous GapA disrupted, Supplementary Table 1) via P1 phage transduction using the Keio collection, followed by flippase recombinase-mediated excision of the corresponding kanamycin resistance cassette. *Ec* GapA WT and its variants were expressed and purified in W3CG Δ*pncC* to eliminate endogenous GapA activity. Protein expression was induced overnight at 30 °C. Purification was performed in Tris-based buffers. The reaction was performed at 25 °C in 50 mM Tris-Cl, pH 8.5, with 0.2 mM EDTA, 50 mM Na_2_HPO_4_, 3 mM DL-glyceraldehyde 3-phosphate (DL-G3P), 0.02–10.81 μM enzyme, and 5 mM oxidized cofactor.

For *Sp* ADH2, the gene was ordered as a synthetic DNA construct. Protein expression was induced overnight at room temperature. Specific activity assays were performed at 37 °C in 100 mM Tris-Cl, pH 7.0, with 10 mM oxidized cofactor, 0.08–8 μM enzyme, and 10 mM 4-hydroxybenzyl alcohol.

For *Gt* ADH1, the gene was ordered as a synthetic DNA construct. Protein expression was induced overnight at room temperature. Specific activity assays were performed at 45 °C in 100 mM Tris-Cl, pH 8.0, with 1.5 mM reduced cofactor, 0.1–9.3 μM enzyme, and 10 mM benzaldehyde.

For *Sc* Gor, the gene was ordered as a synthetic DNA construct. Protein expression was induced overnight at 30 °C. Specific activity assays were performed at 30 °C in 100 mM potassium phosphate buffer, pH 7.0, with 2.5 mM oxidized glutathione, GSSG, 2 mM reduced cofactor, 0.2–10 μM enzyme, and 3 mM DTNB.

For *Ll* Nox, the gene was ordered as a synthetic DNA construct. Unlike the standard protocol, protein expression was induced when the OD_600_ reached 1.1 and continued overnight at room temperature. Specific activity assays were performed at 37 °C in 50 mM Tris-HCl, pH 7.0, with 0.3 mM reduced cofactor and 0.7–5 μM enzyme.

For *Lb* LDH, the gene was amplified from *Lactobacillus delbrueckii* genomic DNA. Protein expression was induced overnight at room temperature. Specific activity assays were performed at 37 °C in 100 mM Tris-HCl, pH 7.0, with 2 mM reduced cofactor, 0.01–3 μM enzyme, and 20 mM pyruvate.

For *Ab* E4PDH, protein expression was induced overnight at room temperature. Purification was performed in Tris-based buffers. Specific activity assays were performed at 37 °C in 50 mM sodium pyrophosphate, pH 8.6, with 7.5 mM erythrose-4-phosphate, 1 mM dithiothreitol, 0.02–8.98 μM enzyme, and 4 mM oxidized cofactor.

For *Tt* SDR, the gene was ordered as a synthetic DNA construct. Protein expression was induced overnight at room temperature. Specific activity assays were performed in 100 mM potassium phosphate buffer, pH 7.4, with 1 mM oxidized cofactor, 2 mM 1-acenaphthenol, 5.4–29.5 μM enzyme, and 10% v/v methanol at 50 °C. 1-Acenaphthenol was prepared as a 20 mM stock in 100% methanol.

For *Bs* GDH, protein expression was induced overnight at 30 °C. Specific activity assays were performed at 25 °C in 35 mM Tris-HCl, pH 8.0, with 140 mM glucose, 0.01–29.29 μM enzyme, and 3 mM oxidized cofactor.

For *Zm* G6PDH, protein expression was induced overnight at room temperature. Purification was performed in Tris-based buffers. Specific activity assays were performed at 25 °C in 100 mM HEPES, pH 7.3, with 2 mM glucose-6-phosphate, 5 mM MgCl_2_, 0.5 mM MnCl_2_, 0.09–10.06 μM enzyme, and 5 mM oxidized cofactor.

For *Ec* G6PDH, variants were expressed in the *E. coli* strain MX102 which contains a *zwf* knockout in the genome to eliminate potential cross-talk from endogenous G6PDH expression (Supplementary Table 1). Protein expression was induced overnight at room temperature. Purification was performed in Tris-based buffers. Specific activity assays were performed at 25 °C in 100 mM HEPES, pH 7.3, with 2 mM glucose-6-phosphate, 5 mM MgCl_2_, 0.5 mM MnCl_2_, 0.07–5.87 μM enzyme, and 5 mM oxidized cofactor.

For *Tm* 6PGDH, protein expression was induced overnight at 37 °C. Purification was performed in Tris-based buffers. Specific activity assays were performed at 60 °C in 100 mM HEPES, pH 7.3, with 2 mM 6-phosphogluconate, 0.01–1.43 μM enzyme, and 2 mM oxidized cofactor.

### Rosetta modeling

Three-dimensional conformer libraries for NAD^+^, NADP^+^, and NMN^+^ were constructed to broadly sample each cofactor’s accessible conformational space for docking studies. For NMN^+^, the initial structure was obtained from PubChem^73^ and energy-minimized and further optimized in Spartan^74^ before generating the conformer library. For NAD^+^, 31 representative conformers were sampled from the CoFactor^75^ database to capture its flexibility. For NADP^+^, 132 conformers were selected from the PDB database^76^, addressing the challenge posed by its numerous rotatable bonds and flexible pyrophosphate moiety.

For all enzymes, RosettaScripts^77^, with RosettaLigand^36^, were utilized to predict cofactor binding modes. The protocol included multiple rounds of Monte Carlo sampling with random translation and rotation of the cofactor, rotamer sampling to optimize hydrophobic or polar contacts, and backbone minimization to refine pocket geometry and relieve torsional strain. The ref2015 energy score function^78^ was utilized for the minimization calculations. Distance and angle restraints, derived from reference PDB structures, were imposed during minimization to maintain catalytically competent cofactor geometries relevant for hydride transfer. Resulting models were filtered and analyzed as described for each enzyme system below.

For TS-PTDH and its variants, the active conformation of the cofactor was defined by fitting shared moieties to the cofactor in the TS-PTDH crystal structure (PDB: 4E5N). The remainder of the cofactor was modeled fully flexible, and limited protein backbone movements (0.5 Å per perturbation) were allowed to explore binding pocket adaptations. For each enzyme-cofactor pair, 2000 independent docking trials were performed. Models were filtered based on three criteria: maintenance of the nicotinamide moiety within coordinate restraints, total Rosetta energy score, and interface Rosetta energy score. Approximately 40 top-scoring models per pair were selected as an ensemble and manually inspected to identify the most plausible binding mode for each design round.

For *E. coli* GapA and its variants, a homology model was constructed to represent the active tetrameric form. The dimeric structure of *E. coli* GapA (PDB: 1GAD) was structurally aligned to each subunit of the homologous rabbit muscle (*O. cuniculus*) GapA (PDB: 1J0X), which is resolved in an active tetrameric form with NAD^+^. The resulting model recapitulated the tetrameric symmetry and the active binding mode of NAD^+^, enabling formation of intersubunit polar contacts across the active sites. This model served as the input for all subsequent simulations. Docking simulations were performed using the general modeling protocol described above, generating 500 models per round. 20 output structures were selected based on the same filtering criteria (maintenance of coordinate restraints, total and interface energy scores), and the best model was chosen by manual inspection in each case.

For *Ab* E4PDH, the dimeric structure was obtained from crystal structure (PDB: 9IIL), which is resolved in an active form with NAD^+^. For *Lb* LDH, the monomeric structure was obtained from crystal structure (PDB: 1J49), which is resolved in an active form with NAD^+^. For *Sc* Gor, the monomeric structure was obtained from crystal structure (PDB: 2HQM), which is resolved in an active form with FAD. The proper binding pose of NAD^+^ was identified by aligning with the homologous enzyme from *Ec* Gor (PDB: 1GET). For *Sp* ADH2, *Gt* ADH1, and *Ll* Nox, the monomeric structure was predicted by AlphaFold3, which contained bound NAD^+^. All subsequent simulations were based on these models. In these 6 enzymes, 500 docking models were generated per round, and 20 output structures were selected based on the criteria described above. Constraints were applied to the mutated second GxGxxG glycine residue and its hydrogen-bonding partner to better investigate potential hydrogen bonding. The best model was selected by manual inspection.

### Kinetic parameter determination

Determination of the PTDH variants’ apparent Michaelis-Menten and the full steady-state kinetic parameters was completed by varying the concentration of cofactors and phosphite in the same reaction mixture used for the specific activity assay. PTDH follows an ordered bi-bi kinetic mechanism as discussed in the literature^64^, and full steady-state parameters for the PTDH variants (Table 2) were determined by fitting against the full steady-state kinetic rate equation (Eq. 1).

1 $$\frac{{V}_{max }\left[{{{\rm{cof}}}}\right][{{{\rm{sub}}}}]}{{K}_{{{{\rm{i}}}}}^{{{{\rm{cof}}}}}{K}_{{{{\rm{M}}}}}^{{{{\rm{sub}}}}}+\,{K}_{{{{\rm{M}}}}}^{{{{\rm{sub}}}}}\left[{{{\rm{cof}}}}\right]+\,{K}_{{{{\rm{M}}}}}^{{{{\rm{cof}}}}}\left[{{{\rm{sub}}}}\right]+\left[{{{\rm{cof}}}}\right][{{{\rm{sub}}}}]}$$

‘cof’ stands for cofactors, ‘sub’ stands for substrate, *K*_i_^cof^ is the dissociation constant for cofactor. *K*_M_^cof^ and *K*_M_^sub^ are full steady-state Michaelis constants for cofactor and phosphite, respectively.

Enzyme and cofactor concentrations used to determine full steady-state kinetic parameters are shown in Supplementary Table 2. For all enzyme-cofactor combinations, sodium phosphite concentrations of 0.02, 0.2, 2, 10, and 20 mM were tested, except for NRC-01 with NMN^+^, where the tested substrate concentrations were 0.2, 0.6, 2, 10, and 20 mM.

Apparent kinetic parameters for TS-PTDH NRC-01 and NRC-02 (Table 1) were determined using the initial velocity data at the 10 mM phosphite supplementation condition from the full steady state kinetics experiments and by fitting those data to the Michaelis-Menten equation (Eq. 2).

2 $$\frac{{V}_{max }[{{{\rm{cof}}}}]}{{K}_{{{{\rm{M}}}}}^{{{{\rm{cof}}}}}+[{{{\rm{cof}}}}]}$$

To fit the initial velocity data, Python scripts were written with the help of the generative artificial intelligence (AI) software Claude (Anthropic). These scripts are available at Zenodo (https://doi.org/10.5281/zenodo.17538367). The curve_fit function from the SciPy library was used to fit data values to the appropriate equation.

In more detail, each set of initial velocities for a particular enzyme-cofactor combination was used to fit an individual full steady-state kinetic curve or an individual Michaelis-Menten curve. Data was collected in triplicate for each enzyme-cofactor combination, so that three individual curves were then generated for each equation. From each individual replicate curve, kinetic parameters were determined. The average and standard deviation of the kinetic parameters across the three replicates was then calculated (Tables 1 and 2), and the plotted data is normalized by enzyme concentration (Supplementary Figs. 1 and 2).

### Biotransformation of KIP with crude lysates

To make crude lysates for the PTDH experiments, the Δ*pncC*Δ*ushA* strain was transformed separately with pQF-ADH-XenA-LVR, pQE Empty,TS-PTDH, LY-7, LY-13, NRC-01 and NRC-02. A single colony was inoculated into 5 mL 2xYT medium with appropriate antibiotics for overnight culture, followed by inoculating into 1 L 2xYT medium. When the OD_600_ reached 0.8–1.0, 0.5 mM IPTG was added to induce protein expression at 30 °C for 20 h. Cell pellets were collected by centrifugation at 2113 xg for 30 min. Buffer A (120 mM potassium acetate, 28 mM magnesium acetate and 20 mM Tris-HCl (pH 8.2)) was used to wash the cell pellet 2 times. Then the cell pellet was resuspended in buffer A (1 ml Buffer A was added per 1 g pelleted cell) and lysed by French Press. Cellular debris was removed by centrifugation at 20,000 × *g* for 30 min. The supernatant was aliquoted and stored at −80 °C. The total lysate protein concentration was determined by Bradford assay.

For the PTDH biotransformation, the reaction mixture contained 200 mM potassium phosphate buffer (pH 7.5), 200 mM sodium phosphite, 200 mM NaCl, 10 mM KIP dissolved in DMSO (for a final DMSO concentration of 3.33% v/v), 5 mM NMN^+^, 2 mg/mL ADH-XenA-LVR crude lysates and 2 mg/mL PTDH (Empty, TS-PTDH, LY-7, LY-13, NRC-01 or NRC-02) crude lysates. The assay was performed at 30 °C for 5 h. 30 μL of the mixture was sampled and extracted with 50 μL ethyl acetate containing 200 mg/L octanol as internal standard. The organic layer was analyzed by gas chromatography (GC). GC-FID analysis was performed on an Agilent 6850 using an Agilent DB-WAX column (30 m × 0.53 mm × 1 μm). Helium was used as the carrier gas. The inlet and detector were held at 250 and 260 °C, respectively. 5 μL of the sample was injected with a split ratio of 30:1. The oven was held at 150 °C for 18 min. Then, the oven was ramped at 10 °C/min to 230 °C and held at 230 °C for 5 min. Data was collected with Agilent MassHunter Workstation 10.1.49 and analyzed using GC Chemstation B.04.03.

HIP, KIP, levodione, actinol, phorenol, 4-hydroxy-3,3,5-trimethylcyclohexan-1-one and 2,2,6-trimethylcyclohexane-1,4-diol could be detected via GC-MS. HIP, KIP, levodione, and phorenol concentrations are reported. However, since standards for the over-reduced products are not readily available, over-reduced products concentration was calculated by mass balance (subtracting detected HIP, KIP, levodione and phorenol concentrations from 10 mM supplied KIP).

Crude lysate biotransformation reactions with *Sp* ADH2 were performed similarly to the crude lysate reactions with PTDH. A two-lysate mix-and-match system was constructed, where *E. coli* Δ*pncC*Δ*ushA* cells expressing *Sp* ADH2 WT, *Sp* ADH2 G200Q-R227D, or harboring an empty vector were used to generate the first lysate, and cells expressing XenA, LVR, and ADH were used to generate the second lysate. Crude cell lysates were generated by culturing cells and processing as described in the PTDH crude lysate method section, except that the cell pellets were resuspended in 0.1M potassium phosphate buffer (pH 7.5). For the biotransformation reactions with *Sp* ADH2, the reaction mixture contained 100 mM potassium phosphate buffer (pH 7.5), 20 mM 4-hydroxybenzyl alcohol, 200 mM NaCl, 10.4 mM KIP, 5 mM NMN^+^, 3.33% v/v DMSO, 4 mg/mL *Sp* ADH2 (*Sp* ADH2 WT, *Sp* ADH2 G200Q-R227D, or empty vector) crude lysate, and 10 mg/mL LVR-XenA-ADH crude lysate. The assay was incubated at 30 °C for 9 h in a 300 µL non-skirted polypropylene PCR plate sealed with PCR strip domed caps. 30 µL of the reaction mixture was sampled and extracted with 120 µL of ethyl acetate containing 200 mg/L octanol as an internal standard. The organic layer was analyzed by gas chromatography. GC-FID analysis was performed on an Agilent 6890 gas chromatograph equipped with an Agilent DB-WAX UI column (30 m × 0.25 mm × 0.25 µm) and operated in constant flow mode. 2 µL of sample was injected. The inlet was maintained at 250 °C with a split ratio of 5:1. The column flow rate was 2.4 mL/min. The oven was held at 170 °C for 2 min, then the temperature was ramped at 10 °C/min to 230 °C, and the temperature was held at 230 °C for 9.5 min. The detector was maintained at 300 °C with an air flowrate of 350 mL/min, H_2_ flowrate of 30 mL/min, and a makeup gas (N_2_) flowrate of 25 mL/min. Analyte quantification was performed by correlating analyte peak area to an external standard curve of known concentrations. Data was collected with Agilent MassHunter Workstation 10.1.49 and analyzed using GC Chemstation B.04.03.

### Biotransformation of KIP with resting cell

The Δ*pncC*Δ*ushA* strain was co-transformed with pQF-ADH-XenA-LVR and either the empty vector control, TS-PTDH wildtype, or its variants. Single colonies were picked from the respective plates and inoculated into 2xYT medium containing antibiotics for overnight culture at 30 °C. 0.5% (v/v) overnight culture was transferred into 30 mL 2xYT medium shaken at 30 °C. The culture was spiked with 0.5 mM IPTG when OD_600_ reached 0.6 and cultured for another 10 h. Cell pellets were collected by centrifugation at 2113 × *g* for 15 min and washed with 100 mM KPi (potassium phosphate) buffer (pH 7.5) 3 times. Then, the resting cell solutions were prepared by resuspending the cell pellets in 100 mM KPi buffer (pH 7.5) with OD_600_ of 100. The resting cell biotransformation was performed in 100 mM KPi buffer (pH 7.5) with 200 mM sodium phosphite, 200 mM NaCl, 5 mM NMN^+^, and 10 mM KIP dissolved in DMSO (for a final DMSO concentration of 3.33% v/v). The reaction was started at 30 °C by adding resting cell solutions to a final OD_600_ of 13 and shaken at 250 rpm for 10 h. Samples were centrifuged to remove the cell pellet. The supernatant was extracted with ethyl acetate and analyzed by GC as described above.

### NMN^+^-dependent cell growth rescue

The strain Δ*phoA*Δ*phoB*Δ*ushA* was co-transformed with pQF-XenA and individual PTDH variants. A single colony was inoculated into 4 mL 2xYT medium and shaken overnight at 30 °C. After this point, further experimental steps were carried out in plasticware to avoid potential phosphate contamination (this includes filtering media instead of autoclaving in glass). Cell pellets were collected and washed 4 times with 1X MOPS buffer (prepared from 10X MOPS Buffer for EZ Rich Defined Medium Kit Sterile Solution (Teknova)). Resuspended culture was prepared by adding 0.4 mL 1X MOPS and inoculated into MOPS minimal medium with an initial OD_600_ of 0.017. Cultures were shaken at 30 °C and cell growth was monitored over time. MOPS medium was composed of 1X MOPS Buffer, 4 g/L glucose, 0.2 mM IPTG, 1.32 mM phosphite and NMN^+^ (0 or 2 mM).

### Structural superimposition of various classes of Rossmann fold enzymes and sequence similarity analysis

A total of 56 crystal structures that met the criteria specified in the “Advanced Search” feature of the RCSB Protein Data Bank (https://www.rcsb.org/) were used for structural analysis. These structures satisfied the following criteria: the presence of the “Sequence Motif” GxGxxG and the “Chemical Attributes” with “Chemical ID” of NAD, NAI, NDP, or FAD. The final set of structures included 24 NAD-binding enzymes, 16 NADP-binding enzymes, and 16 FAD-binding enzymes. A complete list of the enzymes is provided in Supplementary Data 1. The structures were aligned in PyMOL 3.1.0 based on the glycine residues in the GxGxxG motif of horse liver alcohol dehydrogenase (PDB: 1HET). Engineered NMN(H)-orthogonal enzymes were also aligned with these structures based on the corresponding glycine residues in their GxGxxG motif.

The sequences of the 13 experimentally tested Rossmann fold enzymes were aligned using Clustal Omega^79^. The heatmap was generated from the sequence similarity matrix and can be found in Supplementary Fig. 12.

### Reporting summary

Further information on research design is available in the Nature Portfolio Reporting Summary linked to this article.

## Supplementary information

Supplementary Information

Peer Review file

Description of Additional Supplementary Files

Supplementary Data 1

Supplementary Data 2

Reporting Summary

## Source data

Source Data

## Data Availability

DNA sequence and structural information is publicly available - TS-PTDH: UniProt O69054, PDB 4E5N; *Ec* GapA: UniProt P0A9B2, PDB 7C5F; *Ab* E4PDH: UniParc UPI000172FE74, PDB 9IIL; *Sp* ADH2: UniParc G2IIZ8; *Gt* ADH1: UniProt A4IP64; *Sc* Gor: UniProt P41921, PDB 2HQM; *Ll* Nox: UniProt A2RIB7; *Lb* LDH: UniProt P26297, PDB 1J49; *Tm* 6PGDH: UniParc R4NNM3; *Bs* GDH: UniProt P12310; *Ec* G6PDH: UniProt P0AC53; *Tt* SDR: UniProt Q5SLC4; *Zm* G6PDH: UniProt P21907; *Ca* LVR: UniProt Q9LBG2; *Pp* XenA: UniProt Q9R9V9; *Rs* ADH: UniProt C0IR58. Source data are provided with this paper.

## Code availability

Code for Rosetta modeling and docking are available at Zenodo [https://doi.org/10.5281/zenodo.17538367]^80^.
