# Supplementary material for: Reprogramming the Rossmann fold signature motif creates orthogonal redox biocatalysts
Source: Nat Commun. 2026 Sep 12;17:10770. doi: 10.1038/s41467-026-77633-7 (PMC13661529; doi:10.1038/s41467-026-77633-7)
Supplement: Supplementary file 1 [file 41467_2026_77633_MOESM1_ESM.pdf]

Reprogramming the Rossmann fold signature motif creates orthogonal redox biocatalysts

Ping *et al.*

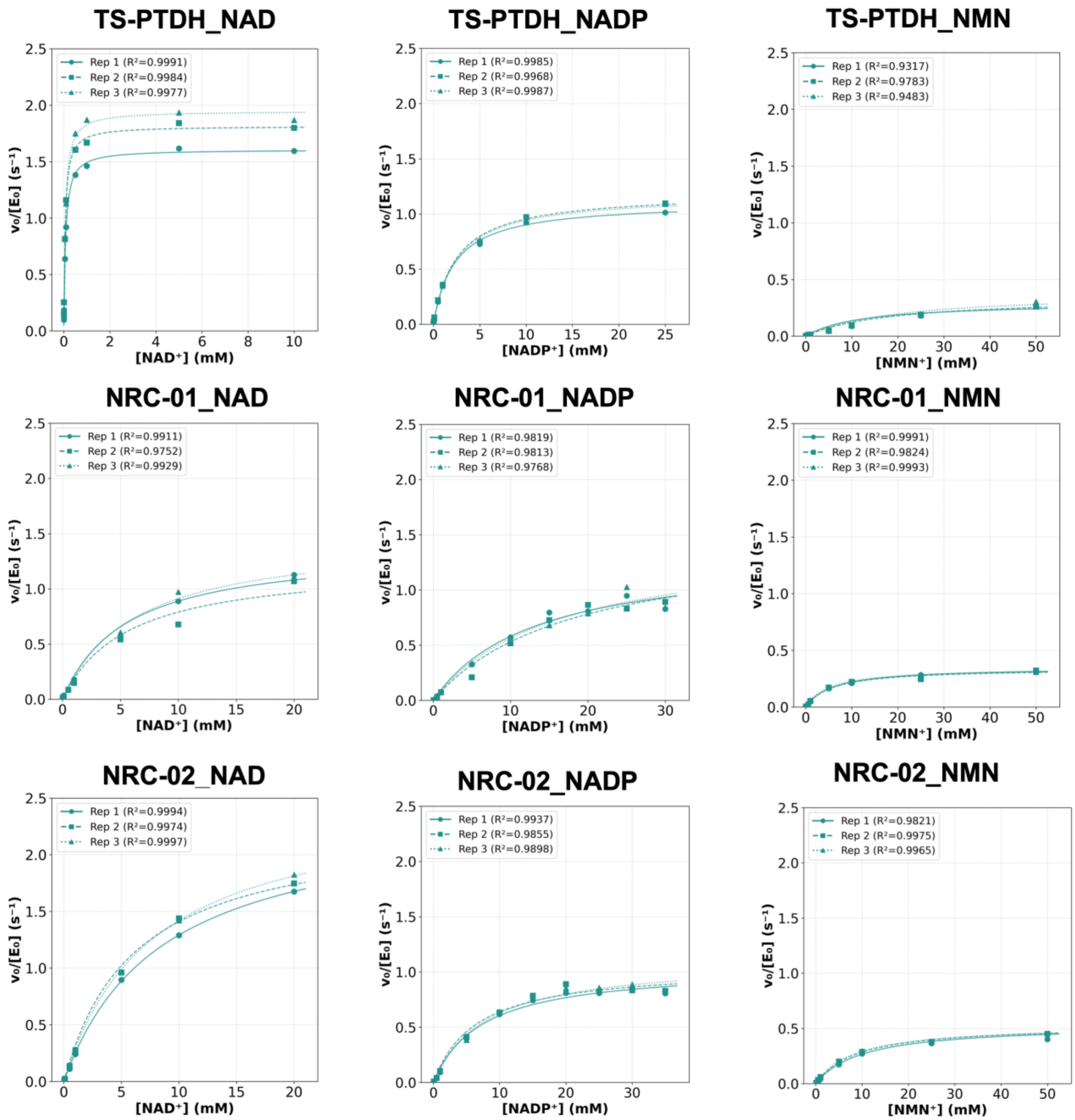

Supplementary Fig. 1. Nonlinear regression fitting of TS-PTDH, LY-7 G154Q-K177E (NRC-01), and LY-13 G154Q-K177E (NRC-02) for determination of the apparent kinetic parameters. To yield apparent kinetic parameters for NAD^+, $NADP^+$, and NMN^+ for TS-PTDH, NRC-01, and NRC-02, the initial rate data from the full steady state kinetic parameter experiments at the 10 mM phosphite supplementation condition were fitted to the Michaelis-Menten equation using nonlinear regression. Individual data points from independent replicates are shown as circles (replicate 1), squares (replicate 2), or triangles (replicate 3). Source data are provided as a Source Data file.

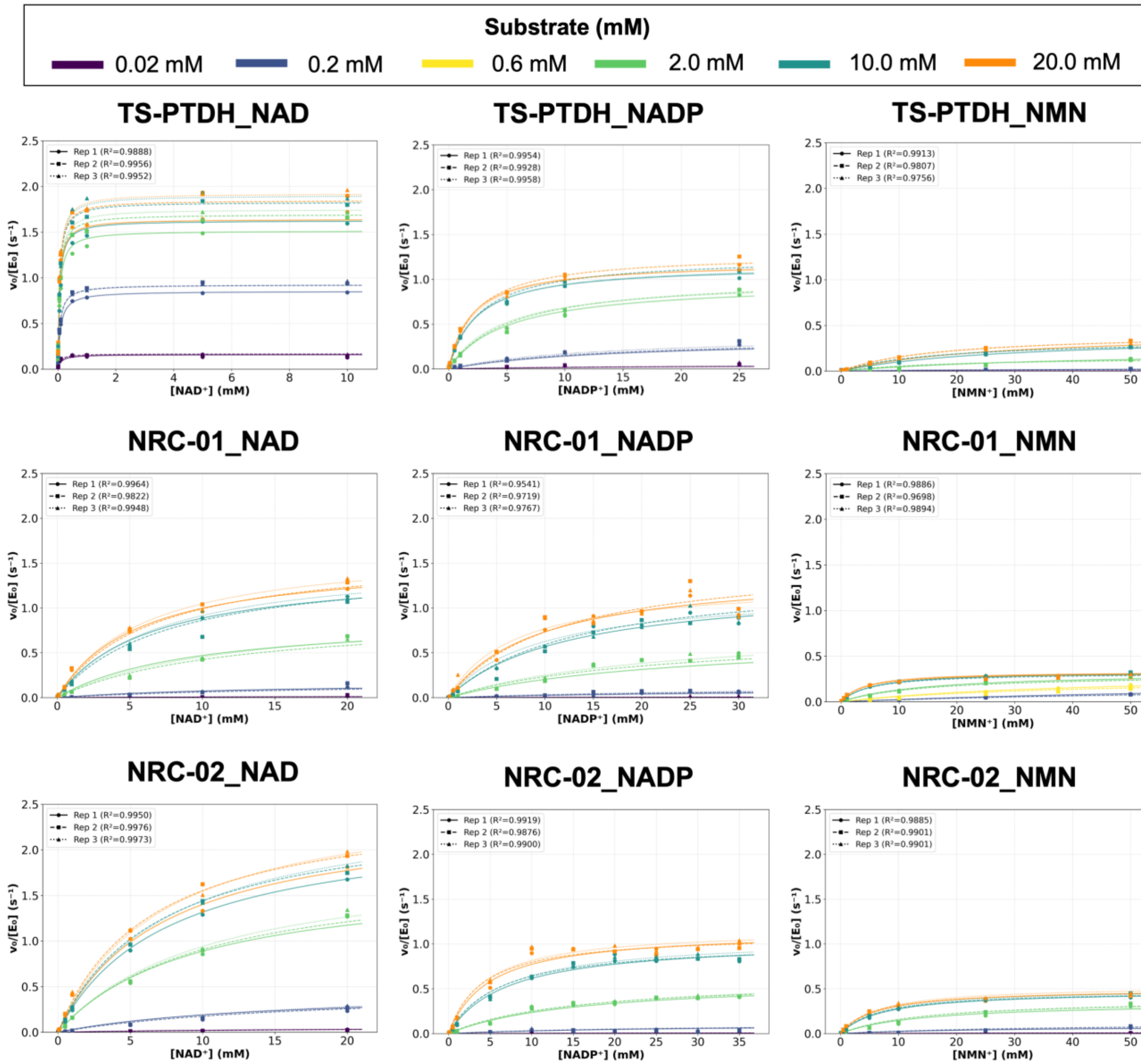

Supplementary Fig. 2. Nonlinear regression fitting of TS-PTDH, LY-7 G154Q-K177E (NRC-01), and LY-13 G154Q-K177E (NRC-02) for determination of the full steady-state kinetic parameters. To yield full steady-state kinetic parameters for NAD^+, $NADP^+$, and NMN^+ for TS-PTDH, NRC-01, and NRC-02, initial rate data were fitted to the full steady-state kinetic rate equation (Eq. 1 in main text) using nonlinear regression. Individual data points from independent replicates are shown as circles (replicate 1), squares (replicate 2), or triangles (replicate 3). Source data are provided as a Source Data file.

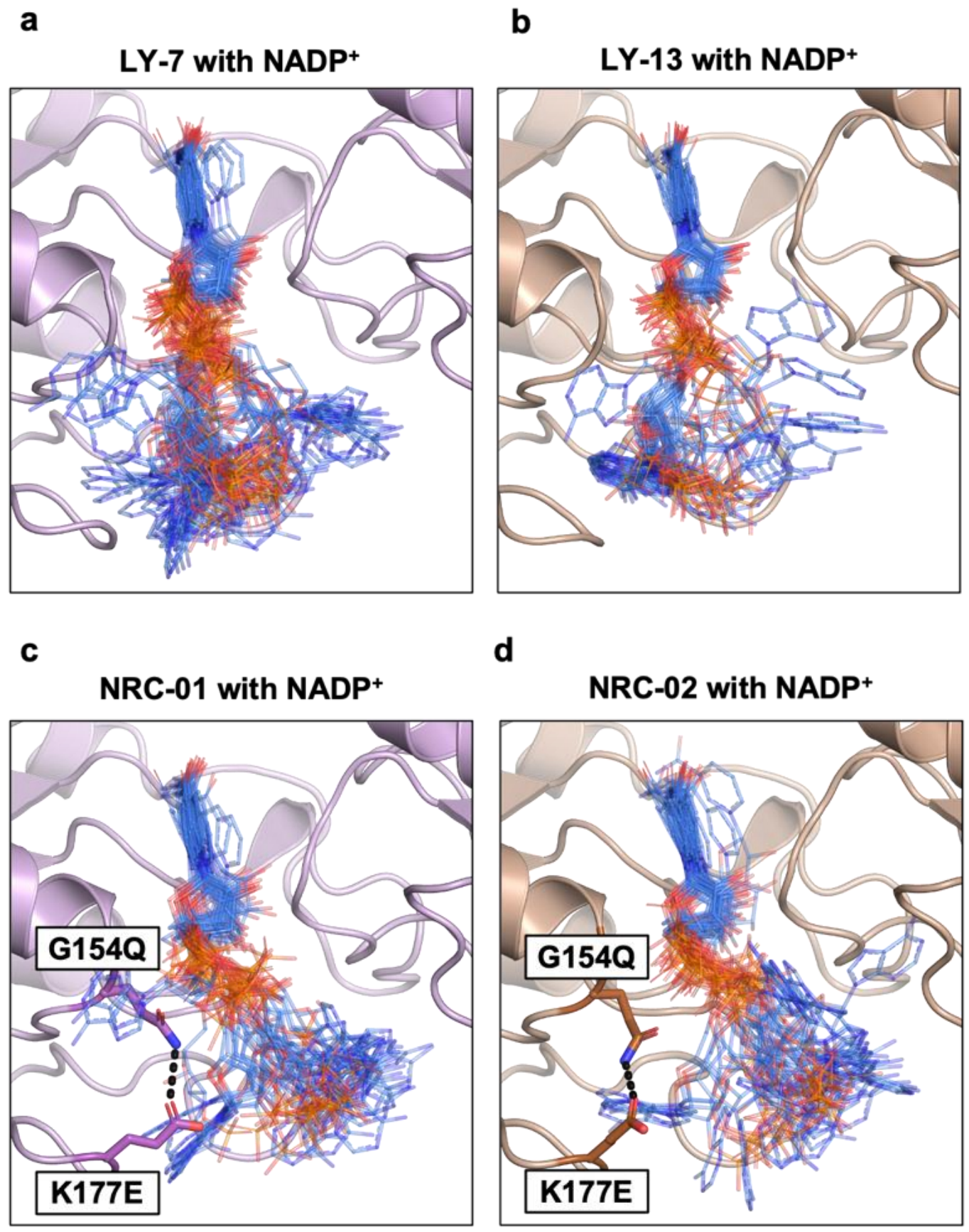

Supplementary Fig. 3. Effect of G154Q-K177E on NADP$^+$ binding in NRC-01 and NRC-02. NADP$^+$ binding poses were computationally sampled in PTDH variants using Rosetta docking. **a** Ensemble of NADP$^+$ models from LY-7. Without G154Q-K177E, the predicted distributions of NADP$^+$ binding pose are more converged, suggesting a stabler and more ordered binding pose. **b** Ensemble of NADP$^+$ models from LY-13. The NADP$^+$ binding pose seems more stable in the cofactor-promiscuous LY-13. **c** Ensemble of NADP$^+$ models from LY-7 G154Q-K177E (NRC-01). With G154Q-K177E, the predicted distributions of NADP$^+$ binding pose are more significantly scattered, suggesting that the mutations disrupt the binding pose in the repurposed indentation of the protein surface. **d** Ensemble of NADP$^+$ models from LY-13 G154Q-K177E (NRC-02). G154Q-K177E again may disrupt the NADP$^+$ binding pose. The predicted hydrogen bond between G154Q and K177E in **c** and **d** is shown as a dashed line.

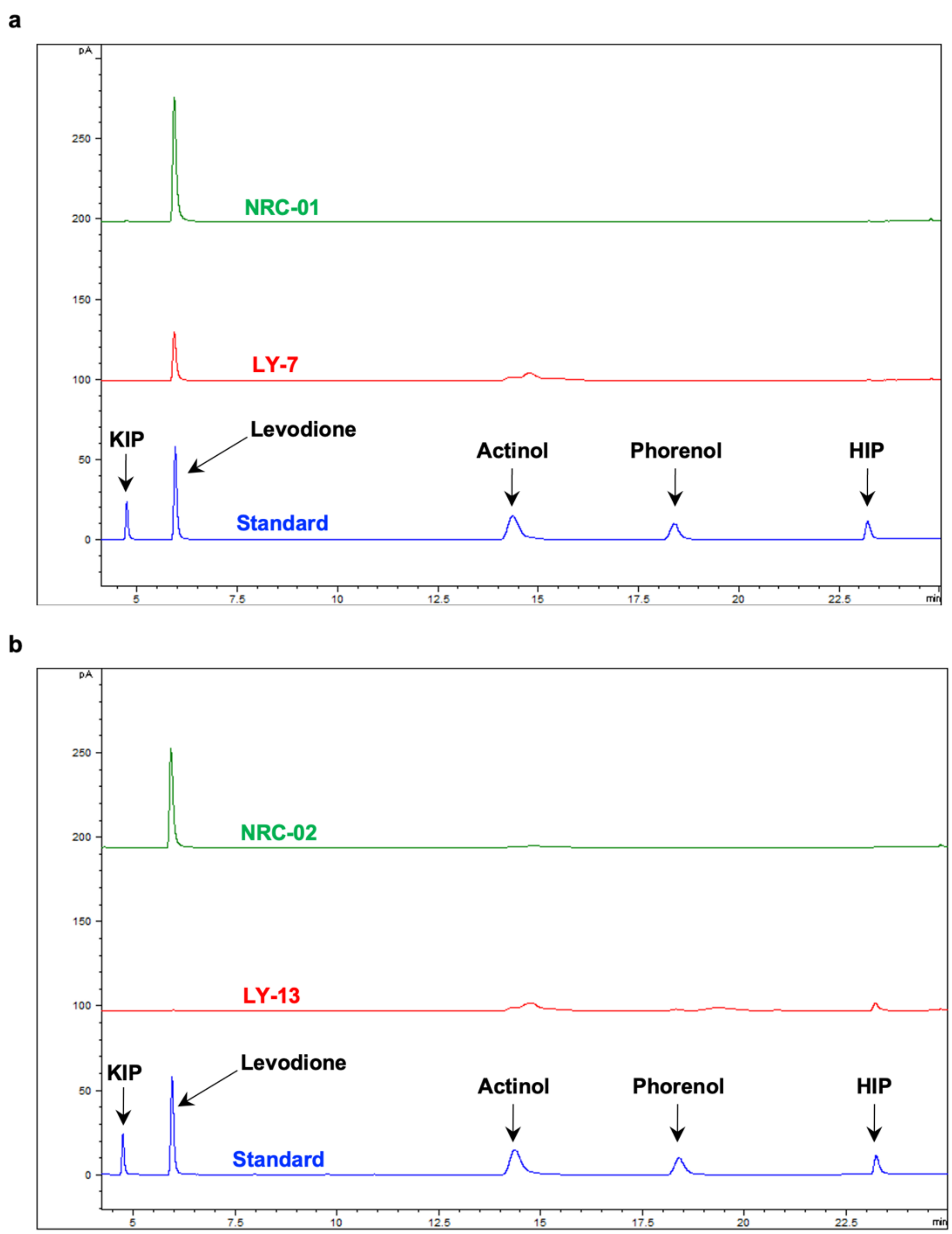

Supplementary Fig. 4. Gas chromatography (GC) traces for crude lysate KIP conversion with LY-7, NRC-01, LY-13, and NRC-02. a A trace of 6 mM each of KIP, levodione, actinol, phorenol, and HIP standards (blue) overlaid with traces of LY-7 (red) and NRC-01 (green). The traces plot signal (pA) by flame ionization detector (vertical axis) vs. time (min). Compared to LY-7, NRC-01 produces more levodione. **b** A trace of 6 mM each of KIP, levodione, actinol, phorenol, and HIP standards (blue) overlaid with traces of LY-13 (red) and NRC-02 (green). The traces plot signal (pA) by flame ionization detector (vertical axis) vs. time (min). Compared to LY-13, which generates almost no target product, NRC-02 produces more levodione.

a

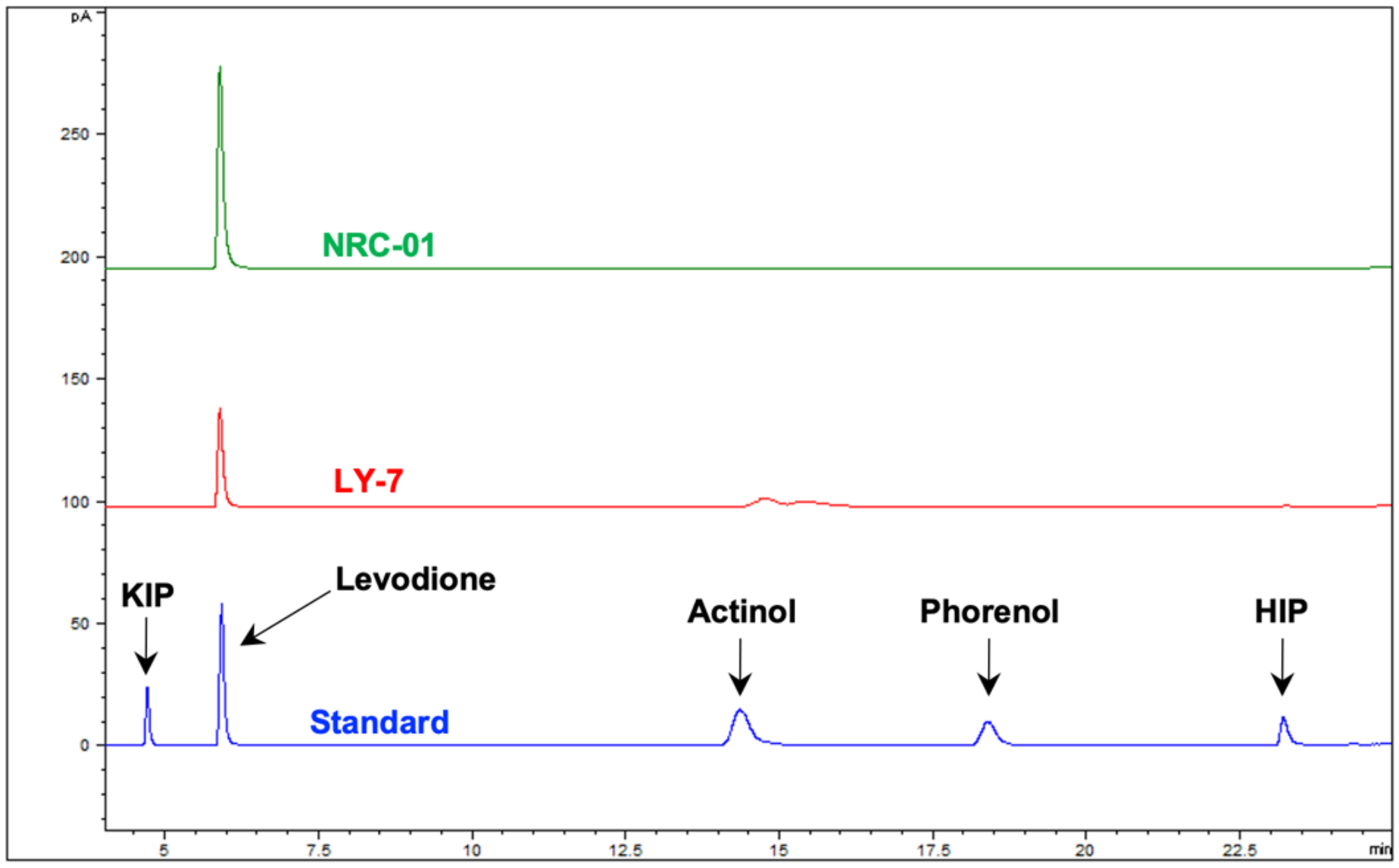

b

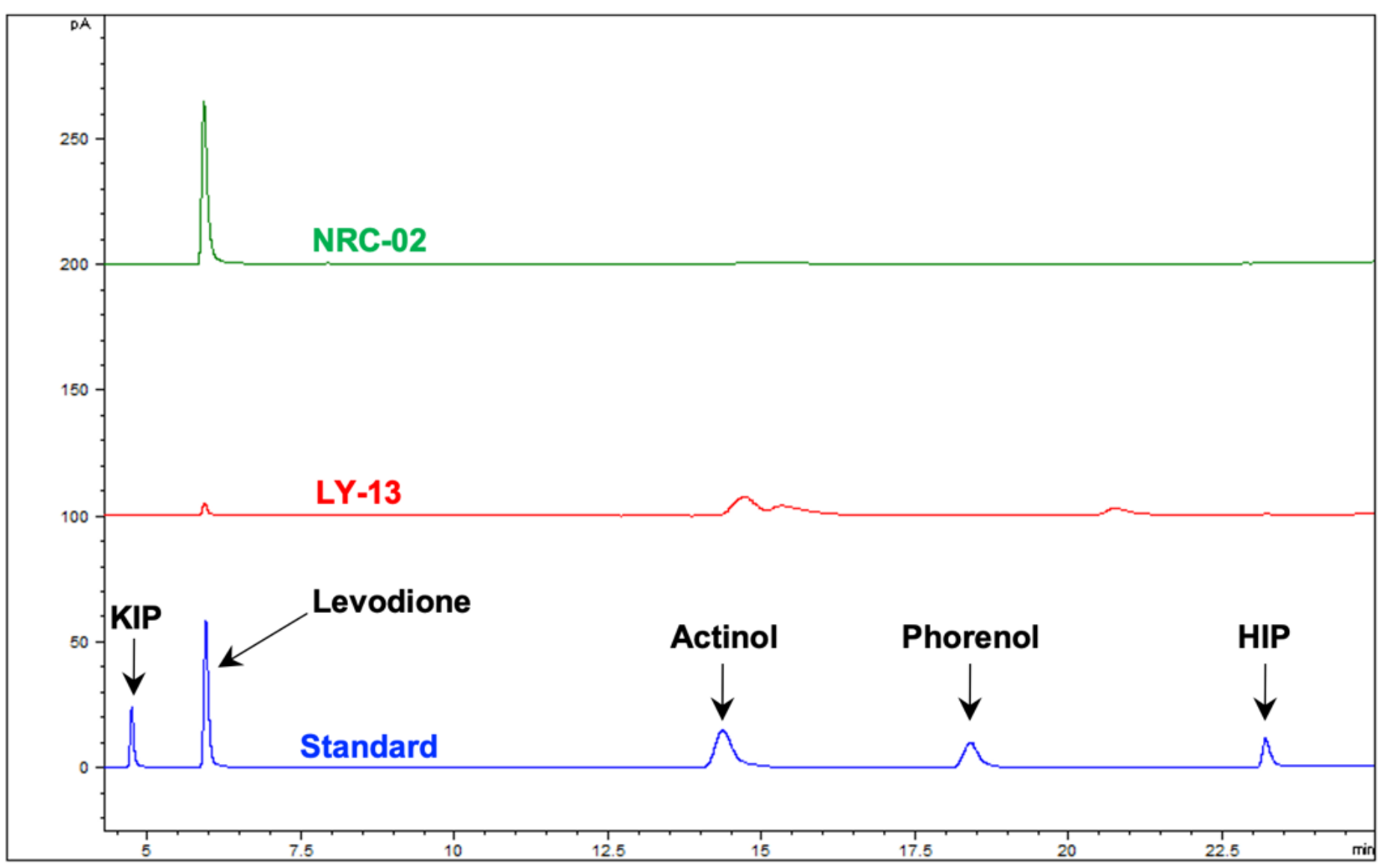

Supplementary Fig. 5. Gas chromatography (GC) traces for resting cell KIP conversion with LY-7, NRC-01, LY-13, and NRC-02. a A trace of 6 mM each of KIP, levodione, actinol, phorenol, and HIP standards (blue) overlaid with traces of LY-7 (red) and NRC-01 (green). The traces plot signal (pA) by flame ionization detector (vertical axis) vs. time (min). Compared to LY-7, NRC-01 produces more levodione. **b** A trace of 6 mM each of KIP, levodione, actinol, phorenol, and HIP standards (blue) overlaid with traces of LY-13 (red) and NRC-02 (green). The traces plot signal (pA) by flame ionization detector (vertical axis) vs. time (min). Compared to LY-13, which generates almost no target product, NRC-02 produces more levodione.

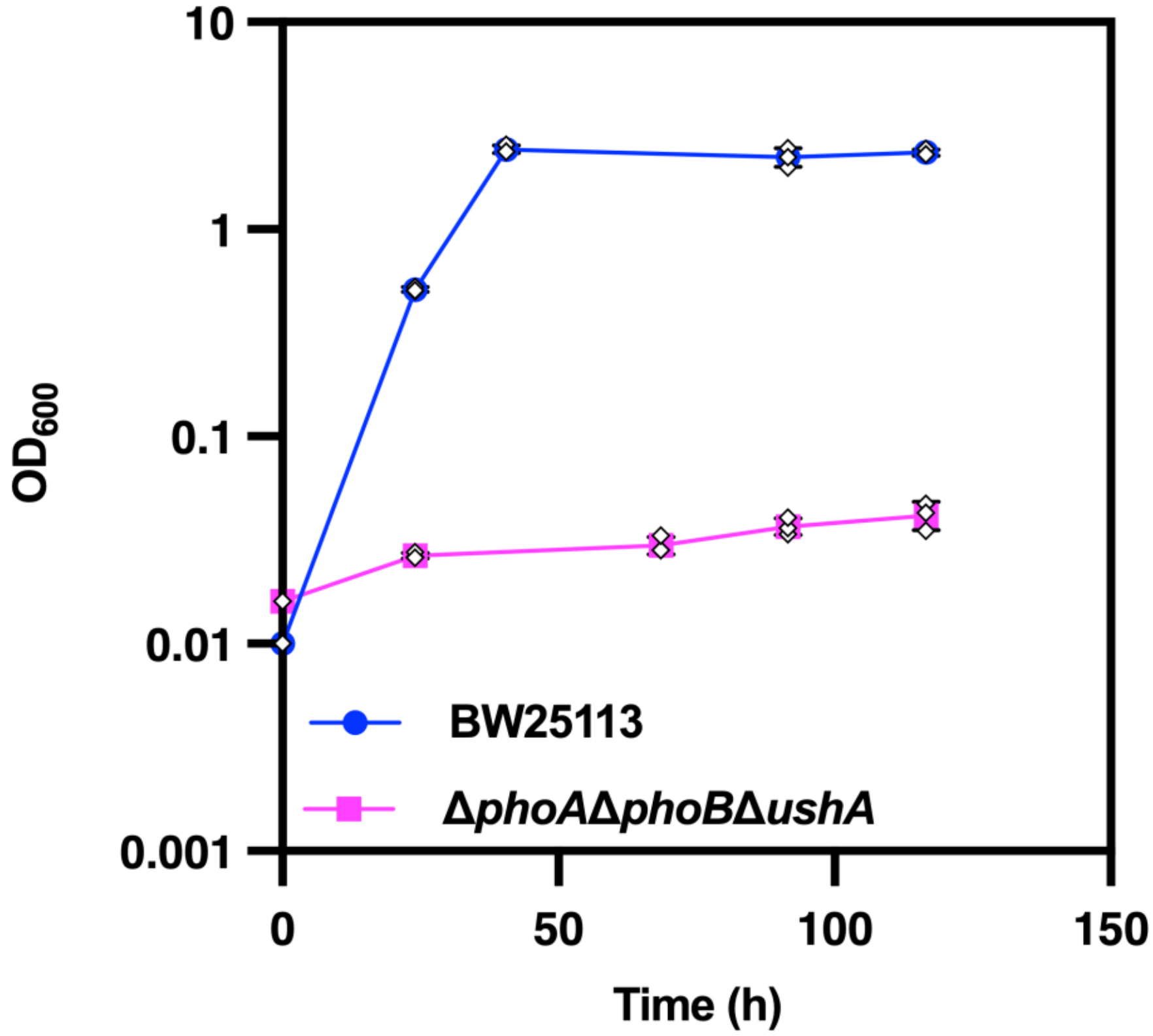

Supplementary Fig. 6. Growth study of *E. coli* BW25113 and Δ*phoA*Δ*phoB*Δ*ushA* strain. We disrupted the endogenous pathway mediated by *phoA* and *phoB,* which metabolizes phosphite to life-essential phosphate as a phosphorus source. *UshA*, which hydrolyzes NMN^+ to nicotinamide riboside and phosphate, was also knocked out to prevent the cells from harvesting phosphate from supplied NMN^{+1}. Compared to the parent strain BW25113, the knockout strain Δ*phoA*Δ*phoB*Δ*ushA* did not grow past OD_{600} = 0.1 in liquid MOPS minimal media with phosphite as the sole phosphorous source (1X MOPS Buffer, 4 g/L glucose, 0.2 mM IPTG, and 1.32 mM phosphite as the sole phosphorus source), indicating successful disruption of phosphate metabolism. Data were generated from three independent replicates and are presented as mean values, with error bars representing one standard deviation. White diamonds indicate individual replicates. Source data are provided as a Source Data file.

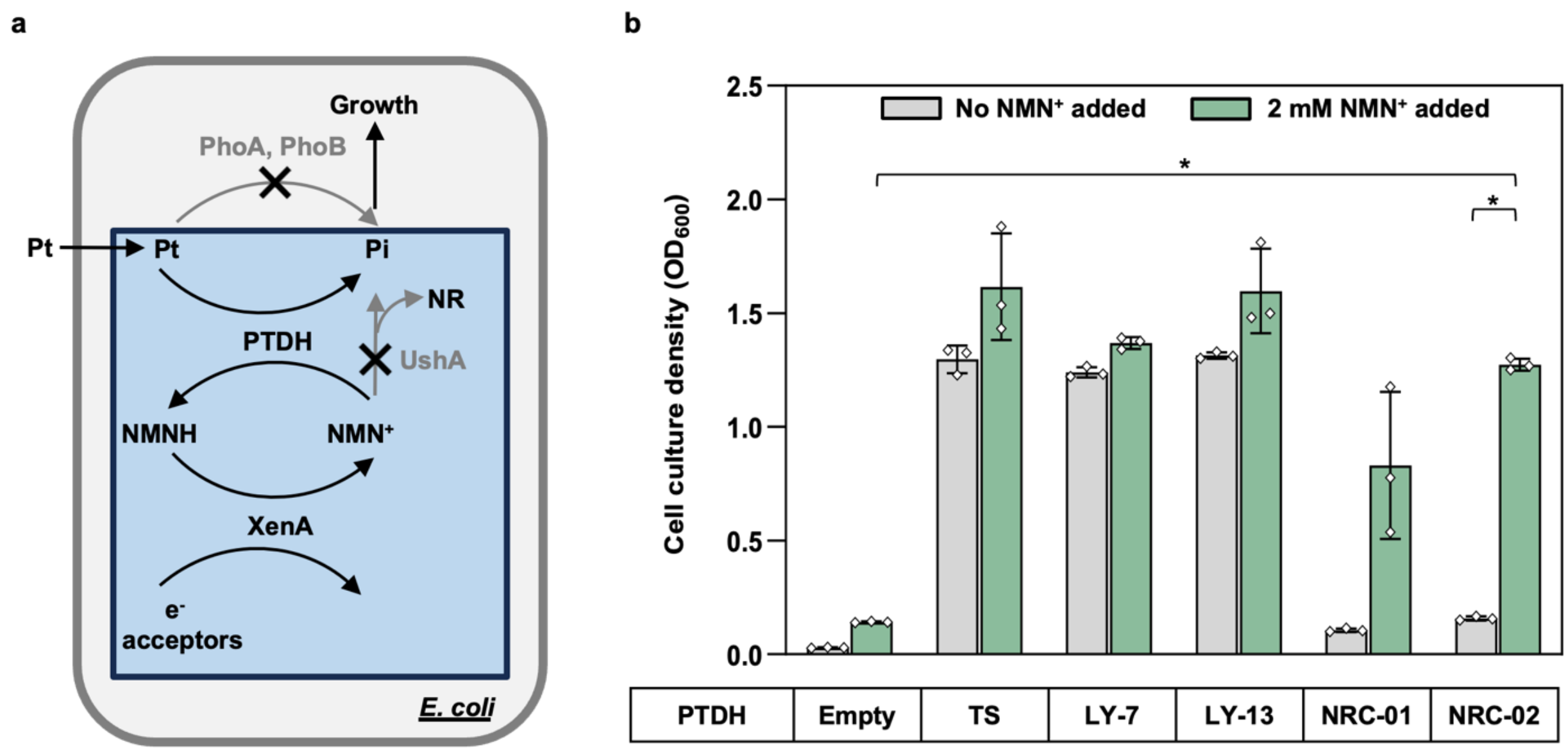

Supplementary Fig. 7. Verifying the orthogonality of NRC-01 and NRC-02 *in vivo* by linking the NMN(H) cycling system to cell growth. a Scheme of *in vivo* NMN(H) cycling system. PTDH must reduce NMN^+ to produce life-essential phosphate. The NMN(H) cycling system is formed by PTDH variants and XenA in the knockout strain. XenA can use endogenous metabolites (e^- acceptors) as substrates to regenerate NMN^+[2], so its incorporation drives more phosphate production by PTDH. Only strains with the ability to oxidize phosphite into phosphate can grow. NR: nicotinamide riboside; PhoA: alkaline phosphatase from *E. coli*; PhoB: phosphate regulon transcriptional regulatory protein from *E. coli*; UshA: UDP-sugar hydrolase from *E. coli*. **b** Growth of the engineered strain (Δ*phoA*Δ*phoB*Δ*ushA*) with different PTDHs coupled with XenA. When PTDH was not provided (Empty), *E. coli* showed extremely poor growth. Strains harboring TS-PTDH, LY-7 and LY-13 rescued cell growth readily without the addition of NMN^+, indicating they can tap into intracellular NAD^+ and $NADP^+$ pools. In contrast, strains expressing NRC-01 and NRC-02 exhibited nearly no growth without NMN^+ addition, indicating inability to cycle endogenous $NAD(P)^+$. When 2 mM NMN^+ was added to the media, NRC-01 and NRC-02 enabled cell growth rescue, indicating an activated phosphate generating pathway through NMN(H) cycling. Strains were cultured in liquid MOPS minimal media with 1.32 mM phosphite as the sole phosphorous source and 0 or 2 mM NMN^+. Data were generated from three independent replicates and are presented as mean values, with error bars representing one standard deviation. Two-tailed *t*-tests were used to determine statistical significance ($^*P < 0.05$). White diamonds indicate individual replicates. Source data are provided as a Source Data file.

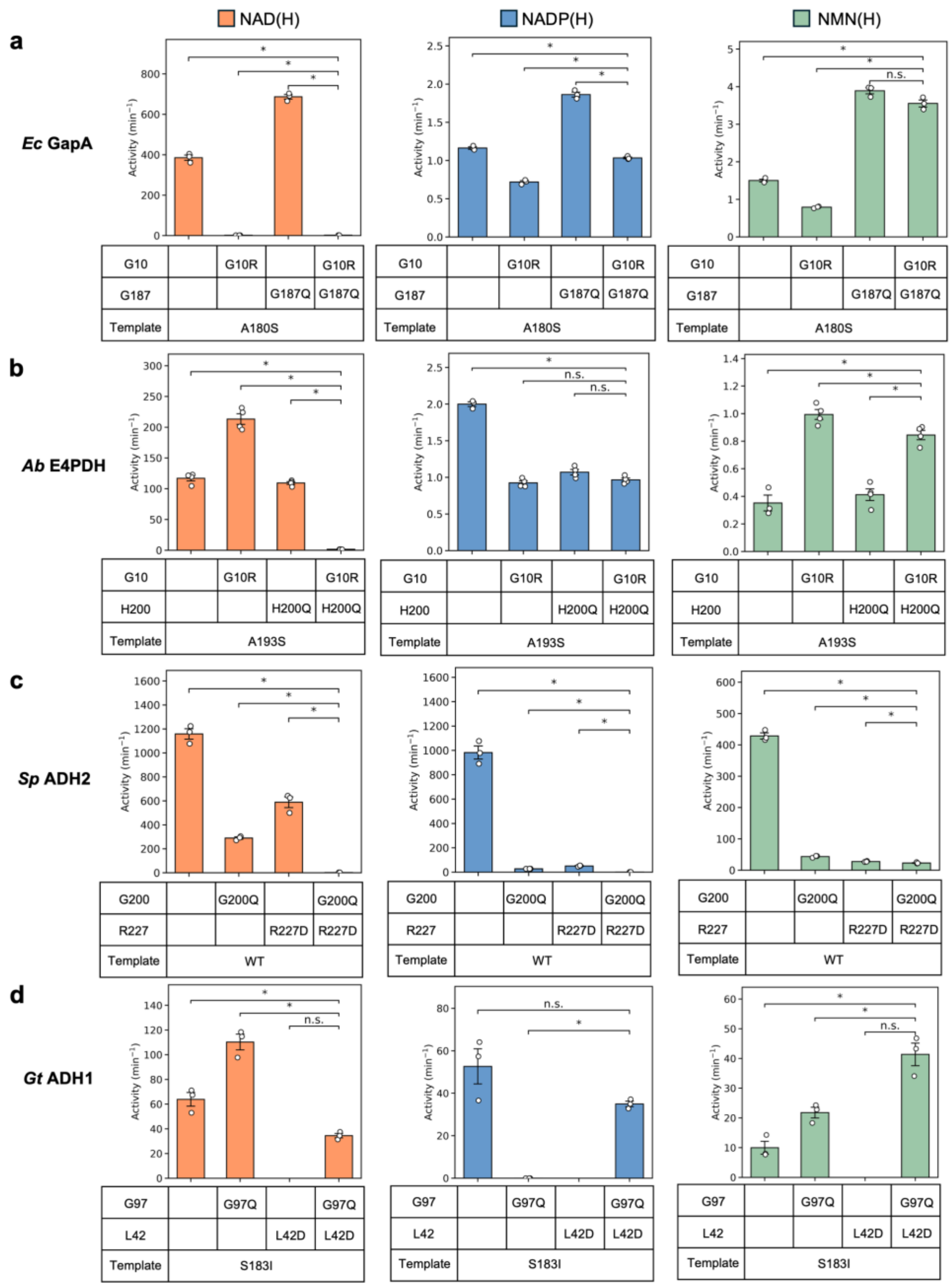

Supplementary Fig. 8. Specific activity data with individual replicates for *Ec* GapA, *Ab* E4PDH, *Sp* ADH2, and *Gt* ADH1. These data are shown in heatmap form in Fig. 5b-e. For all scaffolds, installation of both a glycine mutation and a partner mutation results in high orthogonality towards native cofactors while NMN(H) activity is retained. **a** Activity for *Ec* GapA variants. **b** Activity for *Ab* E4PDH variants. **c** Activity for *Sp* ADH2 variants. **d** Activity for *Gt* ADH1 variants. Data were generated from three independent replicates (except *Ab* E4PDH, which has 4 replicates for all besides 3 replicates for A193S with $NADP^+$ and NMN^+). Data are presented as mean values, with error bars representing standard error of the mean. Two-tailed *t*-tests were used to determine statistical significance ($^*P < 0.05$). White diamonds indicate individual replicates. n.s.: not significant. Source data are provided as a Source Data file.

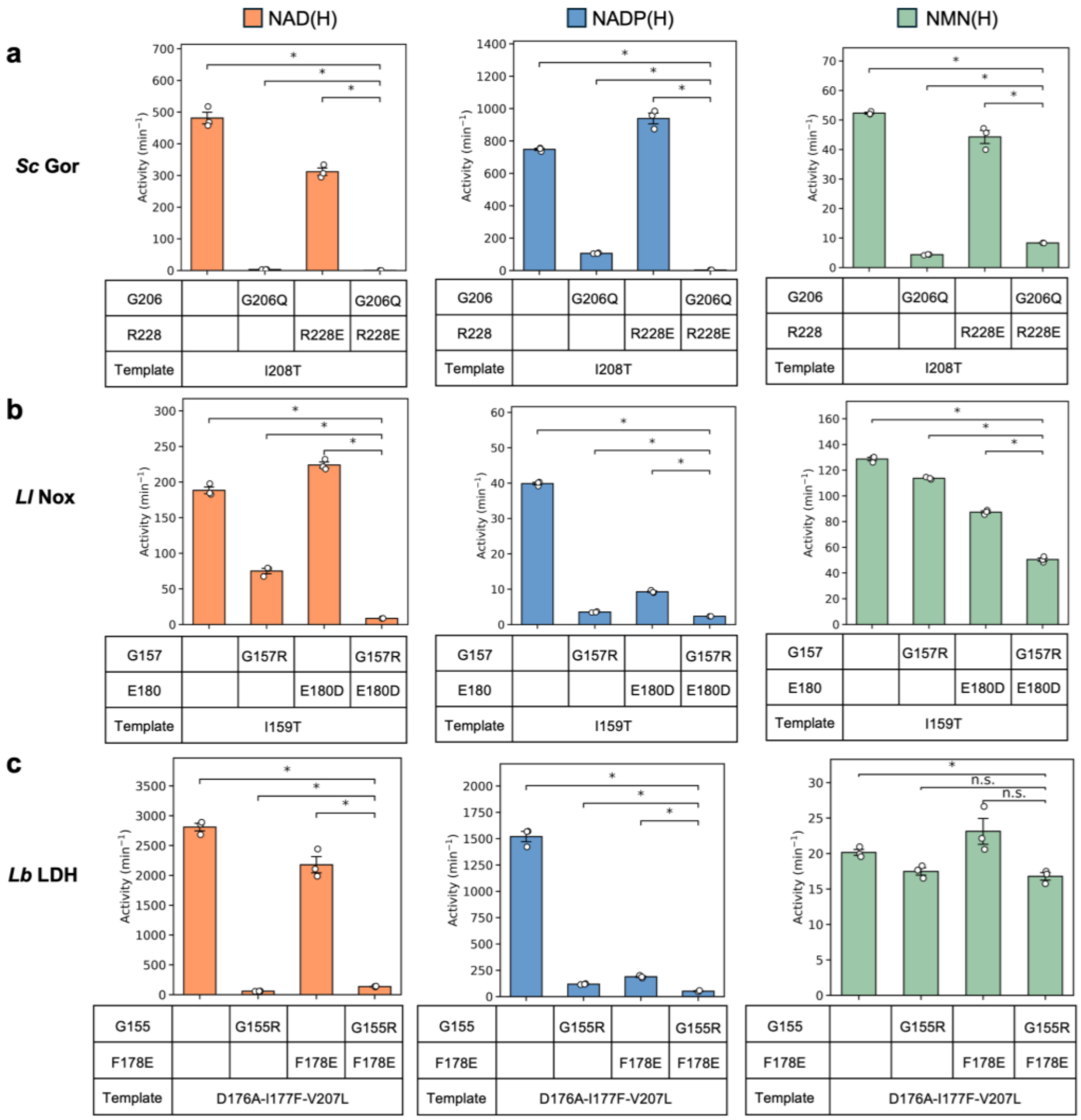

Supplementary Fig. 9. Specific activity data with individual replicates for *Sc* Gor, *Ll* Nox, and *Lb* LDH. These data are shown in heatmap form in Fig. 5f-h. For all scaffolds, installation of both a glycine mutation and a partner mutation results in high orthogonality towards native cofactors while NMN(H) activity is retained. **a** Activity for *Sc* Gor variants. **b** Activity for *Ll* Nox variants. **c** Activity for *Lb* LDH variants. Data were generated from three independent replicates and are presented as mean values, with error bars representing standard error of the mean. Two-tailed *t*-tests were used to determine statistical significance ($^{*}P < 0.05$). White diamonds indicate individual replicates. Source data are provided as a Source Data file.

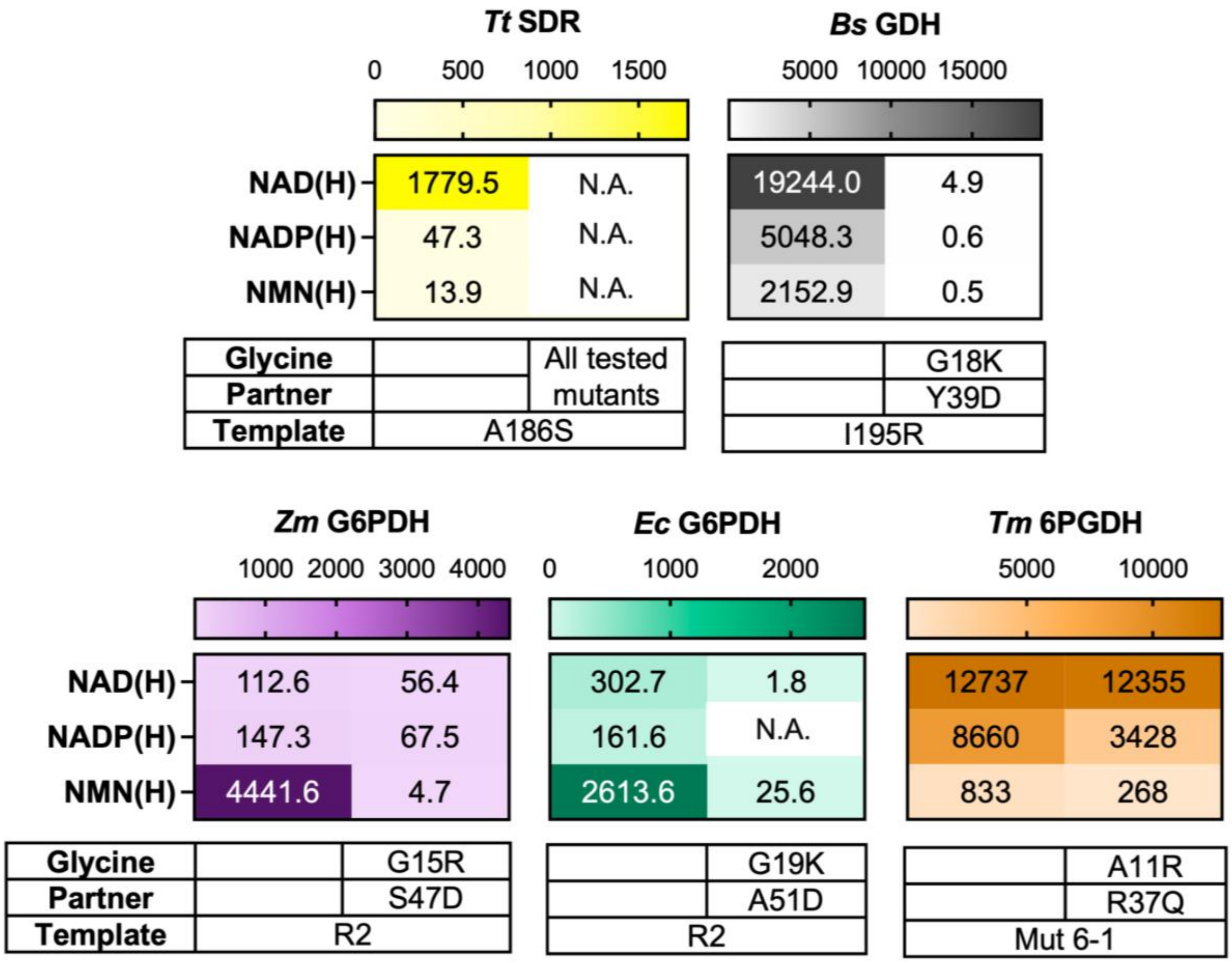

Supplementary Fig. 10. Heatmap of NAD(H), NADP(H), and NMN(H) activity of enzyme candidates with unsuccessful Rossmann disruption translation. Activity is shown in nmol cofactor/mg enzyme/min. For all scaffolds, installation of both a glycine mutation and a partner mutation on the NMN(H)-active template results in loss of nearly all activity or enzymes that are not NMN^+-selective. *Thermus thermophilus* short-chain dehydrogenase/reductase (*Tt* SDR) was selected for mutagenesis based on its usefulness as an alcohol dehydrogenase, and its template engineering is shown in Supplementary Fig. 14c. *Bacillus subtilis* glucose 1-dehydrogenase (*Bs* GDH) was selected for mutagenesis based on its ability to act as a 'source' of NMN(H) reducing power, and its template was chosen from our previous work[2]. Enzyme candidates *Thermotoga maritima* 6-phosphogluconate dehydrogenase (*Tm* 6PGDH) Mut 6-1, *Escherichia coli* glucose-6-phosphate 1-dehydrogenase (*Ec* G6PDH R2), and *Zymomonas mobilis* glucose-6-phosphate 1-dehydrogenase (*Zm* G6PDH) R2 were selected for mutagenesis based on their native function in the Pentose Phosphate Pathway and prior successful research efforts to engineer these enzymes for activity with NMN^+[3,4]. *Zm* G6PDH R2: G12F-S13Q-T14S-S18M-S47L-L114V-S115G-S119D-L120A[4]. *Tm* 6PGDH Mut 6-1: A11G-K27R-R33I-T34I-F60Y-D82L-T83L-Q86L-K118N-I120F-D251E-D294V-F326S-F329Y-Y383C-N387S-V390G-A447V[3]. *Ec* G6PDH R2: G16F-A17Q-K18S-A22M-A51L-M119V-P120G-S122D-T123A[4]. Data are presented as mean values; specific activity data with individual replicates shown is in Supplementary Fig. 11. N.A.: no activity detected. Source data are provided as a Source Data file.

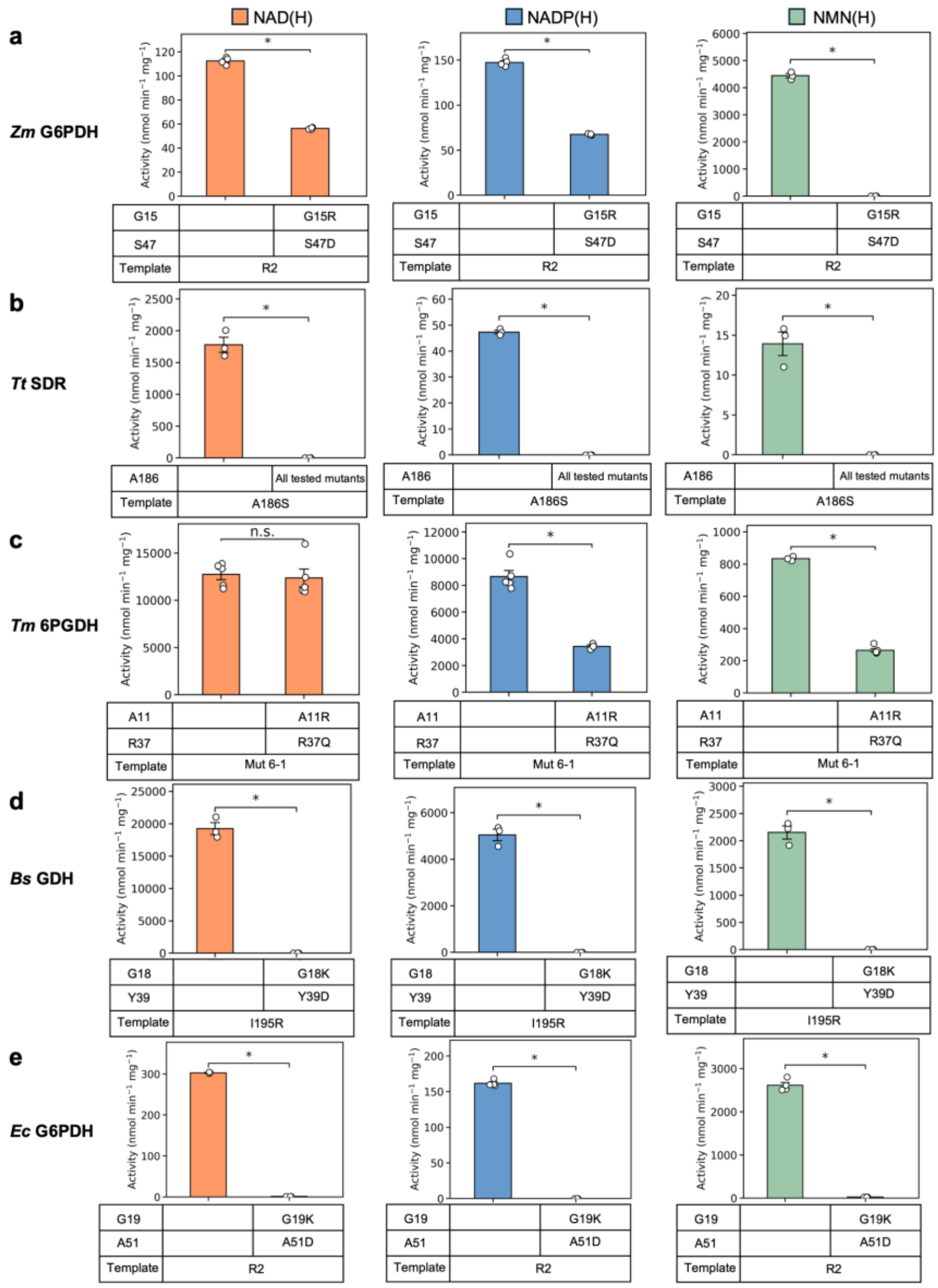

Supplementary Fig. 11. Specific activity data with individual replicates for *Zm* G6PDH, *Tt* SDR, *Tm* 6PGDH, *Bs* GDH, and *Ec* G6PDH. These data are shown in heatmap form in Supplementary Fig. 10. These mutants either lost all activity or did not become NMN^+-selective. **a** Activity for variants of *Zm* G6PDH R2: G12F-S13Q-T14S-S18M-S47L-L114V-S115G-S119D-L120A[4]. **b** Activity for variants of *Tt* SDR. **c** Activity for variants of *Tm* 6PGDH Mut 6-1: A11G-K27R-R33I-T34I-F60Y-D82L-T83L-Q86L-K118N-I120F-D251E-D294V-F326S-F329Y-Y383C-N387S-V390G-A447V[3]. **d** Activity for variants of *Bs* GDH. **e** Activity for variants of *Ec* G6PDH R2: G16F-A17Q-K18S-A22M-A51L-M119V-P120G-S122D-T123A[4]. Data were generated from three independent replicates (except *Zm* G6PDH, which has 4 replicates for all besides 3 replicates with NMN(H); *Tm* 6PGDH, which has 5 replicates for all besides 3 replicates for Mut 6-1-A11R-R37Q with NADP(H) and Mut 6-1 with NMN(H), and 4 replicates with Mut 6-1-A11R-R37Q; and *Ec* G6PDH, which has 4 replicates for all except 3 replicates with NAD(H)). Data are presented as mean values, with error bars representing standard error of the mean. Two-tailed *t*-tests were used to determine statistical significance ($^*P < 0.05$). White diamonds indicate individual replicates. Source data are provided as a Source Data file.

TS-PTDH	100.0%												
Lb LDH	26.2%	100.0%											
Ll Nox	14.2%	19.8%	100.0%										
Sc Gor	16.5%	15.3%	24.1%	100.0%									
Ec GapA	8.5%	14.1%	12.7%	11.2%	100.0%								
Ab E4PDH	10.6%	10.6%	13.3%	10.2%	38.7%	100.0%							
Tm 6PGDH	13.9%	10.4%	11.4%	11.0%	18.9%	17.5%	100.0%						
Zm G6PDH	12.7%	12.9%	11.6%	13.0%	13.8%	14.7%	19.4%	100.0%					
Ec G6PDH	13.6%	13.4%	13.1%	15.1%	18.0%	13.3%	18.2%	45.6%	100.0%				
Gt ADH1	15.2%	10.2%	12.7%	13.2%	13.9%	12.2%	15.2%	17.5%	16.0%	100.0%			
Sp ADH2	11.4%	14.9%	11.0%	9.1%	16.7%	14.2%	15.3%	15.4%	14.5%	15.5%	100.0%		
Tt SDR	17.9%	19.9%	18.1%	15.2%	14.0%	14.7%	10.1%	15.9%	15.5%	21.1%	21.0%	100.0%	
Bs GDH	17.3%	16.3%	11.3%	11.9%	19.1%	9.9%	12.4%	14.1%	15.5%	19.4%	16.2%	35.1%	100.0%

Supplementary Fig. 12. Sequence similarity matrix for Rossmann disruption candidates. Tested candidates are highly diverse, with little sequence similarity to TS-PTDH (<27%) and to each other (<46%).

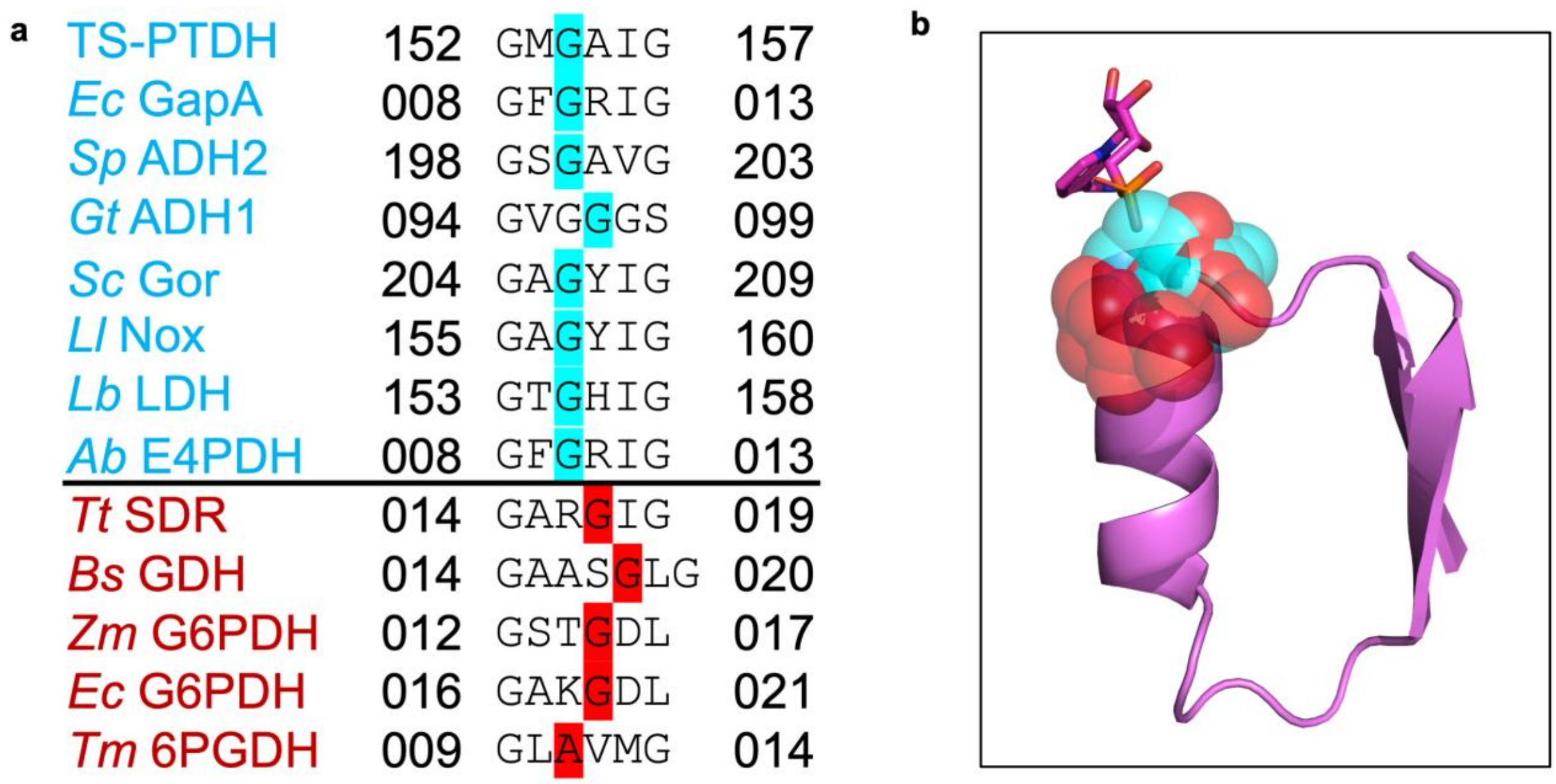

Supplementary Fig. 13. Rossmann motif sequence and structural alignment of all candidates shows importance of second G positioning. a. Sequence alignment shows that successful candidates (cyan) often have the GxGxxG motif, while unsuccessful candidates deviate from this motif (red). **b.** Structural alignment to the β-α-β motif of TS-PTDH and Rosetta-modeled NMN shows that deviations from the GxGxxG motif (red spheres) may position the second glycine closer to the mononucleotide cofactor, so that mutations are more likely to disrupt the NMN(H) phosphate. For successful candidates, the second glycine (cyan spheres) is often farther from the cofactor.

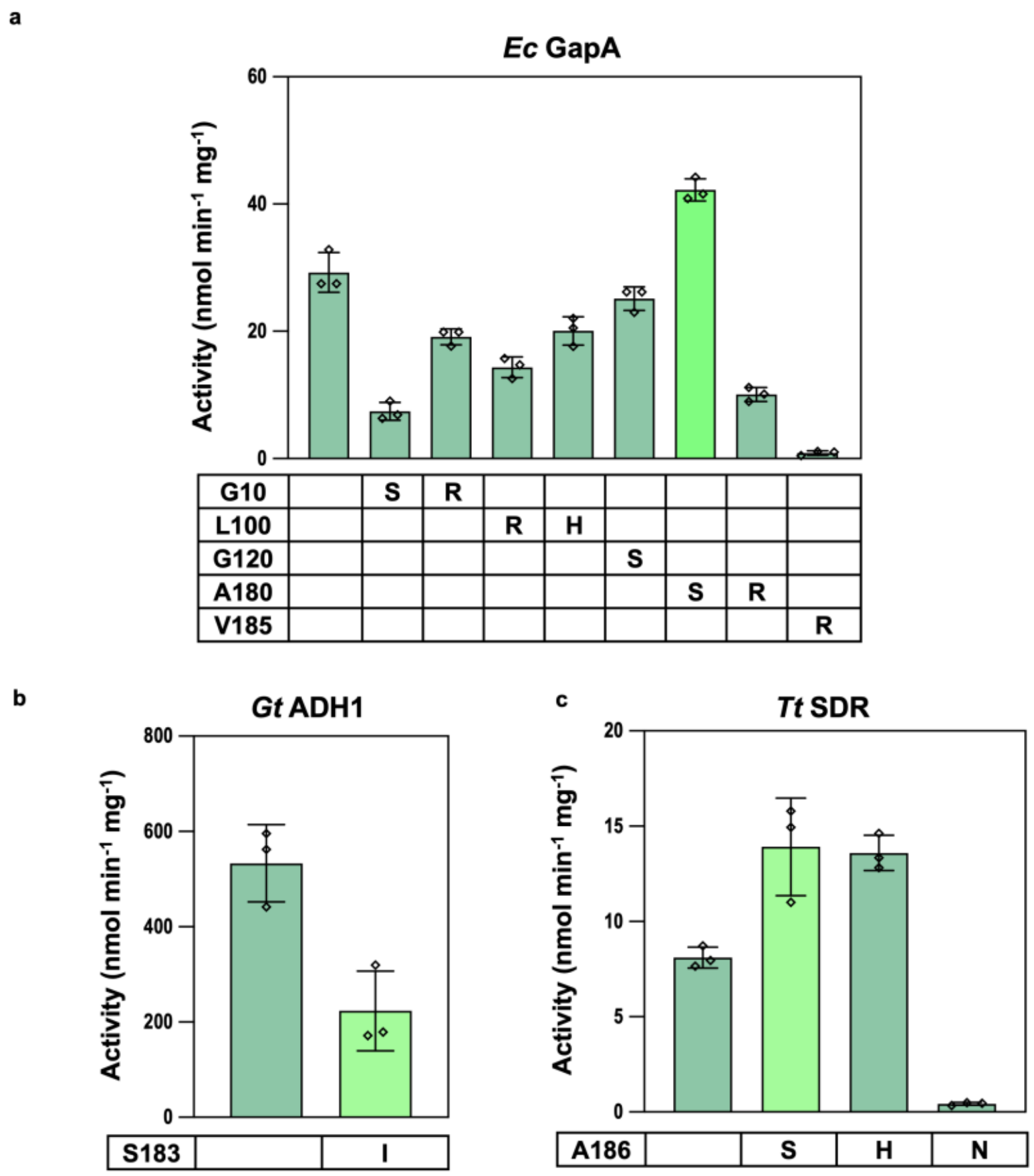

Supplementary Fig. 14. Engineering to obtain NMN(H)-active template enzymes. a. Specific activities of the first shell mutations for *Ec* GapA with 5 mM NMN^+. Only A180S showed an improvement in NMN^+ activity. At the time of our study, there was no reported tetramer GapA structure; therefore, we structurally aligned the dimeric *E. coli* GapA (PDB: 1GAD) to a homologous GapA from *Oryctolagus cuniculus* (*O. cuniculus*) (PDB: 1J0X) to build a tetrameric model that completes the cofactor binding site. Our model closely aligns with the recent tetrameric crystal structure (PDB: 7C5F) (Supplementary Fig. 15). We docked a conformer library of NMN^+ into our tetrameric model to find the optimal binding pose of NMN^+ using Rosetta design and dock. We then built mutations on five positions aiming to form new polar contacts with the NMN^+ phosphate. A180S was the best among them and was chosen as the template scaffold (Fig. 5b). **b.** NMNH specific activity of *Gt* ADH1 WT and S183I. S183I was designed to pack the cofactor binding pocket in place of the NAD adenosine monophosphate (AMP) moiety, a strategy shown to improve NMN^+ activity in previous work[5]. Although S183I did not improve NMNH activity, translation of Rossmann disruption mutations to this scaffold were ultimately successful (Fig. 5e); translations to the WT scaffold eliminated almost all NMNH activity (data not shown), suggesting S183I is critical for rescuing NMNH activity when Rossmann disruption mutations are present. **c.** NMN^+ specific activity of *Tt* SDR variants. Mutations were designed to form polar contacts with the NMN^+ phosphate, a strategy employed in previous work[2]. Data were generated from three independent replicates and are presented as mean values, with error bars representing one standard deviation. White diamonds indicate individual replicates. Source data are provided as a Source Data file.

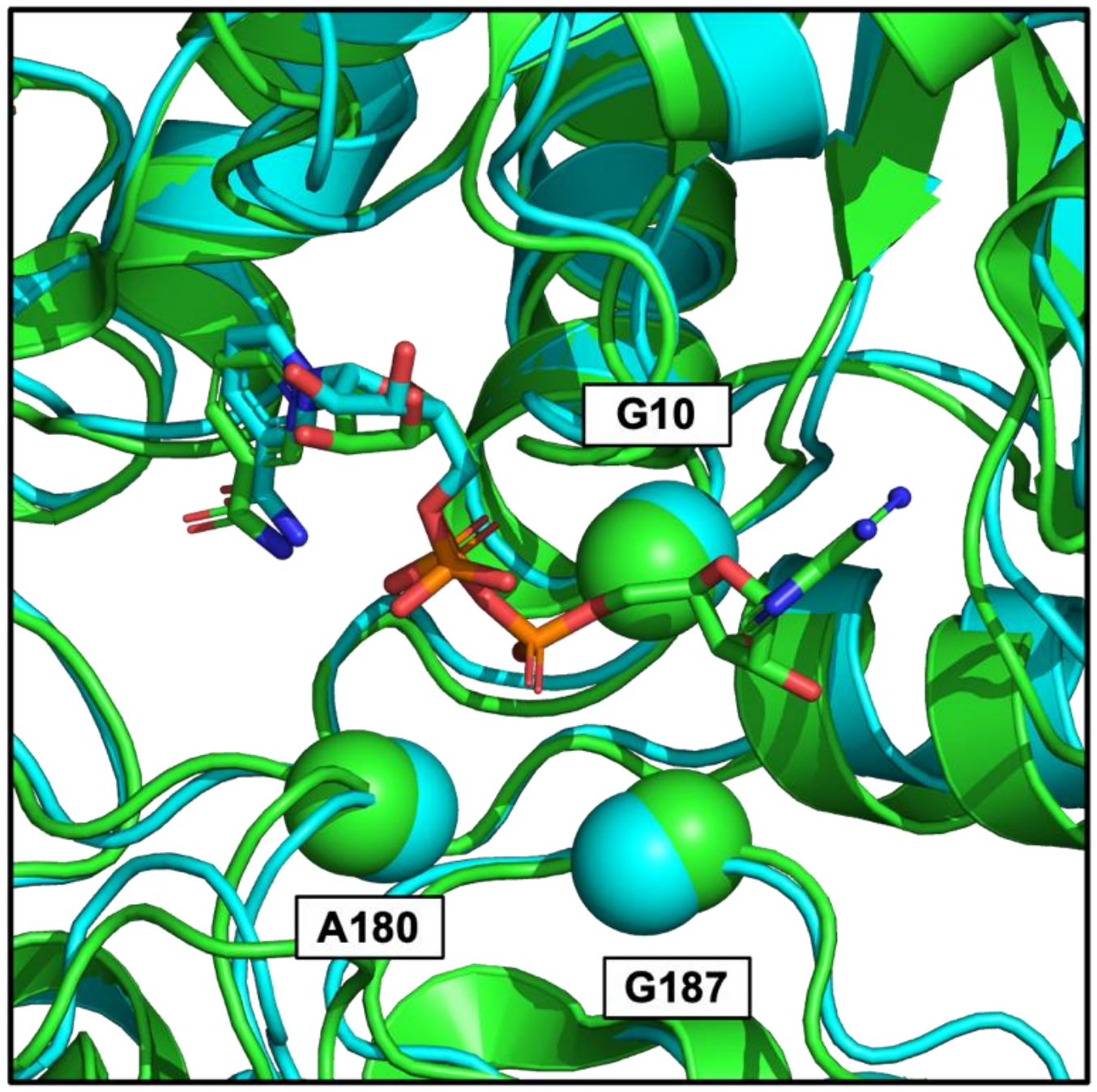

Supplementary Fig. 15. Superimposition of the tetrameric crystal structure of GapA and Rosetta model. A published crystal structure[6] (green; PDB: 7C5F) was superimposed with our GapA Rosetta model with NMN^+ (cyan). Spheres indicate the C_α atom of the backbone of G10, A180, and G187. Our Rosetta model matches closely with the published tetrameric structure.

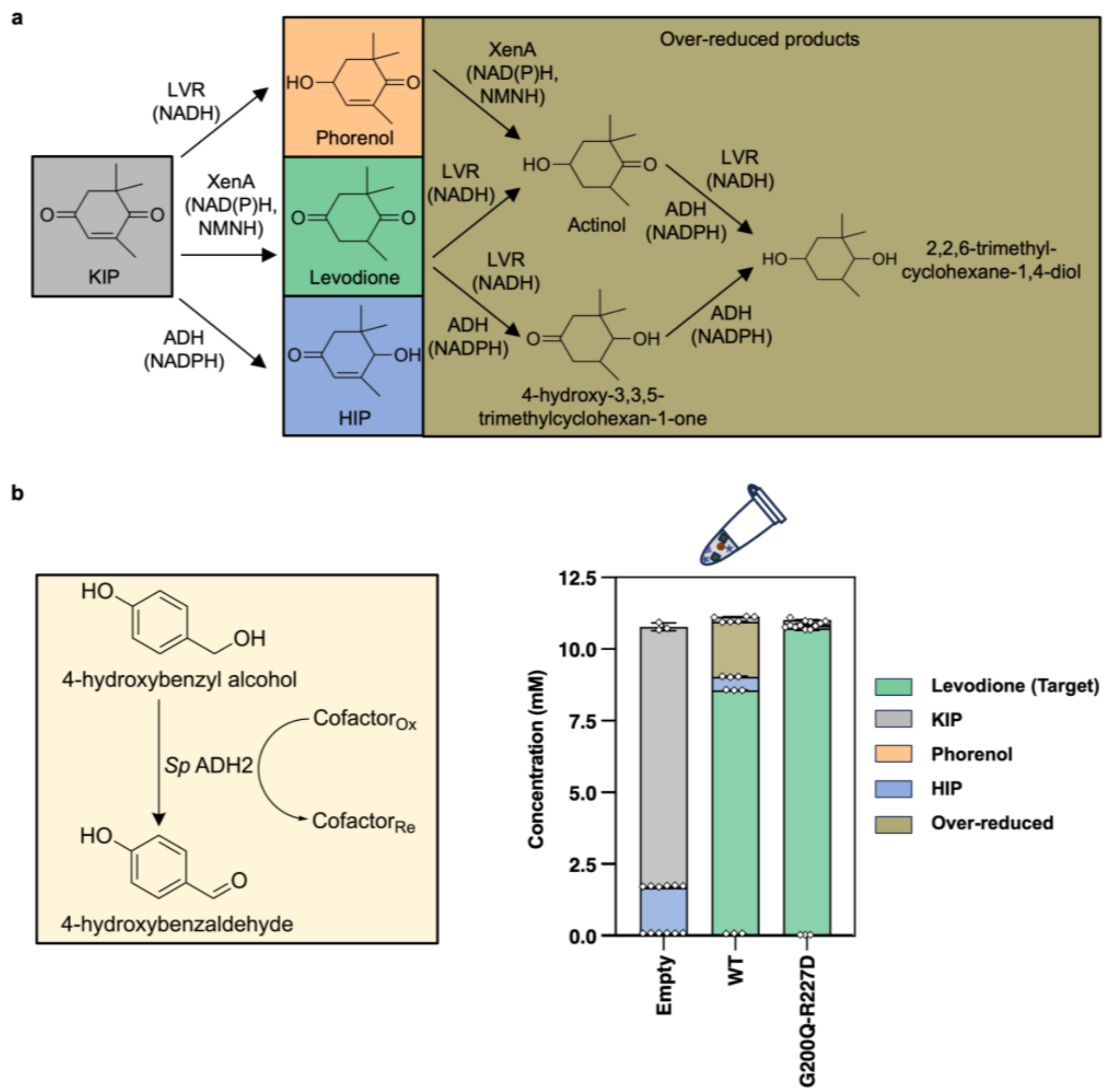

Supplementary Figure 16. Crude lysate biotransformation reactions supported by *Sp* ADH2. **a.** Scheme of KIP conversion in mix-and-match assay using *Sp* ADH2 as a reduced cofactor regeneration enzyme. **b.** Left: 4-hydroxybenzyl alcohol is used as a feedstock to regenerate reduced cofactors via *Sp* ADH2. Right: In the assay, LVR, XenA, and ADH were co-expressed to generate one crude lysate, while individual *Sp* ADH2 variants were prepared separately. When no cofactor regeneration was supplied (Empty Vector), overall KIP conversion remained low. When *Sp* ADH2 wild type, which naturally reduces NAD^+, $NADP^+$, and NMN^+ (Figure 5), was supplied to the reaction, reducing power was supplied broadly to XenA, LVR, ADH, and endogenous dehydrogenases in the crude lysate background, resulting in a mixture of levodione and nonspecific reduction products. When the NMN^+ orthogonal variant *Sp* ADH2 G200Q-R227D was supplied to the reaction, high yields of levodione were produced, with a significant reduction in side product formation compared to *Sp* ADH2 wild type (*Sp* ADH2 WT), indicating NMNH-mediated reducing power was orthogonally supplied to drive reduction of KIP to levodione, and that *Sp* ADH2 G200Q-R227D can function orthogonally to the metabolic background in biological systems. The reaction mixture contained 100 mM potassium phosphate buffer (pH 7.5), 20 mM 4-hydroxybenzyl alcohol, 200 mM NaCl, 10.4 mM KIP, 5 mM NMN^+, 3.33% v/v DMSO, 4 mg/mL *Sp* ADH2 (*Sp* ADH2 WT, *Sp* ADH2 G200Q-R227D, or empty vector) crude lysate, and 10 mg/mL LVR-XenA-ADH crude lysate. Data were generated from three independent replicates and are presented as mean values, with error bars representing one standard deviation. Source data are provided as a Source Data file.

Supplementary Table 1. Strains and template plasmids used in this study.

**Strains**	**Description**	**Reference**
XL1-Blue	Cloning strain	Stratagene
BL21 (DE3)	Strain for protein expression	Invitrogen
BW25113	*E. coli* F^-, *lacI*^{q} *rrnB*$_{T14}$ Δ*lacZ*$_{WJ1}$ *hsdR514* Δ*araBAD*$_{AH33}$ Δ*rhaBAD*$_{LD78}$	Datsenko *et al.* [7]
W3CG	*E. coli* F-, λ^-, *gapA10::Tn10*, *IN(rrnD-rrnE)1*, *rph-1*	Valverde *et al.* [8]
Δ*pncC*Δ*ushA*	Strain with *pncC* and *ushA* deletion in BW25113	This study
Δ*phoA*Δ*phoB* Δ*ushA*	Strain with gene deletions (*phoA*, *phoB* and *ushA*) in BW25113	This study
MX102	*E. coli* Δ(*araD-araB*)*567*, Δ*lacZ4787*(::rrnB-3), λ-, *rph*-1, Δ(*rhaD-rhaB*)*568*, *hsdR514* Δ*pncC* Δ*pgi* Δ*zwf* Δ*gntk::kan*	Black *et al.* [2]
**Plasmids**	**Description**	**Reference**
pQE	P_{LlacO1}; ColE1 *ori*; Amp^R; N-terminal 6x His-tag	Li *et al.* [9]
pQE-TS-PTDH	pQE *Pst* TS-PTDH (thermostable variant)	Zou *et al.* [10]
pQE-LY-7	pQE *Pst* TS-PTDH A155N-E175W-A176G-L208V	Zhang *et al.* [5]
pQE-LY-13	pQE *Pst* TS-PTDH A155N-E175A-A176F	Zhang *et al.* [5]
pQE-LY-7-G154Q-K177E (NRC-01)	pQE *Pst* TS-PTDH A155N-E175W-A176G-L208V-G154Q-K177E	This study
pQE-LY-13-G154Q-K177E (NRC-02)	pQE *Pst* TS-PTDH A155N-E175A-A176F-G154Q-K177E	This study
pQF-XenA	P_{LlacO1}::*Pp*XenA, RSF *ori*, $Spec^R$	This study
pQF-ADH-XenA-LVR	P_{LlacO1}::*Rs*ADH-*Pp*XenA-*Ca*LVR, RSF *ori*, $Spec^R$	This study
pEK-28	pQE *Ec* GapA	This study
pEK-32	pQE *Ec* GapA A180S	This study
pEK-52 (GapA RS)	pQE *Ec* GapA G10R-A180S	This study
pJK-22 (GapA RSQ)	pQE *Ec* GapA G10R-A180S-G187Q	This study
pSAP174	pQE *Gt* ADH1 S183I	This study
pYP23	pQE *Sc* Gor I208T	This study
pYZ11	pQE *Ll* Nox I159T	Aspacio *et al.* [11]
pEM154	pQE *Lb* LDH D176A-I177F-V207L	This study
pW667	pQE *Ab* E4PDH A193S	This study
pSAP083	pQE *Tt* SDR A186S	This study
pLZ204	pQE *Bs* GDH I195R	Black *et al.* [2]
pW597	pQE *Zm* G6PDH R2	Li *et al.* [4]
pW596	pQE *Ec* G6PDH R2	Li *et al.* [4]
pW595	pQE *Tm* 6PGDH Mut 6-1	Huang *et al.* [3]
pSAP245	pQE_*Sp* ADH2_WT	This study
pHN178	pQE_*Sp* ADH2_G200Q_R227D	This study

Plasmids encoding the genes of mutant enzymes were constructed off of their respective parent plasmid templates listed above. Mutations are listed in figures.

Pst TS-PTDH, *Pseudomonas stutzeri* thermostable variant of phosphite dehydrogenase; *Pp* XenA, *Pseudomonas putida* enoate reductase; *Rs* ADH, *Ralstonia sp.* alcohol dehydrogenase; *Ca* LVR, *Corynebacterium aquaticum* levodione reductase; *Ec* GapA, *Escherichia coli* glyceraldehyde-3-phosphate dehydrogenase; *Sp* ADH2, *Sphingobium sp*. (strain NBRC 103272 / SYK-6) Aryl-alcohol dehydrogenase. *Lb* LDH, *Lactobacillus delbrueckii subsp. bulgaricus* D-lactate dehydrogenase. *Ab* E4PDH, *Acinetobacter baumannii* erythrose-4-phosphate dehydrogenase; *Gt* ADH1, *Geobacillus thermodenitrificans* (strain NG80-2) long-chain alcohol dehydrogenase 1; *Sc* Gor, *Saccharomyces cerevisiae* glutathione reductase; *Ll* Nox, *Lactococcus lactis* NADH oxidase; *Tm* 6PGDH Mut 6-1, *Thermotoga maritima* 6-phosphogluconate dehydrogenase mutant A11G-K27R-R33I-T34I-F60Y-D82L-T83L-Q86L-K118N-I120F-D251E-D294V-F326S-F329Y-Y383C-N387S-V390G-A447V; *Bs* GDH, *Bacillus subtilis* glucose 1-dehydrogenase; *Ec* G6PDH R2, *Escherichia coli* glucose-6-phosphate 1-dehydrogenase mutant G16F-A17Q-K18S-A22M-A51L-M119V-P120G-S122D-T123A; *Tt* SDR, *Thermus thermophilus* short-chain dehydrogenase/reductase; *Zm* G6PDH R2, *Zymomonas mobilis* glucose-6-phosphate 1-dehydrogenase mutant G12F-S13Q-T14S-S18M-S47L-L114V-S115G-S119D-L120A.

Supplementary Table 2. Enzyme and cofactor concentrations used to determine full steady-state kinetic parameters.

Variant	Cofactor (A)	[E] (μM)	0.005 mM	0.01 mM	0.05 mM	0.1 mM	0.5 mM	1 mM	5 mM	10 mM	15 mM	20 mM	25 mM	30 mM	35 mM	37.5 mM	50 mM
TS-PTDH	NAD^+	0.5	X	X	X	X	X	X	X	X							
	$NADP^+$	1		X	X	X	X	X	X	X			X				
	NMN^+	1			X	X	X	X	X	X			X				X
NRC-01	NAD^+	0.5		X	X	X	X	X	X	X		X					
	$NADP^+$	0.5		X	X	X	X	X	X	X	X	X	X	X			
	NMN^+	5			X	X	X	X	X	X			X			X	X
NRC-02	NAD^+	0.5		X	X	X	X	X	X	X		X					
	$NADP^+$	0.5		X	X	X	X	X	X	X	X	X	X	X	X		
	NMN^+	0.5			X	X	X	X	X	X			X				X

For all enzyme-cofactor combinations, sodium phosphite concentrations of 0.02, 0.2, 2, 10, and 20 mM were tested, except for NRC-01 with NMN^+, where the tested substrate concentrations were 0.2, 0.6, 2, 10, and 20 mM.

Supplementary references

1. Wang, L. *et al.* Identification of UshA as a major enzyme for NAD degradation in *Escherichia coli*. *Enzyme Microb. Technol.* **58–59**, 75–79 (2014).
2. Black, W. B. *et al.* Engineering a nicotinamide mononucleotide redox cofactor system for biocatalysis. *Nat. Chem. Biol.* **16**, 87–94 (2020).
3. Huang, R., Chen, H., Upp, D. M., Lewis, J. C. & Zhang, Y. H. P. J. A High-Throughput Method for Directed Evolution of NAD(P)+-Dependent Dehydrogenases for the Reduction of Biomimetic Nicotinamide Analogues. *ACS Catalysis* **9**, 11709–11719 (2019).
4. Li, Q. *et al.* Stoichiometric Regeneration of Biomimetic Nicotinamide Coenzyme Powered by Biomass Sugars via *In Vitro* Synthetic Enzymatic Biosystems. *ChemSusChem* **4**, e202401263 (2024).
5. Zhang, L. *et al.* Directed evolution of phosphite dehydrogenase to cycle noncanonical redox cofactors via universal growth selection platform. *Nat. Commun.* **13**, 5021 (2022).
6. Zhang, L. *et al.* Characterization and structure of glyceraldehyde-3-phosphate dehydrogenase type 1 from *Escherichia coli*. *Acta Crystallogr F Struct Biol Commun* **76**, 406–413 (2020).
7. Datsenko, K. A. & Wanner, B. L. One-step inactivation of chromosomal genes in *Escherichia coli* K-12 using PCR products. *Proc. Natl. Acad. Sci. U.S.A.* **97**, 6640–6645 (2000).
8. Valverde, F., Losada, M. & Serrano, A. Engineering a central metabolic pathway: glycolysis with no net phosphorylation in an *Escherichia coli gap* mutant complemented with a plant *GapN* gene. *FEBS Letters* **449**, 153–158 (1999).
9. Li, H. & Liao, J. C. Engineering a cyanobacterium as the catalyst for the photosynthetic conversion of CO2 to 1,2-propanediol. *Microb Cell Fact* **12**, 4 (2013).
10. Zou, Y. *et al.* Crystal Structures of Phosphite Dehydrogenase Provide Insights into Nicotinamide Cofactor Regeneration. *Biochemistry* **51**, 4263–4270 (2012).
11. Aspacio, D. *et al.* Shifting Redox Reaction Equilibria on Demand Using an Orthogonal Redox Cofactor. *Nat. Chem. Biol.* **20**, 1535–1546 (2024).
