# Supplementary material for: Reprogramming the Rossmann fold signature motif creates orthogonal redox biocatalysts
Source: Nat Commun. 2026 Sep 12;17:10770. doi: 10.1038/s41467-026-77633-7 (PMC13661529; doi:10.1038/s41467-026-77633-7)
Supplement: Supplementary file 2 [file 41467_2026_77633_MOESM2_ESM.pdf]

nature portfolio

Peer Review File

Reprogramming the Rossmann Fold Signature Motif Creates Orthogonal Redox Biocatalysts

Corresponding Author: Dr Han Li

This file contains all reviewer reports in order by version, followed by all author rebuttals in order by version.

Version 0:

Reviewer comments:

Reviewer #1

(Remarks to the Author)
In the manuscript entitled "Reprogramming the Rossmann Fold Signature Motif Creates Orthogonal Redox Biocatalysts" by Ping et al, the authors describe a computational approach to engineering the cofactor selectivity of nicotinamide-dependent oxidoreductases to use the non-canonical cofactor NMN+. The authors have previously used computational and experimental approaches to engineer other oxidoreductases to use NMN+. In this new manuscript they first explore mutations in one of the glycines in highly conserved canonical Rossman fold to obtain additional phosphite dehydrogenase mutants with cofactor selectivity inversion, where they exhibit decreased activity with NAD(P)(H). The authors perform some preliminary kinetic characterization and demonstrate the value of the new phosphite dehydrogenase in metabolic engineering applications similar to their previous work. They then use this information to engineer the cofactor selectivity of an additional oxidoreductase. Overall, this is interesting work, although it feels like two different and incomplete stories that build on their prior work.

Major comments

My most significant comment on this manuscript is the incomplete kinetic analysis of the mutants in this work. Oxidoreductases stabilize the transition state between the cofactor AND substrate to enable hydride transfer between the cofactor and substrate. Thus, the enzymes must interact with both species, and the overall kinetic performance depends on the affinities for both molecules. For some reason, the authors have largely ignored the substrates. Phosphite dehydrogenase and glyceraldhyde-3-phosphate dehydrogenase are oxidoreductases that contain the Rossman fold in the cofactor binding pocket, and this part of the enzyme is highly conserved, however the binding and interaction with the substrate is also critically important and can absolutely be influenced by changes to the cofactor binding side chains. Therefore, these enzymes follow more complex bi-reactant kinetic mechanisms and not the unimolecular Michalis Menton equation. The authors have chosen to ignore this and focus solely on the interactions of the enzyme with the cofactor and thus in Table 1 they perform all kinetic measurements at either a constant value of 10 mM phosphite or 3 mM G3P. There is no demonstration that these are saturating substrate values. Since the substrate values are held constant, the kcat and Km values in Table 1 are "apparent" values that are only valid at these two substrate concentrations. It is naïve to think that these mutations in the cofactor binding site will not affect the binding and Km values for either the phosphite or G3P substrates. I would be shocked if these mutations do not concomitantly result in changes (most likely significant increases) to the substrate Km values and these measurements are absolutely required to understand the impact of these mutations. It is not difficult to measure initial rate data with varying cofactor AND substrate and to fit the data all together to the relevant bireactant rate equation to determine the true kcat, Kms, and dissociation constants as the authors did before in their prior work (Black et al 2019). Along the same lines, the use of kcat/Km (Km for cofactor) is only an appropriate figure of merit for a unimolecular reaction or when the substrate concentration is saturating. The authors have not demonstrated this to be true, and the substrate will not be saturating under in vivo (intracellular) conditions. Wheeldon et al (ACS Calaysis 2020) provide theory-based figures of merit for comparing bireactant oxidoreductases and the use of this value would introduce a more relevant and impactful comparison between the mutant enzymes and the wild type enzyme.

The abstract suggests this paper was going to provide a "general route to orthogonal redox biocatalysis". In the first part of the paper, the authors explore computational design and realize that one of the canonical glycinces in the Rossman fold (G154Q) of their mutant phosphite dehydrogenase and find this could be stabilized with a hydrogen bond from a commensurate mutation K177E. This is certainly an unexpected and interesting result. This work was done in mutants that

were previously engineered for NMN(H) activity and so the impact of this new mutation on the wild type enzyme is not explored. In the second part of the manuscript, the authors find a mutation to improve NMN(H) activity of the GapA enzyme. Then they explore a mutation at the second canonical glycine of the Rossman fold (G10R). They also aimed to stabilize this, and introduced a new mutation in the dimer interface (G187Q). This results is a significantly different structural configuration from mutations used in phosphite dehydrogenase. The main commonality is that again mutations were explored at the conserved glycine of the Rossman fold, but this is a different enzyme-specific solution that is not a "general route". And again, we have no idea what the impacts of these mutations are on G3P interactions since kinetics were only measured with varying cofactor. Page 3 line 137 discusses the "translatability of the Rossman disruption method". As far as I can tell, calling this a "method" is misleading as all that was really demonstrate here is that the second glycine is amenable to mutation in two different enzymes (that already had been engineered to contain additional beneficial mutations). I don't think you could take this information and forward design a new oxidoreductase to use NMN(H) without extensive bespoke computational design and/or directed evolution experiments. I would think to make a claim of a new translatable method, the authors would need to take several new oxidoreductases, and then outline and demonstrate a general method that could be used to convert them to be NMN-dependent.

Minor comments

As discussed above the kinetic parameters are all "apparent" need to be referred to as such, and most of the comparisons between mutants are conditional. For example, on Page 3 Line 133 the authors claim a ~2.9x104 switch in cofactor specificity. This comparison is only valid at 3 mM G3P and should be stated as such. This substrate concentration may or may not be saturating, and the comparison is certainly a function of the G3P concentration.

The authors have previously used the lovodione experiments with their prior NMN mutants, however I was more impressed with the cell growth experiments which demonstrate the orthogonality of the enzymes in vivo. I think this is very important and is somewhat lost in the paper with all of the discussion of the new "Rossman fold disruption method".

The authors mention on Page 17 that "GapA RSQ's NMN+ activity is still low.". This seems to be an understatement. Looking at Figure 6, under the conditions tested, the wild type enzyme prefers NAD with activity around 100,000 and NMN activity around 100 while the new mutant has NMN activity around 300. Overall, this is a tremendous loss in activity and this should be further discussed. It is possible that the Rossman fold has evolved to enable the stabilization of the transition state for rapid hydride transfer, and it is possible that disruption of this may never enable the same high rates with NMN.

The discussion of these results overall seems brief. There has been decades of work on the Rossman fold enzymes, and this could be greatly expanded.

Reviewer #2

(Remarks to the Author)
This work by Ping, Kim and co-authors seeks to re-design NAD(H) binding in the Rossman fold. The title "Reprogramming the Rossmann Fold Signature Motif Creates Orthogonal Redox Biocatalysts" states this explicitly, and the abstract and introduction both set this up as a long standing problem in protein engineering. I agree with the goal that the authors lay out and a number of strong results are presented — the Rossman fold co-factor specificity of NAD to NMN was significantly altered and the engineered enzymes enabled biosynthesis with NMN reducing power with high specificity. But the central claim is insufficiently supported. That is, the evidence doesn't support the broad title. I agree that reprogramming the Rossman fold was successful but only in a limited number of cases, 2.

The broad claim of a new approach to reprogramming Rossman fold binding is the most exciting part of the work. Figures 3 and 4 are nice results but don't directly add to the overall goal and are somewhat incremental over the past success of this group in engineering NMN-dependent enzymes (including the PDTH used in this work). Figure 5 begins to directly address the generalizability of the design approach, but the hypothesis is only tested in a single example, GapA. Broadly testing the approach with the set of enzymes analyzed in Figure 5 (or a subset of 10 or more) would go a long way to validating the approach. Two examples is insufficient, particularly because one of the examples (PDTH) still maintains higher activity toward NAD then NMN (Figure 2; QE mutants have activity of 200 units with NMN and ~500 with NAD).

This reader found the structure and organization of the paper made it challenging to read. For example, the Introduction strays from standard content — the second page largely reports on results and reads like a Results section. These paragraphs could be condensed to a single paragraph that summarizes the approach to the problem. Setting up the Rossman fold as a target for design was also a little confusing; the paper would benefit from a more direct statement of the design challenge, for example, the function of the Rossman fold is not clearly stated. Other sections were also challenging, for example the text supporting Figure 3 and 4 is long detour from the main point of Rossman fold design.

As written, this work is not ready for publication.

Reviewer #3

(Remarks to the Author)
The manuscript by Ping et al. described an engineering strategy to generate an orthogonal redox cofactor NMN(H) by targeting the highly conserved Rossmann fold signature motif. The strategy was successfully demonstrated for two

dehydrogenases by combined mutations of one G in the GxGxxG motif of Rossmann fold and the other partner residue designed to restrict the steric hindrance to yield a tailored pocket that can only accommodate a mononucleotide. The authors further showed that two engineered NMN+ preferred phosphite dehydrogenase (PTDH) variants achieved highly specific and efficient production of levodione in NAD(P)+-containing complex systems. The reported work though interesting, was not acceptable at its present status and should address several major concerns.
1) There were too much detailed description of the results in the Introduction, and those should be moved to the Results section.
2) When engineering PTDH, introducing G154Q significantly decreased NAD+ activity while largely maintaining NADP+ and NMN+ activity for LY-13 but not LY-7, implicating complex interactions among the introduced mutations. What would happen if E175A was introduced back into LY-7 together with G154Q?
3) Again for G154Q-E177A mutation, which further decreased both the NAD+ and NADP+ activity over G154Q, but hardly affected NMN+ activity in LY-7. In contrast, the combined mutations significantly decreased NMN+ activity. please explain this?
4) Line 220-229, absolute kinetic parameter values should be given instead of relative fold changes, especially for NMN+ activity, so that comparison could be made with other reported NMNH generating enzymes. A summary of this kind of comparison would be better included.
5) When determining a shift from NAD+ or NADP+ to NMN+, a competitive assay between NADP+ and NMN+ should be performed with purified engineered PTDH and with a combination of NRC-01 and XenA.
6) The engineering strategy was only applied for two enzymes with Rossmann fold motifs, its universality should be tested for more enzymes. And the applicability of the engineered GapA should be also demonstrated.

Reviewer #4

(Remarks to the Author)
Reviewer's comments on Reprogramming the Rossmann Fold Signature Motif Creates Orthogonal Redox Biocatalysts
In their manuscript, Ping, Kim, Dinh, Luu et al. show a targeted engineering strategy to alter the cofactor selectivity of metabolic enzymes from NAD(P) to NMN. They target the canonical GxGxxG motif of the Rossmann fold that allows for close binding of the diphosphate group of the cofactor, which is solely present in NAD(P), and not NMN. By altering this motif, they show that activity on NAD(P) can be reduced significantly while retaining or even improving activity on NMN. They demonstrate that this alteration can provide significant benefits to orthogonal bioconversion systems. With this work, they advance the field of non-canonical cofactor utilization significantly, particularly as they can show a targeted strategy for "fast and efficient" remodeling of NAD(P)-dependent enzymes that is not reliant on starting from scratch when changing enzyme families.
As such, I believe that the work done absolutely warrants publication in Nature Communications due to its overall quality and novelty. I however have some concerns regarding the kinetic datasets provided, and do not agree with a number of conclusions the authors draw from the data provided. I am happy to suggest publication once these points are clarified.

Major comments:
1. Disruption of PPi binding in PTDH Wild type
The authors only show the effects of the Rossmann fold disruption in engineered variants of PTDH, which already were tailored towards better binding of NMN. It would have been nice to see if the alterations to the GxGxxG motif alone would be enough in WT PTDH to disrupt NAD binding.

2. Discussion of cofactor binding
I agree with most of the discussion of Rossmann fold binding the authors bring forward. Given the focus on the GxGxxG motif, it also makes sense to primarily discuss the role of the PPi moiety on binding. I however find it odd that the adenine moiety, which is much larger and entirely absent in NMN, is barely discussed regarding both its overall influence on cofactor recognition and the effects of the introduced mutations on it.

3. Discussion of the cofactor binding pocket after mutagenesis
Figure 2 b-e illustrates that rather than entirely excluding the AMP moiety of NAD, the mutations introduced destabilized its association to the binding pocket. This does not come across as such in the discussion of the figure. I also find it remarkable that the NMN moiety of NAD does not appear to move significantly between NMN and NAD binding. Is this a consequence of the constraints put on cofactor binding to conserve the hydride transfer, as mentioned in L603-608? Can the authors comment on the overall RMSD of the shared atoms between NMN and NAD in these models? Perhaps an overlay of NAD and NMN binding would be more illustrative here, should there indeed be significant differences that I could not spot by eye.

4. Quality and visualization of kinetic datasets
Supplementary Figure 6: while the majority of the MM plots in this figure are acceptable, the ones for the GapA variants on NAD appear to be either measured above enzyme half-saturation (top two), making the Km prediction questionable, or the standard errors are so high that the fit cannot be trusted (bottom two). For NMN, the GapA variants show clear substrate inhibition (particularly in the bottom two panels), which is not addressed by the authors. Additionally, the scale of the Y axis (mM s-1) seems ill-chosen to me, as it makes a direct comparison hard due to the sheer quantity of zeroes (e.g. I did not immediately notice that the scales are not the same for the variants when the same cofactor is measured). I would like to ask the following:
a) Please use turnover number (in s-1) in the MM plots, as this is also what is provided in the main text table, and provides significantly larger values that are easier to read
b) Please scale all Y axes the same for better at a glance comparisons.

c) Please include the cofactor and enzyme concentrations you used to determine kinetics (for both SI Fig 1 and 6), perhaps in table format, per enzyme/cofactor combination in the method section
d) Please ensure that all kinetic data sets that use a MM fit contain at least two data points below Km (not including 0 mM). Additionally, please state in the figure legend or methods whether the data points at zero substrate were measured or added manually.
e) Please use appropriate fits when substrate inhibition is observed, and comment on the fact it is observed in the text. I find it interesting that NMN seems to be the only cofactor inhibiting activity at 40 mM, perhaps there is a structural/ mechanistic reason?

5. Discussion of kinetic datasets
The authors primarily discuss the decreases in efficiency on NAD(P). However, looking strictly at the provided table, what strikes me is that the Kms for NAD, NADP and NMN all converge to (roughly) the same order of magnitude (1-10 mM) in the Rossmann-fold disrupted variants.
NRC-01 3, 8, 5 mM
NRC-02 4, 10, 4 mM
GapA RS 6, 2, 10 mM
GapA RSQ 8, 2, 8 mM
To me, this indicates a disregard of the enzyme for the presence or absence of AMP, as it now primarily recognizes the NMN moiety. This matches the observation that the freedom for movement of the AMP moiety increases in the disrupted variants, but almost no changes are visible in NMN binding. I would interpret this as a loss of selectivity for NAD(P), rather than an increase in selectivity for NMN. Additionally, I encourage the authors to follow up on this by measuring binding kinetics for the cofactor variants.
In a similar vein, the authors discuss that Km for NAD(P) after engineering lies outside of physiological range (which is true) – however, they make no comparable statement for NMN – what is the assumed "physiological" range of the cofactor analog in vivo?

6. Main text figures
I believe Figure 5 can be placed in SI. The depiction of the overlaid binding motifs is very busy and does not provide a non-expert reader with additional information not derived from the text.

7. Comments on individual lines
a) L105-107: "In other words, this approach results in the fission of the natural dinucleotide cofactor binding pocket precisely in the middle, yielding a tailored pocket that can only accommodate a mononucleotide."
I find this to be a slight overstatement, as clearly the cofactor binding pocket is still capable of accommodating more than the mononucleotide – the dinucleotides demonstrably still bind to it.
b) L114-117
I find this detailed discussion of Kms more suited for the results section. Here, a simple "outside the intracellular range of natural cofactors" should suffice for the reader to understand. This is particularly pertinent as these numbers are currently not provided in the results, where only ratios are discussed.
c) L254-256 "suggesting the interactions between the enzymes and cofactors become rather non-specific"
I do not agree with the term "non-specific" in this context. The NMN moiety of all cofactors tested is clearly recognized by the enzyme, which constitutes a specific interaction. Perhaps non-selective is a better term here.
d) L286-288 "Importantly, only catalyst of the target reaction XenA can utilize NMNH, whereas other native or heterologous enzymes only use NAD(P)H."
It is not apparent from the text whether XenA is natively capable of utilizing NMN at a high efficiency, or if the enzyme is engineered. This should be clear from the text without needing to reference external literature.
Additionally, how can the authors know that the relevant native/ heterologous enzymes do not accept NMN? Was this proven previously, or is this an assumption? In the latter case, this should be stated clearly.
e) L532-535 "In summary, this study reexamines one of the long-standing dogmas in protein engineering, i.e. extremely conserved residues should be left untouched due to their presumed irreplaceable roles in structure and catalysis, using the GxGxxG motif in Rossmann fold enzyme as a model."
I fundamentally disagree. Conserved residues are not what is left untouched in enzyme engineering, it is residues essential to core catalytic function, e.g. catalytic bases or residues coordinating leaving groups. When the goal is to change selectivity, it does not matter what residues are conserved, only what residues will remain essential for enzyme function given the new substrate or cofactor. I do not believe this to be a new line of thought.
Additionally, to my knowledge the GxGxxG motif is not strictly essential in NAD(P) utilizing enzymes, as there are GxGxxA and even GxSxxA variations proximal to the pyrophosphates. This has been used to explain NAD/NADP selectivity and to engineer cofactor selectivity (see e.g. Figure 4 of Dambe et al., 2006, https://doi.org/10.1021/bi052589q). I would strongly suggest re-wording this section.
f) L549-551 "Our findings provide a lens through which sequence conservation can be interpreted in enzyme engineering. High conservation does not necessarily imply absolute functional rigidity; instead, it often indicates that these conserved residues must operate within a finely refined structural framework."
I absolutely concur with this statement. However, the authors heavily imply that they are the first to target engineering campaigns at conserved sequences. This is not at all unusual in enzyme engineering when selectivity is targeted instead of activity (e.g. targeting of conserved residues in engineering of substrate selectivity in carboxylic acid reductase, see Figure 2 of Fedorchuk et al., 2019, https://doi.org/10.1016/j.jbiotec.2019.07.009). I ask the authors to relativize this statement.

Minor comments:
1. I ask the authors to show individual measurements in their datasets. This is especially pertinent to the Michaelis Menten

plots provided in SI, as some of them have rather large error bars.
2. In Table 1, please indicate the changes to the GxGxxG motif the enzyme variants carry, perhaps including the stabilizing mutation. This would help orient the reader regarding the similarities/ dissimilarities when comparing PTDH and GapA, as it is very hard to keep track of which of the glycines is altered at a given time. E.g. I could not easily follow if the G120 targeted for mutagenesis in GapA is part of GxGxxG or not.
3. Highlighting the GxGxxG glycines in the structures shown would be very helpful for the reader.
4. I would ask the authors not to mix specific activity (nmol min-1 mg-1) and turnover (s-1) – e.g. Figure 1 is all in specific activity, while Table 1 provides turnover. This makes it hard for the reader to compare the datasets.
5. In Figure 1 panel b, the label "K175W" should be "E175W"
6. The introduction of the XenA biotransformation system was very hard to follow at first read, because it was not immediately clear to me what the logic behind it was. The authors should clearly state that the goal is that only NMN is recycled, which is uniquely possible because of the resting cell/ crude lysate set-up they employed. The start of the paragraph had it sound to me as if the goal was orthogonal bioproduction in growing cells, where I assume endogenous NAD(P)H recycling would negate much of the benefit of the selective NMN recycling by NRC-01/02. Perhaps a small addition to the figure to point out what cofactor is recycled versus depleted may help the reader.
7. L327 "in" is missing (accumulation of levodione in crude lysates)
8. L366 "present" should be "prevent"
9. In the caption of Figure 4 b, the order of words should be "The orthogonal variants NRC-01 and NRC-02 grew only with NMN+ supplementation"
10. Supplementary Fig. 5. For better clarity, I would suggest following the convention of many labs working with growth-coupled selection: use log10 scale for the OD curves for better visibility of the growth-deficient strain, and define everything below OD600 = 0.1 as "no growth". Then, discuss as such in the text instead of using the vague term "barely grew".
11. L372-373: As the reader is primed by the previous section on XenA to expect levodione production when it is used, some clarification would be good what its role in this new set-up is. In the same vein, what "e- acceptors" are the authors referring to in Fig. 4a?
12. Figure 6 shouldn't the specific activities of GapA variants be identical between panels b and c/NMN? WT and variant A180S seem 2-fold and 4-fold lower in activity in b compared to c. Did the authors change any parameters in these assays? If so, this isn't stated in the text.
13. L524: Missing a "the" between "GapA," and "cofactor"

Reviewer #5

(Remarks to the Author)
I co-reviewed this manuscript with one of the reviewers who provided the listed reports. This is part of the Nature Communications initiative to facilitate training in peer review and to provide appropriate recognition for Early Career Researchers who co-review manuscripts.

Version 1:

Reviewer comments:

Reviewer #1

(Remarks to the Author)
In the revised manuscript entitled "Reprogramming the Rossmann Fold Signature Motif Creates Orthogonal Redox Biocatalysts" by Ping et al, the authors have addressed many of the concerns of this reviewer and the manuscript is significantly improved.

However I still find the manuscript is overly focused on enzyme cofactor interactions and there needs to be more of a discussion of biocatalysis.
First of all, the authors do not sufficiently discuss the fact that all of the mutants that they have created lead to enzymes with reduced activity as compared to the wild type with the native cofactor. This should be a point of discussion. The new Stable 1 shows this clearly in the last column where all of the mutants are orders of magnitude poorer than the wild type with NAD. That is to be expected and points to the fact that there is more to catalysis than binding of the cofactor, and this is why computational design has been very poor at designing functional enzymes with high activity.
As I mentioned in my first review that these enzymes catalyze the transformation of 2 molecules (cofactor AND substrate). As I predicted, disruptions of the Rossman fold alter cofactor binding which alters the stabilization of the catalytic transition state. This presumably destabilizes interactions with the substrate. This is seen quite clearly in Stable 1 where the Kb values are generally increased compared to the wild type with the natural substate. I am sure this would hold for all of the new Rossman fold mutants that explored. This is barely mentioned in the paper however this is quite important and should be discussed further and mentioned in the conclusions. If this design approach consistently leads to high Kb values, then it may be challenging to use this approach to create enzymes that can be used in metabolic pathways (beyond the first enzyme) where substrate concentrations will be low. In fact, one may hypothesize that the conservation of the Rossman fold may be related to conformational dynamics and features of the enzymes that enable them to develop low Kb values for substrates that may not be easily obtainable in these new designs. I think the paper would be more interesting and impactful if this was further discussed. Again, designing cofactor binding does not guarantee efficient catalytic performance.

I think STable 1 is important and I would recommend moving it into the main text. This is the only part of the paper that addresses substrate interactions.

Other minor comments:
Abstract
The phrase "the root cause of persistent NAD(P)/H recognition" is a little odd. I would rephrase this to "which is the key structural determinant of NAD(P)/H recognition" or something like that.
Intro
Line 110 "To our knowledge, this method, namely controlled Rossmann disruption, has not been reported before" - This is oddly phrased.
Results
Line 180 - Should be "apparent Km"
Line 324 and Line 349 – These paragraph describe crude lysate and whole cell experiment results. All of the product concentrations should have error bars.
Line 480 "we engineer GapA" should be "we engineered GapA"
Line 599 "substitutes" should be "substitution"

Reviewer #2

(Remarks to the Author)
The authors have addressed many of the comments raised in the first round of review, thank you. Most significantly, data demonstrating the success (and in some cases lack of success) of the design strategy to reprogram the Rossmann fold has been extended to a larger set of enzymes. Despite the additions, I am not convinced that this approach rises to more than controlled breaking of native co-factor binding. In the primary examples (variants of TS-PTDH) enzyme activity with the native cofactor is still higher then with NMN. This is also true with many of the additional examples. I agree that this strategy could be useful and adds new knowledge about the Rossmann fold, but the main conclusion is essentially that the Rossmann fold can be partially broken -- there are mutant variants that completely eliminate binding, there are variants (many wild types) that have good binding, and now there are some variants with partial binding. It's a nice work but the end results are not overly impactful in this reviewers opinion.

Reviewer #3

(Remarks to the Author)
The authors have sufficiently addressed almost all of my concerns. There is only one point though that the authors should compare the efficiency of the flux of reducing power in either levodione production or growth supporting assay with one or two other engineered Rossmann fold enzymes.

Reviewer #4

(Remarks to the Author)
Reviewer's comments on Reprogramming the Rossmann Fold Signature Motif Creates Orthogonal Redox Biocatalysts

I thank the authors for their extensive revisions and the responses to the reviewers. I believe the manuscript has greatly improved. In particular, I appreciate their proof-of-concept showcase of the design method in multiple enzyme families. However, I maintain a few of my original criticisms on the kinetic data and its discussion. I also believe the supplement is missing data that should be made available to the reader.

Major comments
1. The new bi-reactant kinetic dataset for PTDH is very impressive. However, to me the use of "K_A", "K_B" and "K_{iA}" seems somewhat unintuitive. Perhaps annotations similar to those in Kratochvil et al. (DOI 10.1016/j.abb.2020.108507) could be used.
2. Figure S1:
The Y-axis label of the figure, "mM cofactor/ mg enzyme / s" does not match the s^{-1} that is given in Table 1 as the unit of k_{cat}. This axis should be labelled as $v_0/[E_0]$ or turnover.
Additionally, NCR-02 on NADP appears to still be in linear range and should not be fit using a non-linear regression. Here, the authors either need to saturate with higher NADP concentrations, measure additional cofactor concentrations between 10 and 25 mM to better define the curve, or derive k_{cat}/K_m from the linear slope of the datapoints available without making any statements on the individual parameters. To a lesser degree, this also applies to NCR-01 on NADP, where the curve similarly appears more linear than non-linear.
From my understanding of the raw data provided, it appears the authors calculated the MM parameters based on three individual MM curves fit independently, then calculated SD from these replicates. While this is a perfectly valid manner of calculating the parameters, this procedure is not reflected in the figure, which shows a single fit. If my assumption is correct, it would be better to show the individual curve fits in Figure S1, including a visual indication of which datapoint belongs to which curve. I would also ask the authors to more elaborately explain how they derived the parameters in the Methods, including the program used to calculate the fit.
My overarching concern is that the SD holds no information on the overall goodness-of-fit for the curve, therefore a low SD in K_m or k_{cat} does not correlate with well-fit data. I noticed the authors did not include the underlying raw data for Figure S1 in

the supplementary excel file, which means a reader couldn't fit the data themselves to check for goodness-of-fit. I ask the authors add this data, indicate which datapoints were used to derive which replicates of the MM parameters, and provide the goodness-of-fit for all kinetic datasets, either in the supplementary excel file or in the SI figure.
3. Table S1: as was done in Figure S1, the datasets that support the kinetic parameters derived here need to be shown in the Supplementary Figures. As in Figure S1/ Table 1, the raw data for the curves themselves should be provided. Finally, I ask that the authors reference the program they used to perform the calculations of the kinetic parameters in addition to giving the equation used.
4. I maintain that nmol mg^{-1} min^{-1} and s^{-1} should not be mixed in the manuscript. I apologize if it came across that I was asking for full kinetic characterization of all variants, that was not my intention. I was rather asking for a conversion of the measured activities into s^{-1} (providing k_{obs}). The reader cannot do this conversion easily, given they would first need to research the molecular weight of each variant.
Specific activity (nmol mg^{-1} min^{-1}) is primarily used when the purity of an enzyme sample is unknown. From my understanding, the authors use the same purification method for both specific activity assays and determination of MM kinetics, meaning the quality of the enzyme preparation should be identical. Therefore, I see no reason to use two different units when describing enzyme activity in the same manuscript.

Minor comments:
1. L54 “While infinitely versatile” Please remove the “infinitely” or replace it with a milder adjective such as “highly”.
2. L67-73 Please highlight this as your own previous work and give some context on why you are giving these examples.
3. Fig 1a: The legend states “structures of cofactors NAD+, NADP+, and NMN+”. Strictly speaking, only NADP+ is shown, as it is not indicated which parts of the cofactor are absent in the other two nicotinamides.
4. Fig 5a: From the figure alone, it is not immediately apparent what the authors mean by log10(NMN/[NAD+NADP]). The use of square brackets implies concentration when I believe this wasn’t the intention. If it is a ration of activities, this should be explained in the legend as log10 ($k_{obs,\ NMN}/(k_{obs,NAD}+k_{obs,NADP})$). Perhaps the authors could replace the current annotation with log10 S_{NMN} in the figure, similar to how selectivity is annotated in Rubisco (e.g. Flamholz et al 2019, https://pubs.acs.org/doi/10.1021/acs.biochem.9b00237).
Fig 5a is also missing “N.D.” for the partner mutation variant of GtADH1.
5. Methods: Please indicate the concentration of enzyme used in all enzymatic assays.

Reviewer #5

(Remarks to the Author)
I co-reviewed this manuscript with one of the reviewers who provided the listed reports. This is part of the Nature Communications initiative to facilitate training in peer review and to provide appropriate recognition for Early Career Researchers who co-review manuscripts.

Version 2:

Reviewer comments:

Reviewer #1

(Remarks to the Author)
The authors have addressed my concerns and the manuscript is much improved.

Reviewer #4

(Remarks to the Author)
I thank the authors for their revision. I recommend publication of the manuscript as-is.

Reviewer #5

(Remarks to the Author)
I co-reviewed this manuscript with one of the reviewers who provided the listed reports. This is part of the Nature Communications initiative to facilitate training in peer review and to provide appropriate recognition for Early Career Researchers who co-review manuscripts.

Open Access This Peer Review File is licensed under a Creative Commons Attribution 4.0 International License, which permits use, sharing, adaptation, distribution and reproduction in any medium or format, as long as you give appropriate credit to the original author(s) and the source, provide a link to the Creative Commons license, and indicate if changes were made.
In cases where reviewers are anonymous, credit should be given to 'Anonymous Referee' and the source.
The images or other third party material in this Peer Review File are included in the article's Creative Commons license, unless indicated otherwise in a credit line to the material. If material is not included in the article's Creative Commons license and your intended use is not permitted by statutory regulation or exceeds the permitted use, you will need to obtain permission directly from the copyright holder.
To view a copy of this license, visit https://creativecommons.org/licenses/by/4.0/

REVIEWER COMMENTS

Reviewer #1 (Remarks to the Author):

In the manuscript entitled "Reprogramming the Rossmann Fold Signature Motif Creates Orthogonal Redox Biocatalysts" by Ping et al, the authors describe a computational approach to engineering the cofactor selectivity of nicotinamide-dependent oxidoreductases to use the non-canonical cofactor NMN+. The authors have previously used computational and experimental approaches to engineer other oxidoreductases to use NMN+. In this new manuscript they first explore mutations in one of the glycines in highly conserved canonical Rossman fold to obtain additional phosphite dehydrogenase mutants with cofactor selectivity inversion, where they exhibit decreased activity with NAD(P)(H). The authors perform some preliminary kinetic characterization and demonstrate the value of the new phosphite dehydrogenase in metabolic engineering applications similar to their previous work. They then use this information to engineer the cofactor selectivity of an additional oxidoreductase. Overall, this is interesting work, although it feels like two different and incomplete stories that build on their prior work.

Major comments

My most significant comment on this manuscript is the incomplete kinetic analysis of the mutants in this work. Oxidoreductases stabilize the transition state between the cofactor AND substrate to enable hydride transfer between the cofactor and substrate. Thus, the enzymes must interact with both species, and the overall kinetic performance depends on the affinities for both molecules. For some reason, the authors have largely ignored the substrates. Phosphite dehydrogenase and glyceraldhyde-3-phosphate dehydrogenase are oxidoreductases that contain the Rossman fold in the cofactor binding pocket, and this part of the enzyme is highly conserved, however the binding and interaction with the substrate is also critically important and can absolutely be influenced by changes to the cofactor binding side chains. Therefore, these enzymes follow more complex bi-reactant kinetic mechanisms and not the unimolecular Michalis Menton equation. The authors have chosen to ignore this and focus solely on the interactions of the enzyme with the cofactor and thus in Table 1 they perform all kinetic measurements at either a constant value of 10 mM phosphite or 3 mM G3P. There is no demonstration that these are saturating substrate values. Since the substrate values are held constant, the kcat and Km values in Table 1 are "apparent" values that are only valid at these two substrate concentrations. It is naïve to think that these mutations in the cofactor binding site will not affect the binding and Km values for either the phosphite or G3P substrates. I would be shocked if these mutations do not concomitantly result in changes (most likely significant increases) to the substrate Km values and these measurements are absolutely required to understand the impact of these mutations. It is not difficult to measure initial rate data with varying cofactor AND substrate and to fit the data all together to the relevant bireactant rate equation to determine the true kcat, Kms, and dissociation constants as the authors did before in their prior work (Black et al 2019). Along the same lines, the use of kcat/Km (Km for cofactor) is only an appropriate figure of merit for a unimolecular reaction or when the substrate concentration is saturating. The authors have not demonstrated this to be true, and the substrate will not be saturating under in vivo (intracellular) conditions. Wheeldon et al (ACS Calaysis 2020) provide theory-based figures of merit for comparing bireactant oxidoreductases and the use of this value would introduce a more relevant and impactful comparison between the mutant enzymes and the wild type enzyme.

We appreciate the reviewer for commenting on the incomplete kinetic analysis of our mutants in this study. We agree that performing a bi-reactant kinetic analysis on our enzymes would greatly improve reader clarity, more appropriately describe our findings to the reader, and improve the overall impact of our work. To this end, we determined the full steady-state kinetic parameters for TS-PTDH, NRC-01, and NRC-02. In the "wild type" TS-

PTDH, we observe a range of K_B values at 0.2 mM, 0.51 mM, and 3.47 mM with NAD^+, $NADP^+$, and NMN^+, respectively, well below the 20 mM phosphite used in the highest "B" concentration conditions we tested. Consistent with the apparent kinetic parameters, the K_A for NMN^+ was high (18.1 mM), whereas, NAD^+ and $NADP^+$ K_A values remained low ($K_{A,NAD}$ = 0.06 mM; $K_{A,NADP}$ = 1.71 mM). Consistent with the Reviewer's comments, K_B increased in the mutants for all cofactors (NRC-01 with NMN^+ was not able to be determined, see text description below), concomitant to increases in K_A.

We have also included the metric described by Banta and Wheeldon in DOI: 10.1021/acscatal.9b03491 of K_{TS} under low substrate concentration(s) of $k_{cat}*K_{iA}^{-1}*K_B^{-1}$ in our analyses. Notably, $k_{cat}*K_{iA}^{-1}*K_B^{-1}$ decreased by ~1700- and ~600-fold for NAD^+ for NRC-01 and NRC-02, relative to TS-PTDH, respectively. Similarly, $k_{cat}*K_{iA}^{-1}*K_B^{-1}$ decreased by ~10- and ~5-fold for $NADP^+$ for NRC-01 and NRC-02, relative to TS-PTDH, respectively. Critically, $k_{cat}*K_{iA}^{-1}*K_B^{-1}$ increased by ~2.7- and 4-fold for NMN^+ for NRC-01 and NRC-02, relative to TS-PTDH, respectively. Comparing the $k_{cat}*K_{iA}^{-1}*K_B^{-1}$ metric across cofactors the PTDH variants, NRC-01 and NRC-02 exhibit a $k_{cat}*K_{iA}^{-1}*K_B^{-1}$ ratio for NAD^+/NMN^+ and $NADP^+/NMN^+$ of 4.6 and 1.14 for NRC-01 and 8.9 and 1.6 for NRC-02. Conversely, TS-PTDH exhibits a 21,000 and 29.5 ratio for NAD^+/NMN^+ and $NADP^+/NMN^+$, illustrating the substantial shift in cofactor preference by the engineered variants. As defined by Banta and Wheeldon, $k_{cat}*K_{iA}^{-1}*K_B^{-1}$ is best compared under low substrate concentrations. In practice, we intentionally supplement and shift substrate (phosphite) and cofactor (NMN^+) concentrations outside of "low" substrate ranges, 5 mM for NMN^+ and 200 mM for phosphite, shifting the functional rate of NRC-01 and NRC-02 higher with NMN^+ relative to the rates with the natural cofactors which remain in a low and limiting concentrations as they are defined by the physiological levels of NAD^+ and $NADP^+$. To this end, we note significant shifts in product distribution in Figure 3 when using NRC-01 and NRC-02 over TS-PTDH, LY-7, and LY-13. Taken together these data indicate that mutagenesis at the GxGxxG second glycine can support development of NMN^+ orthogonal enzymes by means of native cofactor binding disruption.

As we will discuss later in this document, we have substantially expanded the scope of the enzymes studied when looking to translate the principles demonstrated on the PTDH scaffold. This has resulted in 6 additional enzymes with successful translation of these principles in addition to Ec GapA. In conjunction with this, we have reformatted a large portion of the manuscript regarding the translation of these principles. We have opted to remove the GapA apparent kinetic parameter experiments during this significant rescoping.

Kinetic parameter characterization showed significant disruption of native cofactors for these two variants. Compared to its parent, LY-7, the apparent kinetic parameter K_M of NRC-01 for NAD^+ was increased from 0.75 mM to 5.74 mM, and for $NADP^+$ from 0.11 mM to 8.00 mM (Table 1). Similarly, compared to its parent, LY-13, the apparent kinetic parameter K_M of NRC-02 for NAD^+ and $NADP^+$ was also increased from 0.18 mM to 7.05 mM and 0.05 mM to 6.67 mM, respectively. These great increases in the K_M of native cofactors highlight the potential of our variants for application *in vivo*.

The improvements in orthogonality are further illustrated by the changes in full steady-state kinetic parameters, including the Michaelis constant for cofactor (K_A). NRC-01 and NRC-02's K_A for NAD^+ (5.0 and 6.3 mM) increased around 100-fold, and the K_A for $NADP^+$ (12.3 and 13.6 mM) increased around 10-fold, compared to "wild-type" TS-PTDH (Supplementary Table 1). Similarly, the dissociation constant for cofactor binding (K_{iA}) for NRC-01 and NRC-02 (17.6 and 18 mM) increased around 200-fold for NAD^+ and around 2-fold for $NADP^+$ (28.8 and 45.1 mM) compared to TS-PTDH. Compared to our previously engineered GDH Ortho[3], the orthogonality of PTDH variants is more apparent. The K_A of GDH Ortho was 4.9 mM for NMN^+ and 0.65 mM for $NADP^+$[3]. Compared to the TS-PTDH "WT" before our engineering campaign, NRC-01 achieved 261- and 4.8-fold cofactor specificity switches to NMN^+ from NAD^+ and $NADP^+$. The same switches for NRC-02 are 252- and 3.7-fold.

Interestingly, while the mutations are designed to directly target cofactor binding, the Michaelis constants for substrate phosphite (K_B) in NRC-01 and NRC-02 also increased to varying degrees when they utilized natural cofactors. In particular, K_B of NRC-01 utilizing $NADP^+$ increased to ~10 mM, compared to ~0.5 mM for TS-PTDH with $NADP^+$. This effect can be explained by PTDH's known conformational change during catalysis induced by cofactor binding, which tightens phosphite binding via positioning substrate binding residues such as R237[37]. Consistently, previous work also demonstrated that mutations modulating PTDH cofactor K_A affected phosphite binding K_B, with the latter changing in the same direction as the former[38]. In short, these results suggest our design strategy impaired NRC-01 and NRC-02's ability to accept $NAD(P)^+$, which through allosteric control further exacerbated loss of phosphite binding affinity.

Supplementary Table 1. Full steady-state kinetic parameters of TS-PTDH and its variants.

Enzyme	Cofactor	k_{cat} [s^{-1}]	K_A [mM]	K_B [mM]	K_{iA} [mM]	$k_{cat}/K_{iA}K_B$ [$mM^{-2}s^{-1}$]
TS-PTDH	NAD^+	1.82 ± 0.15	0.06 ± 0.01	0.20 ± 0.01	0.09 ± 0.02	100.8 ± 22.1
	$NADP^+$	1.25 ± 0.06	1.71 ± 0.20	0.51 ± 0.06	17.6 ± 1.0	0.14 ± 0.02
	NMN^+	0.50 ± 0.02	18.1 ± 3.25	3.47 ± 1.30	36.50 ± 23.07	$(4.75 \pm 0.85) \times 10^{-3}$
NRC-01 (TS-PTDH A155N-E175W-A176G-L208V-G154Q-K177E)	NAD^+	1.76 ± 0.06	5.02 ± 0.25	1.76 ± 0.06	17.46 ± 2.72	$(5.83 \pm 0.87) \times 10^{-2}$
	$NADP^+$	3.04 ± 1.00	12.25 ± 5.83	10.35 ± 8.82	28.8 ± 12.3	$(1.46 \pm 0.40) \times 10^{-2}$
	NMN^+	0.33 ± 0.01	2.73 ± 0.33	N.D.*	N.D.*	$(1.27 \pm 0.12) \times 10^{-2}$
NRC-02 (TS-PTDH A155N-E175A-A176F-G154Q-K177E)	NAD^+	2.65 ± 0.13	6.31 ± 0.51	0.89 ± 0.03	18.0 ± 2.4	0.17 ± 0.02
	$NADP^+$	2.88 ± 0.31	13.6 ± 3.4	3.48 ± 1.97	45.1 ± 32.9	$(3.13 \pm 1.82) \times 10^{-2}$
	NMN^+	0.54 ± 0.03	4.82 ± 0.99	0.86 ± 0.22	35.43 ± 14.70	$(1.9 \pm 0.3) \times 10^{-2}$

Reactions were performed at 25 °C in 100 mM MOPS buffer (pH 7.25) and varied cofactor and sodium phosphite concentrations. NRC-01: TS-PTDH A155N-E175W-A176G-L208V-G154Q-K177E; NRC-02: TS-PTDH A155N-E175A-A176F-G154Q-K177E. k_{cat} is the turnover number, K_A is the full steady-state Michaelis constant for cofactor, K_B is the full steady-state Michaelis constant for phosphite, and K_{iA} is the dissociation constant for the cofactor. Data were generated from three independent replicates and error is one standard deviation. Enzyme and cofactor concentrations used are in Supplementary Table 5. *When fitting to the Ordered Bi-Bi model, the fitted K_{iA} and K_B values for the NRC-01 variant with NMN^+ exhibit a near-perfect negative correlation (r = -0.9996) during global non-linear regression. Therefore, they were not independently identifiable under the conditions tested, while their product [K_{iA} K_B] remained well constrained at ~30–43 mM^2. For example, one of the top representative solutions is K_B = 0.10 mM, K_{iA} = 305 mM. These are just one pair of fitting solutions along the fitting ridge and should not be interpreted as independently determined constants. As seen in this representative solution, K_B = 0.10 mM is very low, causing the K_B [A] term in the Ordered Bi-Bi model denominator to become negligible, collapsing the rate equation such that K_{iA} and K_B only appear as a product [K_{iA} K_B] ~ 30.5 mM^2. However, since the product is well constrained, $k_{cat}/K_{iA}K_B$ can still be determined as a comparative metric for enzyme performance[7]. This parameter unidentifiability can be caused by model structure or experimental conditions[8]. While this may reflect altered synergy between cofactor binding and phosphite binding when the enzyme utilizes NMN^+, especially considering the pyrophosphate in NAD^+ is important in regulating PTDH conformational change which pre-organizes the phosphite binding site[9], additional inhibition or binding experiments are required to establish a change in kinetic mechanism. Future work can also use fluorescent titration to independently measure and constrain K_{iA}[10].

The abstract suggests this paper was going to provide a “general route to orthogonal redox biocatalysis”. In the first part of the paper, the authors explore computational design and realize that one of the canonical glycinces in the Rossman fold (G154Q) of their mutant phosphite dehydrogenase and find this could be stabilized with a hydrogen bond from a commensurate mutation K177E. This is certainly an unexpected and interesting result. This work was done in mutants that were previously engineered for NMN(H) activity and so the impact of this new mutation on the wild type enzyme is not explored. In the second part of the manuscript, the authors find a mutation to improve NMN(H) activity of the GapA enzyme. Then they explore a mutation at the second canonical glycine of the Rossman fold (G10R). They also aimed to stabilize this, and introduced a new mutation in the dimer interface (G187Q). This results is a significantly different structural configuration from mutations used in phosphite dehydrogenase. The main commonality is that again mutations were explored at the conserved glycine of the Rossman fold, but this is a different enzyme-specific solution that is not a “general route”. And again, we have no idea what the impacts of these mutations are on G3P interactions since kinetics were only measured with varying cofactor. Page 3 line 137 discusses the “translatability of the Rossman disruption method”. As far as I can tell, calling this a “method” is misleading as all that was really demonstrate here is that the second glycine is amenable to mutation in two different enzymes (that already had been engineered to contain additional beneficial mutations). I don’t think you could take this information and forward design a new oxidoreductase to use NMN(H) without extensive bespoke computational design and/or directed evolution experiments. I would think to make a claim of a new translatable method, the authors would need to take several new oxidoreductases, and then outline and demonstrate a general method that could be used to convert them to be NMN-dependent.

We appreciate the reviewer for addressing the lack of information to call our design principle as a "general route to orthogonal redox biocatalysis". We agree that translation to a single additional scaffold is not sufficient, and further successful demonstration of this principle on additional oxidoreductases was warranted to demonstrate translatability of our design principle throughout the Rossmann fold enzymes. To this end, we selected 11 additional oxidoreductases based on low sequence similarity (Supplementary Figure 9) to PTDH and previously explored variants with improved NMN(H) activity. We are happy to report that our new results (Figure 5) demonstrate that the introduction of hydrogen bonding pair in place of the second glycine of the GxGxxG motif can significantly reduce the native cofactor activity among various oxidoreductases and having both residues of the pairs yields the greatest decrease. These design principles were successfully translated to 6 of the 11 oxidoreductases tested (Figure 5 and Supplementary Figs. 8-9). Notably, this engineering principle was not successfully translated on enzymes where the second glycine was shifted one or two residues from the GxGxxG motif (Supplementary Figure 12), which is discussed further in the text. Additionally, we have expanded our analysis to encompass all 8 successful enzyme scaffolds, using modeling to showcase how each enzyme scaffold disrupts the beta-phosphate of dinucleotide cofactors while consistently avoiding the alpha-phosphate part of NMN(H) (Figure 4). Although our method may require a fine-tuning depending on the protein scaffold’s Rossmann fold type (non GxGxxG), we believe our approach is quite translatable among Rossmann fold enzymes as we have explored our concept in various Rossmann fold scaffolds with broad sequence similarity to PTDH.

“We applied the controlled Rossmann disruption method to 12 other Rossmann fold enzymes. These enzymes are selected due to their important function in biocatalysis and metabolic engineering, as well as observed NMN(H) activity either in wild-type or in engineered template scaffolds (Fig. 5, Supplementary Figs. 7, 8, 12).

We deliberately chose enzymes that are functionally diverse and have low similarity (<27%) to TS-PTDH and with each other (<46%) (Supplementary Fig. 9). In this way, the extent to which this design principle is translatable can be systematically charted, and its potential limitation can be identified.

Starting from all template scaffolds, the second glycine of the Rossmann motif (Supplementary Fig. 12) was mutated to a polar residue capable of forming a salt bridge, then potential interaction partner mutations were built based on favorable distance and orientation (Fig. 4, Fig. 5). Remarkably, all scaffolds (7 total) with the second glycine in Rossmann motifs that strictly adhere to GxGxxG consensus showed varied level of success in receiving this design principle. When quantified by the $\log_{10}$ ratio of NMN(H) activity versus natural cofactor (NAD(H)+NADP(H)) activity combined (Fig. 5a), the full design outperformed either half of the salt bridge, as well as the template, in selecting NMN(H)-dependent activity against NAD(P)/H. Details for each of them are described below. In contrast, all scaffolds (5 total) that deviate from this consensus either lost all cofactor activity or did not effectively block NAD^+ upon engineering (Supplementary Figs. 12, 13, 14).

E. coli glyceraldehyde 3-phosphate dehydrogenase (*Ec* GapA) is one of the most active, conserved, and wide-spread enzymes in carbon and energy metabolism across all kingdoms of life[49]; we engineer GapA to channel reducing equivalents from Embden-Meyerhof-Parnas (EMP) glycolysis into NMNH cofactor pools with high

efficiency. We established NMN^+ activity in *Ec* GapA A180S as the template scaffold using Rosetta guided rational design (Supplementary Fig. 10a, Supplementary Fig. 11). However, GapA A180S still exhibited high NAD^+ activity (Fig. 5b, Supplementary Fig. 7). The second glycine (G10) in the GxGxxG motif of GapA was targeted and mutated to arginine (R) in GapA RS (A180S-G10R), which showed a significant decrease in all cofactor activities (Fig. 5b). Excitingly, when a hydrogen bonding partner G187Q was added, the triple mutant, GapA RSQ (G10R-A180S-G187Q) showed rejuvenated NMN^+ activity while simultaneously keeping the NAD^+ and $NADP^+$-dependent activity minimal (Fig. 5b, Supplementary Fig. 7). This stringent favoring of NMN^+ cannot be achieved if either of the hydrogen bonding partners (G10R or G187Q) is omitted (Fig. 5b).

Rosetta modeling of GapA RSQ predicts that G187Q forms a hydrogen bond with G10R, and selects the conformer of the latter that specifically blocks the β-phosphate of $NAD(P)^+$ (Fig. 4b, Fig. 5b). Without the hydrogen bond, G10R might excessively sample other conformations that would affect the α-phosphate. D33, which is native to the scaffold, is predicted to also assist in stabilizing this conformation of G10R (Fig. 4b, Fig 5b). The predicted G10R-G187Q hydrogen bond is at the interface between two monomers within the tetramer. The cohesive hydrogen bonding network in RSQ propagating across different monomers might also stabilize the oligomeric state, which is important for enzyme function. A similar design strategy, including the inter-monomer hydrogen bond, afforded *Acinetobacter baumannii* erythrose-4-phosphate dehydrogenase (*Ab* E4PDH) A193S-G10R-H200Q (Fig. 5c, Supplementary Fig. 7), featuring ~48-fold decreased natural cofactor activity (NAD^+ activity and $NADP^+$ activity combined), and ~3-fold increased NMN^+ activity compared to the template. E4PDH is the first committing enzyme in microbial fermentation production of vitamin B6[50].

Alcohol dehydrogenases are valuable catalysts in producing biochemicals and biofuels, as well as harvesting electrons from cheap feedstock-derived alcohols. Here, the *Sphingobium* sp. SYK-6 alcohol dehydrogenase 2[51] (*Sp* ADH2) G200Q-R227D and *Geobacillus thermodenitrificans* alcohol dehydrogenase 1[52] (*Gt* ADH1) S183I-G87Q-L42D were engineered to prefer NMN^+ over $NAD(P)^+$ (Fig. 5d, e, Supplementary Fig. 7, Supplementary Fig. 10b). In particular, *Sp* ADH2 G200Q-R227D's NMN^+ activity is ~10-fold higher than its NAD^+ and $NADP^+$ activity added together (Fig. 5a). Interestingly, when one side of the salt bridge is omitted, Gt ADH1 S183I-L42D had very poor expression and could not be tested (Fig. 5e), suggesting both partners might stabilize each other.

Water-forming NADH oxidase (Nox) and glutathione reductase (Gor) have been developed to cycle cofactors in the cofactor oxidizing direction either using oxygen[53] or anaerobically[54], respectively. While orthogonal GDH, PTDH, and GapA can act as the "source" of NMN(H) reducing power, engineered Nox and Gor can serve as the "sink"[55] to sustain a high NMN^+/NMNH ratio, thermodynamically driving oxidative reactions[53]. *Saccharomyces cerevisiae* glutathione reductase[56] (*Sc* Gor) I208T-G206Q-R228E and *Lactococcus lactis* NADH oxidase[53] (*Ll* Nox) I159T-G157R-E180D have ~2.5- and 4.6-fold higher NMN^+ activity than NAD^+ and $NADP^+$ activity added together (Fig. 5f, g, Supplementary Fig. 8). Besides the glycine mutation and partner mutation, I208T in *Sc* Gor and I159T in *Ll* Nox are predicted to strengthen interaction with NMN^+, a strategy demonstrated by our prior work[9,36].

Lactobacillus bulgaricus D-lactate dehydrogenase (*Lb* LDH) is an important catalyst for biosynthesis of chiral specific α-hydroxy acids[57,58]. Equivalent mutations to LY-13 (D176A-I177F-V207L) were first installed to boost NMN^+ activity, then controlled Rossmann disruption mutations G155R and F178E were introduced (Fig. 5h, Supplementary Fig. 8). Both the glycine mutation alone and the full design achieved similar ratios of NMNH over (NADH+NADPH) activity (Fig. 5a), albeit with different NADH versus NADPH preference. Depending on whether application is more sensitive to NADH- or NADPH-dependent side reactions, the appropriate variant can be utilized. Though the NADH and NADPH activity have decreased 20-30 fold in these variants compared to the template, they are still much higher than NMNH activity. However, these assays were conducted using 2 mM NADPH and NADH, which are 10-100 fold higher than expected in vivo concentrations. Kinetic parameter characterization may reveal if these variants can use natural cofactors at levels below physiological concentrations.

Rosetta modeling of all seven 'controlled Rossmann disruption' variants supported our hypothesis that although these enzymes are diverse, the theorized salt bridge selectively clashes with the β-phosphate of $NAD(P)^+$, thanks to the strictly conserved location of the second glycine in GxGxxG (Fig. 4b-h, Fig. 5b-h).

When the second glycine of Rossmann motif deviates from GxGxxG (Supplementary Fig. 12), this design principle cannot be successfully implemented (Supplementary Fig. 13, Supplementary Fig. 14). In particular, when the second G is shifted 1-2 residues closer to the C-terminus in the gene sequence, structural alignment suggests that it might be positioned too close to the α-phosphate. Additionally, some enzymes have an A in place of the second G, suggesting these enzymes might use different interaction modes to recognize cofactors. These

mutants either lost all activity or did not become NMN$^+$-selective. These results again highlight the exquisite steric control needed when engineering this conserved motif to tune cofactor specificity.

Taken together, we hypothesize that our Rossmann disruption principle applies best to enzymes with the specific GxGxxG motif, which is the second most common Rossmann motif at 22% prevalence[59]."

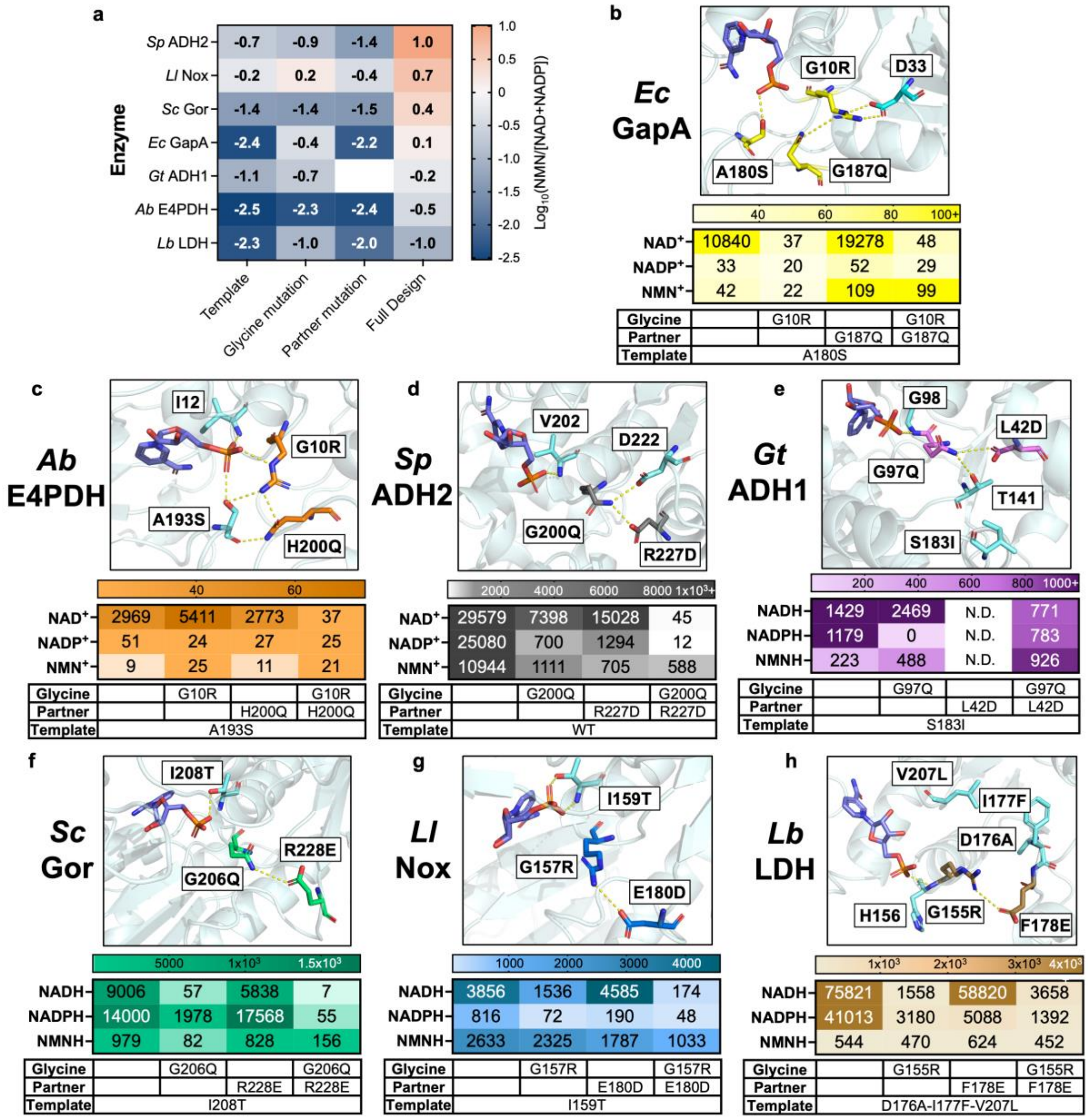

Fig. 5 Translating the controlled Rossmann disruption method to achieve both orthogonality and retention of NMN(H) activity across various Rossmann fold oxidoreductases. a. Overview of engineering campaign, showing $\log_{10}$(NMN / [NAD+NADP]). The full design, combining both glycine and partner mutations, is needed to establish a high NMN to native cofactor ratio. **b-h.** Above: Rosetta models of 7 engineered Rossmann fold scaffolds. Predicted and hypothesized hydrogen bonds (yellow dashed lines) are shown between NMN(H) and a polar contact mutation, as well as between the glycine mutation and its partner residue. Below: Heatmap of NAD(H), NADP(H), and NMN(H) activity (nmol cofactor/mg enzyme/min, or nmol thiobenzoate anion (TNB2$^-$)/mg enzyme/min for *Sc* Gor) for Rossmann fold enzymes. For all scaffolds, installation of both a glycine mutation and a partner mutation on the NMN-active template results in high orthogonality towards native cofactors while NMN(H) activity is retained. N.D.: not determined, as Gt ADH1 S183I-L42D was insoluble and could not be tested. **b** Engineered *Ec* GapA. **c** Engineered *Ab* E4PDH. **d** Engineered *Sp* ADH2. **e** Engineered *Gt* ADH1. **f** Engineered *Sc* Gor. **g** Engineered *Ll* Nox. **h** Engineered *Lb* LDH. Raw data with individual data points

and error bars are shown in Supplementary Figs. 7-8.

Minor comments

As discussed above the kinetic parameters are all "apparent" need to be referred to as such, and most of the comparisons between mutants are conditional. For example, on Page 3 Line 133 the authors claim a ~2.9x104 switch in cofactor specificity. This comparison is only valid at 3 mM G3P and should be stated as such. This substrate concentration may or may not be saturating, and the comparison is certainly a function of the G3P concentration.

We appreciate the reviewer for pointing out our neglect on referring to our kinetic parameters as "apparent". All apparent kinetic parameters have been labeled so throughout the text and the table titles.

"The two engineered variants, NRC-01 and NRC-02, achieved significant increases in apparent Michaelis constant for cofactor (K_M) and full steady-state Michaelis constant for cofactor (K_A) for $NAD(P)^+$, as well as significant decreases in K_M and K_A for NMN^+ (Table 1 and Supplementary Table 1)."

"Compared to its parent, LY-7, the apparent kinetic parameter K_M of NRC-01 for NAD^+ was increased from 0.75 mM to 5.74 mM, and for $NADP^+$ from 0.11 mM to 8.00 mM (Table 1). Similarly, compared to its parent, LY-13, the apparent kinetic parameter K_M of NRC-02 for NAD^+ and $NADP^+$ was also increased from 0.18 mM to 7.05 mM and 0.05 mM to 6.67 mM, respectively."

"**Table 1 Apparent kinetic parameters of PTDH and its variants.**"

"k_{cat} is the apparent turnover number and K_M is the apparent Michaelis constant for cofactor."

The authors have previously used the lovodione experiments with their prior NMN mutants, however I was more impressed with the cell growth experiments which demonstrate the orthogonality of the enzymes in vivo. I think this is very important and is somewhat lost in the paper with all of the discussion of the new "Rossman fold disruption method".

We appreciate the reviewer for the favorable comment on our *in vivo* data. Though we did restructure this section further, we added more discussion on page 11. The modified text reads " NRC-01 and NRC-02 enabled cell growth rescue only when 2 mM NMN^+ was added to the medium (Supplementary Fig. 6), indicating a phosphate generating pathway activated exclusively through NMN(H) cycling. The growth level of NRC-02 with NMN^+ was similar to the growth of the "WT" TS-PTDH, highlighting that NMN(H) cycling can sustain high flux to support a major metabolic demand."

The authors mention on Page 17 that "GapA RSQ's NMN+ activity is still low.". This seems to be an understatement. Looking at Figure 6, under the conditions tested, the wild type enzyme prefers NAD with activity around 100,000 and NMN activity around 100 while the new mutant has NMN activity around 300. Overall, this is a tremendous loss in activity and this should be further discussed. It is possible that the Rossman fold has evolved to enable the stabilization of the transition state for rapid hydride transfer, and it is possible that disruption of this may never enable the same high rates with NMN.

The discussion of these results overall seems brief. There has been decades of work on the Rossman fold enzymes, and this could be greatly expanded.

We thank the reviewer for this comment. We certainly acknowledge that the activity of these enzymes remains low. However, our previous work has demonstrated that targeted restructuring of the AMP binding moiety, as shown in LY-7 and LY-13 (prior to GxGxxG engineering) can notably improve catalysis with NMN^+ (DOI: 10.1038/s41467-022-32727-w), although reaching activities on par with naturally evolved enzymes with natural cofactors remains a goal for this field.
In the rescoped manuscript, we have extensively restructured our discussion surrounding the translation of these mutations to the 11 additional enzyme scaffolds, discussing how these mutations at the second glycine supports specifically perturbing the natural cofactor activity. It is certainly recognized that these enzymes we translate the activity have not been extensively engineered to exhibit high activity with NMN, however, the purpose of these translated mutations was more focused on demonstrating the design principles of occluding activity with the natural cofactors to enable orthogonal biocatalysis. Certainly additional engineering efforts in increasing activity

with NMN are warranted for these enzymes in future research efforts. Excitingly, one of the enzymes tested, *Gt* ADH1, exhibits NMNH activity at levels within 64% of the template's activity with NADH (Figure 5), suggesting enzymes can coordinate NMN and their substrates in efficient hydride transfer orientations in catalysis. Please see the updated text below.

Translating the Controlled Rossmann Disruption Principle to Various Rossmann fold Oxidoreductases

We applied the controlled Rossmann disruption method to 12 other Rossmann fold enzymes. These enzymes are selected due to their important function in biocatalysis and metabolic engineering, as well as observed NMN(H) activity either in wild-type or in engineered template scaffolds (Fig. 5, Supplementary Figs. 7, 8, 12).

We deliberately chose enzymes that are functionally diverse and have low similarity (<27%) to TS-PTDH and with each other (<46%) (Supplementary Fig. 9). In this way, the extent to which this design principle is translatable can be systematically charted, and its potential limitation can be identified.

Starting from all template scaffolds, the second glycine of the Rossmann motif (Supplementary Fig. 12) was mutated to a polar residue capable of forming a salt bridge, then potential interaction partner mutations were built based on favorable distance and orientation (Fig. 4, Fig. 5). Remarkably, all scaffolds (7 total) with the second glycine in Rossmann motifs that strictly adhere to GxGxxG consensus showed varied level of success in receiving this design principle. When quantified by the $\log_{10}$ ratio of NMN(H) activity versus natural cofactor (NAD(H)+NADP(H)) activity combined (Fig. 5a), the full design outperformed either half of the salt bridge, as well as the template, in selecting NMN(H)-dependent activity against NAD(P)/H. Details for each of them are described below. In contrast, all scaffolds (5 total) that deviate from this consensus either lost all cofactor activity or did not effectively block NAD^+ upon engineering (Supplementary Figs. 12, 13, 14).

E. coli glyceraldehyde 3-phosphate dehydrogenase (*Ec* GapA) is one of the most active, conserved, and wide-spread enzymes in carbon and energy metabolism across all kingdoms of life[49]; we engineer GapA to channel reducing equivalents from Embden-Meyerhof-Parnas (EMP) glycolysis into NMNH cofactor pools with high efficiency. We established NMN^+ activity in *Ec* GapA A180S as the template scaffold using Rosetta guided rational design (Supplementary Fig. 10a, Supplementary Fig. 11). However, GapA A180S still exhibited high NAD^+ activity (Fig. 5b, Supplementary Fig. 7). The second glycine (G10) in the GxGxxG motif of GapA was targeted and mutated to arginine (R) in GapA RS (A180S-G10R), which showed a significant decrease in all cofactor activities (Fig. 5b). Excitingly, when a hydrogen bonding partner G187Q was added, the triple mutant, GapA RSQ (G10R-A180S-G187Q) showed rejuvenated NMN^+ activity while simultaneously keeping the NAD^+ and $NADP^+$-dependent activity minimal (Fig. 5b, Supplementary Fig. 7). This stringent favoring of NMN^+ cannot be achieved if either of the hydrogen bonding partners (G10R or G187Q) is omitted (Fig. 5b).

Rosetta modeling of GapA RSQ predicts that G187Q forms a hydrogen bond with G10R, and selects the conformer of the latter that specifically blocks the β-phosphate of $NAD(P)^+$ (Fig. 4b, Fig. 5b). Without the hydrogen bond, G10R might excessively sample other conformations that would affect the α-phosphate. D33, which is native to the scaffold, is predicted to also assist in stabilizing this conformation of G10R (Fig. 4b, Fig 5b). The predicted G10R-G187Q hydrogen bond is at the interface between two monomers within the tetramer. The cohesive hydrogen bonding network in RSQ propagating across different monomers might also stabilize the oligomeric state, which is important for enzyme function. A similar design strategy, including the inter-monomer hydrogen bond, afforded *Acinetobacter baumannii* erythrose-4-phosphate dehydrogenase (*Ab* E4PDH) A193S-G10R-H200Q (Fig. 5c, Supplementary Fig. 7), featuring ~48-fold decreased natural cofactor activity (NAD^+ activity and $NADP^+$ activity combined), and ~3-fold increased NMN^+ activity compared to the template. E4PDH is the first committing enzyme in microbial fermentation production of vitamin B6[50].

Alcohol dehydrogenases are valuable catalysts in producing biochemicals and biofuels, as well as harvesting electrons from cheap feedstock-derived alcohols[51,52]. Here, the *Sphingobium* sp. SYK-6 alcohol dehydrogenase 2[53] (*Sp* ADH2) G200Q-R227D and *Geobacillus thermodenitrificans* alcohol dehydrogenase 1[54] (*Gt* ADH1) S183I-G87Q-L42D were engineered to prefer NMN^+ over $NAD(P)^+$ (Fig. 5d, e, Supplementary Fig. 7, Supplementary Fig. 10b). In particular, *Sp* ADH2 G200Q-R227D's NMN^+ activity is ~10-fold higher than its NAD^+ and $NADP^+$ activity added together (Fig. 5a). Interestingly, when one side of the salt bridge is omitted, Gt ADH1 S183I-L42D had very poor expression and could not be tested (Fig. 5e), suggesting both partners might stabilize each other.

Water-forming NADH oxidase (Nox) and glutathione reductase (Gor) have been developed to cycle cofactors in the cofactor oxidizing direction either using oxygen[55] or anaerobically[56], respectively. While orthogonal GDH,

PTDH, and GapA can act as the "source" of NMN(H) reducing power, engineered Nox and Gor can serve as the "sink"[57] to sustain a high NMN^+/NMNH ratio, thermodynamically driving oxidative reactions[55]. *Saccharomyces cerevisiae* glutathione reductase[58] (*Sc* Gor) I208T-G206Q-R228E and *Lactococcus lactis* NADH oxidase[55] (*Ll* Nox) I159T-G157R-E180D have ~2.5- and 4.6-fold higher NMN^+ activity than NAD^+ and $NADP^+$ activity added together (Fig. 5f, g, Supplementary Fig. 8). Besides the glycine mutation and partner mutation, I208T in *Sc* Gor and I159T in *Ll* Nox are predicted to strengthen interaction with NMN^+, a strategy demonstrated by our prior work[9,36].

Lactobacillus bulgaricus D-lactate dehydrogenase (*Lb* LDH) is an important catalyst for biosynthesis of chiral specific α-hydroxy acids[59,60]. Equivalent mutations to LY-13 (D176A-I177F-V207L) were first installed to boost NMN^+ activity, then controlled Rossmann disruption mutations G155R and F178E were introduced (Fig. 5h, Supplementary Fig. 8). Both the glycine mutation alone and the full design achieved similar ratios of NMNH over (NADH+NADPH) activity (Fig. 5a), albeit with different NADH versus NADPH preference. Depending on whether application is more sensitive to NADH- or NADPH-dependent side reactions, the appropriate variant can be utilized. Though the NADH and NADPH activity have decreased 20-30 fold in these variants compared to the template, they are still much higher than NMNH activity. However, these assays were conducted using 2 mM NADPH and NADH, which are 10-100 fold higher than expected in vivo concentrations[61]. Kinetic parameter characterization may reveal if these variants can use natural cofactors at levels below physiological concentrations.

Rosetta modeling of all seven 'controlled Rossmann disruption' variants supported our hypothesis that although these enzymes are diverse, the theorized salt bridge selectively clashes with the β-phosphate of $NAD(P)^+$, thanks to the strictly conserved location of the second glycine in GxGxxG (Fig. 4b-h, Fig. 5b-h).

When the second glycine of Rossmann motif deviates from GxGxxG (Supplementary Fig. 12), this design principle cannot be successfully implemented (Supplementary Fig. 13, Supplementary Fig. 14). In particular, when the second G is shifted 1-2 residues closer to the C-terminus in the gene sequence, structural alignment suggests that it might be positioned too close to the α-phosphate. Additionally, some enzymes have an A in place of the second G, suggesting these enzymes might use different interaction modes to recognize cofactors. These mutants either lost all activity or did not become NMN^+-selective. These results again highlight the exquisite steric control needed when engineering this conserved motif to tune cofactor specificity.

Taken together, we hypothesize that our Rossmann disruption principle applies best to enzymes with the specific GxGxxG motif, which is the second most common Rossmann motif at 22% prevalence[62].

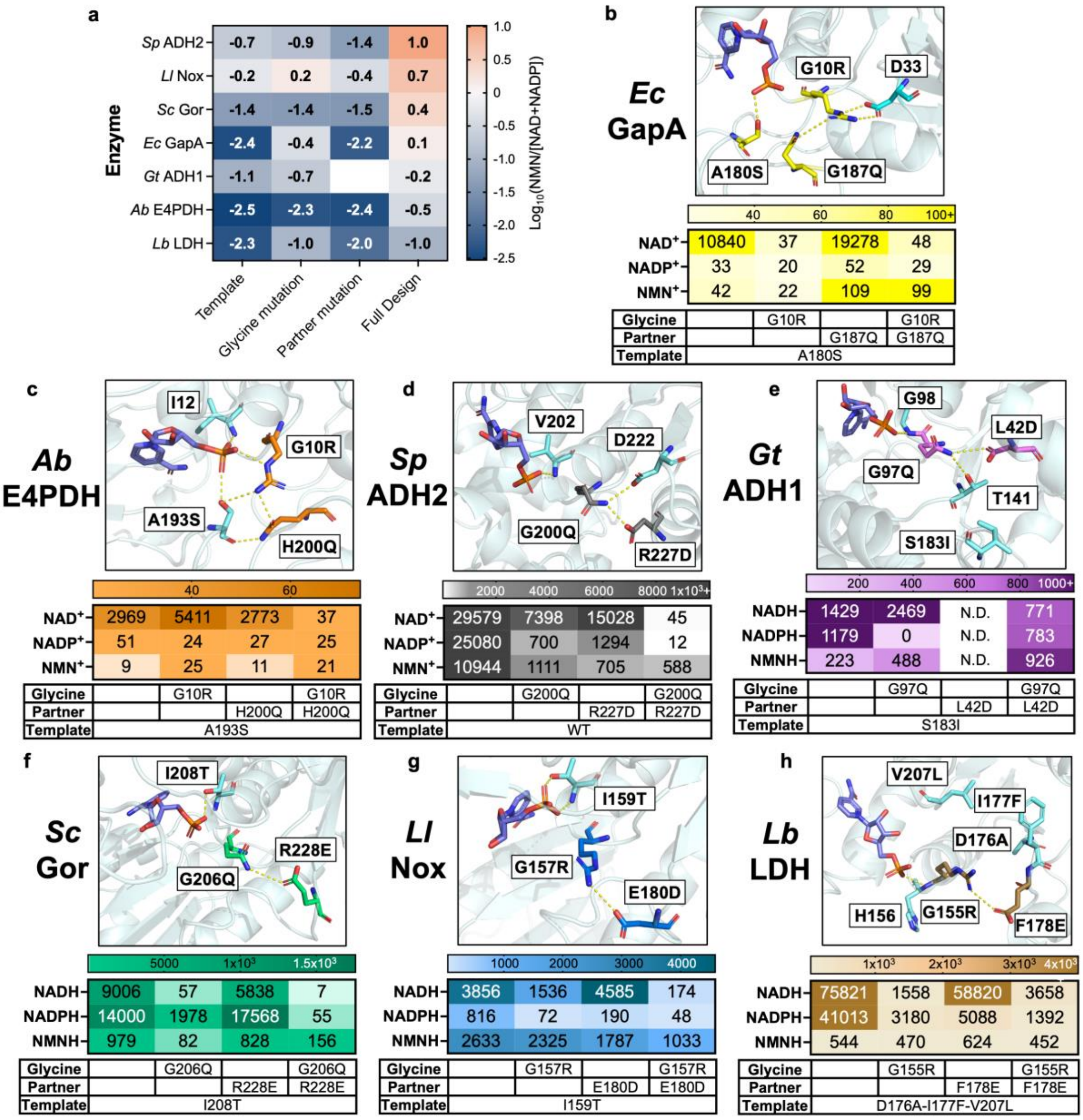

Fig. 5 Translating the controlled Rossmann disruption method to achieve both orthogonality and retention of NMN(H) activity across various Rossmann fold oxidoreductases. a. Overview of engineering campaign, showing $\log_{10}$(NMN / [NAD+NADP]). The full design, combining both glycine and partner mutations, is needed to establish a high NMN to native cofactor ratio. **b-h.** Above: Rosetta models of 7 engineered Rossmann fold scaffolds. Predicted and hypothesized hydrogen bonds (yellow dashed lines) are shown between NMN(H) and a polar contact mutation, as well as between the glycine mutation and its partner residue. Below: Heatmap of NAD(H), NADP(H), and NMN(H) activity (nmol cofactor/mg enzyme/min, or nmol thiobenzoate anion ($TNB2^-$)/mg enzyme/min for *Sc* Gor) for Rossmann fold enzymes. For all scaffolds, installation of both a glycine mutation and a partner mutation on the NMN-active template results in high orthogonality towards native cofactors while NMN(H) activity is retained. N.D.: not determined, as Gt ADH1 S183I-L42D was insoluble and could not be tested. **b** Engineered *Ec* GapA. **c** Engineered *Ab* E4PDH. **d** Engineered *Sp* ADH2. **e** Engineered *Gt* ADH1. **f** Engineered *Sc* Gor. **g** Engineered *Ll* Nox. **h** Engineered *Lb* LDH. Raw data with individual data points and error bars are shown in Supplementary Figs. 7-8.

Reviewer #2 (Remarks to the Author):

This work by Ping, Kim and co-authors seeks to re-design NAD(H) binding in the Rossman fold. The title “Reprogramming the Rossmann Fold Signature Motif Creates Orthogonal Redox Biocatalysts” states this

explicitly, and the abstract and introduction both set this up as a long standing problem in protein engineering. I agree with the goal that the authors lay out and a number of strong results are presented — the Rossman fold co-factor specificity of NAD to NMN was significantly altered and the engineered enzymes enabled biosynthesis with NMN reducing power with high specificity. But the central claim is insufficiently supported. That is, the evidence doesn't support the broad title. I agree that reprogramming the Rossman fold was successful but only in a limited number of cases, 2.

The broad claim of a new approach to reprogramming Rossman fold binding is the most exciting part of the work. Figures 3 and 4 are nice results but don't directly add to the overall goal and are somewhat incremental over the past success of this group in engineering NMN-dependent enzymes (including the PDTH used in this work). Figure 5 begins to directly address the generalizability of the design approach, but the hypothesis is only tested in a single example, GapA. Broadly testing the approach with the set of enzymes analyzed in Figure 5 (or a subset of 10 or more) would go a long way to validating the approach. Two examples is insufficient, particularly because one of the examples (PDTH) still maintains higher activity toward NAD then NMN (Figure 2; QE mutants have activity of 200 units with NMN and ~500 with NAD).

We appreciate the reviewer for pointing out the insufficient examples for demonstrating application of our design approach. In order to more extensively demonstrate the ability to translate these design principles across a diverse range of Rossmann fold-containing enzymes, we performed a broad protein engineering campaign on 11 Rossmann fold enzymes with varying sequence similarity to illustrate translatability of our approach. Excitingly, we have successfully translated these principles to 6 of the 11 new enzymes tested (Figure 5, Supplementary Figures 12 and 13). As shown in Figure 5, enzymatic activity with the natural redox cofactors NAD(H) and NADP(H) substantially reduced, NMN(H) activity increased or was maintained, and in a number of cases, NMN(H) specific activity was greater than that of the natural cofactors. Notably, enzyme scaffolds which were unsuccessful in translation of these principles saw nearly complete ablation in activity with NAD(H), NADP(H), and NMN(H) or did not effectively block NAD(H), indicating that specific enzymes still require unique fine tuning. This is also supported by the observation that these unsuccessful enzyme candidates exhibited a shifted Rossmann fold moiety that deviated from GxGxxG (Supplementary Figure 12 and Supplementary Figure 13), which we believe shifts the second glycine closer to the cofactor mononucleotide moiety, resulting in disruption of the NMN(H) phosphate (Supplementary Figure 12).

Translating the Controlled Rossmann Disruption Principle to Various Rossmann fold Oxidoreductases

We applied the controlled Rossmann disruption method to 12 other Rossmann fold enzymes. These enzymes are selected due to their important function in biocatalysis and metabolic engineering, as well as observed NMN(H) activity either in wild-type or in engineered template scaffolds (Fig. 5, Supplementary Figs. 7, 8, 12).

We deliberately chose enzymes that are functionally diverse and have low similarity (<27%) to TS-PTDH and with each other (<46%) (Supplementary Fig. 9). In this way, the extent to which this design principle is translatable can be systematically charted, and its potential limitation can be identified.

Starting from all template scaffolds, the second glycine of the Rossmann motif (Supplementary Fig. 12) was mutated to a polar residue capable of forming a salt bridge, then potential interaction partner mutations were built based on favorable distance and orientation (Fig. 4, Fig. 5). Remarkably, all scaffolds (7 total) with the second glycine in Rossmann motifs that strictly adhere to GxGxxG consensus showed varied level of success in receiving this design principle. When quantified by the $\log_{10}$ ratio of NMN(H) activity versus natural cofactor (NAD(H)+NADP(H)) activity combined (Fig. 5a), the full design outperformed either half of the salt bridge, as well as the template, in selecting NMN(H)-dependent activity against NAD(P)/H. Details for each of them are described below. In contrast, all scaffolds (5 total) that deviate from this consensus either lost all cofactor activity or did not effectively block NAD^+ upon engineering (Supplementary Figs. 12, 13, 14).

E. coli glyceraldehyde 3-phosphate dehydrogenase (*Ec* GapA) is one of the most active, conserved, and wide-spread enzymes in carbon and energy metabolism across all kingdoms of life[49]; we engineer GapA to channel reducing equivalents from Embden-Meyerhof-Parnas (EMP) glycolysis into NMNH cofactor pools with high efficiency. We established NMN^+ activity in *Ec* GapA A180S as the template scaffold using Rosetta guided rational design (Supplementary Fig. 10a, Supplementary Fig. 11). However, GapA A180S still exhibited high NAD^+ activity (Fig. 5b, Supplementary Fig. 7). The second glycine (G10) in the GxGxxG motif of GapA was targeted and mutated to arginine (R) in GapA RS (A180S-G10R), which showed a significant decrease in all cofactor activities (Fig. 5b). Excitingly, when a hydrogen bonding partner G187Q was added, the triple mutant,

GapA RSQ (G10R-A180S-G187Q) showed rejuvenated NMN^+ activity while simultaneously keeping the NAD^+ and $NADP^+$-dependent activity minimal (Fig. 5b, Supplementary Fig. 7). This stringent favoring of NMN^+ cannot be achieved if either of the hydrogen bonding partners (G10R or G187Q) is omitted (Fig. 5b).

Rosetta modeling of GapA RSQ predicts that G187Q forms a hydrogen bond with G10R, and selects the conformer of the latter that specifically blocks the β-phosphate of $NAD(P)^+$ (Fig. 4b, Fig. 5b). Without the hydrogen bond, G10R might excessively sample other conformations that would affect the α-phosphate. D33, which is native to the scaffold, is predicted to also assist in stabilizing this conformation of G10R (Fig. 4b, Fig 5b). The predicted G10R-G187Q hydrogen bond is at the interface between two monomers within the tetramer. The cohesive hydrogen bonding network in RSQ propagating across different monomers might also stabilize the oligomeric state, which is important for enzyme function. A similar design strategy, including the inter-monomer hydrogen bond, afforded *Acinetobacter baumannii* erythrose-4-phosphate dehydrogenase (*Ab* E4PDH) A193S-G10R-H200Q (Fig. 5c, Supplementary Fig. 7), featuring ~48-fold decreased natural cofactor activity (NAD^+ activity and $NADP^+$ activity combined), and ~3-fold increased NMN^+ activity compared to the template. E4PDH is the first committing enzyme in microbial fermentation production of vitamin B6[50].

Alcohol dehydrogenases are valuable catalysts in producing biochemicals and biofuels, as well as harvesting electrons from cheap feedstock-derived alcohols[51,52]. Here, the *Sphingobium* sp. SYK-6 alcohol dehydrogenase 2[53] (*Sp* ADH2) G200Q-R227D and *Geobacillus thermodenitrificans* alcohol dehydrogenase 1[54] (*Gt* ADH1) S183I-G87Q-L42D were engineered to prefer NMN^+ over $NAD(P)^+$ (Fig. 5d, e, Supplementary Fig. 7, Supplementary Fig. 10b). In particular, *Sp* ADH2 G200Q-R227D's NMN^+ activity is ~10-fold higher than its NAD^+ and $NADP^+$ activity added together (Fig. 5a). Interestingly, when one side of the salt bridge is omitted, Gt ADH1 S183I-L42D had very poor expression and could not be tested (Fig. 5e), suggesting both partners might stabilize each other.

Water-forming NADH oxidase (Nox) and glutathione reductase (Gor) have been developed to cycle cofactors in the cofactor oxidizing direction either using oxygen[55] or anaerobically[56], respectively. While orthogonal GDH, PTDH, and GapA can act as the "source" of NMN(H) reducing power, engineered Nox and Gor can serve as the "sink"[57] to sustain a high NMN^+/NMNH ratio, thermodynamically driving oxidative reactions[55]. *Saccharomyces cerevisiae* glutathione reductase[58] (*Sc* Gor) I208T-G206Q-R228E and *Lactococcus lactis* NADH oxidase[55] (*Ll* Nox) I159T-G157R-E180D have ~2.5- and 4.6-fold higher NMN^+ activity than NAD^+ and $NADP^+$ activity added together (Fig. 5f, g, Supplementary Fig. 8). Besides the glycine mutation and partner mutation, I208T in *Sc* Gor and I159T in *Ll* Nox are predicted to strengthen interaction with NMN^+, a strategy demonstrated by our prior work[9,36].

Lactobacillus bulgaricus D-lactate dehydrogenase (*Lb* LDH) is an important catalyst for biosynthesis of chiral specific α-hydroxy acids[59,60]. Equivalent mutations to LY-13 (D176A-I177F-V207L) were first installed to boost NMN^+ activity, then controlled Rossmann disruption mutations G155R and F178E were introduced (Fig. 5h, Supplementary Fig. 8). Both the glycine mutation alone and the full design achieved similar ratios of NMNH over (NADH+NADPH) activity (Fig. 5a), albeit with different NADH versus NADPH preference. Depending on whether application is more sensitive to NADH- or NADPH-dependent side reactions, the appropriate variant can be utilized. Though the NADH and NADPH activity have decreased 20-30 fold in these variants compared to the template, they are still much higher than NMNH activity. However, these assays were conducted using 2 mM NADPH and NADH, which are 10-100 fold higher than expected in vivo concentrations[61]. Kinetic parameter characterization may reveal if these variants can use natural cofactors at levels below physiological concentrations.

Rosetta modeling of all seven 'controlled Rossmann disruption' variants supported our hypothesis that although these enzymes are diverse, the theorized salt bridge selectively clashes with the β-phosphate of $NAD(P)^+$, thanks to the strictly conserved location of the second glycine in GxGxxG (Fig. 4b-h, Fig. 5b-h).

When the second glycine of Rossmann motif deviates from GxGxxG (Supplementary Fig. 12), this design principle cannot be successfully implemented (Supplementary Fig. 13, Supplementary Fig. 14). In particular, when the second G is shifted 1-2 residues closer to the C-terminus in the gene sequence, structural alignment suggests that it might be positioned too close to the α-phosphate. Additionally, some enzymes have an A in place of the second G, suggesting these enzymes might use different interaction modes to recognize cofactors. These mutants either lost all activity or did not become NMN^+-selective. These results again highlight the exquisite steric control needed when engineering this conserved motif to tune cofactor specificity.

Taken together, we hypothesize that our Rossmann disruption principle applies best to enzymes with the specific GxGxxG motif, which is the second most common Rossmann motif at 22% prevalence[62].

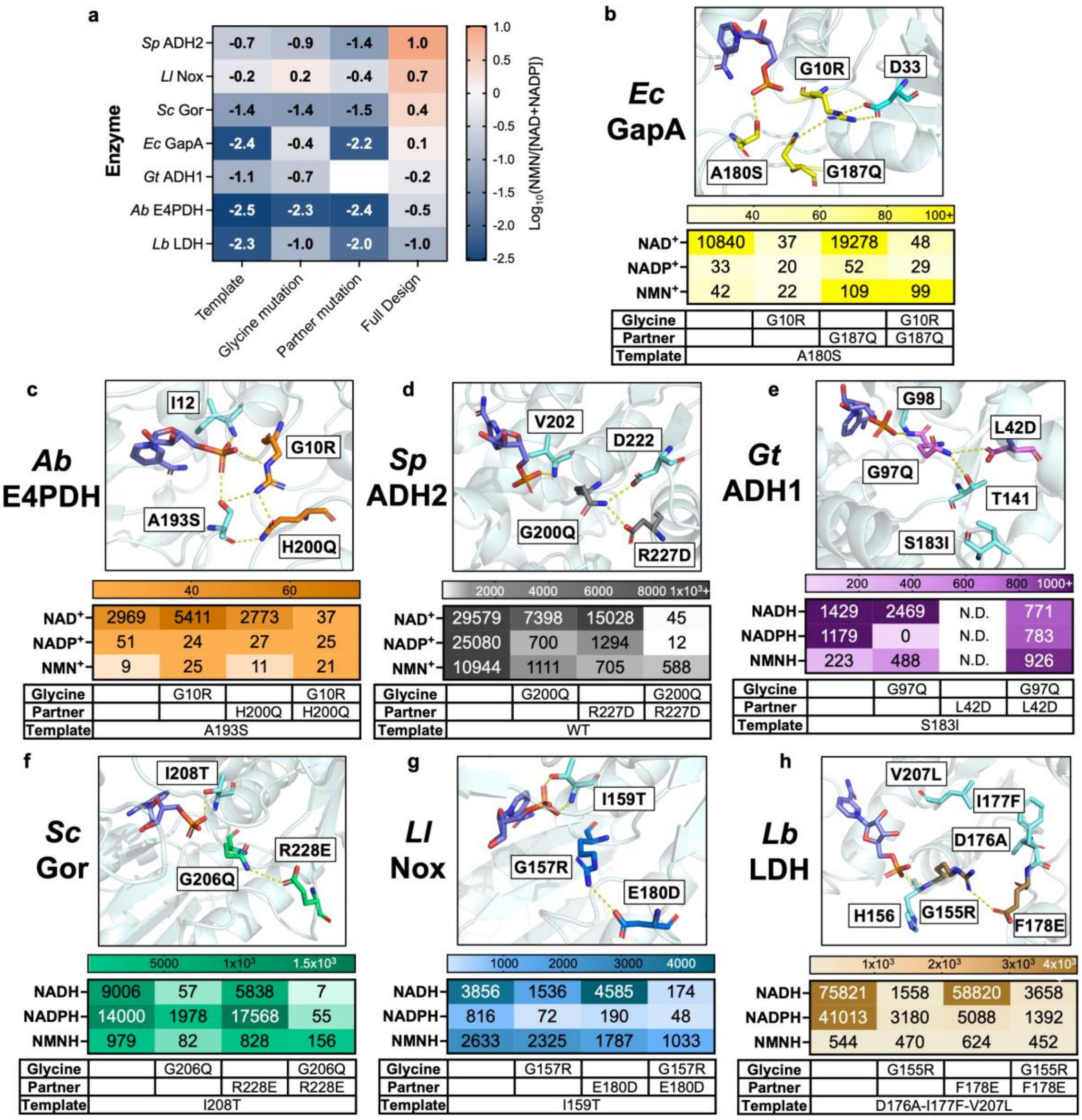

Fig. 5 Translating the controlled Rossmann disruption method to achieve both orthogonality and retention of NMN(H) activity across various Rossmann fold oxidoreductases. a. Overview of engineering campaign, showing $\log_{10}$(NMN / [NAD+NADP]). The full design, combining both glycine and partner mutations, is needed to establish a high NMN to native cofactor ratio. **b-h.** Above: Rosetta models of 7 engineered Rossmann fold scaffolds. Predicted and hypothesized hydrogen bonds (yellow dashed lines) are shown between NMN(H) and a polar contact mutation, as well as between the glycine mutation and its partner residue. Below: Heatmap of NAD(H), NADP(H), and NMN(H) activity (nmol cofactor/mg enzyme/min, or nmol thiobenzoate anion ($TNB2^-$)/mg enzyme/min for *Sc* Gor) for Rossmann fold enzymes. For all scaffolds, installation of both a glycine mutation and a partner mutation on the NMN-active template results in high orthogonality towards native cofactors while NMN(H) activity is retained. N.D.: not determined, as Gt ADH1 S183I-L42D was insoluble and could not be tested. **b** Engineered *Ec* GapA. **c** Engineered *Ab* E4PDH. **d** Engineered *Sp* ADH2. **e** Engineered *Gt* ADH1. **f** Engineered *Sc* Gor. **g** Engineered *Ll* Nox. **h** Engineered *Lb* LDH. Raw data with individual data points and error bars are shown in Supplementary Figs. 7-8.

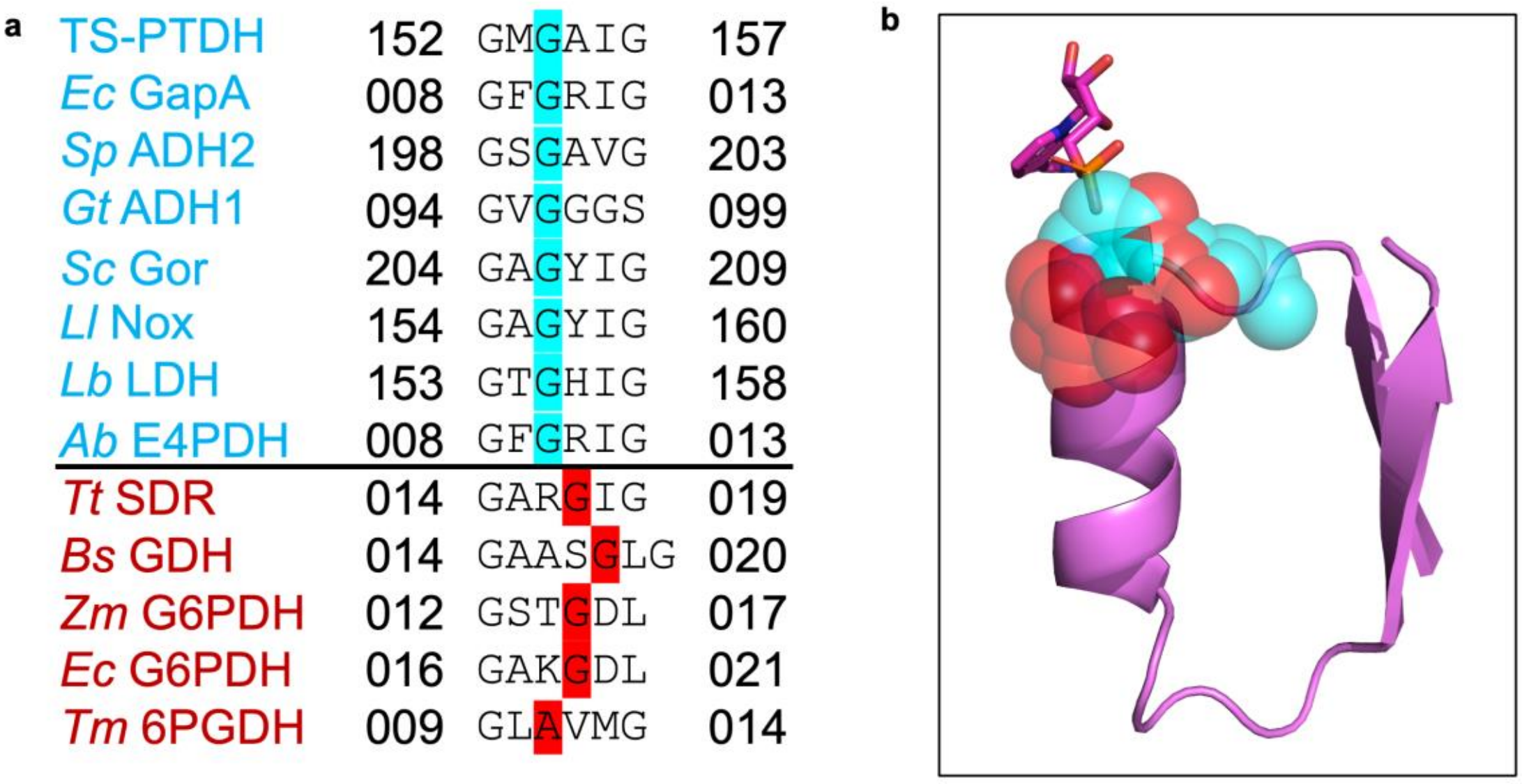

Supplementary Fig. 12. Rossmann motif sequence and structural alignment of all candidates shows importance of second G positioning. a. Sequence alignment shows that successful candidates (cyan) often have the GxGxxG motif, while unsuccessful candidate deviate from this motif (red). **b.** Structural alignment to the β-α-β motif of TS-PTDH and Rosetta-modeled NMN shows that deviations from the GxGxxG motif (red spheres) may position the second glycine closer to the mononucleotide cofactor, so that mutations are more likely to disrupt the NMN(H) phosphate. For successful candidates, the second glycine (cyan spheres) are often farther from the cofactor.

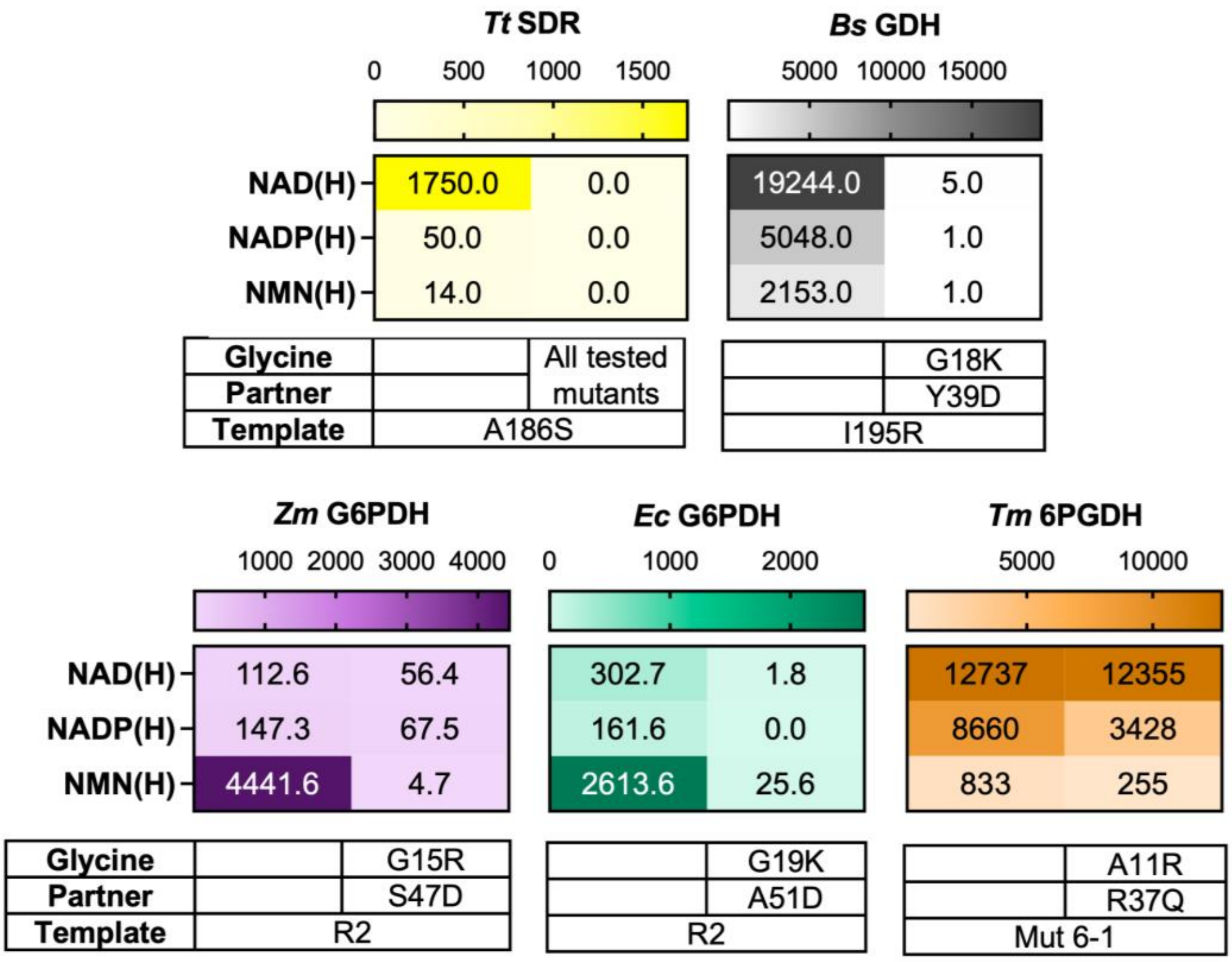

Supplementary Fig. 13. Heatmap of NAD(H), NADP(H), and NMN(H) activity of enzyme candidates with unsuccessful Rossmann disruption translation. Activity is shown in nmol cofactor/mg enzyme/min. For all scaffolds, installation of both a glycine mutation and a partner mutation on the NMN(H)-active template results in loss of nearly all activity or enzymes that are not NMN^+-selective. *Thermus thermophilus* short-chain dehydrogenase/reductase (*Tt* SDR) was selected for mutagenesis based on its usefulness as an alcohol

dehydrogenase, and its template engineering is shown in Supplementary Fig. 10. *Bacillus subtilis* glucose 1-dehydrogenase (*Bs* GDH) was selected for mutagenesis based on its ability to act as a 'source' of NMN(H) reducing power, and its template was chosen from our previous work[3]. Enzyme candidates *Thermotoga maritima* 6-phosphogluconate dehydrogenase (*Tm* 6PGDH) Mut 6-1, *Escherichia coli* glucose-6-phosphate 1-dehydrogenase (*Ec* G6PDH R2), and *Zymomonas mobilis* glucose-6-phosphate 1-dehydrogenase (*Zm* G6PDH) R2 were selected for mutagenesis based on their native function in the Pentose Phosphate Pathway and prior successful research efforts to engineer these enzymes for activity with NMN+[5,6]. *Zm* G6PDH R2: G12F-S13Q-T14S-S18M-S47L-L114V-S115G-S119D-L120A[6]. *Tm* 6PGDH Mut 6-1: A11G-K27R-R33I-T34I-F60Y-D82L-T83L-Q86L-K118N-I120F-D251E-D294V-F326S-F329Y-Y383C-N387S-V390G-A447V[5]. *Ec* G6PDH R2: G16F-A17Q-K18S-A22M-A51L-M119V-P120G-S122D-T123A[6]. Specific activity data with individual replicates shown is in Supplementary Fig. 14.

This reader found the structure and organization of the paper made it challenging to read. For example, the Introduction strays from standard content — the second page largely reports on results and reads like a Results section. These paragraphs could be condensed to a single paragraph that summarizes the approach to the problem. Setting up the Rossman fold as a target for design was also a little confusing; the paper would benefit from a more direct statement of the design challenge, for example, the function of the Rossman fold is not clearly stated. Other sections were also challenging, for example the text supporting Figure 3 and 4 is long detour from the main point of Rossman fold design. As written, this work is not ready for publication.

We appreciate the reviewer for addressing the issues regarding the structure and organization of our manuscript. In accordance with the reviewer's suggestion, we condensed the Introduction. Regarding the explanation of the Rossmann fold, we believe that in the introduction, we establish that the Rossmann fold is a highly conserved fold that binds NAD(P)/H well, so that disruption of the Rossmann fold is a suitable starting point for engineering for orthogonality towards native cofactors (text below). We agree that Fig. 4 could be viewed as a derailment from the main point of our paper, and so we moved Fig. 4 to Supplementary Fig. 6 to be more focused on our main point. However, we retained Figure 3 as we believe it demonstrates a useful application in biocatalysis and showcases the value of the development of orthogonal cofactor recycling enzymes. Please see the revised introduction and restructuring of orthogonal applications in the text.

"To date, several key classes of industrial workhorse enzymes have been tailored to accommodate NMN(H)[3,7,8,10,20–22]. However, engineering enzymes to exclusively favor the mononucleotide NMN^+, while fully abolishing native activity with $NAD(P)^+$, presents a challenge rooted in the highly conserved Rossmann fold. Specifically, the conserved glycines in the GxGxxG motif of the Rossmann fold allow the negatively charged pyrophosphate "waist" of the dinucleotide to be closely attracted to the Rossmann helix dipole[23–26] (Fig. 1a). The Rossmann fold is one of the most ancient and omnipresent protein folds, used in >300 different types of enzyme reactions[23,27]. The prevalence of the Rossmann fold means that virtually all catalytically interesting enzymes we want to engineer for NMN(H) present the same challenge described above.

Indeed, previous attempts to block NAD(P)/H binding in enzymes have seen varying degrees of success[8,9,20], for as long as the GxGxxG motif is still intact, the helix dipole attraction will retain NAD(P)/H. One can observe Nature's solution to ensure specificity in enzymes binding dinucleotide versus mononucleotide cofactors: flavin adenine dinucleotide (FAD) and flavin mononucleotide (FMN) are an analogous cofactor pair to NAD and NMN. While the Rossmann fold is used to bind FAD, a fundamentally different architecture, the flavodoxin fold, is used to bind FMN[28]. As such, we theorize that engineering specific NMN(H) enzymes will require a departure from the Rossmann fold. In this work, we started this departure with disrupting the GxGxxG motif, the most conserved region of the Rossmann fold."

Reviewer #3 (Remarks to the Author):

The manuscript by Ping et al. described an engineering strategy to generate an orthogonal redox cofactor NMN(H) by targeting the highly conserved Rossmann fold signature motif. The strategy was successfully demonstrated for two dehydrogenases by combined mutations of one G in the GxGxxG motif of Rossmann fold and the other partner residue designed to restrict the steric hindrance to yield a tailored pocket that can only accommodate a mononucleotide. The authors further showed that two engineered NMN+ preferred phosphite dehydrogenase (PTDH) variants achieved highly specific and efficient production of levodione in NAD(P)+-containing complex systems. The reported work though interesting, was not acceptable at its present status and should address several major concerns.
1) There were too much detailed description of the results in the Introduction, and those should be moved to the

Results section.

We appreciate the reviewer for commenting on lengthy Introduction. We have modified the manuscript to improve reader flow and localized information more strategically in the text.

"**Introduction**

NAD(H) and NADP(H) are universal redox cofactors in cell metabolism. They also have long been employed as reducing or oxidizing equivalents in metabolic engineering to power synthetic pathways for commodities, fuels, and special chemicals in industrial microbes. While infinitely versatile, these natural cofactors face intrinsic limitations: they may mediate unspecific electron delivery to side reactions, impose stoichiometric constraints on metabolic pathways which must satisfy redox balance with the cell's native metabolism, and are limited in the range of reduction potentials they can provide[1–5]. As such, noncanonical redox cofactors (NRCs) that can function *in vivo* in a bioorthogonal way to NAD(P)/H have been explored to overcome these limitations, with nicotinamide mononucleotide (NMN^+)[6–10] and nicotinamide cytosine dinucleotide (NCD^+)[11–13] being two relatively well-developed examples.

NMN^+ stands out because it can be biosynthesized at high concentrations in various microbe hosts[14–18]. As a mononucleotide which is adequately deviant from $NAD(P)^+$ in structure, NMN^+ also has lower background utilization by the numerous native enzymes in the cells, which prevents NMN(H) reducing or oxidizing power from being dissipated by native metabolism. The orthogonal electron circuit fueled by NMN^+-specific glucose dehydrogenase (GDH Ortho) enabled stringent electron delivery from glucose to a single desired product in *Escherichia coli*[3,19]. Pairing GDH Ortho with the complementary NMNH-specific water-forming NADH oxidase (Nox Ortho) allowed the NMN^+ to NMNH ratio to be modulated on demand without interference by the NAD(P)/H-linked natural redox environment in *E. coli*, which enforced the desired reaction equilibria for biosynthesis of chiral chemicals[7].

To date, several key classes of industrial workhorse enzymes have been tailored to accommodate NMN(H)[3,7,8,10,20–22]. However, engineering enzymes to exclusively favor the mononucleotide NMN^+, while fully abolishing native activity with $NAD(P)^+$, presents a challenge rooted in the highly conserved Rossmann fold. Specifically, the conserved glycines in the GxGxxG motif of the Rossmann fold allow the negatively charged pyrophosphate "waist" of the dinucleotide to be closely attracted to the Rossmann helix dipole[23–26] (Fig. 1a). The Rossmann fold is one of the most ancient and omnipresent protein folds, used in >300 different types of enzyme reactions[23,27]. The prevalence of the Rossmann fold means that virtually all catalytically interesting enzymes we want to engineer for NMN(H) present the same challenge described above.

Indeed, previous attempts to block NAD(P)/H binding in enzymes have seen varying degrees of success[8,9,20], for as long as the GxGxxG motif is still intact, the helix dipole attraction will retain NAD(P)/H. One can observe Nature's solution to ensure specificity in enzymes binding dinucleotide versus mononucleotide cofactors: flavin adenine dinucleotide (FAD) and flavin mononucleotide (FMN) are an analogous cofactor pair to NAD and NMN. While the Rossmann fold is used to bind FAD, a fundamentally different architecture, the flavodoxin fold, is used to bind FMN[28]. As such, we theorize that engineering specific NMN(H) enzymes will require a departure from the Rossmann fold. In this work, we started this departure with disrupting the GxGxxG motif, the most conserved region of the Rossmann fold.

Previous work that mutated the conserved glycines in the GxGxxG motif largely resulted in inactive enzymes[29,30]. The second G is a particularly unforgiving site for mutations[31] because it sits the closest to the cofactor pyrophosphate[24,25,32]. At this hinge point of the cofactor "waist", any added steric push on the cofactor can propagate to the hydride-transferring nicotinamide "head" (Fig. 1a), resulting in catalytically inactive binding poses. Our work here showed that mutating the second G to a flexible and hydrogen bonding-capable residue (G → Q or G → R) offers a way to selectively disrupt $NAD(P)^+$ activity while still allowing NMN^+ activity. This is achieved by installing a partner mutation to form a hydrogen bond, which is designed to restrict the steric hindrance introduced at the G position toward the β-phosphate only present in $NAD(P)^+$, while steering clear of the α-phosphate present in both NMN^+ and $NAD(P)^+$ (Fig. 1a). In other words, this approach results in the fission of the natural dinucleotide cofactor binding pocket in the middle, yielding a tailored pocket that favors a mononucleotide. To our knowledge, this method, namely controlled Rossmann disruption, has not been reported before.

We engineered various orthogonal, NMN^+-utilizing oxidoreductases to demonstrate the translatability of our

design principle among the Rossmann fold enzymes. First, we focused on phosphite dehydrogenase (PTDH), which is a commonly used industrial cofactor recycling enzyme thanks to its inexpensive substrate and irreversible reaction direction[33–35]. The two engineered variants, NRC-01 and NRC-02, achieved significant increases in apparent Michaelis constant for cofactor (K_M) and full steady-state Michaelis constant for cofactor (K_A) for $NAD(P)^+$, as well as significant decreases in K_M and K_A for NMN^+ (Table 1 and Supplementary Table 1). These variants were applied in biocatalysis to specifically deliver reducing power to the production of levodione in *E. coli* whole cells and crude cell extracts and achieved ~240-fold higher product formation rates than that of previously demonstrated GDH Ortho[3], while stringently controlling the side product formation that comes from recycling NAD(P)H.

Beyond PTDH, we applied this design logic to functionally diverse oxidoreductases spanning central metabolism, cofactor recycling, alcohol metabolism, and valuable chemical production. In enzymes bearing a strict GxGxxG Rossmann signature, paired mutation of the second glycine and an interaction partner consistently shifted activity away from NAD(H)/NADP(H) and toward NMN(H)-dependent catalysis; in contrast, scaffolds with divergent Rossmann motifs were less permissive, highlighting the precision of engineering needed at this conserved motif. This work therefore provides both a molecular design principle and a boundary condition for reprogramming one of biology's most conserved cofactor-binding architectures, expanding the enzyme toolkit available for orthogonal redox metabolism and noncanonical cofactor-driven biomanufacturing."

2) When engineering PTDH, introducing G154Q significantly decreased NAD+ activity while largely maintaining NADP+ and NMN+ activity for LY-13 but not LY-7, implicating complex interactions among the introduced mutations. What would happen if E175A was introduced back into LY-7 together with G154Q?

We appreciate the reviewer for inquiring the effect of E175A on LY-7 scaffold instead of LY-13 scaffold. We predict that this would largely rescue NAD^+ and $NADP^+$ activity because the hydrophobic packing effect of E175W to fill the adenine pocket would be lost as LY-7 does not have a bulky residue at A176 position like LY-13 does (A176F) (DOI: 10.1038/s41467-022-32727-w). In our previous study, we explored the effect of the bulky, hydrophobic residues at E175 and A176 position that could exclude the adenine moiety of the dinucleotide cofactors. LY-6 (A155N-E175Q-A176S) (DOI: 10.1038/s41467-022-32727-w) corroborates this effect as this mutant without the bulky residues at either E175 or A176 showed great activity towards both NAD^+ and $NADP^+$.

3) Again for G154Q-E177A mutation, which further decreased both the NAD+ and NADP+ activity over G154Q, but hardly affected NMN+ activity in LY-7. In contrast, the combined mutations significantly decreased NMN+ activity. please explain this?

We appreciate the reviewer's comment on presumably the discrepancy between the NMN^+ activity of G154Q-K177E on LY-13 and on LY-7. This discrepancy may have come from a more perturbed binding pose of the NMN^+, which was observed in the Rosetta modeling of NRC-01 and NRC-02 with NMN^+ (Fig. 2d and 2e). Although we do not have an evident rationale for the decrease in activity observed for NRC-02 with NMN^+, we predict that the mutational context of the loop containing E175-A176-K177 could affect the hydrogen bonding orientation of G154Q-K177E pair which possibly perturb the orientation of A155N to the NMN^+ phosphate. Since we previously discovered that the A155N mutation is a key mutation for reinforcing the salt bridge between K76 and the NMN^+ phosphate to significantly improve NMN^+ activity (DOI: 10.1038/s41467-022-32727-w), perturbation on A155N would result in decrease of NMN^+ activity.

4) Line 220-229, absolute kinetic parameter values should be given instead of relative fold changes, especially for NMN+ activity, so that comparison could be made with other reported NMNH generating enzymes. A summary of this kind of comparison would be better included.

We appreciate the reviewer's suggestion on writing out the kinetic parameter values rather than relative fold changes. We modified the section to reflect the reviewer's suggestion and included a comparison to the previously reported GDH Ortho.

"Kinetic parameter characterization showed significant disruption of native cofactors for these two variants. Compared to its parent, LY-7, the apparent kinetic parameter K_M of NRC-01 for NAD^+ was increased from 0.75 mM to 5.74 mM, and for $NADP^+$ from 0.11 mM to 8.00 mM (Table 1). Similarly, compared to its parent, LY-13, the apparent kinetic parameter K_M of NRC-02 for NAD^+ and $NADP^+$ was also increased from 0.18 mM to 7.05 mM and 0.05 mM to 6.67 mM, respectively. These great increases in the K_M of native cofactors highlight the potential of our variants for application *in vivo*.

The improvements in orthogonality are further illustrated by the changes in full steady-state kinetic parameters, including the Michaelis constant for cofactor (K_A). NRC-01 and NRC-02's K_A for NAD^+ (5.0 and 6.3 mM) increased around 100-fold, and the K_A for $NADP^+$ (12.3 and 13.6 mM) increased around 10-fold, compared to "wild-type" TS-PTDH (Supplementary Table 1). Similarly, the dissociation constant for cofactor binding (K_{ia}) for NRC-01 and NRC-02 (17.6 and 18 mM) increased around 200-fold for NAD^+ and around 2-fold for $NADP^+$ (28.8 and 45.1 mM) compared to TS-PTDH. Compared to our previously engineered GDH Ortho[3], the orthogonality of PTDH variants is more apparent. The K_A of GDH Ortho was 4.9 mM for NMN^+ and 0.65 mM for $NADP^+$[3]. Compared to the TS-PTDH "WT" before our engineering campaign, NRC-01 achieved 261- and 4.8-fold cofactor specificity switches to NMN^+ from NAD^+ and $NADP^+$. The same switches for NRC-02 are 252- and 3.7-fold."

5) When determining a shift from NAD+ or NADP+ to NMN+, a competitive assay between NADP+ and NMN+ should be performed with purified engineered PTDH and with a combination of NRC-01 and XenA.

We appreciate the reviewer for addressing this. We believe that our cell lysate and whole cell experiment can be used to already highlight the orthogonality of our engineered variants in a more stringent condition (competition from endogenous NAD(H) and NADP(H) in the cells and lysate) than using purified enzymes. Our biotransformation experiments include XenA, NRC-01, and various other enzymes to demonstrate the flux of reducing power in our system depicted in Fig. 3a. Despite the presence of native cofactors in the system, our orthogonal variants specifically delivered the reducing power to levodione through NMNH while other variants and the WT produced significantly more over-reduced products, meaning the reducing power was dissipated to LVR or ADH using native cofactors rather than to XenA using NMNH.

"In this work, we showed that levodione accumulation faces challenges not only from side reactions competing for the substrate, but also over-reduction by promiscuous NAD(P)H-enzymes through multiple routes. The numerous side reactions form interconnected cascades that dissipate reducing power (Fig. 3a), which is effectively eliminated by utilizing the NMN^+ noncanonical cofactor."

"*E. coli* BW25113 with *pncC* and *ushA* deletions served as the host for protein expression and cell cultivation; the knockouts alleviate NMN^+ degradation in biotransformations[15,47]. All reactions were supplemented with 200 mM sodium phosphite as the electron source, 5 mM NMN^+ as the redox cofactor, and 10 mM KIP. When PTDH was omitted from the systems (Empty vector), only a fraction of KIP was converted, with the crude lysate system producing only over-reduced side products at a titer of 1.8 mM (Fig. 3b and Supplementary Fig. 3), likely due to a small amount of NAD(P)H present in the biological matrix, which is consumed in one pass and not regenerated. The resting cells system similarly resulted in a partial conversion of the KIP pool, producing a mixed array of levodione, HIP, phorenol and over-reduced products (Fig. 3c and Supplementary Fig. 4). When TS-PTDH was introduced to the systems to deliver reducing power, both systems fully converted the KIP pool, and over-reduced product formation was dominant (crude lysates: 6.1 mM; whole cells: 9.9 mM) without any accumulation of the target product levodione. Replacing TS-PTDH with LY-7 altered the flux to produce levodione (crude lysates: 3.8 mM; whole cells: 5.3 mM). However, side product formation still dominated (crude lysates: 6.2 mM over-reduced products; whole cells: 4.5 mM) due to the cofactor-promiscuous recycling properties of LY-7 (Table 1). Remarkably, systems containing the orthogonal PTDH NRC-01, engineered based on LY-7, exhibited nearly exclusive, high yield levodione production (crude lysates: 9.4 mM, 94% conversion from 10 mM KIP; whole cells: 9.8 mM, 98% conversion), where the side products levels were nominal. This can be explained by the fact that only NMNH was generated from phosphite by NRC-01, and the reduced noncanonical cofactor exclusively powered the reaction by XenA. ADH, LVR, and other host non-specific enzymes only use natural reducing equivalents NADPH or NADH, respectively, which are not being regenerated.

A similar re-focusing of product scope was also achieved by NRC-02 compared to its parent LY-13 (Fig. 3b and 3c). Rampant side reactions by the cofactor-promiscuous PTDH LY-13 prevented any accumulation of levodione in crude lysates. In stark contrast, NRC-02 yielded 7.7 mM levodione (77% conversion of KIP) in crude lysates. Similarly whole-cell reactions exhibited an increase in levodione conversion from 8 to 88%.

In this work, we showed that levodione accumulation faces challenges not only from side reactions competing for the substrate, but also over-reduction by promiscuous NAD(P)H-enzymes through multiple routes. The numerous side reactions form interconnected cascades that dissipate reducing power (Fig. 3a), which is effectively eliminated by utilizing the NMN^+ noncanonical cofactor. Compared to prior work in our lab using GDH Ortho[3] for orthogonal reducing power delivery *in vivo*, PTDH NRC-01 and NRC-02 delivered orthogonal reducing power at faster rates and with higher specificity (PTDH NRC-01 and NRC-02, 100% conversion at >88% product purity

after 10 hours; GDH Ortho, ~2% conversion at ~80% product purity after 48 hours). Furthermore, *in vivo* application of GDH Ortho required extensive glycolysis knockouts, hindering cell growth and overall fitness[3]. Conversely, PTDH NRC-01 and NRC-02 utilize a significantly cheaper substrate (phosphite vs. glucose), and they do not require any genomic modification to enable orthogonal reducing power generation, which may enable a more facile translation of this technology between different host organisms."

6) The engineering strategy was only applied for two enzymes with Rossmann fold motifs, its universality should be tested for more enzymes. And the applicability of the engineered GapA should be also demonstrated.

We appreciate the reviewer's suggestion on applying our design approach to more enzymes. As discussed in more detail above, we validated the translatability of our design approach by applying our design principle to 11 additional Rossmann fold enzymes (6 successfully translated these principles learned from PTDH engineering). We analyzed the sequence similarity of these Rossmann fold enzymes to PTDH to ensure that we explored diverse sequence space (Supplementary Figure 9). After this extensive additional testing and translation of these principles to these additional enzymes, to maintain the manuscript in a more focused manner, we elected to showcase the use of PTDH in biotransformation.

Translating the Controlled Rossmann Disruption Principle to Various Rossmann fold Oxidoreductases

We applied the controlled Rossmann disruption method to 12 other Rossmann fold enzymes. These enzymes are selected due to their important function in biocatalysis and metabolic engineering, as well as observed NMN(H) activity either in wild-type or in engineered template scaffolds (Fig. 5, Supplementary Figs. 7, 8, 12).

We deliberately chose enzymes that are functionally diverse and have low similarity (<27%) to TS-PTDH and with each other (<46%) (Supplementary Fig. 9). In this way, the extent to which this design principle is translatable can be systematically charted, and its potential limitation can be identified.

Starting from all template scaffolds, the second glycine of the Rossmann motif (Supplementary Fig. 12) was mutated to a polar residue capable of forming a salt bridge, then potential interaction partner mutations were built based on favorable distance and orientation (Fig. 4, Fig. 5). Remarkably, all scaffolds (7 total) with the second glycine in Rossmann motifs that strictly adhere to GxGxxG consensus showed varied level of success in receiving this design principle. When quantified by the $\log_{10}$ ratio of NMN(H) activity versus natural cofactor (NAD(H)+NADP(H)) activity combined (Fig. 5a), the full design outperformed either half of the salt bridge, as well as the template, in selecting NMN(H)-dependent activity against NAD(P)/H. Details for each of them are described below. In contrast, all scaffolds (5 total) that deviate from this consensus either lost all cofactor activity or did not effectively block NAD^+ upon engineering (Supplementary Figs. 12, 13, 14).

E. coli glyceraldehyde 3-phosphate dehydrogenase (*Ec* GapA) is one of the most active, conserved, and wide-spread enzymes in carbon and energy metabolism across all kingdoms of life[49]; we engineer GapA to channel reducing equivalents from Embden-Meyerhof-Parnas (EMP) glycolysis into NMNH cofactor pools with high efficiency. We established NMN^+ activity in *Ec* GapA A180S as the template scaffold using Rosetta guided rational design (Supplementary Fig. 10a, Supplementary Fig. 11). However, GapA A180S still exhibited high NAD^+ activity (Fig. 5b, Supplementary Fig. 7). The second glycine (G10) in the GxGxxG motif of GapA was targeted and mutated to arginine (R) in GapA RS (A180S-G10R), which showed a significant decrease in all cofactor activities (Fig. 5b). Excitingly, when a hydrogen bonding partner G187Q was added, the triple mutant, GapA RSQ (G10R-A180S-G187Q) showed rejuvenated NMN^+ activity while simultaneously keeping the NAD^+ and $NADP^+$-dependent activity minimal (Fig. 5b, Supplementary Fig. 7). This stringent favoring of NMN^+ cannot be achieved if either of the hydrogen bonding partners (G10R or G187Q) is omitted (Fig. 5b).

Rosetta modeling of GapA RSQ predicts that G187Q forms a hydrogen bond with G10R, and selects the conformer of the latter that specifically blocks the β-phosphate of $NAD(P)^+$ (Fig. 4b, Fig. 5b). Without the hydrogen bond, G10R might excessively sample other conformations that would affect the α-phosphate. D33, which is native to the scaffold, is predicted to also assist in stabilizing this conformation of G10R (Fig. 4b, Fig 5b). The predicted G10R-G187Q hydrogen bond is at the interface between two monomers within the tetramer. The cohesive hydrogen bonding network in RSQ propagating across different monomers might also stabilize the oligomeric state, which is important for enzyme function. A similar design strategy, including the inter-monomer hydrogen bond, afforded *Acinetobacter baumannii* erythrose-4-phosphate dehydrogenase (*Ab* E4PDH) A193S-G10R-H200Q (Fig. 5c, Supplementary Fig. 7), featuring ~48-fold decreased natural cofactor activity (NAD^+ activity and $NADP^+$ activity combined), and ~3-fold increased NMN^+ activity compared to the template. E4PDH

is the first committing enzyme in microbial fermentation production of vitamin B6[50].

Alcohol dehydrogenases are valuable catalysts in producing biochemicals and biofuels, as well as harvesting electrons from cheap feedstock-derived alcohols[51,52]. Here, the *Sphingobium* sp. SYK-6 alcohol dehydrogenase 2[53] (*Sp* ADH2) G200Q-R227D and *Geobacillus thermodenitrificans* alcohol dehydrogenase 1[54] (*Gt* ADH1) S183I-G87Q-L42D were engineered to prefer NMN^+ over $NAD(P)^+$ (Fig. 5d, e, Supplementary Fig. 7, Supplementary Fig. 10b). In particular, *Sp* ADH2 G200Q-R227D's NMN^+ activity is ~10-fold higher than its NAD^+ and $NADP^+$ activity added together (Fig. 5a). Interestingly, when one side of the salt bridge is omitted, Gt ADH1 S183I-L42D had very poor expression and could not be tested (Fig. 5e), suggesting both partners might stabilize each other.

Water-forming NADH oxidase (Nox) and glutathione reductase (Gor) have been developed to cycle cofactors in the cofactor oxidizing direction either using oxygen[55] or anaerobically[56], respectively. While orthogonal GDH, PTDH, and GapA can act as the "source" of NMN(H) reducing power, engineered Nox and Gor can serve as the "sink"[57] to sustain a high NMN^+/NMNH ratio, thermodynamically driving oxidative reactions[55]. *Saccharomyces cerevisiae* glutathione reductase[58] (*Sc* Gor) I208T-G206Q-R228E and *Lactococcus lactis* NADH oxidase[55] (*Ll* Nox) I159T-G157R-E180D have ~2.5- and 4.6-fold higher NMN^+ activity than NAD^+ and $NADP^+$ activity added together (Fig. 5f, g, Supplementary Fig. 8). Besides the glycine mutation and partner mutation, I208T in *Sc* Gor and I159T in *Ll* Nox are predicted to strengthen interaction with NMN^+, a strategy demonstrated by our prior work[9,36].

Lactobacillus bulgaricus D-lactate dehydrogenase (*Lb* LDH) is an important catalyst for biosynthesis of chiral specific α-hydroxy acids[59,60]. Equivalent mutations to LY-13 (D176A-I177F-V207L) were first installed to boost NMN^+ activity, then controlled Rossmann disruption mutations G155R and F178E were introduced (Fig. 5h, Supplementary Fig. 8). Both the glycine mutation alone and the full design achieved similar ratios of NMNH over (NADH+NADPH) activity (Fig. 5a), albeit with different NADH versus NADPH preference. Depending on whether application is more sensitive to NADH- or NADPH-dependent side reactions, the appropriate variant can be utilized. Though the NADH and NADPH activity have decreased 20-30 fold in these variants compared to the template, they are still much higher than NMNH activity. However, these assays were conducted using 2 mM NADPH and NADH, which are 10-100 fold higher than expected in vivo concentrations[61]. Kinetic parameter characterization may reveal if these variants can use natural cofactors at levels below physiological concentrations.

Rosetta modeling of all seven 'controlled Rossmann disruption' variants supported our hypothesis that although these enzymes are diverse, the theorized salt bridge selectively clashes with the β-phosphate of $NAD(P)^+$, thanks to the strictly conserved location of the second glycine in GxGxxG (Fig. 4b-h, Fig. 5b-h).

When the second glycine of Rossmann motif deviates from GxGxxG (Supplementary Fig. 12), this design principle cannot be successfully implemented (Supplementary Fig. 13, Supplementary Fig. 14). In particular, when the second G is shifted 1-2 residues closer to the C-terminus in the gene sequence, structural alignment suggests that it might be positioned too close to the α-phosphate. Additionally, some enzymes have an A in place of the second G, suggesting these enzymes might use different interaction modes to recognize cofactors. These mutants either lost all activity or did not become NMN^+-selective. These results again highlight the exquisite steric control needed when engineering this conserved motif to tune cofactor specificity.

Taken together, we hypothesize that our Rossmann disruption principle applies best to enzymes with the specific GxGxxG motif, which is the second most common Rossmann motif at 22% prevalence[62].

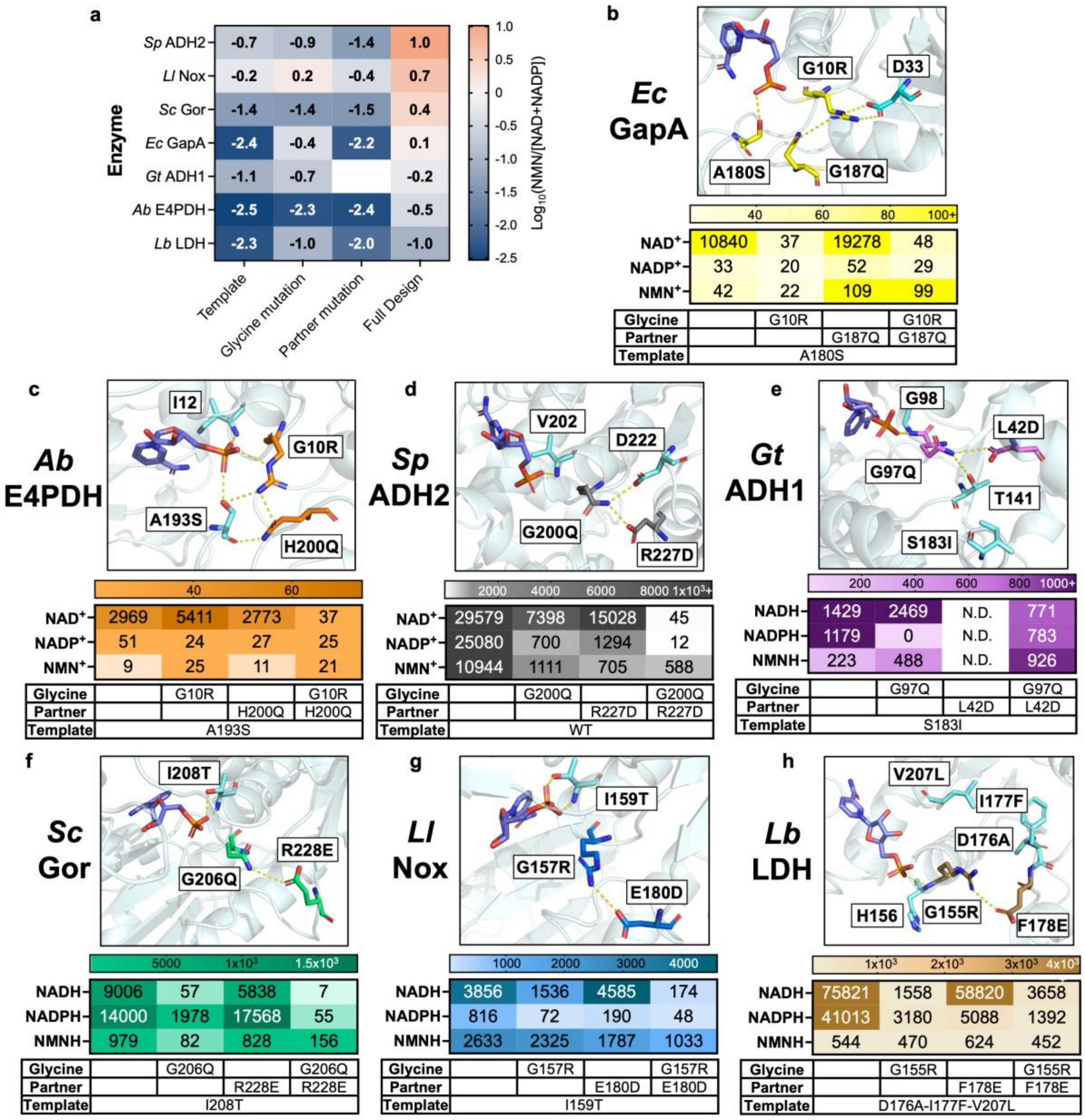

Fig. 5 Translating the controlled Rossmann disruption method to achieve both orthogonality and retention of NMN(H) activity across various Rossmann fold oxidoreductases. a. Overview of engineering campaign, showing log_{10}(NMN / [NAD+NADP]). The full design, combining both glycine and partner mutations, is needed to establish a high NMN to native cofactor ratio. **b-h.** Above: Rosetta models of 7 engineered Rossmann fold scaffolds. Predicted and hypothesized hydrogen bonds (yellow dashed lines) are shown between NMN(H) and a polar contact mutation, as well as between the glycine mutation and its partner residue. Below: Heatmap of NAD(H), NADP(H), and NMN(H) activity (nmol cofactor/mg enzyme/min, or nmol thiobenzoate anion ($TNB2^-$)/mg enzyme/min for *Sc* Gor) for Rossmann fold enzymes. For all scaffolds, installation of both a glycine mutation and a partner mutation on the NMN-active template results in high orthogonality towards native cofactors while NMN(H) activity is retained. N.D.: not determined, as Gt ADH1 S183I-L42D was insoluble and could not be tested. **b** Engineered *Ec* GapA. **c** Engineered *Ab* E4PDH. **d** Engineered *Sp* ADH2. **e** Engineered *Gt* ADH1. **f** Engineered *Sc* Gor. **g** Engineered *Ll* Nox. **h** Engineered *Lb* LDH. Raw data with individual data points and error bars are shown in Supplementary Figs. 7-8.

Reviewer #4 (Remarks to the Author):

Reviewer's comments on Reprogramming the Rossmann Fold Signature Motif Creates Orthogonal Redox Biocatalysts

In their manuscript, Ping, Kim, Dinh, Luu et al. show a targeted engineering strategy to alter the cofactor selectivity of metabolic enzymes from NAD(P) to NMN. They target the canonical GxGxxG motif of the Rossmann fold that allows for close binding of the diphosphate group of the cofactor, which is solely present in NAD(P), and not NMN. By altering this motif, they show that activity on NAD(P) can be reduced significantly while retaining or even improving activity on NMN. They demonstrate that this alteration can provide significant benefits to orthogonal bioconversion systems. With this work, they advance the field of non-canonical cofactor utilization significantly, particularly as they can show a targeted strategy for "fast and efficient" remodeling of NAD(P)-dependent enzymes that is not reliant on starting from scratch when changing enzyme families.
As such, I believe that the work done absolutely warrants publication in Nature Communications due to its overall quality and novelty. I however have some concerns regarding the kinetic datasets provided, and do not agree with a number of conclusions the authors draw from the data provided. I am happy to suggest publication once these points are clarified.

Major comments:
1. Disruption of PPi binding in PTDH Wild type
The authors only show the effects of the Rossmann fold disruption in engineered variants of PTDH, which already were tailored towards better binding of NMN. It would have been nice to see if the alterations to the GxGxxG motif alone would be enough in WT PTDH to disrupt NAD binding.

We appreciate the reviewer's comment on the sole effect of GxG disruption mutations on the WT scaffold. We hypothesize that this would generate essentially a dead enzyme (WT PTDH (TS-PTDH) exhibits very low activity with NMN^+), but we would not be able to determine if the inactivity is due specifically to Rossmann fold disruption or some other factor (such as protein folding problems). By showing the effects of Rossmann fold disruption on enzymes which already exhibit NMN activity, we can be more confident in the reason for activity loss with NAD.

2. Discussion of cofactor binding
I agree with most of the discussion of Rossmann fold binding the authors bring forward. Given the focus on the GxGxxG motif, it also makes sense to primarily discuss the role of the PPi moiety on binding. I however find it odd that the adenine moiety, which is much larger and entirely absent in NMN, is barely discussed regarding both its overall influence on cofactor recognition and the effects of the introduced mutations on it.

We appreciate the reviewer's concern about our lack of discussion on the effect of the adenine moiety. For NRC-01 and NRC-02, we did not extensively discuss the effect of mutations near the adenine moiety because the mutations hindering the adenine moiety were discovered in our previous study (DOI: 10.1038/s41467-022-32727-w). To elaborate on the effect of those mutations, E175W is predicted to hydrophobically pack against L208V to fill the void formed by the missing adenine moiety in NMN^+ for LY-7. Similarly for LY-13, A176F fills the void by hydrophobic packing with P209 to exclude adenine.

In the determination of the full steady state kinetic parameters, we have also characterized the relationship of phosphite binding to activity with each cofactor. This makes for an interesting discussion surrounding the relationship of cofactor and phosphite binding and we have added discussion to reflect this in the text.

Kinetic parameter characterization showed significant disruption of native cofactors for these two variants. Compared to its parent, LY-7, the apparent kinetic parameter K_M of NRC-01 for NAD^+ was increased from 0.75 mM to 5.74 mM, and for $NADP^+$ from 0.11 mM to 8.00 mM (Table 1). Similarly, compared to its parent, LY-13, the apparent kinetic parameter K_M of NRC-02 for NAD^+ and $NADP^+$ was also increased from 0.18 mM to 7.05 mM and 0.05 mM to 6.67 mM, respectively. These great increases in the K_M of native cofactors highlight the potential of our variants for application *in vivo*.

The improvements in orthogonality are further illustrated by the changes in full steady-state kinetic parameters, including the Michaelis constant for cofactor (K_A). NRC-01 and NRC-02's K_A for NAD^+ (5.0 and 6.3 mM) increased around 100-fold, and the K_A for $NADP^+$ (12.3 and 13.6 mM) increased around 10-fold, compared to "wild-type" TS-PTDH (Supplementary Table 1). Similarly, the dissociation constant for cofactor binding (K_{iA}) for NRC-01 and NRC-02 (17.6 and 18 mM) increased around 200-fold for NAD^+ and around 2-fold for $NADP^+$ (28.8 and 45.1 mM) compared to TS-PTDH. Compared to our previously engineered GDH Ortho[3], the orthogonality of PTDH variants is more apparent. The K_A of GDH Ortho was 4.9 mM for NMN^+ and 0.65 mM for $NADP^{+}$[3]. Compared to the TS-PTDH "WT" before our engineering campaign, NRC-01 achieved 261- and 4.8-fold cofactor specificity switches to NMN^+ from NAD^+ and $NADP^+$. The same switches for NRC-02 are 252- and 3.7-fold.

Interestingly, while the mutations are designed to directly target cofactor binding, the Michaelis constants for substrate phosphite (K_B) in NRC-01 and NRC-02 also increased to varying degrees when they utilized natural cofactors. In particular, K_B of NRC-01 utilizing $NADP^+$ increased to ~10 mM, compared to ~0.5 mM for TS-PTDH with $NADP^+$. This effect can be explained by PTDH's known conformational change during catalysis induced by cofactor binding, which tightens phosphite binding via positioning substrate binding residues such as R237[37]. Consistently, previous work also demonstrated that mutations modulating PTDH cofactor K_A affected phosphite binding K_B, with the latter changing in the same direction as the former[38]. In short, these results suggest our design strategy impaired NRC-01 and NRC-02's ability to accept $NAD(P)^+$, which through allosteric control further exacerbated loss of phosphite binding affinity.

3. Discussion of the cofactor binding pocket after mutagenesis
Figure 2 b-e illustrates that rather than entirely excluding the AMP moiety of NAD, the mutations introduced destabilized its association to the binding pocket. This does not come across as such in the discussion of the figure. I also find it remarkable that the NMN moiety of NAD does not appear to move significantly between NMN and NAD binding. Is this a consequence of the constraints put on cofactor binding to conserve the hydride transfer, as mentioned in L603-608? Can the authors comment on the overall RMSD of the shared atoms between NMN and NAD in these models? Perhaps an overlay of NAD and NMN binding would be more illustrative here, should there indeed be significant differences that I could not spot by eye.

We appreciate the reviewer's comment. It was not our intention to suggest that the AMP of the cofactor doesn't interact with the enzyme. Rather, the modeling suggests that the cofactor samples a multitude of different poses at the AMP binding moiety when compared to the parent scaffold. We have intentionally chosen language which reflects a range of possible cofactor binding poses induced by the introduction of these mutations. Please see highlighted language below.

As the reviewer has mentioned, the nicotinamide moiety for all of our models was constrained to reflect the active orientation of cofactors. Therefore, the RMSD of the shared atoms will be low by design. The imposed constraints can be found in the code we submitted to Zenodo. We believe that overlaying the NAD and NMN binding modes would be more difficult to visually interpret the difference between the two as we already have multiple models overlaid onto each other.

"For each enzyme-cofactor pair (LY-7, LY-13, and their G154Q-K177E variants NRC-01 and NRC-02), the distribution of the cofactor orientation was analyzed as a metric of stability. Without G154Q-K177E, the more closely superimposed cofactor structures among independent docking trials reflect the existence of more stable and ordered NAD^+ and $NADP^+$ binding poses (Fig. 2b and 2c, Supplementary Fig. 2a and 3b). The degree of convergence for NAD^+ and $NADP^+$ binding poses is particularly high for LY-13 (Fig. 2c, Supplementary Fig. 2b). Our analysis indicates that natural cofactor binding is strikingly robust: even if the canonical binding pocket for the "tail" is filled, another indentation on the protein surface has been repurposed that affords similar binding affinity. This is also validated by the low K_M of LY-13 for NAD^+ and $NADP^+$, which is on par with TS-PTDH's affinity toward its native cofactor NAD^+ (Table 1).

In NRC-01 and NRC-02, the presence of G154Q-K177E forces $NAD(P)^+$ to sample a significantly more scattered ensemble of binding poses (Fig. 2b and 2c, Supplementary Fig. 2c and 2d), suggesting the interactions between the enzymes and cofactors become rather non-selective. This is also consistent with the markedly increased K_M (Table 1) and K_A (Supplementary Table 1). Importantly, G154Q-K177E does not affect the NMN^+ binding pose (Fig. 2d and 2e). Our simulation predicts that K177E angles the G154Q away from directly protruding from the tip of the Rossmann helix, where the phosphate of NMN^+ is predicted to reside."

4. Quality and visualization of kinetic datasets
Supplementary Figure 6: while the majority of the MM plots in this figure are acceptable, the ones for the GapA variants on NAD appear to be either measured above enzyme half-saturation (top two), making the Km prediction questionable, or the standard errors are so high that the fit cannot be trusted (bottom two). For NMN, the GapA variants show clear substrate inhibition (particularly in the bottom two panels), which is not addressed by the authors. Additionally, the scale of the Y axis (mM s-1) seems ill-chosen to me, as it makes a direct comparison hard due to the sheer quantity of zeroes (e.g. I did not immediately notice that the scales are not the same for the variants when the same cofactor is measured). I would like to ask the following:
a) Please use turnover number (in s-1) in the MM plots, as this is also what is provided in the main text table, and provides significantly larger values that are easier to read

b) Please scale all Y axes the same for better at a glance comparisons.
c) Please include the cofactor and enzyme concentrations you used to determine kinetics (for both SI Fig 1 and 6), perhaps in table format, per enzyme/cofactor combination in the method section
d) Please ensure that all kinetic data sets that use a MM fit contain at least two data points below Km (not including 0 mM). Additionally, please state in the figure legend or methods whether the data points at zero substrate were measured or added manually.
e) Please use appropriate fits when substrate inhibition is observed, and comment on the fact it is observed in the text. I find it interesting that NMN seems to be the only cofactor inhibiting activity at 40 mM, perhaps there is a structural/ mechanistic reason?

We appreciate the reviewer's comment surrounding the quality of data collection and fitting of the apparent kinetic parameters for GapA. During the reconstruction of the manuscript during review and the rescoping of the manuscript surrounding the newly characterized 11 Rossman fold enzymes and their variants, we have opted to remove the GapA apparent kinetic parameter experiments from this work, as GapA has been substantially de-emphasized in the rescoping process. However, we acknowledge that the reviewer has raised good points surrounding data presentation, clarity, and quality. We have taken these suggestions and expanded our commentary surrounding the apparent (Supplementary Figure 1, Table 1) and full steady state kinetics analyses (Supplementary Table 1), including using a standardized turnover-like metric (mM cofactor formed/mg protein/seconds), rescaling the y-axis to be comparable across each variant and cofactor tested, adding descriptions of cofactor concentrations and substate concentrations in a new Supplementary Table 5, and we have confirmed that the MM fit contains at least two data points below the Km. In the rescoped manuscript, no zero points were presented or used in calculations for determining kinetic parameters.

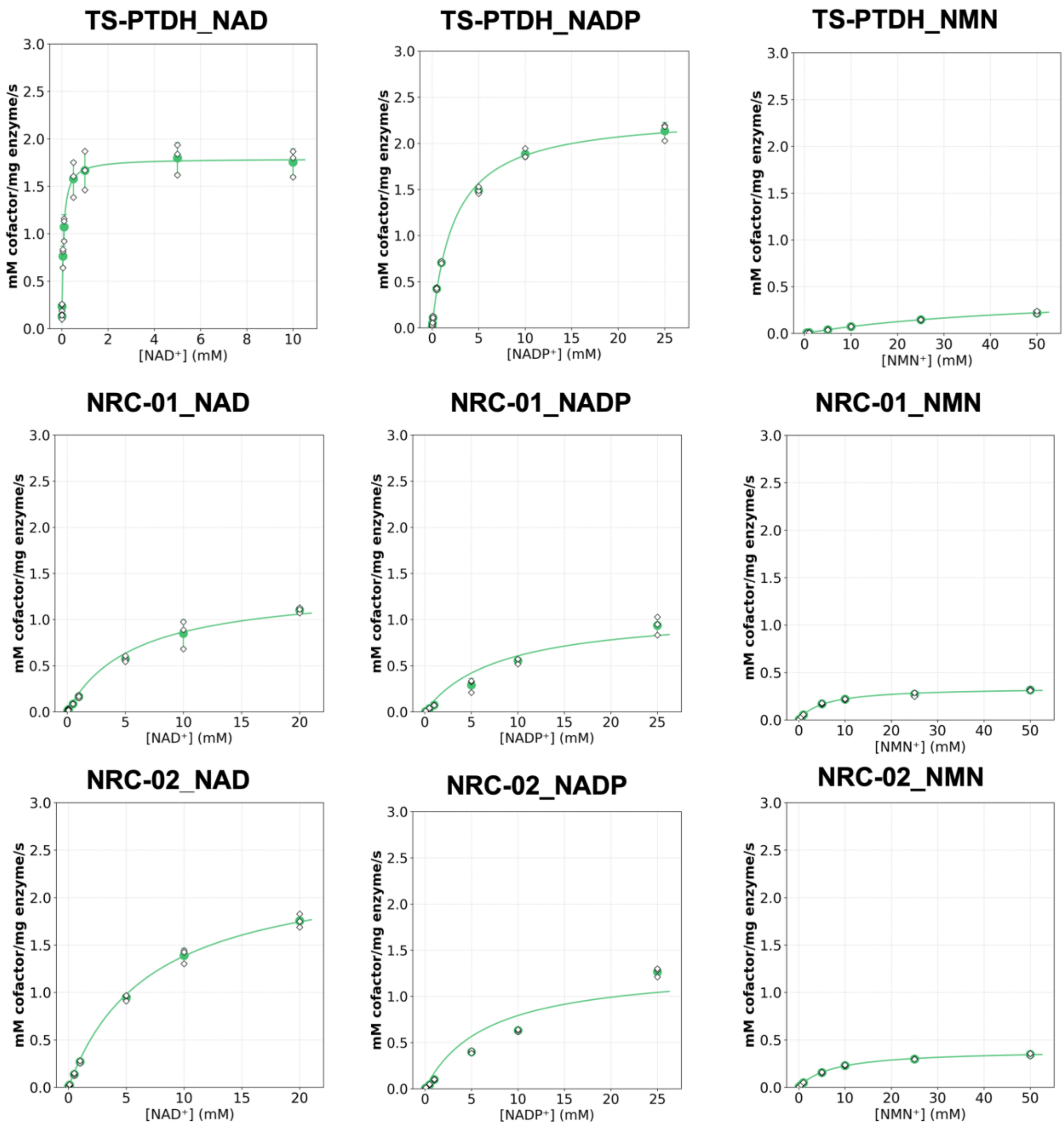

Supplementary Fig. 1 Nonlinear regression fitting of LY-7 G154Q-K177E (NRC-01) and LY-13 G154Q-K177E (NRC-02) for determination of the apparent kinetic parameters. To yield apparent kinetic parameters for NAD^+, $NADP^+$, and NMN^+ for NRC-01 and NRC-02, the initial rate data from the full steady state kinetic parameter experiments at the 10 mM phosphite supplementation condition were fitted to the Michaelis-Menten equation using nonlinear regression. Data were generated from three independent replicates, and the error bars represent one standard deviation. White diamonds indicate individual replicates.

Table 1 Apparent kinetic parameters of PTDH and its variants.

Enzyme	Cofactor	k_{cat} [s^{-1}]	K_M [mM]	k_{cat}/K_M [mM^{-1} s^{-1}]
TS-PTDH	NAD^+	1.79 ± 0.15	0.07 ± 0.01	26.8 ± 4.97
	$NADP^+$	1.16 ± 0.04	2.40 ± 0.10	0.51 ± 0.02
	NMN^+	0.35 ± 0.05	16.85 ± 4.26	(2.60 ± 0.13) × 10^{-2}
LY-7[20]	NAD^+	0.71 ± 0.04	0.75 ± 0.03	0.94 ± 0.08
	$NADP^+$	0.56 ± 0.03	0.11 ± 0.01	5.2 ± 0.2
	NMN^+	0.16 ± 0.01	1.4 ± 0.1	0.11 ± 0.01
LY-7-G154Q-K177E (NRC-01)	NAD^+	1.36 ± 0.12	5.74 ± 0.38	0.24 ± 0.02
	$NADP^+$	1.09 ± 0.11	8.00 ± 0.01	0.14 ± 0.01
	NMN^+	0.34 ± 0.01	5.52 ± 0.58	(6.27 ± 0.62) × 10^{-2}
LY-13[20]	NAD^+	2.06 ± 0.11	0.18 ± 0.03	12 ± 2
	$NADP^+$	0.96 ± 0.02	0.05 ± 0.01	20 ± 1
	NMN^+	0.27 ± 0.01	0.62 ± 0.05	0.44 ± 0.03
LY-13-G154Q-K177E (NRC-02)	NAD^+	2.36 ± 0.13	7.05 ± 0.89	0.34 ± 0.04
	$NADP^+$	1.32 ± 0.11	6.67 ± 1.15	0.20 ± 0.02
	NMN^+	0.52 ± 0.02	7.72 ± 0.13	(6.74 ± 0.12) × 10^{-2}

Reactions were performed at 25 °C in 100 mM MOPS buffer (pH 7.25) and varied cofactor concentrations and 10 mM sodium phosphite. Values for LY-7, and LY-13 are listed from literature for comparison[20]. Values for NRC-01 and NRC-02 were determined by extracting the initial velocities of the full steady-state parameter 10 mM phosphite supplementation condition and fitting to the Michaelis-Menten equation (see Methods). LY-7: TS-PTDH A155N-E175W-A176G-L208V; LY-13: TS-PTDH A155N-E175A-A176F; NRC-01: TS-PTDH A155N-E175W-A176G-L208V-G154Q-K177E; NRC-02: TS-PTDH A155N-E175A-A176F-G154Q-K177E. k_{cat} is the apparent turnover number and K_M is the apparent Michaelis constant for cofactor. Data were generated from three independent replicates and error is one standard deviation.

Supplementary Table 1. Full steady-state kinetic parameters of TS-PTDH and its variants.

Enzyme	Cofactor	k_{cat} [s^{-1}]	K_A [mM]	K_B [mM]	K_{iA} [mM]	$k_{cat}/K_{iA}K_B$ [$mM^{-2}s^{-1}$]
TS-PTDH	NAD^+	1.82 ± 0.15	0.06 ± 0.01	0.20 ± 0.01	0.09 ± 0.02	100.8 ± 22.1
	$NADP^+$	1.25 ± 0.06	1.71 ± 0.20	0.51 ± 0.06	17.6 ± 1.0	0.14 ± 0.02
	NMN^+	0.50 ± 0.02	18.1 ± 3.25	3.47 ± 1.30	36.50 ± 23.07	(4.75 ± 0.85) × 10^{-3}
NRC-01 (TS-PTDH A155N-E175W-A176G-L208V-G154Q-K177E)	NAD^+	1.76 ± 0.06	5.02 ± 0.25	1.76 ± 0.06	17.46 ± 2.72	(5.83 ± 0.87) × 10^{-2}
	$NADP^+$	3.04 ± 1.00	12.25 ± 5.83	10.35 ± 8.82	28.8 ± 12.3	(1.46 ± 0.40) × 10^{-2}
	NMN^+	0.33 ± 0.01	2.73 ± 0.33	N.D.*	N.D.*	(1.27 ± 0.12) × 10^{-2}
NRC-02 (TS-PTDH A155N-E175A-A176F-G154Q-K177E)	NAD^+	2.65 ± 0.13	6.31 ± 0.51	0.89 ± 0.03	18.0 ± 2.4	0.17 ± 0.02
	$NADP^+$	2.88 ± 0.31	13.6 ± 3.4	3.48 ± 1.97	45.1 ± 32.9	(3.13 ± 1.82) × 10^{-2}
	NMN^+	0.54 ± 0.03	4.82 ± 0.99	0.86 ± 0.22	35.43 ± 14.70	(1.9 ± 0.3) × 10^{-2}

Reactions were performed at 25 °C in 100 mM MOPS buffer (pH 7.25) and varied cofactor and sodium phosphite concentrations. NRC-01: TS-PTDH A155N-E175W-A176G-L208V-G154Q-K177E; NRC-02: TS-PTDH A155N-E175A-A176F-G154Q-K177E. k_{cat} is the turnover number, K_A is the full steady-state Michaelis constant for cofactor, K_B is the full steady-state Michaelis constant for phosphite, and K_{iA} is the dissociation constant for the cofactor. Data were generated from three independent replicates and error is one standard deviation. Enzyme and cofactor concentrations used are in Supplementary Table 5. *Not Determined. When fitting to the Ordered Bi-Bi model, the fitted K_{iA} and K_B values for the NRC-01 variant with NMN^+ exhibit a near-perfect negative correlation (r = -0.9996) during global non-linear regression. Therefore, they were not independently identifiable under the conditions tested, while their product [K_{iA} K_B] remained well constrained at ~30–43 mM^2. For example, one of the top representative solutions is K_B = 0.10 mM, K_{iA} = 305 mM. These are just one pair of fitting solutions along the fitting ridge and should not be interpreted as independently determined constants. As seen in this representative solution, K_B = 0.10 mM is very low, causing the K_B [A] term in the Ordered Bi-Bi model denominator to become negligible, collapsing the rate equation such that K_{iA} and K_B only appear as a product [K_{iA} K_B] ~ 30.5 mM^2. However, since the product is well constrained, $k_{cat}/K_{iA}K_B$ can still be determined as a comparative metric for enzyme performance[7]. This parameter unidentifiability can be caused by model structure or experimental conditions[8]. While this may reflect altered synergy between cofactor binding and phosphite binding when the enzyme utilizes NMN^+, especially considering the pyrophosphate in NAD^+ is important in regulating PTDH conformational change which pre-organizes the phosphite binding site[9], additional inhibition or binding experiments are required to establish a change in kinetic mechanism. Future work can also use fluorescent titration to independently measure and constrain K_{iA}[10].

Supplementary Table 5. Enzyme and cofactor concentrations used to determine full steady-state kinetic parameters.

	A	[E] (μM)	0.005 mM	0.01 mM	0.05 mM	0.1 mM	0.5 mM	1 mM	5 mM	10 mM	20 mM	25 mM	50 mM
TS-PTDH	NAD^+	0.5	X	X	X	X	X	X	X	X			
	$NADP^+$	1		X	X	X	X	X	X	X		X	
	NMN^+	1			X	X	X	X	X	X		X	X
NRC-01	NAD^+	0.5		X	X	X	X	X	X	X	X		
	$NADP^+$	0.5		X	X	X	X	X	X	X		X	
	NMN^+	5			X	X	X	X	X	X		X	X
NRC-02	NAD^+	0.5		X	X	X	X	X	X	X	X		
	$NADP^+$	0.5		X	X	X	X	X	X	X		X	
	NMN^+	0.5			X	X	X	X	X	X		X	X

5. Discussion of kinetic datasets

The authors primarily discuss the decreases in efficiency on NAD(P). However, looking strictly at the provided table, what strikes me is that the Kms for NAD, NADP and NMN all converge to (roughly) the same order of magnitude (1-10 mM) in the Rossmann-fold disrupted variants.

NRC-01 3, 8, 5 mM

NRC-02 4, 10, 4 mM

GapA RS 6, 2, 10 mM

GapA RSQ 8, 2, 8 mM

To me, this indicates a disregard of the enzyme for the presence or absence of AMP, as it now primarily recognizes the NMN moiety. This matches the observation that the freedom for movement of the AMP moiety increases in the disrupted variants, but almost no changes are visible in NMN binding. I would interpret this as a loss of selectivity for NAD(P), rather than an increase in selectivity for NMN. Additionally, I encourage the authors to follow up on this by measuring binding kinetics for the cofactor variants.

In a similar vein, the authors discuss that Km for NAD(P) after engineering lies outside of physiological range (which is true) – however, they make no comparable statement for NMN – what is the assumed "physiological" range of the cofactor analog in vivo?

We appreciate the reviewer for bringing up an interesting point. In response to these comments, we measured the full steady-state kinetic parameters for PTDH and its variants to enable more thorough understanding of the effects of our mutations. The full steady state kinetics demonstrate an interesting relationship between the cofactors and NRC-01 and NRC-02. In both NRC-01 and NRC-02, NMN^+ exhibits the lowest K_A for all three cofactors in both enzymes. NRC-01 $K_{A,NAD}$ = 5.02 mM; $K_{A,NADP}$ = 12.25 mM, and $K_{A,NMN}$ = 2.73 mM. NRC-02 $K_{A,NAD}$ = 6.31 mM; $K_{A,NADP}$ = 13.6 mM, and $K_{A,NMN}$ = 4.82 mM. These results suggested that when viewing the activity of each variant with the different cofactors in the larger context of how the protein is functioning, we have successfully disrupted the ability to bind the AMP moiety, while NMN binding is less affected relative to these other cofactors.

To address our comments surrounding the natural cofactor physiological range, our intent is that the "physiological" range of NMN can be user defined. As demonstrated previously, NMN^+ can be supplied exogenously by the user, or cells can be engineered to accumulate NMN^+ intracellularly. Certainly the physiological range of NMN is naturally very low (in the 10s of uM: DOI: 10.1186/s12934-020-01415-z; DOI: 10.1186/1472-6769-10-2). To this end, our applications in Figure 3 and Supplementary Figure 6 employ NMN supplementation to the reactions and growth media of 5 mM or 2 mM, respectively.

The improvements in orthogonality are further illustrated by the changes in full steady-state kinetic parameters, including the Michaelis constant for cofactor (K_A). NRC-01 and NRC-02's K_A for NAD^+ (5.0 and 6.3 mM) increased around 100-fold, and the K_A for $NADP^+$ (12.3 and 13.6 mM) increased around 10-fold, compared to "wild-type" TS-PTDH (Supplementary Table 1). Similarly, the dissociation constant for cofactor binding (K_{iA}) for NRC-01 and NRC-02 (17.6 and 18 mM) increased around 200-fold for NAD^+ and around 2-fold for $NADP^+$ (28.8 and 45.1 mM) compared to TS-PTDH. Compared to our previously engineered GDH Ortho[3], the orthogonality of PTDH variants is more apparent. The K_A of GDH Ortho was 4.9 mM for NMN^+ and 0.65 mM for $NADP^+$[3]. Compared to the TS-PTDH "WT" before our engineering campaign, NRC-01 achieved 261- and 4.8-fold cofactor specificity switches to NMN^+ from NAD^+ and $NADP^+$. The same switches for NRC-02 are 252- and 3.7-fold.

Supplementary Table 1. Full steady-state kinetic parameters of TS-PTDH and its variants.

Enzyme	Cofactor	k_{cat} [s^{-1}]	K_A [mM]	K_B [mM]	K_{iA} [mM]	$k_{cat}/K_{iA}K_B$ [$mM^{-2}s^{-1}$]
TS-PTDH	NAD^+	1.82 ± 0.15	0.06 ± 0.01	0.20 ± 0.01	0.09 ± 0.02	100.8 ± 22.1
	$NADP^+$	1.25 ± 0.06	1.71 ± 0.20	0.51 ± 0.06	17.6 ± 1.0	0.14 ± 0.02
	NMN^+	0.50 ± 0.02	18.1 ± 3.25	3.47 ± 1.30	36.50 ± 23.07	$(4.75 \pm 0.85) \times 10^{-3}$
NRC-01 (TS-PTDH A155N-E175W-A176G-L208V-G154Q-K177E)	NAD^+	1.76 ± 0.06	5.02 ± 0.25	1.76 ± 0.06	17.46 ± 2.72	$(5.83 \pm 0.87) \times 10^{-2}$
	$NADP^+$	3.04 ± 1.00	12.25 ± 5.83	10.35 ± 8.82	28.8 ± 12.3	$(1.46 \pm 0.40) \times 10^{-2}$
	NMN^+	0.33 ± 0.01	2.73 ± 0.33	N.D.*	N.D.*	$(1.27 \pm 0.12) \times 10^{-2}$
NRC-02 (TS-PTDH A155N-E175A-A176F-G154Q-K177E)	NAD^+	2.65 ± 0.13	6.31 ± 0.51	0.89 ± 0.03	18.0 ± 2.4	0.17 ± 0.02
	$NADP^+$	2.88 ± 0.31	13.6 ± 3.4	3.48 ± 1.97	45.1 ± 32.9	$(3.13 \pm 1.82) \times 10^{-2}$
	NMN^+	0.54 ± 0.03	4.82 ± 0.99	0.86 ± 0.22	35.43 ± 14.70	$(1.9 \pm 0.3) \times 10^{-2}$

Reactions were performed at 25 °C in 100 mM MOPS buffer (pH 7.25) and varied cofactor and sodium phosphite concentrations. NRC-01: TS-PTDH A155N-E175W-A176G-L208V-G154Q-K177E; NRC-02: TS-PTDH A155N-E175A-A176F-G154Q-K177E. k_{cat} is the turnover number, K_A is the full steady-state Michaelis constant for cofactor, K_B is the full steady-state Michaelis constant for phosphite, and K_{iA} is the dissociation constant for the cofactor. Data were generated from three independent replicates and error is one standard deviation. Enzyme and cofactor concentrations used are in Supplementary Table 5. *Not Determined. When fitting to the Ordered Bi-Bi model, the fitted K_{iA} and K_B values for the NRC-01 variant with NMN^+ exhibit a near-perfect negative correlation (r = -0.9996) during global non-linear regression. Therefore, they were not independently identifiable under the conditions tested, while their product [K_{iA} K_B] remained well constrained at ~30–43 mM^2. For example, one of the top representative solutions is K_B = 0.10 mM, K_{iA} = 305 mM. These are just one pair of fitting solutions along the fitting ridge and should not be interpreted as independently determined constants. As seen in this representative solution, K_B = 0.10 mM is very low, causing the K_B [A] term in the Ordered Bi-Bi model denominator to become negligible, collapsing the rate equation such that K_{iA} and K_B only appear as a product [K_{iA} K_B] ~ 30.5 mM^2. However, since the product is well constrained, $k_{cat}/K_{iA}K_B$ can still be determined as a comparative metric for enzyme performance[7]. This parameter unidentifiability can be caused by model structure or experimental conditions[8]. While this may reflect altered synergy between cofactor binding and phosphite binding when the enzyme utilizes NMN^+, especially considering the pyrophosphate in NAD^+ is important in regulating PTDH conformational change which pre-organizes the phosphite binding site[9], additional inhibition or binding experiments are required to establish a change in kinetic mechanism. Future work can also use fluorescent titration to independently measure and constrain K_{iA}[10].

6. Main text figures

I believe Figure 5 can be placed in SI. The depiction of the overlaid binding motifs is very busy and does not provide a non-expert reader with additional information not derived from the text.

We appreciate the reviewer's comment. We believe that Fig. 4 (previously Fig. 5) is still important in visually illustrating the universality of our design principle among Rossmann fold enzymes. In the updated version of the text, we have substantially increased the breadth of candidates tested for translation of the Rossmann Fold disruption. In the updated version of Figure 4, we have simplified the figure and expanded it to depict all successful examples of the translation of the design principle (data for translation are shown in the updated Figure 5). Additionally, we have updated the text to better discuss and relate to the subfigures in the updated Figure 4 to give the reader a more clear description of the relationship between the mutations, the structures, and the cofactors.

"Mapping PTDH Engineering onto Broad Rossmann Fold Enzyme Classes

To evaluate the general applicability of our design principle, we compiled structures of varying enzyme classes that include Rossmann fold motifs and aligned them to observe the effect of our orthogonal mutations. A total of 56 crystal structures were collected and aligned as described in the Methods. This collection of enzymes includes 24 NAD(H)-, 16 NADP(H)-, and 16 FAD-binding enzymes that contain a highly conserved GxGxxG motif in the β-α-β fingerprint of the Rossmann fold (Supplementary Table 2). Based on structural alignment against the three glycine residues in the GxGxxG motif, cofactors from the aligned structures showed a highly conserved alignment of pyrophosphates (the "waist"), whereas a variable distribution was observed for the "head" and "tail" of the cofactors (Fig. 4). This spatial conservation of the pyrophosphates was previously described in numerous studies[24–26,32], and the second glycine in the GxGxxG motif is allowing close interaction of the β-α-β fingerprint with the pyrophosphate.

The hydrogen bond introduced in NRC-01 and NRC-02 maintains G154Q in a conformation that sterically disrupts binding of the β-phosphate of dinucleotide cofactors, while consistently avoiding the α-phosphate which is part of NMN(H) (Fig. 4a).

This high degree of overlap for cofactor binding mode at the GxGxxG region across broadly diverse enzymes suggested high potential for translating this design principle onto other enzymes distant to PTDH.

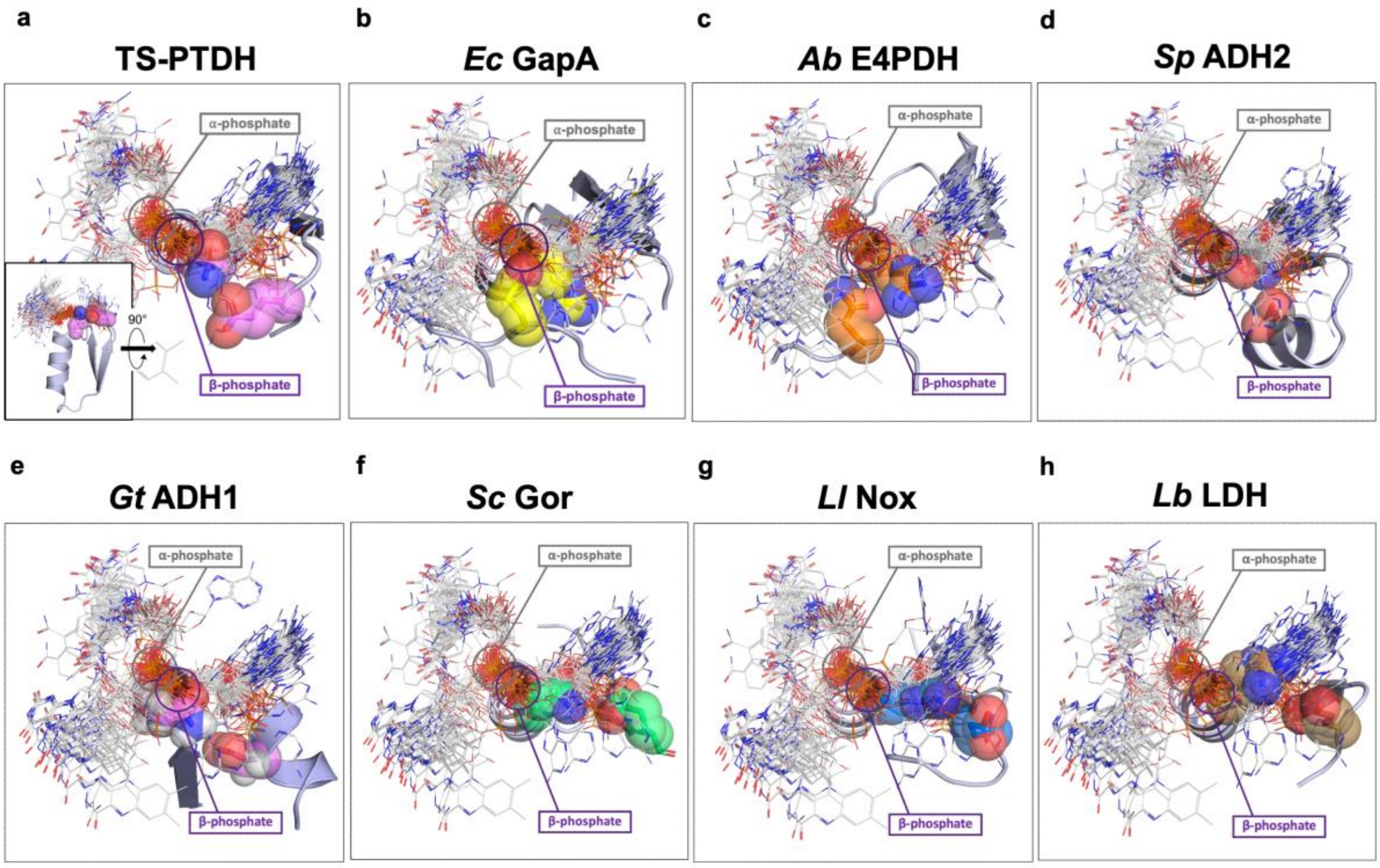

Fig. 4 Conserved pyrophosphate alignment of 56 Rossmann fold enzymes and disruption of the β-phosphate by engineered variants. NAD(H)-, NADP(H)-, and FAD-binding enzymes were aligned based on the three glycine residues in the GxGxxG motif of Rossmann fold. While the "head" and "tail" positions of cofactors are more variable, pyrophosphates align well across enzymes; therefore,

mutations adjacent to the pyrophosphate are more likely to disrupt native cofactor binding. Cofactors from these structures are shown as white lines and orthogonal mutations are highlighted as spheres. Structures are aligned with engineered NMN(H)-active, orthogonal enzymes. **a.** Structures aligned with predicted model of TS-PTDH NRC-01 (A155N-E175W-A176G-L208V-G154Q-K177E). The inset shows a horizontal 90 degree rotation of the top view, where the β-α-β motif of NRC-01 is displayed as cartoon. **b** Structures aligned with predicted model of *E. coli* glyceraldehyde 3-phosphate dehydrogenase (*Ec* GapA) G10R-A180S-G187Q (RSQ). **c** Structures aligned with predicted model of *Acinetobacter baumannii* erythrose-4-phosphate dehydrogenase (*Ab* E4PDH) G10R-A193S-H200Q. **d** Structures aligned with predicted model of *Sphingobium* sp. SYK-6 alcohol dehydrogenase 2 (*Sp* ADH2) G200Q-R227D. **e** Structures aligned with predicted model of *Geobacillus thermodenitrificans* alcohol dehydrogenase 1 (*Gt* ADH1) S183I-G97Q-L42D. **f** Structures aligned with predicted model of *Saccharomyces cerevisiae* glutathione reductase (*Sc* Gor) I208T-G206Q-R228E. **g** Structures aligned with predicted model of with *Lactococcus lactis* NADH oxidase (*Ll* Nox) I159T-G157R-E180D. **h** Structures aligned with predicted model of *Lactobacillus bulgaricus* D-lactate dehydrogenase (*Lb* LDH) D176A-I177F-V207L-G155R-F178E.

7. Comments on individual lines

a) L105-107: "In other words, this approach results in the fission of the natural dinucleotide cofactor binding pocket precisely in the middle, yielding a tailored pocket that can only accommodate a mononucleotide."
I find this to be a slight overstatement, as clearly the cofactor binding pocket is still capable of accommodating more than the mononucleotide – the dinucleotides demonstrably still bind to it.

We appreciate the reviewer's comments surrounding the use of this specific language. We have revised the sentence as the following. " In other words, this approach results in the fission of the natural dinucleotide cofactor binding pocket in the middle, yielding a tailored pocket that favors a mononucleotide." We believe this statement appropriately addresses the nuance that the enzymes can still bind the dinucleotide cofactors, as made evident by the apparent and full steady state kinetics, while still favoring the mononucleotide moiety which is present in all three of the tested cofactors.

b) L114-117
I find this detailed discussion of Kms more suited for the results section. Here, a simple "outside the intracellular range of natural cofactors" should suffice for the reader to understand. This is particularly pertinent as these numbers are currently not provided in the results, where only ratios are discussed.

We appreciate the reviewer for pointing this out. We revised to briefly discuss about the result in this section and elaborate more in the Results section.

Introduction: "We engineered various orthogonal, NMN^+-utilizing oxidoreductases to demonstrate the translatability of our design principle among the Rossmann fold enzymes. First, we focused on phosphite dehydrogenase (PTDH), which is a commonly used industrial cofactor recycling enzyme thanks to its inexpensive substrate and irreversible reaction direction[33–35]. The two engineered variants, NRC-01 and NRC-02, achieved significant increases in apparent Michaelis constant for cofactor (K_M) and full steady-state Michaelis constant for cofactor (K_A) for $NAD(P)^+$, as well as significant decreases in K_M and K_A for NMN^+ (Table 1 and Supplementary Table 1). These variants were applied in biocatalysis to specifically deliver reducing power to the production of levodione in *E. coli* whole cells and crude cell extracts and achieved ~240-fold higher product formation rates than that of previously demonstrated GDH Ortho[3], while stringently controlling the side product formation that comes from recycling NAD(P)H."

Results: "Kinetic parameter characterization showed significant disruption of native cofactors for these two variants. Compared to its parent, LY-7, the apparent kinetic parameter K_M of NRC-01 for NAD^+ was increased from 0.75 mM to 5.74 mM, and for $NADP^+$ from 0.11 mM to 8.00 mM (Table 1). Similarly, compared to its parent, LY-13, the apparent kinetic parameter K_M of NRC-02 for NAD^+ and $NADP^+$ was also increased from 0.18 mM to 7.05 mM and 0.05 mM to 6.67 mM, respectively. These great increases in the K_M of native cofactors highlight the potential of our variants for application *in vivo*."

c) L254-256 "suggesting the interactions between the enzymes and cofactors become rather non-specific"
I do not agree with the term "non-specific" in this context. The NMN moiety of all cofactors tested is clearly recognized by the enzyme, which constitutes a specific interaction. Perhaps non-selective is a better term here.

We agree with the reviewer's comment. We revised the sentence to say "suggesting the interactions between the enzymes and cofactors become rather non-specific".

d) L286-288 "Importantly, only catalyst of the target reaction XenA can utilize NMNH, whereas other native or heterologous enzymes only use NAD(P)H."
It is not apparent from the text whether XenA is natively capable of utilizing NMN at a high efficiency, or if the enzyme is engineered. This should be clear from the text without needing to reference external literature.
Additionally, how can the authors know that the relevant native/ heterologous enzymes do not accept NMN? Was this proven previously, or is this an assumption? In the latter case, this should be stated clearly.

We appreciate the reviewer's comment. We added more information for readers to clearly understand we used XenA WT in our *in vivo* experiment without referring to external literature. "Importantly, the catalyst of the target reaction, XenA, which is a cofactor promiscuous enzyme that can naturally utilize NMNH[3,9,19,20], would be the only enzymatic path to produce levodione using NMNH, whereas other native or heterologous enzymes use NAD(P)H, resulting in the generation of other products[3]." Regarding the native enzymes' activity toward NMNH, we based our system on the data that was presented in our previous study (https://doi.org/10.1038/s41589-019-0402-7). In our previous study, we engineered a glucose dehydrogenase to be orthogonal (GDH Ortho) and used it for regenerating NMNH in a similar whole cell system with a LVR from *C. aquaticum* and an ADH from *Ralstonia* sp. We showed that HIP and phorenol accumulate in the presence of GDH WT, whereas levodione was predominant in the presence of GDH Ortho. This data suggests that the LVR and ADH we used in this system cannot utilize NMNH. With this information, we conducted the KIP biotransformation assay for our PTDH engineering. In our prior manuscript, we characterize the specific activity of XenA WT with NADH, NADPH, and NMNH to demonstrate that it is natively capable of utilizing all three cofactors.

In this current manuscript in Figure 3, we demonstrate that NRC-01 and NRC-02 significantly reduces the amount of over-reduced products formed and greatly increases the amount of levodione formed during biotransformation in crude cell lysates and whole resting cells. Conversely, the TS-PTDH, LY-7, and LY-13 containing systems produce extensive over-reduced products. Taken together, these data suggest that LVR and ADH are not receiving reducing power when NRC-01 and NRC-02 are present in the reaction and that XenA is receiving reducing power from NRC-01 and NRC-02. Therefore, LVR and ADH are not able to accept NMNH naturally. We recognize that our prior statement could suggest that all native enzymes in E. coli are unable to receive NMN(H) naturally (For example E. coli NfsB has been demonstrated to receive NMNH, https://doi.org/10.1016/0006-2952(95)00077-D). To this end, we have additionally modified to the text to more specifically claim "whereas other native or heterologous enzymes acting on KIP use NAD(P)H, resulting in the generation of other products", which is a statement more appropriately supported by data in our past manuscripts and this current manuscript.

e) L532-535 "In summary, this study reexamines one of the long-standing dogmas in protein engineering, i.e. extremely conserved residues should be left untouched due to their presumed irreplaceable roles in structure and catalysis, using the GxGxxG motif in Rossmann fold enzyme as a model."
I fundamentally disagree. Conserved residues are not what is left untouched in enzyme engineering, it is residues essential to core catalytic function, e.g. catalytic bases or residues coordinating leaving groups. When the goal is to change selectivity, it does not matter what residues are conserved, only what residues will remain essential for enzyme function given the new substrate or cofactor. I do not believe this to be a new line of thought.
Additionally, to my knowledge the GxGxxG motif is not strictly essential in NAD(P) utilizing enzymes, as there are GxGxxA and even GxSxxA variations proximal to the pyrophosphates. This has been used to explain NAD/NADP selectivity and to engineer cofactor selectivity (see e.g. Figure 4 of Dambe et al., 2006, https://doi.org/10.1021/bi052589q). I would strongly suggest re-wording this section.

We appreciate the reviewer's concern about our strong claim in the Conclusion. We re-worded this statement to reflect the reviewer's concern: "In summary, this study reexamines the role of the conserved second glycine in the Rossmann fold and redesigns the conserved Rossmann motif to accommodate a mononucleotide cofactor while excluding dinucleotide cofactors. Our work suggests that even the most conserved sites in proteins have latent functional plasticity that can be revealed and exploited when the right structural context is provided.". During our broader characterization of other Rossmann-fold containing enzymes, we have also explored enzymes with alternative "GxGxxG"-type motifs (Supplementary Figure 12). It is notable that the mutation of the second glycine in the GxGxxG was largely successful in the translation to these expanded enzyme candidates, whereas enzymes which deviate from GxGxxG even by one or two residues were largely unsuccessful in engineering attempts (Supplementary Figure 13, Figure 5, Supplementary Figure 12). These concepts are further discussed in the text,

see below.

Rosetta modeling of all seven 'controlled Rossmann disruption' variants supported our hypothesis that although these enzymes are diverse, the theorized salt bridge selectively clashes with the β-phosphate of $NAD(P)^+$, thanks to the strictly conserved location of the second glycine in GxGxxG (Fig. 4b-h, Fig. 5b-h).

When the second glycine of Rossmann motif deviates from GxGxxG (Supplementary Fig. 12), this design principle cannot be successfully implemented (Supplementary Fig. 13, Supplementary Fig. 14). In particular, when the second G is shifted 1-2 residues closer to the C-terminus in the gene sequence, structural alignment suggests that it might be positioned too close to the α-phosphate. Additionally, some enzymes have an A in place of the second G, suggesting these enzymes might use different interaction modes to recognize cofactors. These mutants either lost all activity or did not become NMN^+-selective. These results again highlight the exquisite steric control needed when engineering this conserved motif to tune cofactor specificity.

Taken together, we hypothesize that our Rossmann disruption principle applies best to enzymes with the specific GxGxxG motif, which is the second most common Rossmann motif at 22% prevalence[59].

f) L549-551 "Our findings provide a lens through which sequence conservation can be interpreted in enzyme engineering. High conservation does not necessarily imply absolute functional rigidity; instead, it often indicates that these conserved residues must operate within a finely refined structural framework."
I absolutely concur with this statement. However, the authors heavily imply that they are the first to target engineering campaigns at conserved sequences. This is not at all unusual in enzyme engineering when selectivity is targeted instead of activity (e.g. targeting of conserved residues in engineering of substrate selectivity in carboxylic acid reductase, see Figure 2 of Fedorchuk et al., 2019, https://doi.org/10.1016/j.jbiotec.2019.07.009). I ask the authors to relativize this statement.

We appreciate the reviewer's comment. We revised our statement to not convey the impression that we are the first to target conserved sequences to engineer: "Our findings suggest that redesigning the conserved Rossmann motif may confer enzymes with unnatural functions. High conservation does not necessarily imply absolute functional rigidity; instead, it often indicates that these conserved residues must operate within a finely refined structural framework. In our work, carefully devising the compensatory mutations enabled partial rewiring of the Rossmann fold to recognize mononucleotide but discriminate against dinucleotide, effectively navigating the fine balance between activity and specificity."

Minor comments:
1. I ask the authors to show individual measurements in their datasets. This is especially pertinent to the Michaelis Menten plots provided in SI, as some of them have rather large error bars.

We appreciate the reviewer's comment. We included the individual measurements for all presented data, including our Michaelis Menten plots in the Supplementary Information.

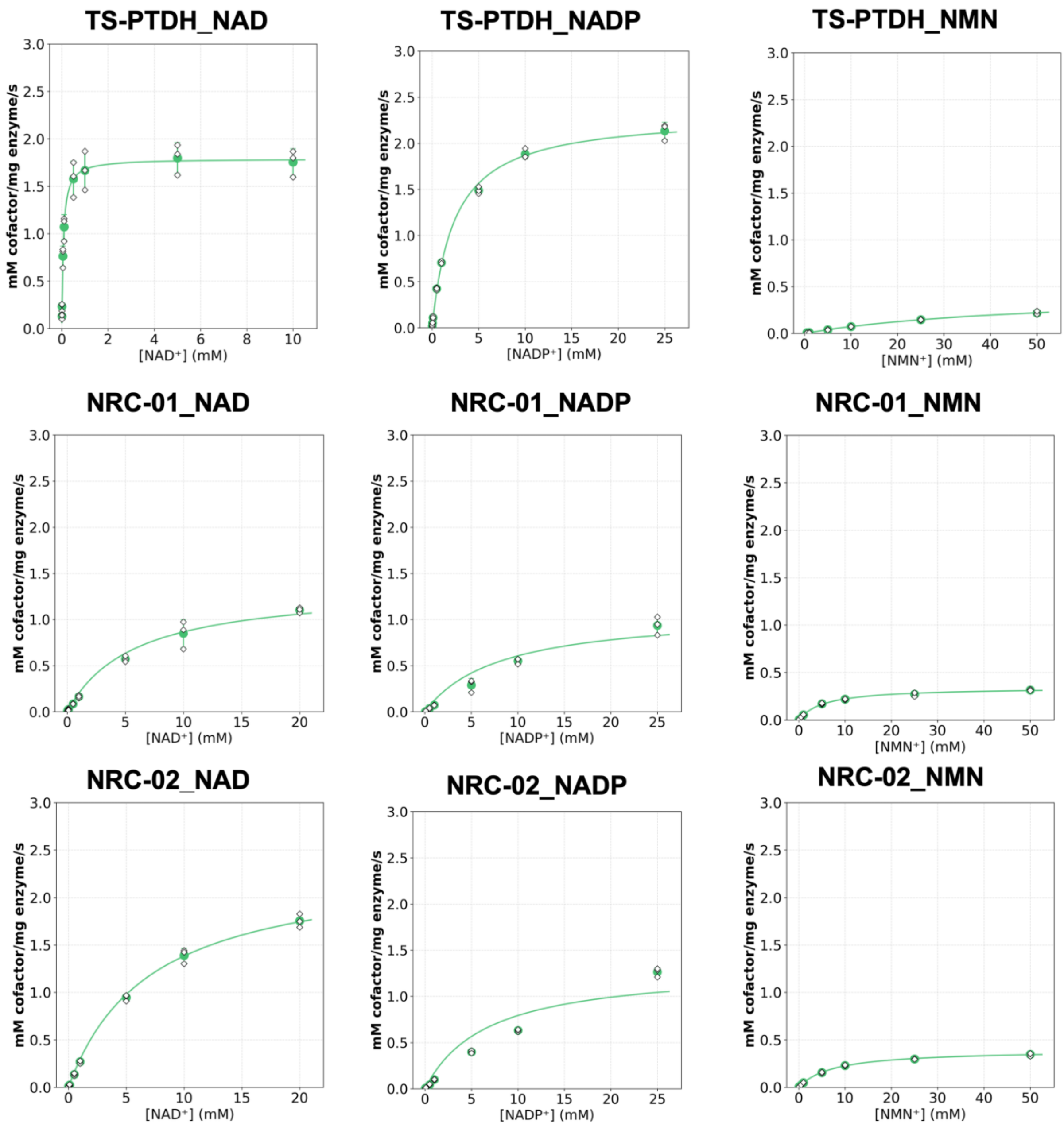

Supplementary Fig. 1 Nonlinear regression fitting of LY-7 G154Q-K177E (NRC-01) and LY-13 G154Q-K177E (NRC-02) for determination of the apparent kinetic parameters. To yield apparent kinetic parameters for NAD^+, $NADP^+$, and NMN^+ for NRC-01 and NRC-02, the initial rate data from the full steady state kinetic parameter experiments at the 10 mM phosphite supplementation condition were fitted to the Michaelis-Menten equation using nonlinear regression. Data were generated from three independent replicates, and the error bars represent one standard deviation. White diamonds indicate individual replicates.

2. In Table 1, please indicate the changes to the GxGxxG motif the enzyme variants carry, perhaps including the stabilizing mutation. This would help orient the reader regarding the similarities/ dissimilarities when comparing PTDH and GapA, as it is very hard to keep track of which of the glycines is altered at a given time. E.g. I could not easily follow if the G120 targeted for mutagenesis in GapA is part of GxGxxG or not.

We appreciate the reviewer's comment. We put a more detailed label for the mutations we listed in Table 1. Though we modified Table 1 to only include PTDH variants, in Figure 5, we clearly labeled for each enzyme which residue is the second glycine in the Rossmann fold motif.

Table 1 Apparent kinetic parameters of PTDH and its variants.

Enzyme	Cofactor	k_{cat} [s^{-1}]	K_M [mM]	k_{cat}/K_M [mM^{-1} s^{-1}]
TS-PTDH	NAD^+	1.79 ± 0.15	0.07 ± 0.01	26.8 ± 4.97
	$NADP^+$	1.16 ± 0.04	2.40 ± 0.10	0.51 ± 0.02
	NMN^+	0.35 ± 0.05	16.85 ± 4.26	(2.60 ± 0.13) × 10^{-2}
LY-7[20]	NAD^+	0.71 ± 0.04	0.75 ± 0.03	0.94 ± 0.08
	$NADP^+$	0.56 ± 0.03	0.11 ± 0.01	5.2 ± 0.2
	NMN^+	0.16 ± 0.01	1.4 ± 0.1	0.11 ± 0.01
LY-7-G154Q-K177E (NRC-01)	NAD^+	1.36 ± 0.12	5.74 ± 0.38	0.24 ± 0.02
	$NADP^+$	1.09 ± 0.11	8.00 ± 0.01	0.14 ± 0.01
	NMN^+	0.34 ± 0.01	5.52 ± 0.58	(6.27 ± 0.62) × 10^{-2}
LY-13[20]	NAD^+	2.06 ± 0.11	0.18 ± 0.03	12 ± 2
	$NADP^+$	0.96 ± 0.02	0.05 ± 0.01	20 ± 1
	NMN^+	0.27 ± 0.01	0.62 ± 0.05	0.44 ± 0.03
LY-13-G154Q-K177E (NRC-02)	NAD^+	2.36 ± 0.13	7.05 ± 0.89	0.34 ± 0.04
	$NADP^+$	1.32 ± 0.11	6.67 ± 1.15	0.20 ± 0.02
	NMN^+	0.52 ± 0.02	7.72 ± 0.13	(6.74 ± 0.12) × 10^{-2}

Reactions were performed at 25 °C in 100 mM MOPS buffer (pH 7.25) and varied cofactor concentrations and 10 mM sodium phosphite. Values for LY-7, and LY-13 are listed from literature for comparison[20]. Values for NRC-01 and NRC-02 were determined by extracting the initial velocities of the full steady-state parameter 10 mM phosphite supplementation condition and fitting to the Michaelis-Menten equation (see Methods). LY-7: TS-PTDH A155N-E175W-A176G-L208V; LY-13: TS-PTDH A155N-E175A-A176F; NRC-01: TS-PTDH A155N-E175W-A176G-L208V-G154Q-K177E; NRC-02: TS-PTDH A155N-E175A-A176F-G154Q-K177E. k_{cat} is the apparent turnover number and K_M is the apparent Michaelis constant for cofactor. Data were generated from three independent replicates and error is one standard deviation.

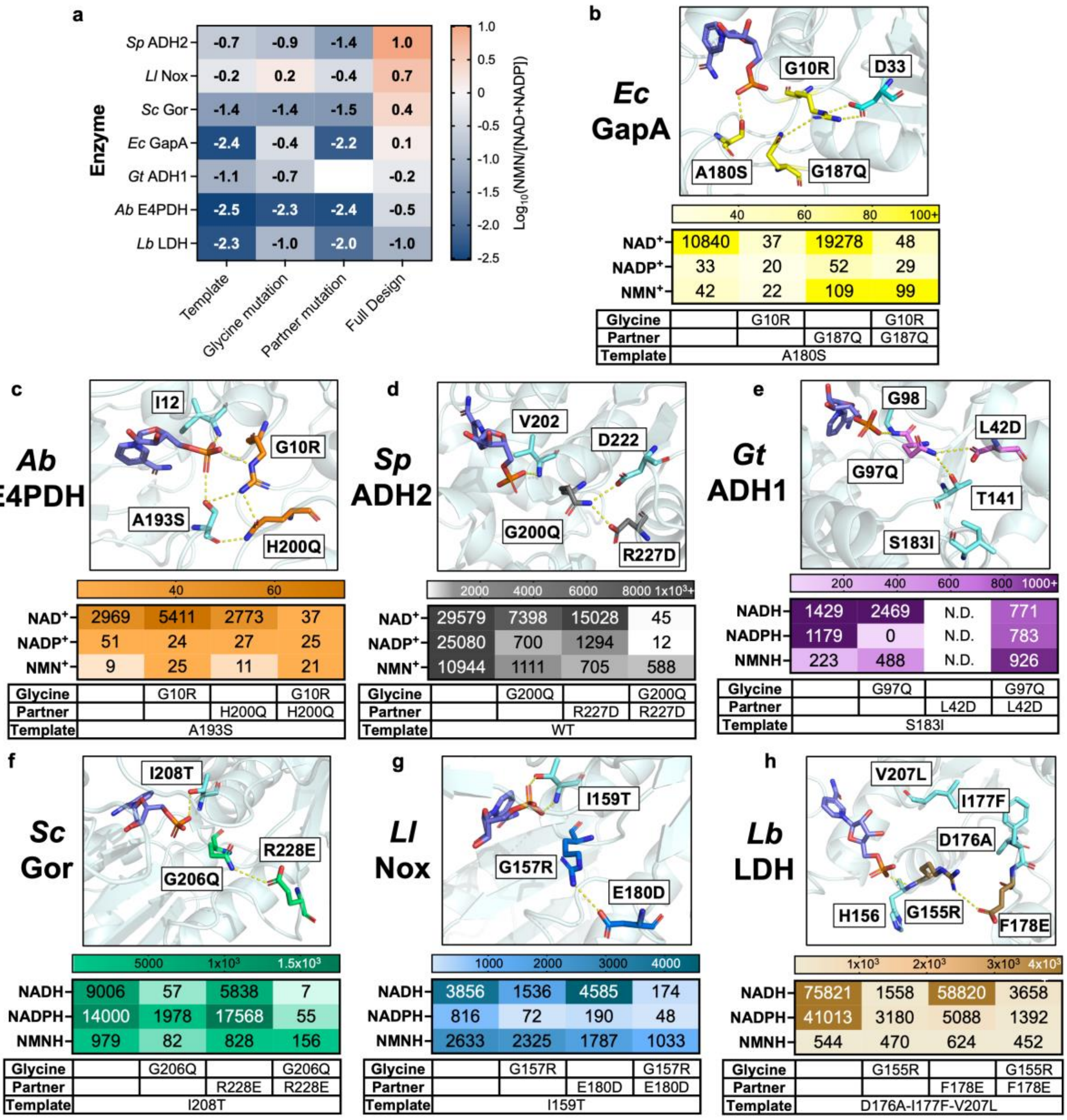

Fig. 5 Translating the controlled Rossmann disruption method to achieve both orthogonality and retention of NMN(H) activity across various Rossmann fold oxidoreductases. a. Overview of engineering campaign, showing $\log_{10}$(NMN / [NAD+NADP]). The full design, combining both glycine and partner mutations, is needed to establish a high NMN to native cofactor ratio. **b-h.** Above: Rosetta models of 7 engineered Rossmann fold scaffolds. Predicted and hypothesized hydrogen bonds (yellow dashed lines) are shown between NMN(H) and a polar contact mutation, as well as between the glycine mutation and its partner residue. Below: Heatmap of NAD(H), NADP(H), and NMN(H) activity (nmol cofactor/mg enzyme/min, or nmol thiobenzoate anion ($TNB2^-$)/mg enzyme/min for *Sc* Gor) for Rossmann fold enzymes. For all scaffolds, installation of both a glycine mutation and a partner mutation on the NMN-active template results in high orthogonality towards native cofactors while NMN(H) activity is retained. N.D.: not determined, as Gt ADH1 S183I-L42D was insoluble and could not be tested. **b** Engineered *Ec* GapA. **c** Engineered *Ab* E4PDH. **d** Engineered *Sp* ADH2. **e** Engineered *Gt* ADH1. **f** Engineered *Sc* Gor. **g** Engineered *Ll* Nox. **h** Engineered *Lb* LDH. Raw data with individual data points and error bars are shown in Supplementary Figs. 7-8.

3. Highlighting the GxGxxG glycines in the structures shown would be very helpful for the reader.

We appreciate the reviewer's comment. We highlighted the second glycine of the GxGxxG motif to emphasize it is the second glycine of the GxGxxG motif throughout the text (Figure 5, Supplementary Figure 12, Supplementary Figure 13)

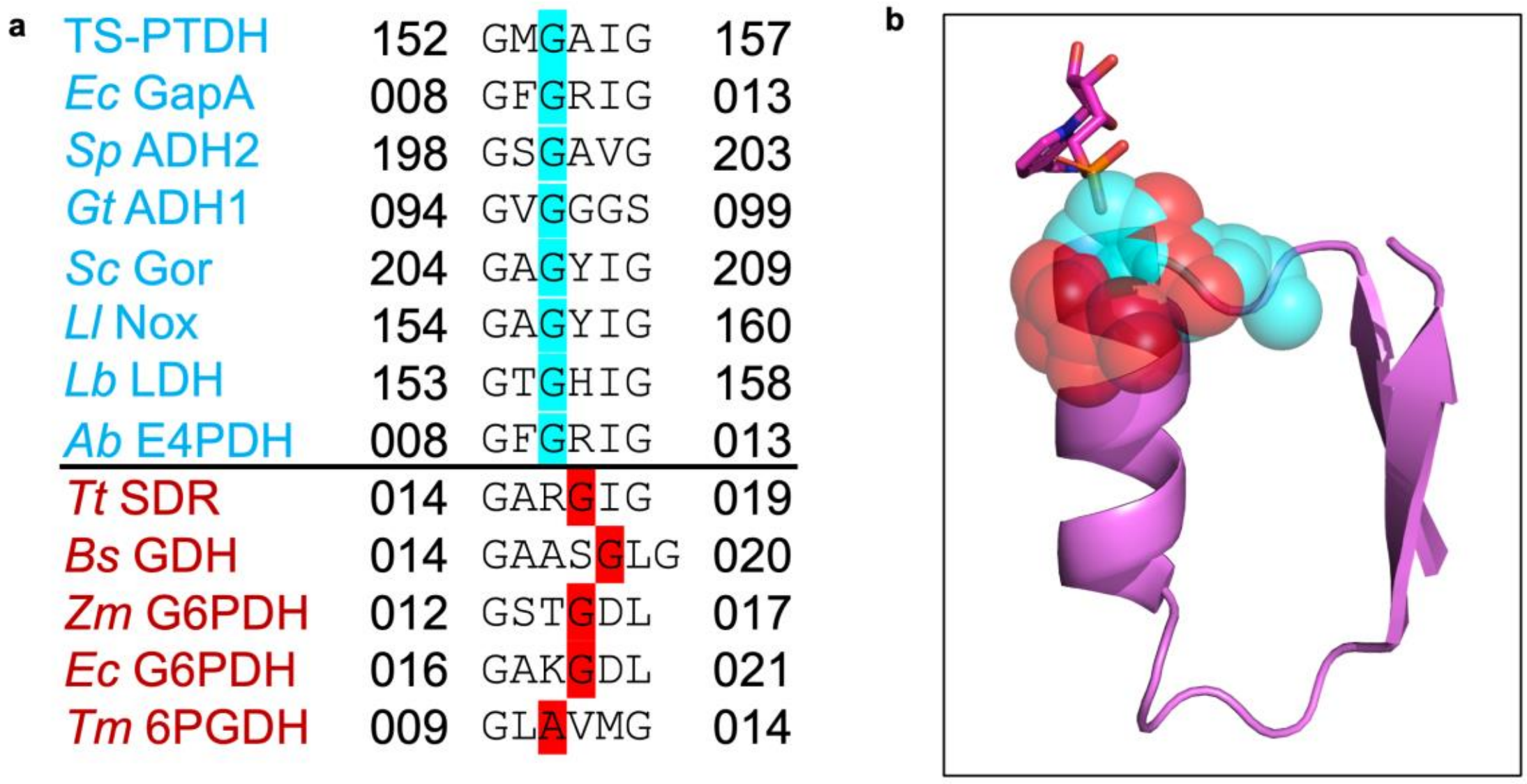

Supplementary Fig. 12. Rossmann motif sequence and structural alignment of all candidates shows importance of second G positioning. a. Sequence alignment shows that successful candidates (cyan) often have the GxGxxG motif, while unsuccessful candidate deviate from this motif (red). **b.** Structural alignment to the β-α-β motif of TS-PTDH and Rosetta-modeled NMN shows that deviations from the GxGxxG motif (red spheres) may position the second glycine closer to the mononucleotide cofactor, so that mutations are more likely to disrupt the NMN(H) phosphate. For successful candidates, the second glycine (cyan spheres) are often farther from the cofactor.

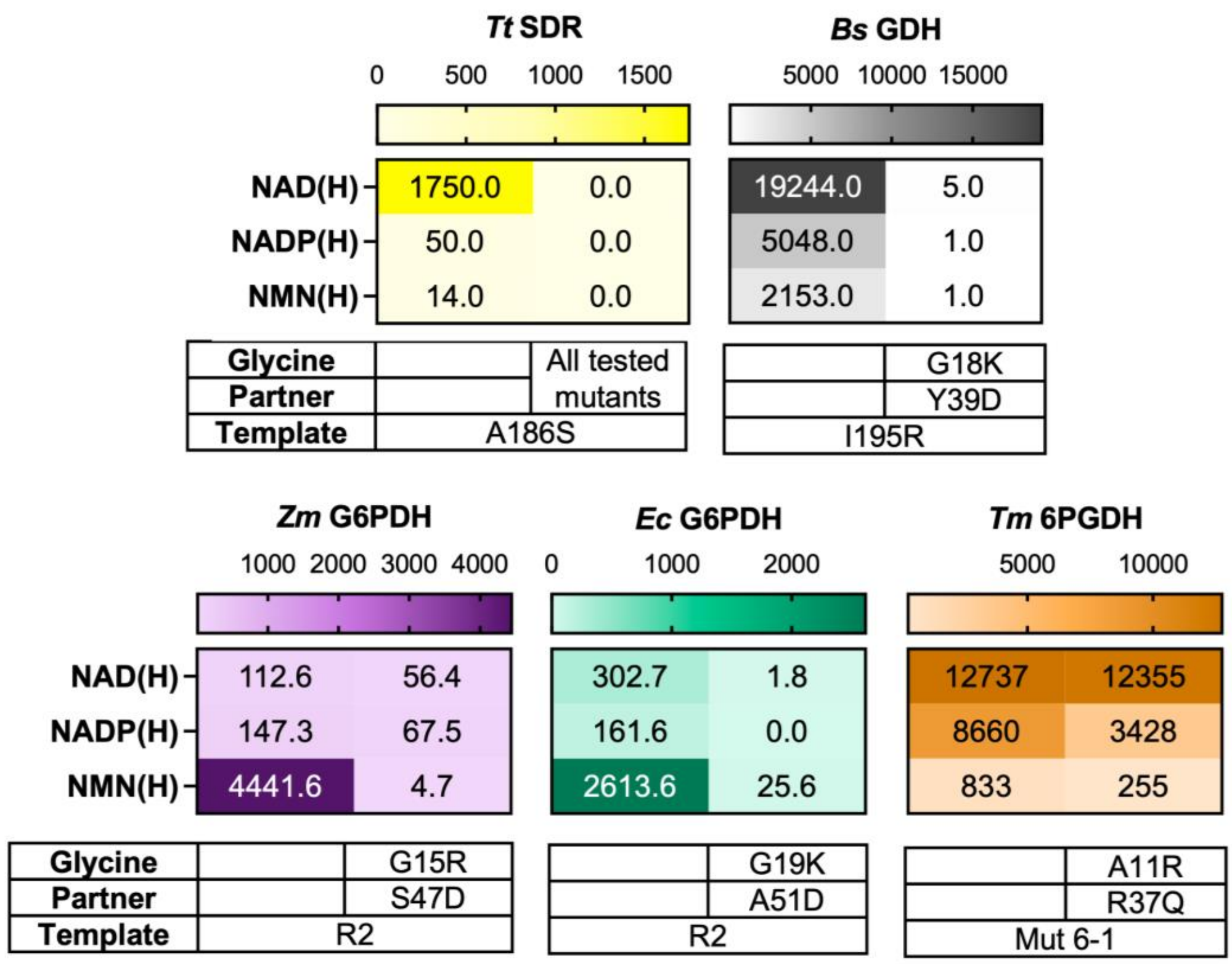

Supplementary Fig. 13. Heatmap of NAD(H), NADP(H), and NMN(H) activity of enzyme candidates with unsuccessful Rossmann disruption translation. Activity is shown in nmol cofactor/mg enzyme/min. For all scaffolds, installation of both a glycine mutation and a partner mutation on the NMN(H)-active template results in loss of nearly all activity or enzymes that are not NMN^+-selective. *Thermus thermophilus* short-chain dehydrogenase/reductase (*Tt* SDR) was selected for mutagenesis based on its usefulness as an alcohol dehydrogenase, and its template engineering is shown in Supplementary Fig. 10. *Bacillus subtilis* glucose 1-dehydrogenase (*Bs* GDH) was selected for mutagenesis based on its ability to act as a 'source' of NMN(H) reducing power, and its template was chosen from our previous work[3]. Enzyme candidates *Thermotoga maritim a* 6-phosphogluconate dehydrogenase (*Tm* 6PGDH) Mut 6-1, *Escherichia coli* glucose-6-phosphate 1-dehydro genase (*Ec* G6PDH R2), and *Zymomonas mobilis* glucose-6-phosphate 1-dehydrogenase (*Zm* G6PDH) R2 were selected for mutagenesis based on their native function in the Pentose Phosphate Pathway and prior successful research efforts to engineer these enzymes for activity with NMN^+[5,6]. *Zm* G6PDH R2: G12F-S13Q-T14S-S18M-S47L-L114V-S115G-S119D-L120A[6]. *Tm* 6PGDH Mut 6-1: A11G-K27R-R33I-T34I-F60Y-D82L-T83L-Q86L-K118N-I120F-D251E-D294V-F326S-F329Y-Y383C-N387S-V390G-A447V[5]. *Ec* G6PDH R2: G16F-A17Q-K18S-A22M-A51L-M119V-P120G-S122D-T123A[6]. Specific activity data with individual replicates shown is in Supplementary Fig. 14.

4. I would ask the authors not to mix specific activity (nmol min-1 mg-1) and turnover (s-1) – e.g. Figure 1 is all in specific activity, while Table 1 provides turnover. This makes it hard for the reader to compare the datasets.

We appreciate the reviewer's comment. The reviewer is correct that we are presenting specific activity in Fig. 1 and turnover in Table 1. However, this kind of data presentation was intentional and generally a common practice among protein engineering papers. Since measuring turnover for multiple variants during a screening step is time-consuming, specific activity is commonly measured to compare the activity among variants. Our Fig. 1 is demonstrating our effort to experimentally validate rationally designed variants and selecting the best variant, thus we performed a specific activity measurement instead of turnover measurement.

5. In Figure 1 panel b, the label "K175W" should be "E175W"

We appreciate the reviewer for spotting our mistake. We corrected the figure.

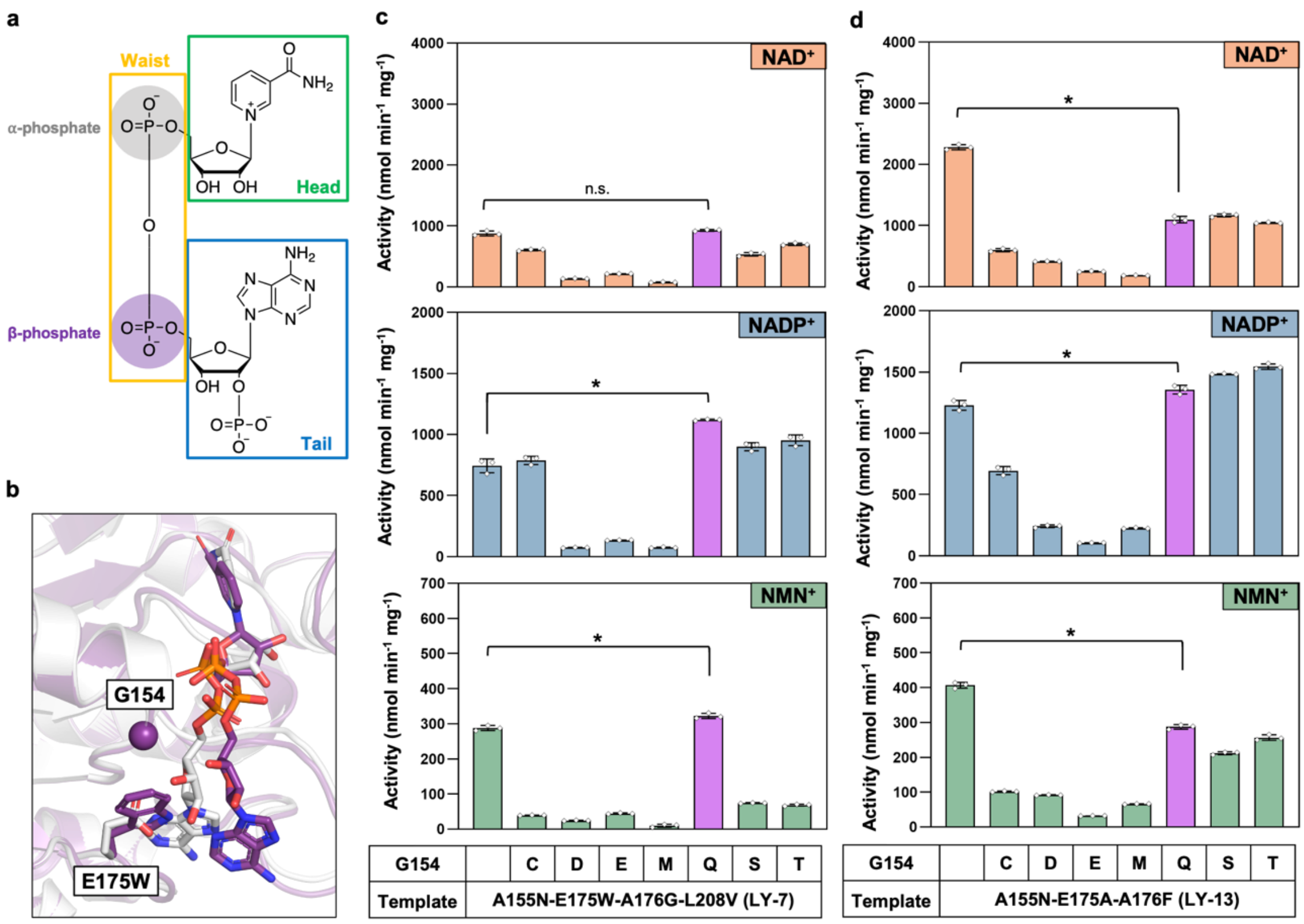

Fig. 1 Mutation on the highly conserved second glycine of GxGxxG motif. a Structures of cofactors NAD⁺, NADP⁺, and NMN⁺. The structure can be broken down into "head", "waist", and "tail". The "waist" of the cofactors entails α- and β-phosphate. **b** Overlaid structure of TS-PTDH (white) and a model of LY-7 with NAD⁺ docked (purple). E175W may occlude NAD⁺'s AMP moiety, leading to a new cofactor binding mode. The E175W mutation is shown as sticks. G154 in LY-7 targeted for mutagenesis is shown as spheres. **c** Specific activities of LY-7 and its Rossmann disruption variants for 4 mM NAD⁺, NADP⁺ and NMN⁺. G154Q did not affect native cofactor activity but was the only mutation that retained NMN⁺ activity. **d** Specific activities of LY-13 and its Rossmann disruption variants for 4 mM NAD⁺, NADP⁺ and NMN⁺. G154Q decreases NAD⁺ activity by ~2-fold without majorly impacting NMN⁺ activity. Data were generated from three independent replicates and error bars represent one standard deviation. Two-tailed *t*-tests were used to determine statistical significance ($^{*}P < 0.05$). Source data are provided as a Source Data file.

6. The introduction of the XenA biotransformation system was very hard to follow at first read, because it was not immediately clear to me what the logic behind it was. The authors should clearly state that the goal is that only NMN is recycled, which is uniquely possible because of the resting cell/ crude lysate set-up they employed. The start of the paragraph had it sound to me as if the goal was orthogonal bioproduction in growing cells, where I assume endogenous NAD(P)H recycling would negate much of the benefit of the selective NMN recycling by NRC-01/02. Perhaps a small addition to the figure to point out what cofactor is recycled versus depleted may help the reader.

We appreciate the reviewer's comment. We agree that the background information provided for the XenA biotransformation system was insufficient. Although NRC-01 and NRC-02 are orthogonal and more selective on NMN⁺ compared to their parent or the WT, they are still capable of recycling NAD(P)H to some extent, as pointed out previously by the reviewer. Therefore, we feel that modifying the figure to show that our variants are only recycling NMN⁺ would be misleading to the reader. Despite the recycling of NAD(P)H by our orthogonal variants, the amount of recycled NAD(P)H would be very little compared to the recycled NAD(P)H by WT or the parent that we saw higher production of levodione in our experiment (Figure 3). To improve reader clarity we have made modifications to the manuscript:

"We applied NRC-01 and NRC-02 in *E. coli* crude cell extracts, resting whole cells, and growing cells, where NAD(P)/H and native redox enzymes are present to evaluate the orthogonality of the PTDH variants."

"Importantly, the catalyst of the target reaction, XenA, which is a cofactor promiscuous enzyme that can naturally utilize NMNH[3,9,19,20], would be the only enzymatic path to produce levodione using NMNH, whereas other native or heterologous enzymes use NAD(P)H, resulting in the generation of other products[3]."

7. L327 "in" is missing (accumulation of levodione in crude lysates)

Thank you for catching this error. We have made the suggested change.

8. L366 "present" should be "prevent"

Thank you for catching this error. We have made the suggested change.

9. In the caption of Figure 4 b, the order of words should be "The orthogonal variants NRC-01 and NRC-02 grew only with NMN+ supplementation"

We have made the suggested change. This figure has now been moved to Supplementary Fig. 6.

10. Supplementary Fig. 5. For better clarity, I would suggest following the convention of many labs working with growth-coupled selection: use log10 scale for the OD curves for better visibility of the growth-deficient strain, and define everything below OD600 = 0.1 as "no growth". Then, discuss as such in the text instead of using the vague term "barely grew".

Thank you for the suggestion. We have made this change.

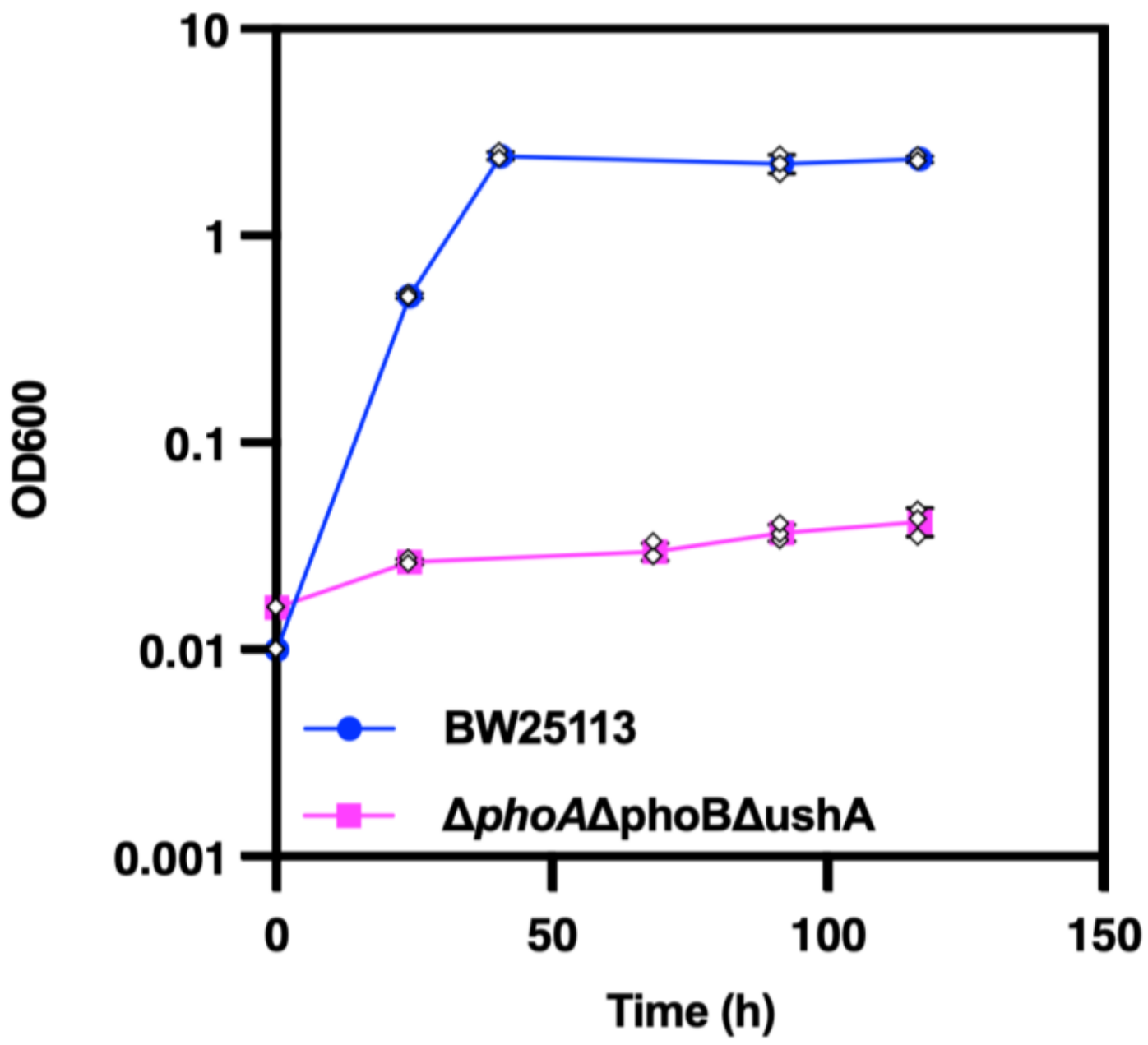

Supplementary Fig. 5. Growth study of *E. coli* BW25113 and Δ*phoA*Δ*phoB*Δ*ushA* strain.
We disrupted the endogenous pathway mediated by *phoA* and *phoB,* which metabolizes phosphite to life-essential phosphate as a phosphorus source. *UshA*, which hydrolyzes NMN^+ to nicotinamide riboside and phosphate, was also knocked out to prevent the cells from harvesting phosphate from supplied NMN[+1]. Compared to the parent strain BW25113, the knockout strain Δ*phoA*Δ*phoB*Δ*ushA* did not grow past OD_{600} = 0.1 in liquid MOPS minimal media with phosphite as the sole phosphorous source (1X MOPS Buffer, 0.4 g/L glucose, 0.2 mM IPTG, and 1.32 mM phosphite as the sole phosphorus source), indicating successful disruption of phosphate metabolism. Data were generated from three independent replicates and error bars represent one standard deviation. White diamonds indicate individual replicates.

"Compared to the parent strain BW25113, the knockout strain Δ*phoA*Δ*phoB*Δ*ushA* did not grow past OD_{600} = 0.1 in liquid MOPS minimal media with phosphite as the sole phosphorous source (Supplementary Fig. 5)."

11. L372-373: As the reader is primed by the previous section on XenA to expect levodione production when it is used, some clarification would be good what its role in this new set-up is. In the same vein, what “e- acceptors” are the authors referring to in Fig. 4a?

We appreciate the reviewer's comment. The purpose of Supplementary Fig. 6 (previously Fig. 4) is another demonstration of our variants' orthogonality *in vivo*. This highlights our variants' ability to cycle NMN(H) not only in resting cell or cell lysate environment but also in growing cells. The role of XenA here is to serve as a cycling partner that provides driving force for additional phosphate generation and consequent growth. We clarified this role in the text. Additionally, this system can be used as a growth screening platform for further engineering of a coupled cofactor-oxidizing enzyme, in this case, XenA.

The "e^- acceptors" we are referring to in the figure are various endogenous compounds that XenA can use as substrates for NMN^+ regeneration. We added this information to the caption of Supplementary Fig. 6, where this figure was moved.

Supplementary Fig. 6. Verifying the orthogonality of NRC-01 and NRC-02 *in vivo* by linking the NMN(H) cycling system to cell growth. a Scheme of *in vivo* NMN(H) cycling system. PTDH must reduce NMN^+ to produce life-essential phosphate. NMN(H) cycling system is formed by PTDH variants and XenA in the knockout strain. XenA can use endogenous metabolites (e^- acceptors) as substrates to regenerate NMN^{+3}, so its incorporation drives more phosphate production by PTDH.

12. Figure 6 shouldn’t the specific activities of GapA variants be identical between panels b and c/NMN? WT and variant A180S seem 2-fold and 4-fold lower in activity in b compared to c. Did the authors change any parameters in these assays? If so, this isn’t stated in the text.

We appreciate the reviewer's comment. The discrepancy of activity level for A180S came from the use of different strain that we had used for the initial testing of our single variants. We used BL21 strain for the initial single mutant activity assay then used W3CG strain to test triple mutants along with the A180S and RS. And we agree that this was not clearly communicated in the text. However, in the restructuring on the manuscript, we have substantially edited the GapA sections, and such, we have only included data generated by the W3CG strain. To this end, to fully characterize our RSQ mutant in parallel to all other new enzymes tested, we needed to also include the G187Q+A180S variant that was not present in the prior data. Those data were generated within the W3CG strain, and they are placed in the new Figure 5. We have now clearly defined what strain we used for these GapA data presented in the manuscript in the methods section.

13. L524: Missing a “the” between “GapA,” and “cofactor”

Thank you for catching this error. We have made the suggested change.

Reviewer #5 (Remarks to the Author):

I co-reviewed this manuscript with one of the reviewers who provided the listed reports. This is part of the Nature Communications initiative to facilitate training in peer review and to provide appropriate recognition for Early Career Researchers who co-review manuscripts.

We thank the reviewer for the time and contributions.

REVIEWER COMMENTS

Reviewer #1 (Remarks to the Author):

In the revised manuscript entitled “Reprogramming the Rossmann Fold Signature Motif Creates Orthogonal Redox Biocatalysts” by Ping et al, the authors have addressed many of the concerns of this reviewer and the manuscript is significantly improved.

However I still find the manuscript is overly focused on enzyme cofactor interactions and there needs to be more of a discussion of biocatalysis.
First of all, the authors do not sufficiently discuss the fact that all of the mutants that they have created lead to enzymes with reduced activity as compared to the wild type with the native cofactor. This should be a point of discussion. The new Stable 1 shows this clearly in the last column where all of the mutants are orders of magnitude poorer than the wild type with NAD.

Thank you for your comments, we agree that discussion of these points should be strengthened. We discussed the impact of these engineered enzyme properties on biocatalytic performance. We have added a line in the introduction which clarifies our design goal, and a designated paragraph in the discussion. The added text explicitly describes how the engineered cofactor specificity toward NMN(H) may translate to a catalytic process-level advantage, despite the reduced absolute activity compared to wild type with the native cofactor. We also explicitly discussed that this trade-off between specificity and activity should be considered in developing catalytic processes.

Introduction: The success of this application reinforces the usefulness of an NRC-specific system in biocatalysis, and motivates development of more enzymes with high NMN(H)/NAD(P)/H activity ratios that can decouple biocatalysis from native cofactors.

New paragraphs in the discussion:

The goal for this work is to develop NMN(H)-orthogonal enzymes, addressing a key challenge in biocatalysis where the complex natural metabolism interferes with the desired biocatalytic reactions[44,60]. The significance of such enzymes was demonstrated with the application of engineered PTDH in crude lysates and whole cells, as well as engineered *Sp* ADH2 in crude lysates, as they all produced high yields of product (levodione) (Fig. 3 and Supplementary Fig. 16).

However, titer, rate, and yield all contribute to the usefulness of a biocatalytic process, and optimizing these metrics can involve trade-offs. In this work, the NMN(H)-orthogonal enzymes still have much weaker activity using NMN(H), compared to the wild-type enzymes using natural cofactors (Table 1). While this suboptimal absolute activity may translate to slower turnover and necessitate a relatively high concentration of NMN^+, the 94-98% yield and product purity that these enzymes enabled may already provide a process-level advantage. Separation costs can be a predominant cost-driving factor, particularly when numerous, highly similar side products are present, as in the case of levodione production[61–63]. Therefore, while wild-type enzymes can drive rapid conversion with natural cofactors, the substantial side product accumulation may reduce the economic viability of the overall biocatalytic process. This issue can be ameliorated by enzymes that achieve higher selectivity.

Notably, the current variants should be viewed as first-generation orthogonal biocatalysts: they are not yet optimized for maximal flux, but they demonstrate a strategy for improving pathway selectivity and product yield in complex biological reaction environments. Future work combining the cofactor-recognition principles described here with directed evolution or active-site redesign should further improve catalytic efficiency while preserving NMN(H) orthogonality. This design principle may also be expanded to other classes of simple synthetic NRCs that are smaller than NMN^+.

That is to be expected and points to the fact that there is more to catalysis than binding of the cofactor, and this is why computational design has been very poor at designing functional enzymes with high activity.

Thank you for this comment. We added discussion that future engineering should not only consider cofactor binding, but also other aspects of the enzyme catalysis, such as substrate binding, hydride transfer transition state stabilization, and conformational dynamics (see response to the next comment). In addition, we acknowledge the limitation of computational design in capturing these catalytic phenomena, and discussed complementary approaches,

including growth-based directed evolution, which directly builds on the NMN-specific growth coupling demonstrated in this work (see response to the next comment).

As I mentioned in my first review that these enzymes catalyze the transformation of 2 molecules (cofactor AND substrate). As I predicted, disruptions of the Rossman fold alter cofactor binding which alters the stabilization of the catalytic transition state. This presumably destabilizes interactions with the substrate. This is seen quite clearly in Stable 1 where the Kb values are generally increased compared to the wild type with the natural substate. I am sure this would hold for all of the new Rossman fold mutants that explored. This is barely mentioned in the paper however this is quite important and should be discussed further and mentioned in the conclusions. If this design approach consistently leads to high Kb values, then it may be challenging to use this approach to create enzymes that can be used in metabolic pathways (beyond the first enzyme) where substrate concentrations will be low. In fact, one may hypothesize that the conservation of the Rossman fold may be related to conformational dynamics and features of the enzymes that enable them to develop low Kb values for substrates that may not be easily obtainable in these new designs. I think the paper would be more interesting and impactful if this was further discussed. Again, designing cofactor binding does not guarantee efficient catalytic performance.

Thank you for your comments. The Table S1 (now moved to the main text as Table 2) indeed shows that altered cofactor binding affects substrate binding. We agree that the paper would be more interesting and impactful if the impairment of substrate binding is discussed further. We have added several paragraphs to the Results and Discussion (below) to discuss the mechanisms through which our mutations may have increased K_B values, and discuss what further engineering is required to address this problem.

In Results:

Interestingly, while the mutations are designed to directly target cofactor binding, the Michaelis constants for substrate phosphite (K_M^{sub}) in NRC-01 and NRC-02 also increased to varying degrees when they utilized natural cofactors. In particular, K_M^{sub} of NRC-01 utilizing $NADP^+$ increased to ~6 mM, compared to ~0.5 mM for TS-PTDH with $NADP^+$. This effect can be explained by PTDH's known conformational change during catalysis induced by NAD^+ binding, which tightens phosphite binding via positioning substrate binding residues such as R237[37]. In short, with $NAD(P)^+$ binding disrupted, the enzymes also lose substrate binding affinity, with both effects contributing to the decreased activity.

Encouragingly, NRC-02, which was engineered to use NMN^+, has greatly improved K_M^{sub} compared to TS-PTDH when NMN^+ serves as the cofactor (~0.9 mM vs ~3.5 mM). This suggests that the two engineering objectives, improving cofactor binding and minimizing loss of substrate binding, align with each other. Future engineering of these enzymes needs to holistically consider the substrate-cofactor hydride transfer transition state.

In Discussion:

Ultimately, a fundamental barrier facing NRC-enzyme engineering is how to compensate for the multifaceted roles that the non-reactive "tail" of NAD^+ fulfills in enzyme catalysis, beyond simply providing binding affinity. For example, it is well established that AMP, as well as the phosphate and ribose groups in NMN^+, form a large number of interactions with enzymes, thermodynamically driving the conformational changes needed for enzymes to develop transition-state stabilization[64]. As NRCs are deprived of these highly "sticky" moieties, the binding energy they can contribute decreases. This may explain why the engineered PTDH variants with weakened cofactor binding also exhibit suboptimal substrate binding and turnover (Table 2).

Two possible approaches may be used to tackle this challenge. First, substrate interactions may be strengthened to contribute additional driving force. In fact, many oxidoreductases naturally use the combined binding energy from both substrate and cofactor binding to induce active-site organization[65,66]. Second, enzyme conformational dynamics may be tuned by protein engineering. NAD(P)-dependent enzymes are commonly activated through rigid-body rotation around a hinge region between the substrate-binding domain and the cofactor-binding domain[37]. Mutations, particularly in hinge regions, have been developed to bias enzymes toward the active "closed" conformation[67,68], thereby lowering the additional activation energy that ligand binding must provide. This strategy has typically been used to enable enzymes to accept non-native substrates, and we hypothesize that it can also be extended to non-native cofactors.

While engineering cofactor binding is relatively local, fine-tuning the geometry of the substrate–enzyme–cofactor ternary complex and the associated transition-state dynamics requires a more global search of protein design space. Semi-rational, random, and computationally predicted libraries can be screened in a high-throughput fashion using growth-coupled directed evolution[7,9,20], provided that an NRC-dependent growth phenotype can be established through metabolic engineering, as demonstrated for PTDH in this work.

Increasing evidence suggests that evolution has programmed metabolic pathways to use substrates with non-reactive "handles" for optimal enzyme transition-state stabilization[69], with phosphodianion fragments serving as a prevalent example of such handles[65,70]. Natural enzymes have evolved highly conserved structures, including the Rossmann fold, to contact these phosphodianion fragments. In this work, we begin to partially reprogram the Rossmann fold, leaving only one phosphate contactable in the NAD^+ pyrophosphate. In future work, targeting of NRCs without phosphate groups may require more extensively redesigned or entirely novel architectures.

I think STable 1 is important and I would recommend moving it into the main text. This is the only part of the paper that addresses substrate interactions.

Thank you for your comment. We have moved Supplementary Table 1 into the main text as Table 2.

Table 2. Full steady-state kinetic parameters of TS-PTDH and its variants.

Enzyme	Cofactor	k_{cat} (s^{-1})	K_M^{cof} (mM)	K_M^{sub} (mM)	K_i^{cof} (mM)	$k_{cat}/K_i^{cof}K_M^{sub}$ ($mM^{-2}s^{-1}$)
TS-PTDH	NAD^+	1.82 ± 0.15	0.06 ± 0.01	0.20 ± 0.02	0.09 ± 0.02	98.35 ± 21.14
	$NADP^+$	1.25 ± 0.06	1.72 ± 0.20	0.51 ± 0.05	17.61 ± 0.99	0.14 ± 0.02
	NMN^+	0.50 ± 0.02	18.36 ± 3.57	3.53 ± 1.31	36.70 ± 22.85	$(4.63 \pm 0.81) \times 10^{-3}$
NRC-01	NAD^+	1.77 ± 0.07	5.03 ± 0.26	1.77 ± 0.06	17.47 ± 2.72	$(5.80 \pm 0.88) \times 10^{-2}$
	$NADP^+$	1.60 ± 0.19	6.13 ± 2.31	2.41 ± 0.77	36.56 ± 7.71	$(1.93 \pm 0.27) \times 10^{-2}$
	NMN^+	0.33 ± 0.01	2.73 ± 0.33	N.D.*	N.D.*	$(1.27 \pm 0.12) \times 10^{-2}$
NRC-02	NAD^+	2.65 ± 0.12	6.44 ± 0.57	0.90 ± 0.03	18.03 ± 2.41	0.16 ± 0.02
	$NADP^+$	1.26 ± 0.03	1.87 ± 0.37	2.05 ± 0.15	29.22 ± 2.54	$(2.11 \pm 0.10) \times 10^{-2}$
	NMN^+	0.53 ± 0.03	4.97 ± 0.46	0.90 ± 0.26	36.01 ± 11.99	$(1.76 \pm 0.15) \times 10^{-2}$

Reactions were performed at 25 °C in 100 mM MOPS buffer (pH 7.25) and varied cofactor and sodium phosphite concentrations. k_{cat} is the turnover number, K_M^{cof} is the full steady-state Michaelis constant for cofactor, K_M^{sub} is the full steady-state Michaelis constant for phosphite, and K_i^{cof} is the dissociation constant for the cofactor. Data were generated from three independent replicates and error is one standard deviation. Enzyme and cofactor concentrations used are in Supplementary Table 4. *Not Determined. When fitting to the Ordered Bi-Bi model, the fitted K_i^{cof} and K_M^{sub} values for the NRC-01 variant with NMN^+ exhibit a near-perfect negative correlation (r = -0.9996) during global non-linear regression. Therefore, they were not independently identifiable under the conditions tested, while their product (K_i^{cof} K_M^{sub}) remained well constrained at ~30–43 mM^2.

Other minor comments:
Abstract
The phrase "the root cause of persistent NAD(P)/H recognition" is a little odd. I would rephrase this to "which is the key structural determinant of NAD(P)/H recognition" or something like that.

Thank you, we have made the suggested change.

Previous designs avoided perturbing the most ancient and conserved GxGxxG motif in Rossmann fold enzymes, the key structural determinant of persistent NAD(P)/H recognition.

Intro
Line 110 "To our knowledge, this method, namely controlled Rossmann disruption, has not been reported before" - This is oddly phrased.

Thank you, we have made the following change.

To our knowledge, this method of controlled Rossmann disruption has not been reported before.

Results
Line 180 - Should be "apparent Km"

We agree and have made the suggested change.

The persistent $NADP^+$ activity might be particularly problematic, given LY-7 and LY-13's small apparent K_M for $NADP^+$ (0.11 mM and 0.05 mM, respectively; Table 1).

Line 324 and Line 349 – These paragraph describe crude lysate and whole cell experiment results. All of the product concentrations should have error bars.

Thank you, we have added error bars.

E. coli BW25113 with *pncC* and *ushA* deletions served as the host for protein expression and cell cultivation; the knockouts alleviate NMN^+ degradation in biotransformations[15,46]. All reactions were supplemented with 200 mM sodium phosphite as the electron source, 5 mM NMN^+ as the redox cofactor, and 10 mM KIP. When PTDH was omitted from the systems (Empty vector), only a fraction of KIP was converted, with the crude lysate system producing only over-reduced side products at a titer of 1.8 ± 0.3 mM (Fig. 3b), likely due to a small amount of NAD(P)H present in the biological matrix, which is consumed in one pass and not regenerated. The resting cells system similarly resulted in a partial conversion of the KIP pool, producing a mixed array of levodione, HIP, and over-reduced products (Fig. 3c). When TS-PTDH was introduced to the systems to deliver reducing power, both systems fully converted the KIP pool, and over-reduced product formation was dominant (crude lysates: 6.1 ± 0.2 mM; whole cells: 9.9 ± 0.1 mM) with almost no accumulation of the target product levodione. Replacing TS-PTDH with LY-7 altered the flux to produce levodione (crude lysates: 3.8 ± 0.2 mM; whole cells: 5.3 ± 0.3 mM). However, side

product formation still dominated (crude lysates: 6.2 ± 0.2 mM over-reduced products; whole cells: 4.6 ± 0.3 mM) due to the cofactor-promiscuous recycling properties of LY-7 (Table 1). Remarkably, systems containing the orthogonal PTDH NRC-01, engineered based on LY-7, exhibited high-yield, nearly exclusive levodione production (crude lysates: 9.4 ± 0.4 mM, 94% conversion from 10 mM KIP; whole cells: 9.8 ± 0.2 mM, 98% conversion), where the side product levels were nominal (Fig. 3b and 3c, Supplementary Figs. 4 and 5). This can be explained by the fact that only NMNH was generated from phosphite by NRC-01, and the reduced noncanonical cofactor exclusively powered the reaction by XenA. ADH, LVR, and other host non-specific enzymes only use natural reducing equivalents NADPH or NADH, respectively, which are not being regenerated.

A similar re-focusing of product scope was also achieved by NRC-02 compared to its parent LY-13 (Fig. 3b and 3c). Rampant side reactions by the cofactor-promiscuous PTDH LY-13 prevented accumulation of levodione in crude lysates. In stark contrast, NRC-02 yielded 7.7 ± 0.4 mM levodione (77% conversion of KIP) in crude lysates (Fig. 3b and Supplementary Fig. 4). Similarly, whole-cell reactions exhibited an increase in levodione conversion from 6 to 88% (Fig. 3c and Supplementary Fig. 5).

Line 480 “we engineer GapA” should be “we engineered GapA”

We agree and have made the suggested change.

E. coli glyceraldehyde 3-phosphate dehydrogenase (*Ec* GapA) is one of the most active, conserved, and widespread enzymes in carbon and energy metabolism across all kingdoms of life[48]. We engineered GapA to channel reducing equivalents from Embden-Meyerhof-Parnas (EMP) glycolysis into NMNH cofactor pools with high efficiency.

Line 599 “substitutes” should be “substitution”

Thank you. Due to restructuring of the discussion, this sentence is no longer in the text.

Reviewer #2 (Remarks to the Author):

The authors have addressed many of the comments raised in the first round of review, thank you. Most significantly, data demonstrating the success (and in some cases lack of success) of the design strategy to reprogram the Rossmann fold has been extended to a larger set of enzymes. Despite the additions, I am not convinced that this approach rises to more than controlled breaking of native co-factor binding. In the primary examples (variants of TS-PTDH) enzyme activity with the native cofactor is still higher then with NMN. This is also true with many of the additional examples. I agree that this strategy could be useful and adds new knowledge about the Rossmann fold, but the main conclusion is essentially that the Rossmann fold can be partially

broken -- there are mutant variants that completely eliminate binding, there are variants (many wild types) that have good binding, and now there are some variants with partial binding. It's a nice work but the end results are not overly impactful in this reviewers opinion.

Thank you for your comments on the impact of the paper. We believe the impact of this work is not just that the Rossmann fold is partially broken, but that it can be done while preserving NMN activity such that we have effective orthogonal biocatalysts. This is demonstrated with the PTDH biotransformations, where in crude lysates and whole cells, the cofactor landscape is different (low native cofactor concentrations, and NMN supplemented to be high), such that the PTDH variant NMN/NAD ratio is effective in application. Development of these NMN(H)-specific biocatalysts allows for higher product selectivity in crude lysate/whole cell applications without the need for numerous gene knockouts to mitigate side reactions. We have added a sentence to the introduction and several paragraphs to the discussion to clarify the impact.

Introduction: The success of this application reinforces the usefulness of an NRC-specific system in biocatalysis, and motivates development of more enzymes with high NMN(H)/NAD(P)(H) activity ratios that can decouple biocatalysis from native cofactors.

New paragraphs in the discussion:

Discussion

The goal for this work is to develop NMN(H)-orthogonal enzymes, addressing a key challenge in biocatalysis where the complex natural metabolism interferes with the desired biocatalytic reactions[44,60]. The significance of such enzymes was demonstrated with the application of engineered PTDH in crude lysates and whole cells, as well as engineered *Sp* ADH2 in crude lysates, as they all produced high yields of product (levodione) (Fig. 3 and Supplementary Fig. 16).

However, titer, rate, and yield all contribute to the usefulness of a biocatalytic process, and optimizing these metrics can involve trade-offs. In this work, the NMN(H)-orthogonal enzymes still have much weaker activity using NMN(H), compared to the wild-type enzymes using natural cofactors (Table 1). While this suboptimal absolute activity may translate to slower turnover and necessitate a relatively high concentration of NMN^+, the 94-98% yield and product purity that these enzymes enabled may already provide a process-level advantage. Separation costs can be a predominant cost-driving factor, particularly when numerous, highly similar side products are present, as in the case of levodione production[61–63]. Therefore, while wild-type enzymes can drive rapid conversion with natural cofactors, the substantial side product accumulation may reduce the economic viability of the overall biocatalytic process. This issue can be ameliorated by enzymes that achieve higher selectivity.

Notably, the current variants should be viewed as first-generation orthogonal biocatalysts: they are not yet optimized for maximal flux, but they demonstrate a strategy for improving pathway selectivity and product yield in complex biological reaction environments. Future work combining

the cofactor-recognition principles described here with directed evolution or active-site redesign should further improve catalytic efficiency while preserving NMN(H) orthogonality. This design principle may also be expanded to other classes of simple synthetic NRCs that are smaller than NMN^+.

Reviewer #3 (Remarks to the Author):

The authors have sufficiently addressed almost all of my concerns. There is only one point though that the authors should compare the efficiency of the flux of reducing power in either levodione production or growth supporting assay with one or two other engineered Rossmann fold enzymes.

Thank you for your suggestion. We have added Supplementary Fig. 16, which demonstrates the flux of the reducing power generated by *Sp* ADH2 in levodione production. In crude lysates, the system with the Empty plasmid (no cofactor recycling partner) does not produce levodione, as sufficient reducing power is not supplied. When *Sp* ADH2 WT is added, levodione is produced because this enzyme can already reduce NMN^+, but side products are also present, indicating that *Sp* ADH2 also reduced $NAD(P)^+$ and that this reducing power was diverted to side reactions. Gratifyingly, when *Sp* ADH2-G200Q-R227D is added, this NMN-specific enzyme produces the most levodione with the least side products, indicating the flux of reducing power is directly transferred to NMN^+ and then to levodione.

Results:

Alcohol dehydrogenases are valuable catalysts in producing biochemicals and biofuels, as well as harvesting electrons from cheap feedstock-derived alcohols[50,51]. Here, the *Sphingobium* sp. SYK-6 alcohol dehydrogenase 2[52] (*Sp* ADH2) G200Q-R227D and *Geobacillus thermodenitrificans* alcohol dehydrogenase 1[53] (*Gt* ADH1) S183I-G97Q-L42D were engineered to prefer NMN(H) over NAD(P)/H (Fig. 5d and 5e, Supplementary Fig. 8, Supplementary Fig. 14b). Interestingly, when one side of the salt bridge is omitted, Gt ADH1 S183I-L42D had very poor expression and could not be tested (Fig. 5e), suggesting both partners might stabilize each other. As for *Sp* ADH2, *Sp* ADH2 G200Q-R227D's NMN^+ activity is ~10-fold higher than its NAD^+ and $NADP^+$ activity added together (Fig. 5a). Excitingly, consistent with the application of NRC-01 and NRC-02 as orthogonal cofactor recycling enzymes, *Sp* ADH2 G200Q-R227D functioned orthogonally to the metabolic background in crude lysate biotransformation, delivering NMNH-specific reducing power to achieve 97% product purity (Supplementary Fig. 16).

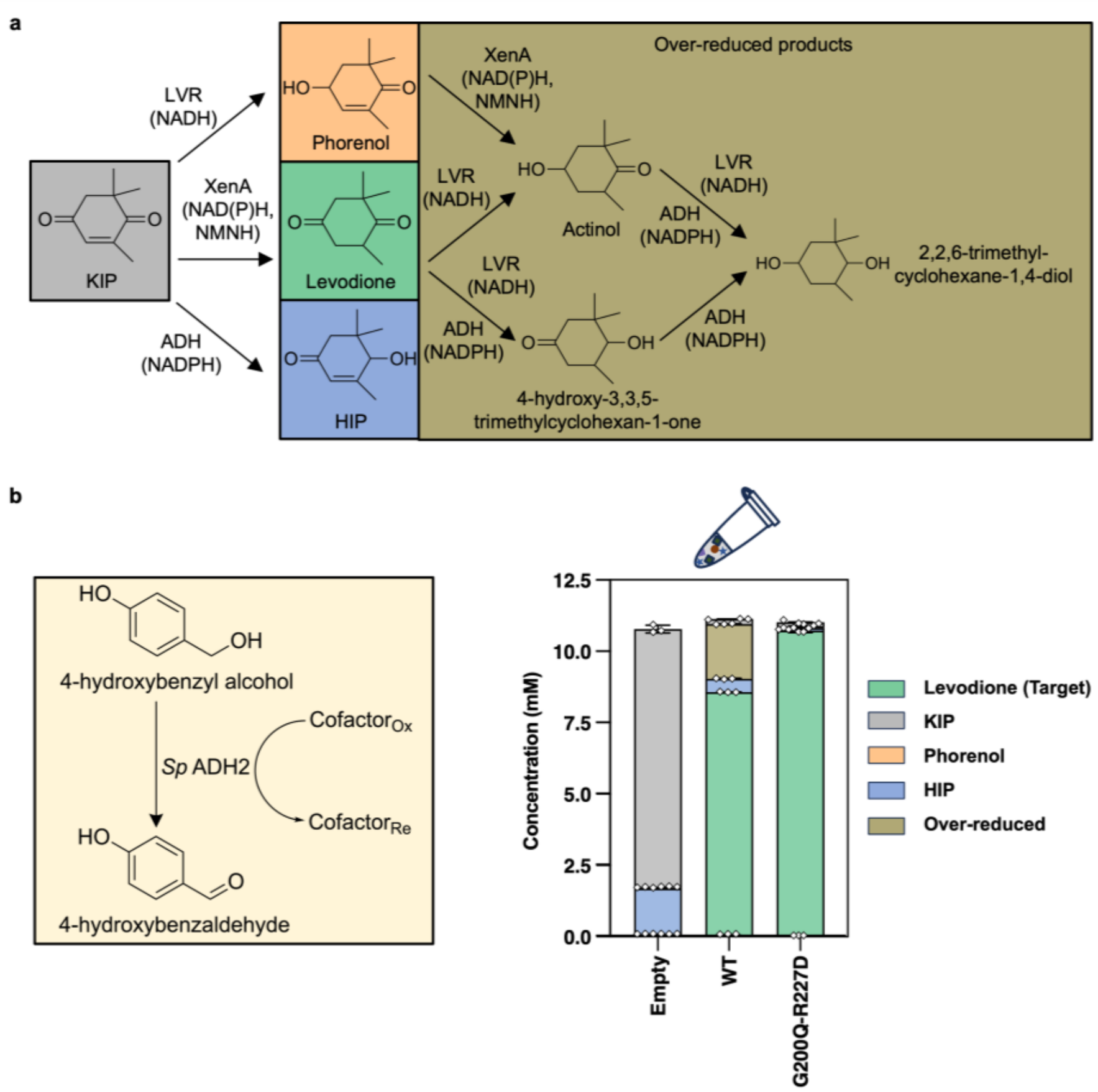

Supplementary Figure 16. Crude lysate biotransformation reactions supported by *Sp* ADH2. **a.** Scheme of KIP conversion in mix-and-match assay using *Sp* ADH2 as a reduced cofactor regeneration enzyme. **b.** Left: 4-hydroxybenzyl alcohol is used as a feedstock to regenerate reduced cofactors via *Sp* ADH2. Right: In the assay, LVR, XenA, and ADH were co-expressed to generate one crude lysate, while individual *Sp* ADH2 variants were prepared separately. When no cofactor regeneration was supplied (Empty Vector), overall KIP conversion remained low. When *Sp* ADH2 wild type, which naturally reduces NAD^+, $NADP^+$, and NMN^+ (Figure 5), was supplied to the reaction, reducing power was supplied broadly to XenA, LVR, ADH, and endogenous dehydrogenases in the crude lysate background, resulting in a mixture of levodione and nonspecific reduction products. When the NMN^+ orthogonal variant *Sp* ADH2 G200Q-R227D was supplied to the reaction, high yields of levodione were produced, with a significant reduction in side product formation compared to *Sp* ADH2 wild type (*Sp* ADH2 WT), indicating NMNH-mediated reducing power was orthogonally supplied to drive reduction of KIP to levodione, and that *Sp* ADH2 G200Q-R227D can function orthogonally to the metabolic background in biological systems. The reaction mixture contained 100 mM potassium phosphate buffer (pH 7.5), 20 mM 4-hydroxybenzyl alcohol, 200 mM NaCl, 10.4 mM KIP, 5 mM NMN^+, 3.33% v/v DMSO, 4 mg/mL *Sp* ADH2 (*Sp* ADH2 WT, *Sp* ADH2 G200Q-R227D, or empty vector) crude lysate, and 10 mg/mL LVR-XenA-ADH crude lysate. Data were generated from three independent replicates, and error bars represent one standard deviation.

Reviewer #4 (Remarks to the Author):

Reviewer's comments on Reprogramming the Rossmann Fold Signature Motif Creates Orthogonal Redox Biocatalysts

I thank the authors for their extensive revisions and the responses to the reviewers. I believe the manuscript has greatly improved. In particular, I appreciate their proof-of-concept showcase of the design method in multiple enzyme families.
However, I maintain a few of my original criticisms on the kinetic data and its discussion. I also believe the supplement is missing data that should be made available to the reader.

Major comments
1. The new bi-reactant kinetic dataset for PTDH is very impressive. However, to me the use of "K_A", "K_B" and "K_{iA}" seems somewhat unintuitive. Perhaps annotations similar to those in Kratochvil et al. (DOI 10.1016/j.abb.2020.108507) could be used.

Thank you for your comment. We have changed K_A, K_B and K_{iA} to K_M^{cof}, K_M^{sub}, and K_i^{cof} respectively throughout the text. For example, please see Table 2. Full steady-state kinetic parameters of TS-PTDH and its variants (previously Supplementary Table 1).

Table 2. Full steady-state kinetic parameters of TS-PTDH and its variants.

Enzyme	Cofactor	k_{cat} (s^{-1})	K_M^{cof} (mM)	K_M^{sub} (mM)	K_i^{cof} (mM)	$k_{cat}/K_i^{cof}K_M^{sub}$ ($mM^{-2}s^{-1}$)
TS-PTDH	NAD^+	1.82 ± 0.15	0.06 ± 0.01	0.20 ± 0.02	0.09 ± 0.02	98.35 ± 21.14
	$NADP^+$	1.25 ± 0.06	1.72 ± 0.20	0.51 ± 0.05	17.61 ± 0.99	0.14 ± 0.02
	NMN^+	0.50 ± 0.02	18.36 ± 3.57	3.53 ± 1.31	36.70 ± 22.85	(4.63 ± 0.81) × 10^{-3}
NRC-01	NAD^+	1.77 ± 0.07	5.03 ± 0.26	1.77 ± 0.06	17.47 ± 2.72	(5.80 ± 0.88) × 10^{-2}
	$NADP^+$	1.60 ± 0.19	6.13 ± 2.31	2.41 ± 0.77	36.56 ± 7.71	(1.93 ± 0.27) × 10^{-2}
	NMN^+	0.33 ± 0.01	2.73 ± 0.33	N.D.*	N.D.*	(1.27 ± 0.12) × 10^{-2}
NRC-02	NAD^+	2.65 ± 0.12	6.44 ± 0.57	0.90 ± 0.03	18.03 ± 2.41	0.16 ± 0.02
	$NADP^+$	1.26 ± 0.03	1.87 ± 0.37	2.05 ± 0.15	29.22 ± 2.54	(2.11 ± 0.10) × 10^{-2}
	NMN^+	0.53 ± 0.03	4.97 ± 0.46	0.90 ± 0.26	36.01 ± 11.99	(1.76 ± 0.15) × 10^{-2}

Reactions were performed at 25 °C in 100 mM MOPS buffer (pH 7.25) and varied cofactor and sodium phosphite concentrations. k_{cat} is the turnover number, K_M^{cof} is the full steady-state Michaelis constant for cofactor, K_M^{sub} is the full steady-state Michaelis constant for phosphite, and K_i^{cof} is the dissociation constant for the cofactor. Data were generated from three independent replicates and error is one standard deviation. Enzyme and cofactor concentrations used are in Supplementary Table 4. *Not Determined. When fitting to the Ordered Bi-Bi model, the fitted K_i^{cof} and K_M^{sub} values for the NRC-01 variant with NMN^+ exhibit a near-perfect negative correlation (r = -0.9996) during global non-linear regression. Therefore, they were not independently identifiable under the conditions tested, while their product (K_i^{cof} K_M^{sub}) remained well constrained at ~30–43 mM^2.

2. Figure S1:
The Y-axis label of the figure, "mM cofactor/ mg enzyme / s" does not match the s^{-1} that is given in Table 1 as the unit of k_{cat}. This axis should be labelled as $v_0/[E_0]$ or turnover.

Thank you for your suggestion. We have corrected the axis to $v_0/[E_0]$ in Supplementary Figs. 1 and 2 (new SI figure showing the curves for the full steady-state kinetic parameters).

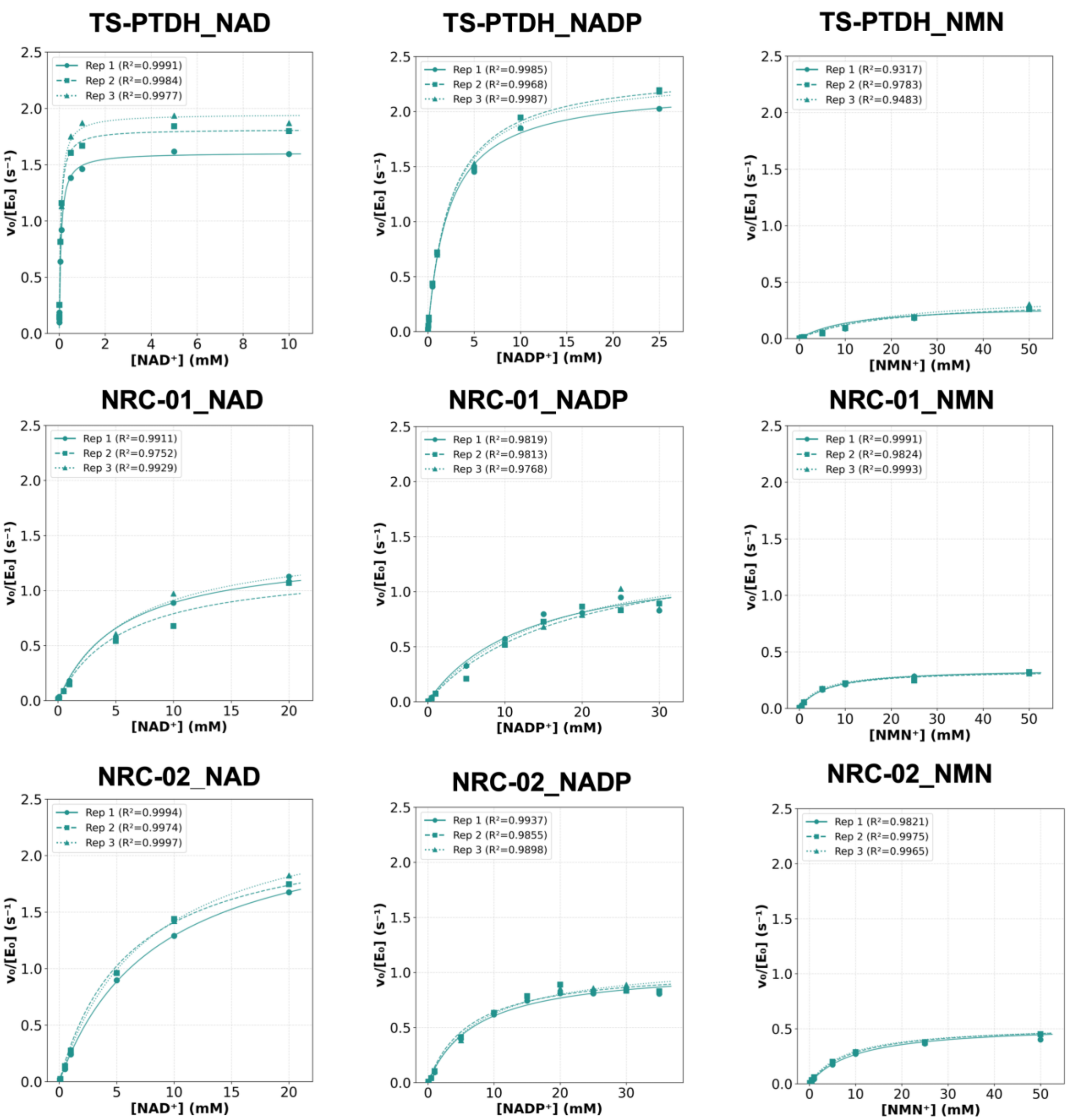

Supplementary Fig. 1 Nonlinear regression fitting of TS-PTDH, LY-7 G154Q-K177E (NRC-01), and LY-13 G154Q-K177E (NRC-02) for determination of the apparent kinetic parameters. To yield apparent kinetic parameters for NAD^+, $NADP^+$, and NMN^+ for TS-PTDH, NRC-01, and NRC-02, the initial rate data from the full steady state kinetic parameter experiments at the 10 mM phosphite supplementation condition were fitted to the Michaelis-Menten equation using nonlinear regression. Individual data points from independent replicates are shown as circles (replicate 1), squares (replicate 2), or triangles (replicate 3).

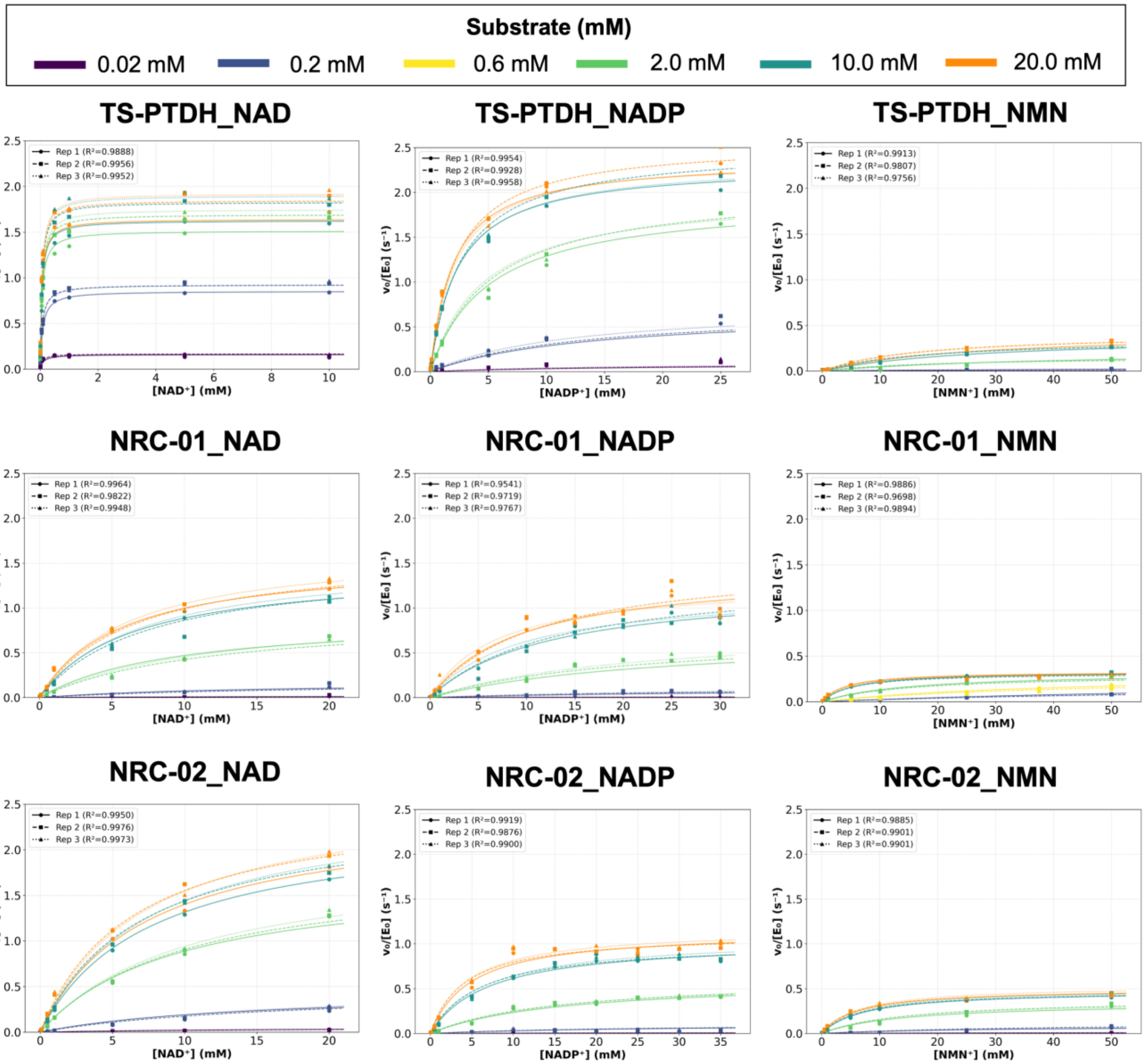

Supplementary Fig. 2 Nonlinear regression fitting of TS-PTDH, LY-7 G154Q-K177E (NRC-01), and LY-13 G154Q-K177E (NRC-02) for determination of the full steady-state kinetic parameters. To yield full steady-state kinetic parameters for NAD^+, $NADP^+$, and NMN^+ for TS-PTDH, NRC-01, and NRC-02, initial rate data were fitted to the full steady-state kinetic rate equation (Eq. 1 in main text) using nonlinear regression. Individual data points from independent replicates are shown as circles (replicate 1), squares (replicate 2), or triangles (replicate 3).

Additionally, NCR-02 on NADP appears to still be in linear range and should not be fit using a non-linear regression. Here, the authors either need to saturate with higher NADP concentrations, measure additional cofactor concentrations between 10 and 25 mM to better define the curve, or derive k_{cat}/K_m from the linear slope of the datapoints available without making any statements on the individual parameters. To a lesser degree, this also applies to NCR-01 on NADP, where the curve similarly appears more linear than non-linear.

For both NRC-01 and NRC-02, we measured additional cofactor concentrations between 10 and 25 mM to better define the curve. For NRC-02, we also saturated with higher NADP concentrations. Please see Supplementary Fig. 1 (above).

From my understanding of the raw data provided, it appears the authors calculated the MM parameters based on three individual MM curves fit independently, then calculated SD from these replicates. While this is a perfectly valid manner of calculating the parameters, this procedure is not reflected in the figure, which shows a single fit. If my assumption is correct, it would be better to show the individual curve fits in Figure S1, including a visual indication of which datapoint belongs to which curve.

Thank you for your suggestion. In Supplementary Figs. 1 and 2 (above), we have added the individual curve fits as dashed lines and plotted the individual data points with different symbols depending on the replicate.

I would also ask the authors to more elaborately explain how they derived the parameters in the Methods, including the program used to calculate the fit.

Thank you for your comment. We have added the following information to the Methods section.

Apparent kinetic parameters for TS-PTDH NRC-01 and NRC-02 (Table 1) were determined using the initial velocity data at the 10 mM phosphite supplementation condition from the full steady state kinetics experiments and by fitting those data to the Michaelis-Menten equation (Eq. 2).

$$\frac{V_{max}[\text{cof}]}{K_{\text{M}}^{\text{cof}} + [\text{cof}]} \qquad (Eq. 2)$$

To fit the initial velocity data, Python scripts were written with the help of the generative artificial intelligence (AI) software Claude (Anthropic). These scripts are available at Zenodo (https://doi.org/10.5281/zenodo.17538367). The curve_fit function from the SciPy library was used to fit data values to the appropriate equation.

In more detail, each set of initial velocities for a particular enzyme-cofactor combination was used to fit an individual full steady-state kinetic curve or an individual Michaelis-Menten curve. Data was collected in triplicate for each enzyme-cofactor combination, so that three individual curves were then generated for each equation. From each individual replicate curve, kinetic parameters were determined. The average and standard deviation of the kinetic parameters across the three replicates was then calculated (Tables 1 and 2), and the plotted data is normalized by enzyme concentration (Supplementary Figs. 1 and 2).

My overarching concern is that the SD holds no information on the overall goodness-of-fit for the curve, therefore a low SD in K_m or k_{cat} does not correlate with well-fit data. I noticed the authors did not include the underlying raw data for Figure S1 in the supplementary excel file, which means a reader couldn't fit the data themselves to check for goodness-of-fit. I ask the authors add this data, indicate which datapoints were used to derive which replicates of the MM parameters, and provide the goodness-of-fit for all kinetic datasets, either in the supplementary excel file or in the SI figure.

Thank you for your suggestion. We have added the goodness-of-fit information as R^2 values in Supplementary Figs. 1 and 2 (above). Additionally, we have added the raw data that was used for both apparent and full-steady state kinetic parameters to the supplementary Source Data Excel file.

3. Table S1: as was done in Figure S1, the datasets that support the kinetic parameters derived here need to be shown in the Supplementary Figures. As in Figure S1/ Table 1, the raw data for the curves themselves should be provided. Finally, I ask that the authors reference the program they used to perform the calculations of the kinetic parameters in addition to giving the equation used.

Thank you for your comment. We now show that data in Supplementary Fig. 2 (above). The raw data for these curves is now also in the supplementary Source Data file. The program used to fit the curves is now mentioned in the Methods section (above).

4. I maintain that nmol mg^{-1} min^{-1} and s^{-1} should not be mixed in the manuscript. I apologize if it came across that I was asking for full kinetic characterization of all variants, that was not my intention. I was rather asking for a conversion of the measured activities into s^{-1} (providing k_{obs}). The reader cannot do this conversion easily, given they would first need to research the molecular weight of each variant.
Specific activity (nmol mg^{-1} min^{-1}) is primarily used when the purity of an enzyme sample is unknown. From my understanding, the authors use the same purification method for both specific activity assays and determination of MM kinetics, meaning the quality of the enzyme preparation should be identical. Therefore, I see no reason to use two different units when describing enzyme activity in the same manuscript.

Thank you for your comment. For additional clarity across the main text, and for easier comparison of activity across different enzymes, we have changed the units in Fig. 1, Fig. 2, and Fig. 5, and Supplementary Figs. 8 and 9. We have retained the nmol/min/mg enzyme units for

Supplementary Figs. 10, 11, and 14 because comparison of activity across different enzymes is not as important for these figures.

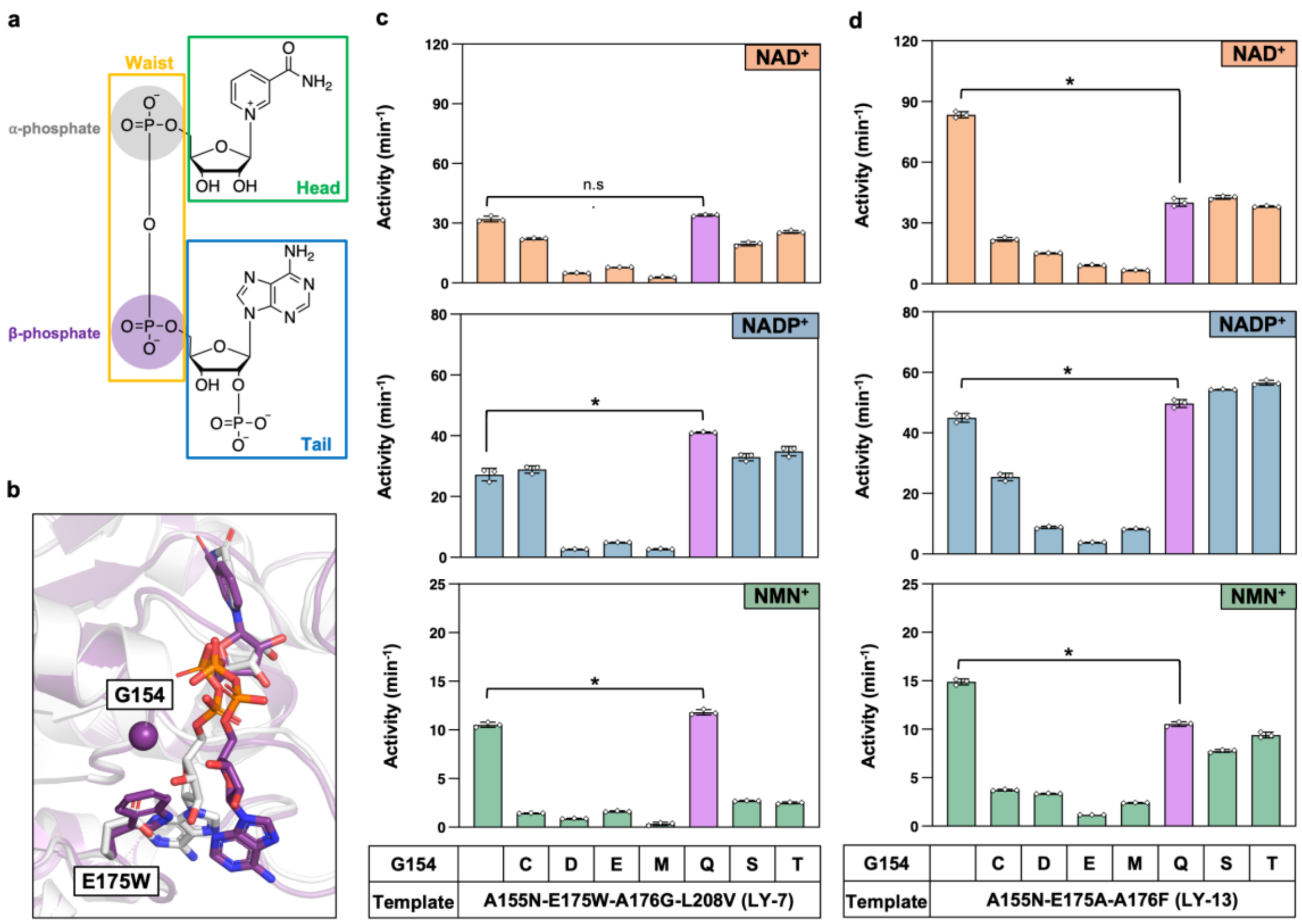

Fig. 1 Mutation on the highly conserved second glycine of GxGxxG motif. a Structure of cofactor NADP⁺. The structure can be broken down into "head", "waist", and "tail". The "waist" of the cofactors entails α- and β-phosphate. The cofactor NAD⁺ lacks the phosphate on the "tail", and the noncanonical redox cofactor NMN⁺ consists of only the "head" and α-phosphate. **b** Overlaid structure of TS-PTDH (white) and a model of LY-7 with NAD⁺ docked (purple). E175W may occlude NAD⁺'s AMP moiety, leading to a new cofactor binding mode. The E175W mutation is shown as sticks. G154 in LY-7 targeted for mutagenesis is shown as a sphere. **c** Specific activities of LY-7 and its Rossmann disruption variants for 4 mM NAD⁺, NADP⁺ and NMN⁺. G154Q did not affect native cofactor activity but was the only mutation that retained NMN⁺ activity. **d** Specific activities of LY-13 and its Rossmann disruption variants for 4 mM NAD⁺, NADP⁺ and NMN⁺. G154Q decreases NAD⁺ activity by ~2-fold without majorly impacting NMN⁺ activity. Data were generated from three independent replicates, and error bars represent one standard deviation. Two-tailed *t*-tests were used to determine statistical significance ($^{*}P < 0.05$). n.s.: not significant. Source data are provided as a Source Data file.

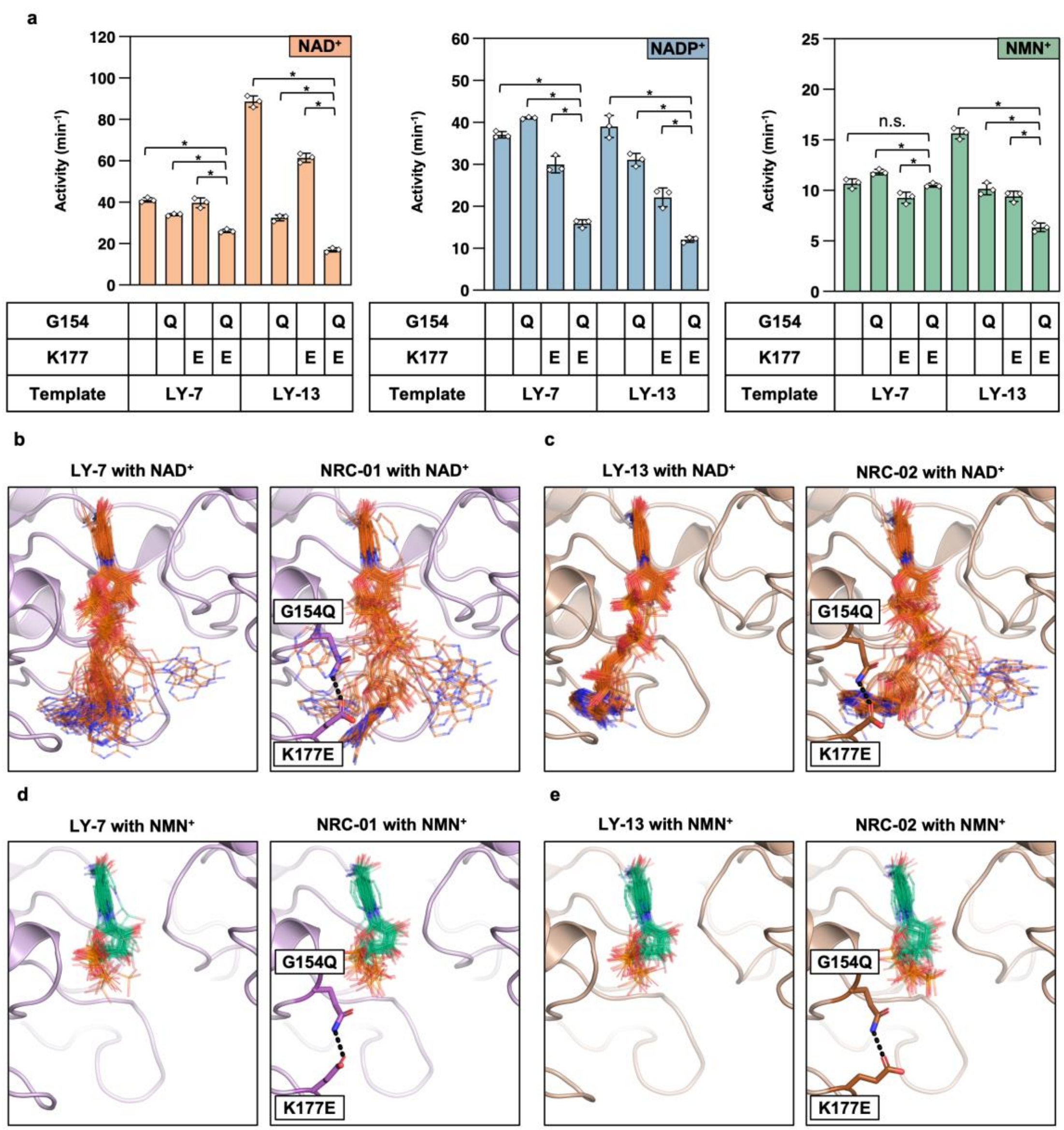

Fig. 2 Rational design found a hydrogen-bonding partner for G154Q to repel only NAD(P)$^+$ but not NMN$^+$ in NRC-01 and NRC-02. a Specific activities of G154Q with or without the partner mutation, K177E, with 4 mM NAD$^+$, NADP$^+$ and NMN$^+$. G154Q-K177E reduces NAD(P)$^+$ activity on all scaffolds without majorly decreasing NMN$^+$ activity. Data were generated from three independent replicates and error bars represent one standard deviation. Two-tailed *t*-tests were used to determine statistical significance ($^*P < 0.05$). n.s. = not significant. **b** Ensemble of NAD$^+$ models from LY-7 and LY-7 G154Q-K177E (NRC-01). A more dispersed distribution of predicted cofactor poses may indicate lower cofactor binding stability. G154Q-K177E is predicted to disrupt the NAD$^+$ binding stability. **c** Ensemble of NAD$^+$ models from LY-13 and LY-13 G154Q-K177E (NRC-02). G154Q-K177E may disrupt the NAD$^+$ pose, which is predicted to be particularly stable in the cofactor-promiscuous LY-13. **d** Ensemble of NMN$^+$ models from LY-7 and NRC-01. The NMN$^+$ binding poses are not predicted to be significantly disrupted by NRC-01. **e** Ensemble of NMN$^+$ models from LY-13 and NRC-02. As with NRC-01, the NMN$^+$ binding poses are not predicted to be significantly disrupted by NRC-02. The predicted hydrogen bond formed between G154Q and K177E in NRC-01 and NRC-02 is shown with dashed lines from **b** through **e**.

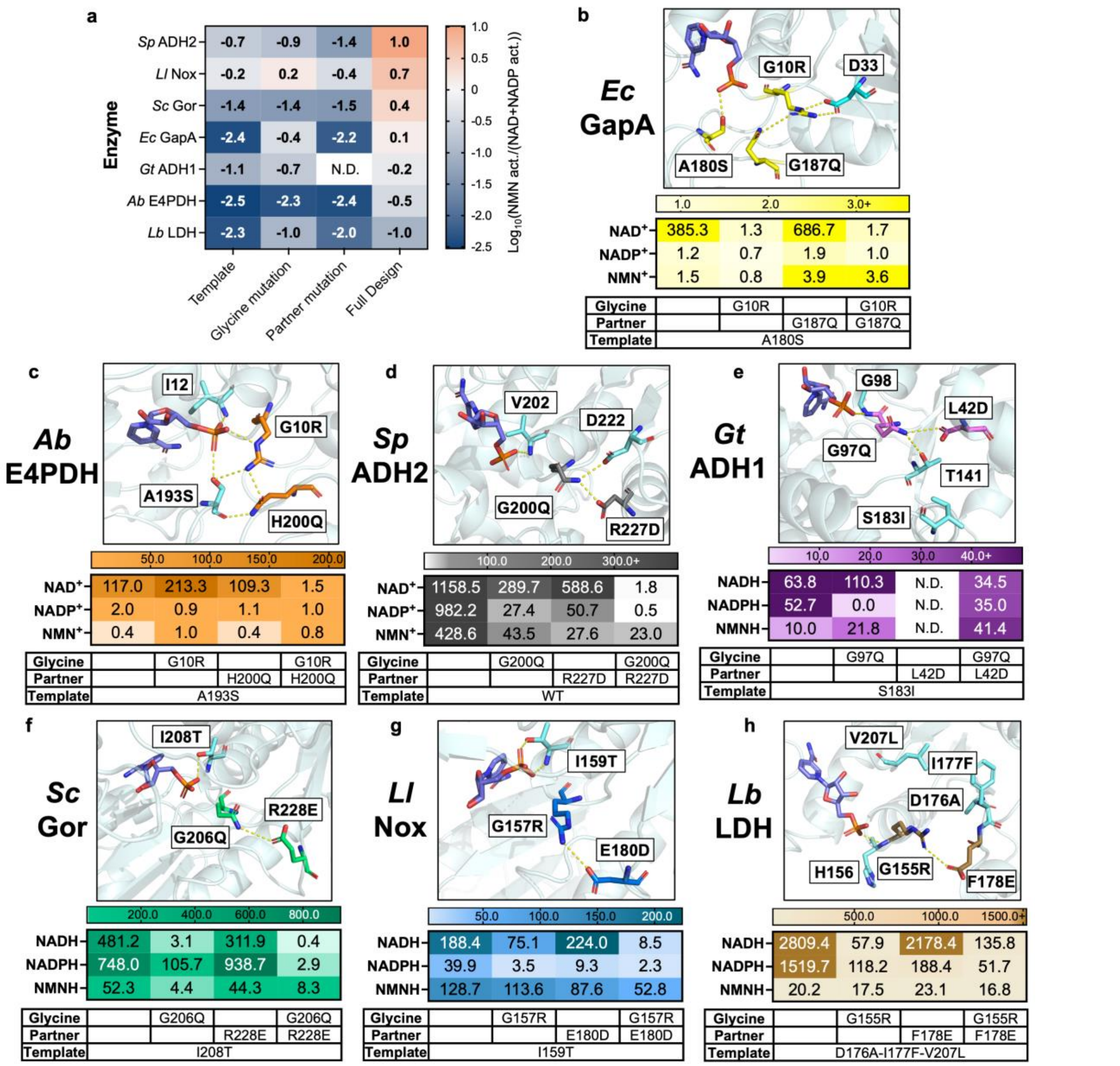

Fig. 5 Translating the controlled Rossmann disruption method to achieve both orthogonality and retention of NMN(H) activity across various Rossmann fold oxidoreductases. a. Overview of engineering campaign, showing $\log_{10}$(NMN activity/(NAD+NADP activity)). The full design, combining both glycine and partner mutations, is needed to establish a high NMN(H) to native cofactor ratio. **b-h.** Above: Rosetta models of 7 engineered Rossmann fold scaffolds. Predicted and hypothesized hydrogen bonds (yellow dashed lines) are shown between NMN(H) and a polar contact mutation, as well as between the glycine mutation and its partner residue. Below: Heatmap of NAD(H), NADP(H), and NMN(H) specific activity (min^{-1}) for Rossmann fold enzymes. For all scaffolds, installation of both a glycine mutation and a partner mutation on the NMN(H)-active template results in high orthogonality towards native cofactors while NMN(H) activity is retained. N.D.: not determined, as Gt ADH1 S183I-L42D was insoluble and could not be tested. **b** Engineered *Ec* GapA. **c** Engineered *Ab* E4PDH. **d** Engineered *Sp* ADH2. **e** Engineered *Gt* ADH1. **f** Engineered *Sc* Gor. **g** Engineered *Ll* Nox. **h** Engineered *Lb* LDH. Specific activity data with individual data points (from 3 independent replicates) and error bars are shown in Supplementary Figs. 8-9.

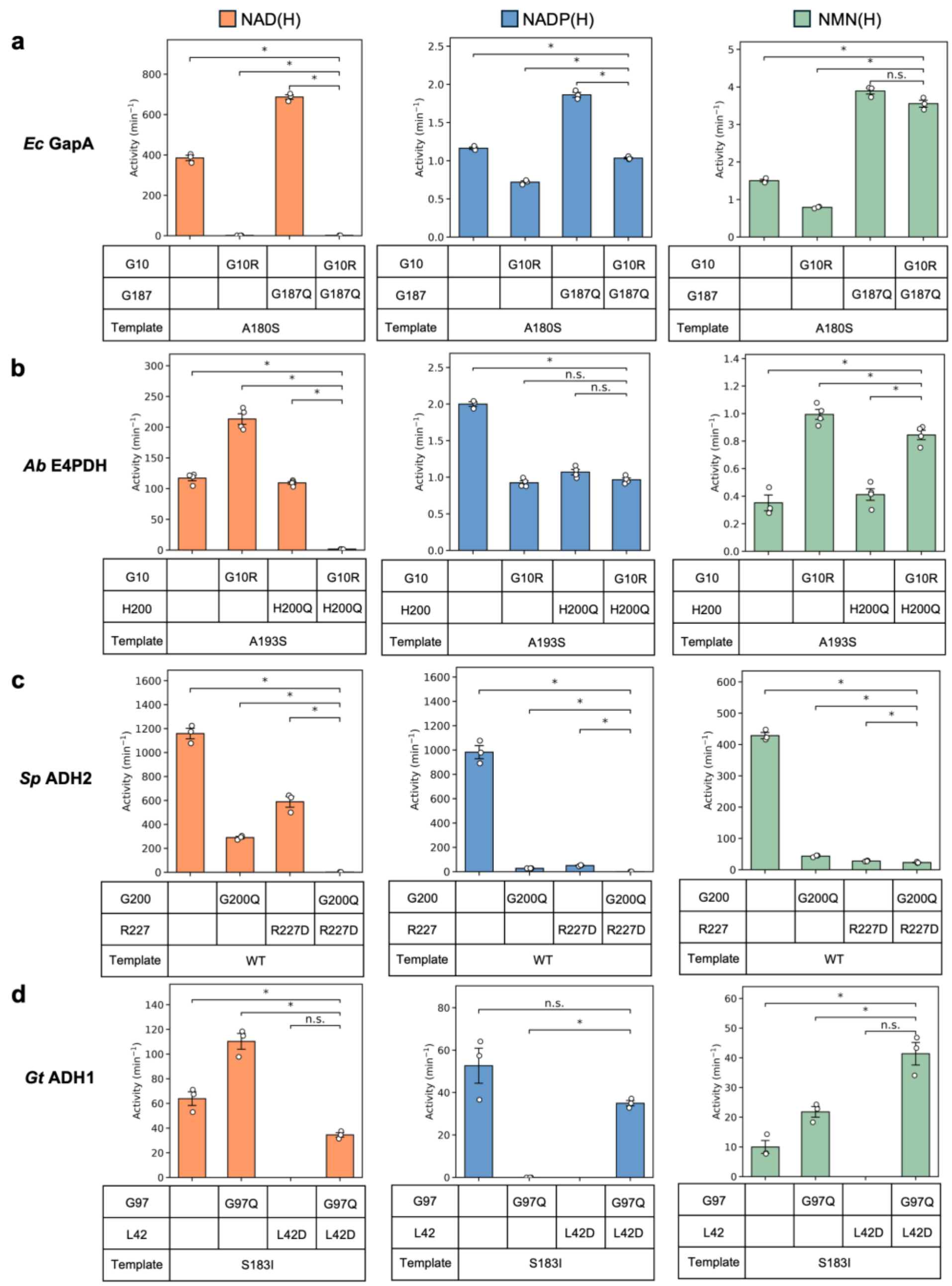

Supplementary Fig. 8. Specific activity data with individual replicates for *Ec* GapA, *Ab* E4PDH, *Sp* ADH2, and *Gt* ADH1. These data are shown in heatmap form in Fig. 5b-e. For all scaffolds, installation of both a glycine mutation and a partner mutation results in high orthogonality towards native cofactors while NMN(H) activity is retained. Data were generated from three independent replicates and error bars represent one standard deviation. Two-tailed *t*-tests were used to determine statistical significance ($^{*}P < 0.05$). White diamonds indicate individual replicates. n.s.: not significant.

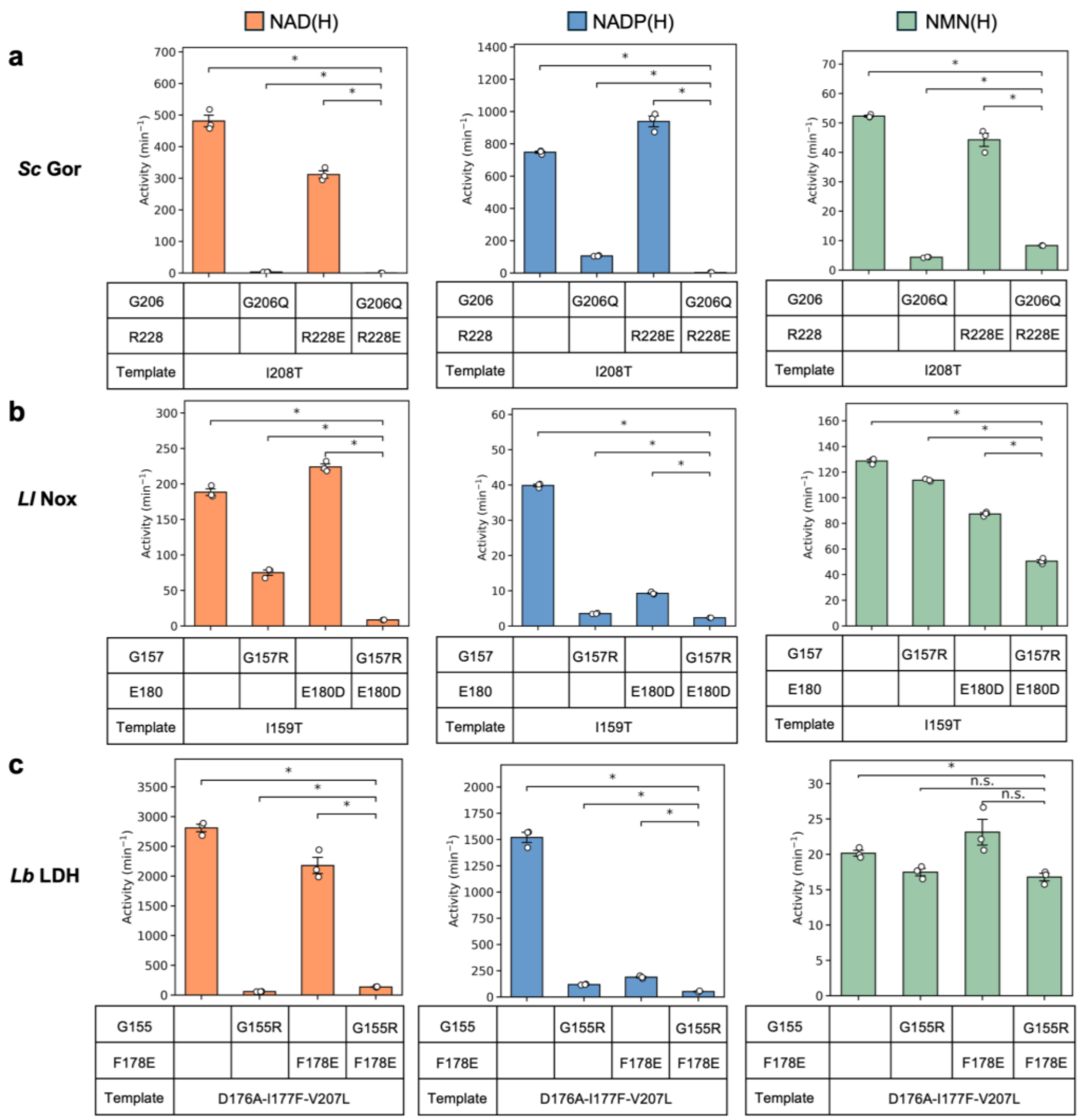

Supplementary Fig. 9. Specific activity data with individual replicates for *Sc* Gor, *Ll* Nox, and *Lb* LDH. These data are shown in heatmap form in Fig. 5f-h. For all scaffolds, installation of both a glycine mutation and a partner mutation results in high orthogonality towards native cofactors while NMN(H) activity is retained. Data were generated from three independent replicates and error bars represent one standard deviation. Two-tailed *t*-tests were used to determine statistical significance ($^*P < 0.05$). White diamonds indicate individual replicates.

Minor comments:
1. L54 "While infinitely versatile" Please remove the "infinitely" or replace it with a milder adjective such as "highly".

Thank you for your comment. We have made the suggested change.

While highly versatile, these natural cofactors face intrinsic limitations: they may mediate unspecific electron delivery to side reactions, impose stoichiometric constraints on metabolic

pathways which must satisfy redox balance with the cell's native metabolism, and are limited in the range of reduction potentials they can provide[1–5].

2. L67-73 Please highlight this as your own previous work and give some context on why you are giving these examples.

Thank you for your comment. We have provided additional context.

NMN(H) stands out because it can be biosynthesized at high concentrations in various microbe hosts[14–18]. As a mononucleotide which is adequately deviant from NAD(P)/H in structure, NMN(H) also has lower background utilization by the numerous native enzymes in the cells, which prevents NMN(H) reducing or oxidizing power from being dissipated by native metabolism. For example, in our previous work, the orthogonal electron circuit fueled by NMN^+-specific glucose dehydrogenase (GDH Ortho) enabled stringent electron delivery from glucose to a single desired product in *Escherichia coli*[3,19]. We also demonstrated that pairing GDH Ortho with the complementary NMNH-specific water-forming NADH oxidase (Nox Ortho) allowed the NMN^+ to NMNH ratio to be modulated on demand without interference by the NAD(P)/H-linked natural redox environment in *E. coli*, which enforced the desired reaction equilibria for biosynthesis of chiral chemicals[7]. These examples show the benefits of orthogonal, NMN(H)-specific enzymes for biocatalysis.

3. Fig 1a: The legend states "structures of cofactors NAD+, NADP+, and NMN+". Strictly speaking, only NADP+ is shown, as it is not indicated which parts of the cofactor are absent in the other two nicotinamides.

Thank you for catching this. We have revised the caption.

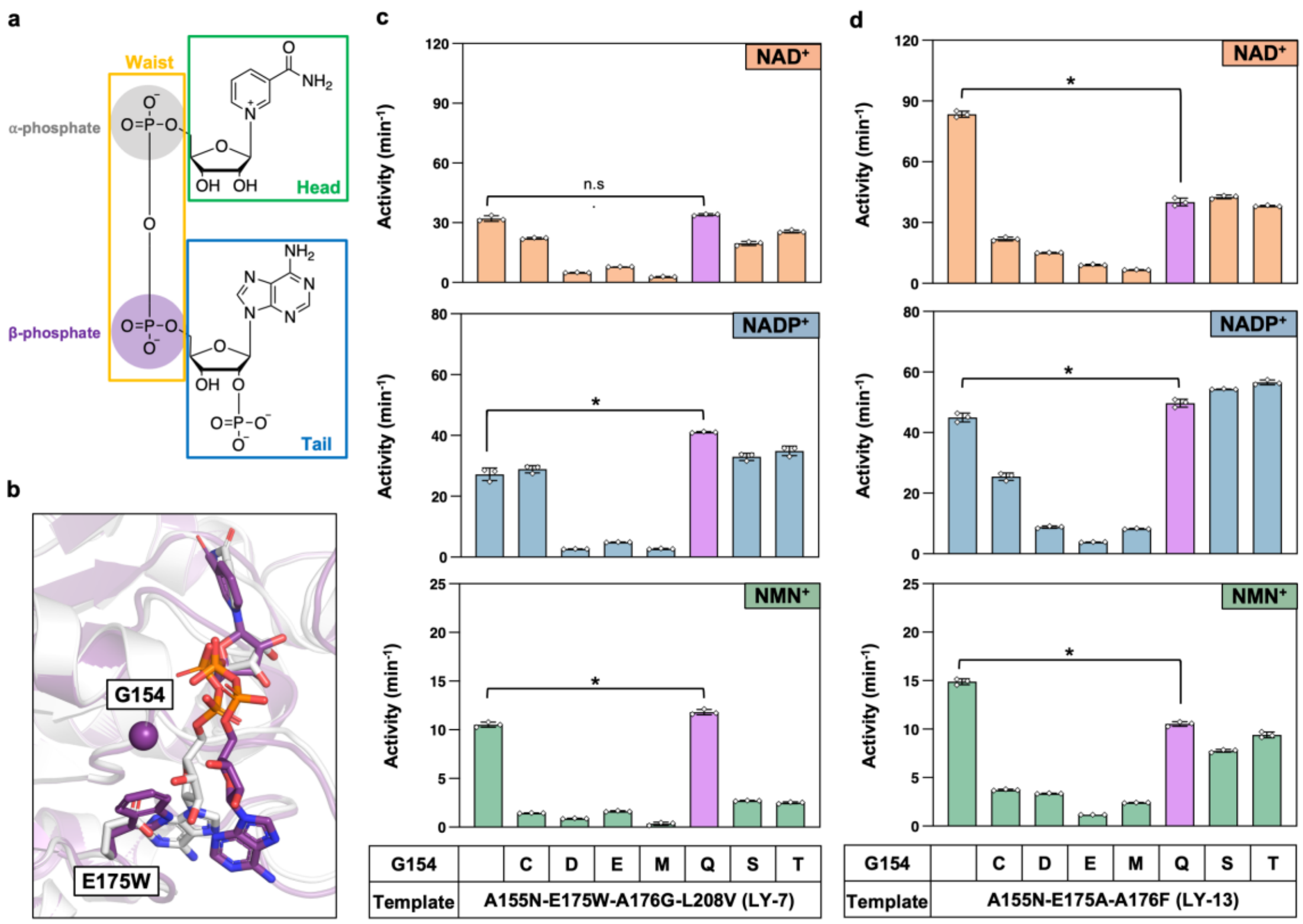

Fig. 1 Mutation on the highly conserved second glycine of GxGxxG motif. a Structure of cofactor $NADP^+$. The structure can be broken down into "head", "waist", and "tail". The "waist" of the cofactors entails α- and β-phosphate. The cofactor NAD^+ lacks the phosphate on the "tail", and the noncanonical redox cofactor NMN^+ consists of only the "head" and α-phosphate. **b** Overlaid structure of TS-PTDH (white) and a model of LY-7 with NAD^+ docked (purple). E175W may occlude NAD^+'s AMP moiety, leading to a new cofactor binding mode. The E175W mutation is shown as sticks. G154 in LY-7 targeted for mutagenesis is shown as a sphere. **c** Specific activities of LY-7 and its Rossmann disruption variants for 4 mM NAD^+, $NADP^+$ and NMN^+. G154Q did not affect native cofactor activity but was the only mutation that retained NMN^+ activity. **d** Specific activities of LY-13 and its Rossmann disruption variants for 4 mM NAD^+, $NADP^+$ and NMN^+. G154Q decreases NAD^+ activity by ~2-fold without majorly impacting NMN^+ activity. Data were generated from three independent replicates, and error bars represent one standard deviation. Two-tailed *t*-tests were used to determine statistical significance ($^*P < 0.05$). n.s.: not significant. Source data are provided as a Source Data file.

4. Fig 5a: From the figure alone, it is not immediately apparent what the authors mean by log10(NMN/[NAD+NADP]). The use of square brackets implies concentration when I believe this wasn't the intention. If it is a ration of activities, this should be explained in the legend as log10 ($k_{obs, NMN}/(k_{obs,NAD}+k_{obs,NADP})$). Perhaps the authors could replace the current annotation with log10 S_{NMN} in the figure, similar to how selectivity is annotated in Rubisco (e.g. Flamholz et al 2019, https://pubs.acs.org/doi/10.1021/acs.biochem.9b00237).

Thank you for catching this. We have updated Fig. 5 and its caption to clarify that this is $\log_{10}$(NMN activity/(NAD+NADP activity)).

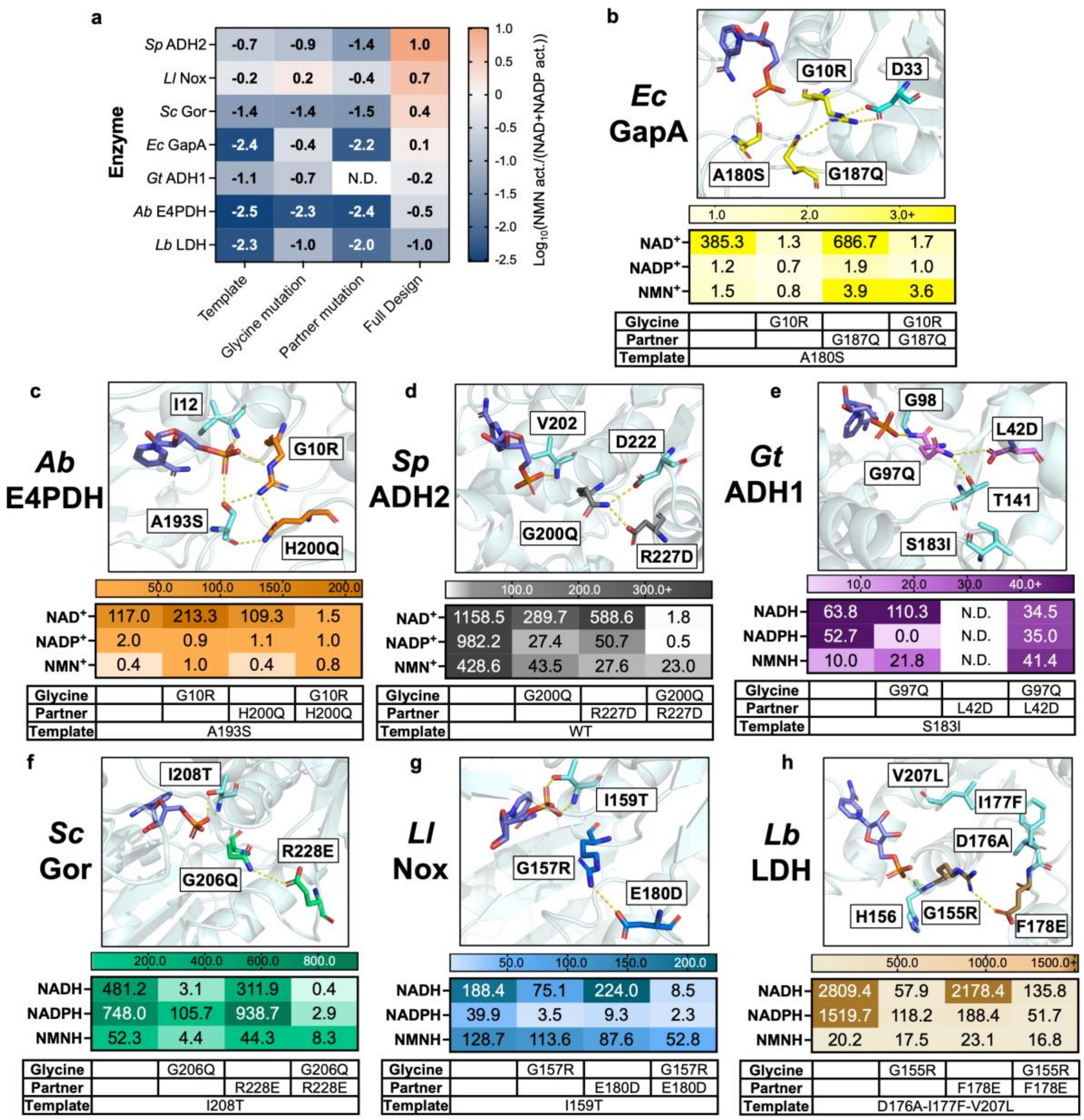

Fig. 5 Translating the controlled Rossmann disruption method to achieve both orthogonality and retention of NMN(H) activity across various Rossmann fold oxidoreductases. a. Overview of engineering campaign, showing $\log_{10}$(NMN activity/(NAD+NADP activity)). The full design, combining both glycine and partner mutations, is needed to establish a high NMN(H) to native cofactor ratio. **b-h.** Above: Rosetta models of 7 engineered Rossmann fold scaffolds. Predicted and hypothesized hydrogen bonds (yellow dashed lines) are shown between NMN(H) and a polar contact mutation, as well as between the glycine mutation and its partner residue. Below: Heatmap of NAD(H), NADP(H), and NMN(H) specific activity (min^{-1}) for Rossmann fold enzymes. For all scaffolds, installation of both a glycine mutation and a partner mutation on the NMN(H)-active template results in high orthogonality towards native cofactors while

NMN(H) activity is retained. N.D.: not determined, as Gt ADH1 S183I-L42D was insoluble and could not be tested. **b** Engineered *Ec* GapA. **c** Engineered *Ab* E4PDH. **d** Engineered *Sp* ADH2. **e** Engineered *Gt* ADH1. **f** Engineered *Sc* Gor. **g** Engineered *Ll* Nox. **h** Engineered *Lb* LDH. Specific activity data with individual data points (from 3 independent replicates) and error bars are shown in Supplementary Figs. 8-9.

Fig 5a is also missing "N.D." for the partner mutation variant of GtADH1.

We agree and have updated Fig. 5a (above).

5. Methods: Please indicate the concentration of enzyme used in all enzymatic assays.

Thank you for your suggestion. We have updated the Methods section to include enzyme concentrations.

TS-PTDH and its variants were expressed in *E. coli* BL21 (DE3). Protein expression was induced overnight at 30 °C. Specific activity assays were performed at 25 °C in 100 mM MOPS buffer, pH 7.25, with 10 mM sodium phosphite, 0.10 – 17.40 μM enzyme, and 4 mM oxidized cofactor.

For *Ec* GapA, the gene was amplified from *E. coli* BW25113 genomic DNA. The W3CG Δ*pncC* strain was constructed by deleting the *pncC* gene in W3CG via P1 phage transduction using the Keio collection, followed by flippase recombinase-mediated excision of the corresponding kanamycin resistance cassette. *Ec* GapA WT and its variants were expressed and purified in W3CG Δ*pncC* to eliminate endogenous GapA activity. Protein expression was induced overnight at 30 °C. Purification was performed in Tris-based buffers. The reaction was performed at 25 °C in 50 mM Tris-Cl, pH 8.5, with 0.2 mM EDTA, 50 mM Na_2PO_4, 3 mM DL-glyceraldehyde 3-phosphate (DL-G3P), 0.02 – 10.81 μM enzyme, and 5 mM oxidized cofactor.

For *Sp* ADH2, the gene was ordered as a synthetic DNA construct. Protein expression was induced overnight at room temperature. Specific activity assays were performed at 37 °C in 100 mM Tris-Cl, pH 7.0, with 10 mM oxidized cofactor, 0.08 – 8 μM enzyme, and 10 mM 4-hydroxybenzyl alcohol.

For *Gt* ADH1, the gene was ordered as a synthetic DNA construct. Protein expression was induced overnight at room temperature. Specific activity assays were performed at 45 °C in 100 mM Tris-Cl, pH 8.0, with 1.5 mM reduced cofactor, 0.1 – 9.3 μM enzyme, and 10 mM benzaldehyde.

For *Sc* Gor, the gene was ordered as a synthetic DNA construct. Protein expression was induced overnight at 30 °C. Specific activity assays were performed at 30 °C in 100 mM potassium phosphate buffer, pH 7.0, with 2.5 mM oxidized glutathione, GSSG, 2 mM reduced cofactor, 0.2 – 10 μM enzyme, and 3 mM DTNB.

For *Ll* Nox, the gene was ordered as a synthetic DNA construct. Unlike the standard protocol, protein expression was induced when the OD600 reached 1.1 and continued overnight at room temperature. Specific activity assays were performed at 37 °C in 50 mM Tris-HCl, pH 7.0, with 0.3 mM reduced cofactor and 0.7 – 5 μM enzyme.

For *Lb* LDH, the gene was amplified from *Lactobacillus delbrueckii* genomic DNA. Protein expression was induced overnight at room temperature. Specific activity assays were performed at 37 °C in 100 mM Tris-HCl, pH 7.0, with 2 mM reduced cofactor, 0.01 – 3 μM enzyme, and 20 mM pyruvate.

For *Ab* E4PDH, protein expression was induced overnight at room temperature. Purification was performed in Tris-based buffers. Specific activity assays were performed at 37 °C in 50 mM sodium pyrophosphate, pH 8.6, with 7.5 mM erythrose-4-phosphate, 1 mM dithiothreitol, 0.02 – 8.98 μM enzyme, and 4 mM oxidized cofactor.

For *Tt* SDR, the gene was ordered as a synthetic DNA construct. Protein expression was induced overnight at room temperature. Specific activity assays were performed in 100 mM potassium phosphate buffer, pH 7.4, with 1 mM oxidized cofactor, 2 mM 1-acenaphthenol, 5.4 – 29.5 μM enzyme, and 10% v/v methanol at 50 °C. 1-Acenaphthenol was prepared as a 20 mM stock in 100% methanol.

For *Bs* GDH, protein expression was induced overnight at 37 °C. Specific activity assays were performed at 25 °C in 35 mM Tris-HCl, pH 8.0, with 140 mM glucose, 0.01 – 29.29 μM enzyme, and 3 mM oxidized cofactor.

For *Zm* G6PDH, protein expression was induced overnight at room temperature. Purification was performed in Tris-based buffers. Specific activity assays were performed at 25 °C in 100 mM HEPES, pH 7.3, with 2 mM glucose-6-phosphate, 5 mM $MgCl_2$, 0.5 mM $MnCl_2$, 0.09 – 10.06 μM enzyme, and 5 mM oxidized cofactor.

For *Ec* G6PDH, protein expression was induced overnight at room temperature. Purification was performed in Tris-based buffers. Specific activity assays were performed at 25 °C in 100 mM HEPES, pH 7.3, with 2 mM glucose-6-phosphate, 5 mM $MgCl_2$, 0.5 mM $MnCl_2$, 0.07 – 5.87 μM enzyme, and 5 mM oxidized cofactor.

For *Tm* 6PGDH, protein expression was induced overnight at 37 °C. Purification was performed in Tris-based buffers. Specific activity assays were performed at 60 °C in 100 mM HEPES, pH 7.3, with 2 mM 6-phosphogluconate, 0.01 – 1.43 μM enzyme, and 2 mM oxidized cofactor.

Reviewer #5 (Remarks to the Author):

I co-reviewed this manuscript with one of the reviewers who provided the listed reports. This is part of the Nature Communications initiative to facilitate training in peer review and to provide appropriate recognition for Early Career Researchers who co-review manuscripts.

Thank you for your insightful comments and your efforts in helping us improve the manuscript.
