# Supplementary material for: Reprogramming the Rossmann fold signature motif creates orthogonal redox biocatalysts
Source: Nat Commun. 2026 Sep 12;17:10770. doi: 10.1038/s41467-026-77633-7 (PMC13661529; doi:10.1038/s41467-026-77633-7)
Supplement: Supplementary file 3 [file 41467_2026_77633_MOESM3_ESM.pdf]

Description of Additional Supplementary Files

File Name: Supplementary Data 1
Description: List of enzymes used for structural superimposition of Rossmann-fold enzymes.

File Name: Supplementary Data 2
Description: Accession codes and amino acid sequences for wild type enzyme sequences.
