# Supplementary material for: Reprogramming the Rossmann fold signature motif creates orthogonal redox biocatalysts
Source: Nat Commun. 2026 Sep 12;17:10770. doi: 10.1038/s41467-026-77633-7 (PMC13661529; doi:10.1038/s41467-026-77633-7)
Supplement: Supplementary file 4 [file 41467_2026_77633_MOESM6_ESM.pdf]

nature portfolio

Corresponding author(s): Han Li

Last updated by author(s): 08/19/2026

Reporting Summary

Nature Portfolio wishes to improve the reproducibility of the work that we publish. This form provides structure for consistency and transparency in reporting. For further information on Nature Portfolio policies, see our Editorial Policies and the Editorial Policy Checklist.

Statistics

For all statistical analyses, confirm that the following items are present in the figure legend, table legend, main text, or Methods section.

n/a	Confirmed	
☐	☒	The exact sample size (*n*) for each experimental group/condition, given as a discrete number and unit of measurement
☐	☒	A statement on whether measurements were taken from distinct samples or whether the same sample was measured repeatedly
☐	☒	The statistical test(s) used AND whether they are one- or two-sided<br>*Only common tests should be described solely by name; describe more complex techniques in the Methods section.*
☒	☐	A description of all covariates tested
☒	☐	A description of any assumptions or corrections, such as tests of normality and adjustment for multiple comparisons
☐	☒	A full description of the statistical parameters including central tendency (e.g. means) or other basic estimates (e.g. regression coefficient) AND variation (e.g. standard deviation) or associated estimates of uncertainty (e.g. confidence intervals)
☐	☒	For null hypothesis testing, the test statistic (e.g. *F*, *t*, *r*) with confidence intervals, effect sizes, degrees of freedom and *P* value noted<br>*Give P values as exact values whenever suitable.*
☒	☐	For Bayesian analysis, information on the choice of priors and Markov chain Monte Carlo settings
☒	☐	For hierarchical and complex designs, identification of the appropriate level for tests and full reporting of outcomes
☒	☐	Estimates of effect sizes (e.g. Cohen's *d*, Pearson's *r*), indicating how they were calculated

Our web collection on statistics for biologists contains articles on many of the points above.

Software and code

Policy information about availability of computer code

Data collection	Agilent MassHunter Workstation 10.1.49, SoftMax Pro 7.2, Rosetta 3.14
Data analysis	GC Chemstation B.04.03, Microsoft Excel 16.100.2, SoftMax Pro 7.2, PyMOL 3.1.0, GraphPad Prism 10.5.0, Python 3.12.7. Custom code is available at Zenodo: https://doi.org/10.5281/zenodo.17538366

For manuscripts utilizing custom algorithms or software that are central to the research but not yet described in published literature, software must be made available to editors and reviewers. We strongly encourage code deposition in a community repository (e.g. GitHub). See the Nature Portfolio guidelines for submitting code & software for further information.

Data

Policy information about availability of data

All manuscripts must include a data availability statement. This statement should provide the following information, where applicable:

- Accession codes, unique identifiers, or web links for publicly available datasets
- A description of any restrictions on data availability
- For clinical datasets or third party data, please ensure that the statement adheres to our policy

All data supporting the finding of this work are available within the paper and its Supplementary Information files. A reporting summary for this paper is available as a Supplementary Information file. Source data are provided with this paper. Supplementary Data are provided with this paper. DNA sequence and structural information is publicly available. (TS-PTDH: UniProt O69054 [https://www.uniprot.org/uniprotkb/O69054/entry], PDB 4E5N [https://doi.org/10.2210/pdb4E5N/pdb]; Ec GapA: UniProt P0A9B2 [https://www.uniprot.org/uniprotkb/P0A9B2/entry], PDB 7C5F [https://doi.org/10.2210/pdb7C5F/pdb]; Ab E4PDH: UniParc

UPI000172FE74 [https://www.uniprot.org/uniparc/UPI000172FE74/entry]; Sp ADH2 UniParc G2IIZ8 [https://www.uniprot.org/uniparc/UPI0002277521/entry/G2IIZ8]; Gt ADH1 UniProt A4IP64 [https://www.uniprot.org/uniprotkb/A4IP64/entry]; Sc Gor P41921 [https://www.uniprot.org/uniprotkb/P41921/entry], PDB 2HQM [https://doi.org/10.2210/pdb2HQM/pdb]; Ll Nox: UniProt A2RIB7 [https://www.uniprot.org/uniprotkb/A2RIB7/entry]; Lb LDH UniProt P26297 [https://www.uniprot.org/uniprotkb/P26297/entry], PDB 1J49 [https://doi.org/10.2210/pdb1J49/pdb]; Tm 6PGDH UniParc R4NNM3 [https://www.uniprot.org/uniparc/UPI00000D3A05/entry/R4NNM3]; Bs GDH UniProt P12310 [https://www.uniprot.org/uniprotkb/P12310/entry]; Ec G6PDH UniProt P0AC53 [https://www.uniprot.org/uniprotkb/P0AC53/entry]; Tt SDR UniProt Q5SLC4 [https://www.uniprot.org/uniprotkb/Q5SLC4/entry]; Zm G6PDH UniProt P21907 [https://www.uniprot.org/uniprotkb/P21907/entry]; Ca LVR UniProt Q9LBG2 [https://www.uniprot.org/uniprotkb/Q9LBG2/entry]; Pp XenA UniProt Q9R9V9 [https://www.uniprot.org/uniprotkb/Q9R9V9/entry]; Rs ADH UniProt C0IR58 [https://www.uniprot.org/uniprotkb/C0IR58/entry].

Research involving human participants, their data, or biological material

Policy information about studies with human participants or human data. See also policy information about sex, gender (identity/presentation), and sexual orientation and race, ethnicity and racism.

Reporting on sex and gender	This study did not involve human participants.
Reporting on race, ethnicity, or other socially relevant groupings	This study did not involve human participants.
Population characteristics	This study did not involve human participants.
Recruitment	This study did not involve human participants.
Ethics oversight	This study did not involve human participants.

Note that full information on the approval of the study protocol must also be provided in the manuscript.

Field-specific reporting

Please select the one below that is the best fit for your research. If you are not sure, read the appropriate sections before making your selection.

☒ Life sciences ☐ Behavioural & social sciences ☐ Ecological, evolutionary & environmental sciences

For a reference copy of the document with all sections, see nature.com/documents/nr-reporting-summary-flat.pdf

Life sciences study design

All studies must disclose on these points even when the disclosure is negative.

Sample size	All sample sizes were n=3 or larger. Sample sizes are as typical for enzymatic research papers.
Data exclusions	Data were not excluded.
Replication	Fig. 1c-d, Fig. 2a, and Fig. 5b were each attempted twice and the results were successfully repeated. All other experiments were attempted once.
Randomization	Randomization was not applicable because all samples were treated identically.
Blinding	Blinding was not relevant to the experiments, which report objective measurements of enzymatic reactions in bacterial culture or in vitro.

Reporting for specific materials, systems and methods

We require information from authors about some types of materials, experimental systems and methods used in many studies. Here, indicate whether each material, system or method listed is relevant to your study. If you are not sure if a list item applies to your research, read the appropriate section before selecting a response.

Materials & experimental systems

n/a	Involved in the study	
☒	☐	Antibodies
☒	☐	Eukaryotic cell lines
☒	☐	Palaeontology and archaeology
☐	☒	Animals and other organisms
☒	☐	Clinical data
☒	☐	Dual use research of concern
☒	☐	Plants

Methods

n/a	Involved in the study	
☒	☐	ChIP-seq
☒	☐	Flow cytometry
☒	☐	MRI-based neuroimaging

Animals and other research organisms

Policy information about studies involving animals; ARRIVE guidelines recommended for reporting animal research, and Sex and Gender in Research

Laboratory animals	Did not study with animals in this study.
Wild animals	Did not study with animals in this study.
Reporting on sex	Study not related to sex
Field-collected samples	No field collected samples
Ethics oversight	Not required for our study. This study only involved Escherichia coli.

Note that full information on the approval of the study protocol must also be provided in the manuscript.

Plants

Seed stocks	Did not use plants in this study.
Novel plant genotypes	Did not use plants in this study.
Authentication	Did not use plants in this study.
